# Multitask fMRI Data Classification via Group-Wise Hybrid Temporal and Spatial Sparse Representations

**DOI:** 10.1523/ENEURO.0478-21.2022

**Published:** 2022-06-03

**Authors:** Limei Song, Yudan Ren, Yuqing Hou, Xiaowei He, Huan Liu

**Affiliations:** 1 School of Information Science & Technology, Northwest University, Xi’an, 710127, China; 2 School of Computer Science and Communication Engineering, Jiangsu University, Jiangsu, 212013, China

**Keywords:** multitask classification, task-based fMRI, group-wise, hybrid temporal and spatial sparse representations

## Abstract

Task-based functional magnetic resonance imaging (tfMRI) has been widely used to induce functional brain activities corresponding to various cognitive tasks. A relatively under-explored question is whether there exist fundamental differences in fMRI signal composition patterns that can effectively classify the task states of tfMRI data, furthermore, whether there exist key functional components in characterizing the diverse tfMRI signals. Recently, fMRI signal composition patterns of multiple tasks have been investigated via deep learning models, where relatively large populations of fMRI datasets are indispensable and the neurologic meaning of their results is elusive. Thus, the major challenges arise from the high dimensionality, low signal-to-noise ratio, interindividual variability, a small sample size of fMRI data, and the explainability of classification results. To address the above challenges, we proposed a computational framework based on group-wise hybrid temporal and spatial sparse representations (HTSSR) to identify and differentiate multitask fMRI signal composition patterns. Using relatively small cohorts of Human Connectome Project (HCP) tfMRI data as test-bed, the experimental results demonstrated that the multitask of fMRI data can be successfully classified with an average accuracy of 96.67%, where the key components in differentiating the multitask can be characterized, suggesting the effectiveness and explainability of the proposed method. Moreover, both task-related components and resting-state networks (RSNs) can be reliably detected. Therefore, our study proposed a novel framework that identifies the interpretable and discriminative fMRI composition patterns and can be potentially applied for controlling fMRI data quality and inferring biomarkers in brain disorders with small sample neuroimaging datasets.

## Significance Statement

Task-based functional magnetic resonance imaging (tfMRI) is known to be able to induce functional brain activities corresponding to various cognitive tasks. However, the neuroscience mechanism of inherent functional differences that can effectively classify the multi-tfMRI data and the key functional components in composition patterns have been rarely tapped. Our proposed framework can uncover the fundamental differences in fMRI signal composition patterns and classify the multitask fMRI data with an average accuracy of 96.67%. In addition, our framework can effectively identify the key components with greater capacity in multitask classification and disclose the underlying network mechanism of these key components.

## Introduction

Researchers have long been endeavoring to induce and decode functional brain activities using functional magnetic resonance imaging (fMRI) data (Poldrack et al., 2009; Jang et al., 2017; Rubin et al., 2017). To detect neural activations embedded in tfMRI data, various computational and statistical methods have been proposed over the past decades, where the general linear model (GLM) is the most prevailing method for tfMRI analysis (Friston, 2009; Mueller et al., 2013). Moreover, independent component analysis (ICA) is another effective approach to characterize functional brain networks (FBNs) based on an assumption of statistically independent relationships between components (Xu et al., 2013), while independence is an ideal hypothesis in mathematics. The study of the visual and auditory perceptual cortex shows that the activity of neurons is highly sparse (Wright et al., 2010). Based on this, a sparse representation algorithm has been applied and demonstrated its effectiveness for functional network identification (Olshausen and Field, 2004; Lv, 2013; Zhao et al., 2018).

While a large number of studies focus on characterizing the concurrent task-evoked brain regions/networks (Calhoun et al., 2001; Lv et al., 2015a; Darnai et al., 2019), the neuroscience of inherent functional differences in composition patterns of multitask fMRI signals has been rarely tapped. Investigating the differences between different task fMRI signals composition could improve better understanding for the organization of the brain’s cognitive functioning, and might contribute to disease diagnosis and classification. In addition, while different cognitive functions are induced by diverse task paradigms, which require functional interactions among different specialized brain regions/networks, another challenging issue rarely explored is whether there exist interpretable and distinctive spatial-temporal components in differentiating fMRI signals under different task designs.

As far as we know, there are several challenges in addressing the above questions. First, as whole-brain fMRI data generally consists of enormous amounts of voxels, group-wise tfMRI signals composed of multiple tasks and subjects have relatively high dimensionality, which inevitably causes overloaded computational burden. Therefore, we would need an efficient computational framework with the capacity of handling the high dimensionality of fMRI signals. Second, the variability and the noises in fMRI signals could be remarkable. Thus, it has been challenging to derive consistent activation patterns from whole-brain fMRI signals of multiple subjects with such a variety of noises, awaiting an effective computational framework (Mueller et al., 2013).

With the successful application of sparse representation and deep learning algorithms in fMRI signals classification studies (Zhang et al., 2016; Parhi, 2019; Wang et al., 2020), there are still some problems. For example, a previous study identified the difference between tfMRI and resting-state fMRI signals via sparse representation method, but it ignored the fundamental differences among different types of task paradigms and lacked the further investigation of the key component of classification (Zhang et al., 2016). In addition, this study derived representative spatial and temporal characteristics via subject-level sparse representation framework, where the correspondence among subjects was confirmed through manual inspection, resulting in time-consuming and laborious work. Recently, while deep learning algorithms are considered to be promising approaches to decode multitask fMRI data, there are still limitations of current studies, including the demand for a large training sample size of fMRI data, which is hard to collect for clinical populations (Litjens et al., 2017; Wen et al., 2018), manual or experimental setting of numerous hyperparameters that are time-consuming and suboptimal (Ching et al., 2018), and the moderate explainability of deep learning models (Guo et al., 2016). For example, Wang et al. (2020) reported that 1034 subjects were used to classify fMRI signals of seven tasks, where these deep learning models are hard to apply to clinical data because of limited sample size, and the neurologic meaning of their findings is elusive. Considering the above challenges and pitfalls of existing research, it is desirable for an appropriate framework that can classify multitask fMRI signals and characterize the key components which play a key role in classification with a small sample case.

In this study, we proposed a group-wise two-stage framework based on hybrid temporal and spatial sparse representations (HTSSR) to identify the intrinsic differences in tfMRI signal composition patterns. Our results demonstrated that both temporal and spatial features could be obtained group-wisely by analyzing only a small proportion (10%) of whole brain fMRI signals. Seven HCP tasks can be classified simultaneously with an average accuracy of 96.67%. Moreover, our framework cannot only effectively identify the key components that can well characterize and differentiate multitask signals, but also imply the underlying neuroscience implications of these components, offering an effective methodology for basic neuroscience and clinical research.

## Materials and Methods

### Overview

**Figure 1.**
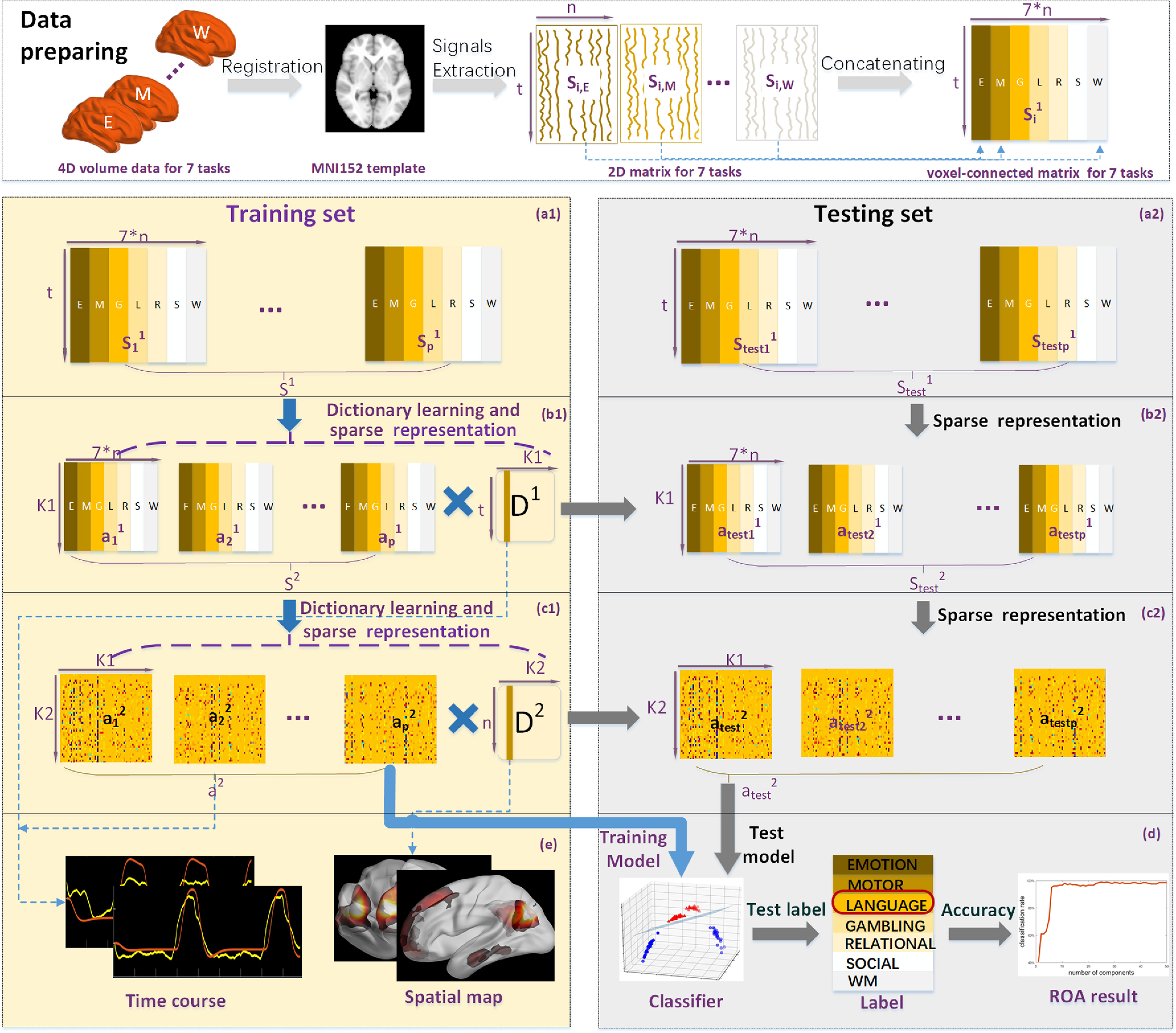
Overview of HTSSR framework and analyses, including (***a1***) training dataset, (***b1***) dictionary learning and TSR on training set to obtain ***D***^1^ and ***α***^1^, (***c1***) dictionary learning and spatial sparse representation (SSR) on training set to obtain ***D***^2^ and ***α***^2^, (***a2***) testing dataset, (***b2***) using ***D***^1^ from training stage to obtain $\alpha_{\text{test}}^{1}$ for testing set $S_{\text{test}}^{1}$, (***c2***) using ***D***^2^ from training stage to obtain $\alpha_{\text{test}}^{2}$, (***d***) training SVM using ***α***^2^ of training set and applying SVM classifier on $\alpha_{\text{test}}^{2}$ for the classification on the testing dataset (SVM-based classification), Ratio of activation (ROA)-based analysis, and key components analysis, (***e***) temporal features and representative functional networks. The asterisk represents multiplication. See also Extended Data Figure 1-1.

The overall framework (Fig. 1) consists of three stages: (1) data preprocessing and preparation; (2) training stage on training set; (3) classification and pos-hoc analyses stage on testing set. In data preprocessing and preparation, for each subject, the whole-brain fMRI data of seven different tasks were extracted and then spatially concatenated to a matrix $S_{i}^{1}$ (Fig. 1, top panel). The whole dataset of 60 subjects was randomly divided into training set and testing set, that is, we randomly selected *p* subjects from all the subjects as training set and set the rest subjects as testing set. Then, all the training subjects’ signal matrices were spatially concatenated to one large matrix ***S***^1^ for training model (Fig. 1*a1*). In temporal sparse representation (TSR) training stage, the online dictionary learning algorithm was used to factorize the large matrix ***S***^1^ of training set into groupwise time-series dictionaries ***D***^1^ and the corresponding loading coefficients ***α***^1^ (Fig. 1*b1*). Afterwards, in the spatial sparse representation (SSR) training stage, the groupwise time-series dictionaries ***D***^1^ and the corresponding loading coefficients ***α***^1^ were fed into the spatial sparse representation (SSR) training to derive the groupwise spatial dictionary ***D***^2^ and loading coefficient ***α***^2^ (Fig. 1*c1*). After the training stage, we then conducted the classification and pos-hoc analyses on testing set. Specifically, based on the group-wise time-series dictionaries ***D***^1^ and ***D***^2^ and the trained model derived from training stage, the loading coefficient ***α***1 test and ***α***2 test for testing set were obtained for classification analysis (Fig. 1*b2–c2*). Afterwards, the loading coefficient ***α***^2^ of training set was adopted to train a support vector machine (SVM) for classification, and the loading coefficient $\alpha_{\text{test}}^{2}$ was fed into the trained SVM model to obtain the label for the testing set and assess the classification performance of proposed method. Note that all the parameters used in the testing set were learned and trained by the training set. In general, we conducted the experiment above 10 times for validation. For each time, we randomly selected *p* subjects from the whole datasets as the training set and set the rest for testing, where 10% of voxels of whole brain for each subject were randomly selected for model training or testing, resulting in that the subjects in the training set and testing set were different for each experiment. Furthermore, the common dictionary ***D***^2^ contains intrinsic functional patterns, and its atoms could estimate spatial maps. By analyzing the active components in the loading coefficient ***α***^2^, the most discriminative atoms in ***α***^2^ can be selected as the key components in classification features (Fig. 1*d*). The temporal features and representative functional networks can be obtained during TSR and SSR, respectively (Fig. 1*e*).

10.1523/ENEURO.0478-21.2022.f1-1 Extended Data Figure 1-1 Truncation of six task designs. Download Figure 1-1, TIF file.

### Data acquisition and preprocessing

In this study, we adopted the seven task fMRI data in Human Connectome Project (HCP) Q1 release (Barch et al., 2013). The acquisition parameters of tfMRI data are as follows: 90 × 10^4^ matrix, 220 mm Field-of-view (FOV), 72 slices, The repetition time (TR) = 0.72s, The echo time (TE) = 33.1 ms, flip angle = 52°, bandwidth (BW) = 2290 Hz/Px, in-plane FOV = 208 × 180 mm, 2.0 mm isotropic voxels.

fMRI preprocessing pipelines included motion correction, spatial smoothing, temporal prewhitening, slice timing correction, global drift removal, and nonlinear registration into 2-mm MNI152 space using FSL FNIRT (http://www.fmrib.ox.ac.uk/fsl/feat5/index.html). Then, we used the standard MNI152 template as the mask to extract each individual tfMRI data, resulting in group-wise spatial correspondence of all the subjects. In this work, 60 subjects in the released dataset were used. More details about data acquisition and preprocessing are referred to (Barch et al., 2013).

The numbers of time points for each task are: emotion (176 frames), motor (284 frames), gambling (253 frames), language (316 frames), relational (232 frames), social (274 frames), and working memory (405 frames). As different tfMRI data have different time points and all these tfMRI data will be then imported to group-wise HTSSR model, we thus performed a truncation preprocessing to equalize the time points of each tfMRI data (176 frames; Extended Data Fig. 1-1). Inevitably, truncation preprocessing has implications in the integrity of task design. Specifically, for example, four events (2BK_BODY-1, 2BK_PLACE-3, 0BK_FACE-6, and 0BK_PLACE-7) were excluded in working memory (WM) task because of data truncation. Nevertheless, for the other tasks, the truncated tfMRI data included at least one block of all the events.

### HTSSR

Before TSR, the whole-brain fMRI signals of each subject were converted to two-dimensional matrix. Then, the matrix ***S***^1^ of the *i*-th subject included seven tasks ( $S_{i}^{1}=[S_{i,E}^{1},S_{i,M}^{1},S_{i,G}^{1},\;S_{i,L}^{1},S_{i,R}^{1},S_{i,s}^{1},S_{i,\;W}^{1}]\in R^{t\times (n\times 7)}$, where $S_{i,E}^{1}\in R^{t\times n}$ with *t* time points and *n* voxels. The seven capital subscripts represent 7 different tasks, respectively (E: emotion, M: motor, G: gambling, L: language, R: relational, S: social, and W: work memory). Each column in the matrix was normalized to have zero mean and unit norm. The whole-brain data with multitasks of all training set were spatially concatenated to compose a multisubject fMRI matrix S1=[S11,S21,...,S]p,∈Rt×(n×7×p), where *p* is the number of subjects in training set (*p* = 30; Fig. 1*a1*). As the online dictionary learning algorithm is an effective way to extract instinct information in original signals (Brett et al., 2002), the algorithm would learn a meaningful dictionary ***D*** consisting of k atoms to represent ***S*** with the corresponding sparse loading coefficient matrix ***α*** (k ≪ n). Specifically, in TSR, the online dictionary learning algorithm can be used to factorize the multisubject fMRI data ***S***^1^ into a group-wise temporal dictionary $D^{1}\in R^{t\times k1}$ and reference weight matrix $\alpha^{1}(\alpha^{1}=[\alpha_{1}^{1},\alpha_{2}^{1}...,\alpha_{p}^{1}]\in R{\;}^{\text{t}\times \left(\text{n}\times 7\times \text{p}\right)}$, $\alpha_{i}^{1}=[\alpha_{i,E}^{1},\alpha_{i,M}^{1},\alpha_{i,G}^{1},\alpha_{i,L}^{1},\alpha_{i,R}^{1},\alpha_{i,S}^{1},\alpha_{i,w}^{1}]\in R^{k1\times (n\times 7)}$.

The loss function for the dictionary learning algorithm was defined in Equation 1 with a l1 regularization that impose a sparse constraint to the loading coefficient, where λ1 is a regularization parameter which can balance the regression residual and sparsity level:

(1) min12‖S1−Dα1‖F2+λ1‖α1‖1,11.

To prevent ***D***^1^ from arbitrarily large values which leads to trivial solution of the optimization, its columns d_1_, d_2_, ……d_k_ are constrained by Equation 2:

(2) $$C≜\{D^{1}\in R{\;}^{\text{t}\times \text{k}1},.t.\forall j=\;1,\cdots ,k1\;,\;\;d_{j}^{T}d_{j}\leq 1\}.$$

To reduce the computational burden, we randomly chose only 10% whole-brain signals in each subject during learning dictionary ***D***^1^ (Liu et al., 2017). The flowchart is shown in Figure 1*b1*.

In this work, the dictionary size k1 and value of λ1 were determined experimentally and empirically (k1 = 200, λ1 = 0.05). After TSR, each atom of resulting ***D***^1^ matrix contained the temporal information in the functional brain, while the corresponding loading coefficient matrix ***α***^1^ contained the spatial distribution of each component (Fig. 1*e*).

The next major goal was to obtain groupwise spatial features that could reveal the distinctive organization patterns of the fMRI signals under different task stimulation, which was achieved in SSR. In SSR, we combined the reference weight matrices of all subjects obtained in TSR, to obtain one matrix $S^{2}(S^{2}=\;[S_{1}^{2},S_{2}^{2},...,S_{p}^{2}]\in R^{\text{t}\times \left(\text{n}\times 7\times \text{p}\right)}$, where $S^{2}=[{\left(\alpha_{i,E}^{1}\right)}^{T},{\left(\alpha_{i,M}^{1}\;\alpha_{i,G}^{1}\right)}^{T},{\left(\alpha_{i,L}^{1}\right)}^{T},{\left(\alpha_{i,R}^{1}\right)}^{T},{\left(\alpha_{i,s}^{1}\right)}^{T},{\left(\alpha_{i,w}^{1}\right)}^{T}]\in R^{\text{n}\times \left(7\times \text{k}1\;\right)}$. Then, ***S***^2^ would be served as the input for SSR to obtain a groupwise spatial dictionary $D^{2}\in R{\;}^{\text{n}\times \text{k}2}$ and the corresponding loading coefficients ***α***^2^. Note that $\alpha^{2}=\left[\alpha_{1}^{2},\alpha_{2}^{2},\cdots ,\alpha_{\text{p}}^{2}\right]\in R^{\text{k}2\times (\text{K}1\times 7\times \text{P})}$, where $\alpha_{i}^{2}=\left[{\left(\alpha_{i,\text{E}}^{2}\right)}^{\text{T}},{\left(\alpha_{i,\text{M}}^{2}\right)}^{\text{T}},{\left(\alpha_{i,\text{G}}^{2}\right)}^{\text{T}},{\left(\alpha_{i,\text{L}}^{2}\right)}^{\text{T}},{\left(\alpha_{i,\text{R}}^{2}\right)}^{\text{T}},{\left(\alpha_{i,\text{S}}^{2}\right)}^{\text{T}},{\left(\alpha_{i,\text{W}}^{2}\right)}^{\text{T}}\right]\in R^{\text{k2}\times \text{K1}\times \text{7}}$. In SSR, we set parameters experimentally and empirically as follows: *k*2 = 50, λ2 = 0.1.

To derive the loading coefficients for testing set for further classification analysis, firstly, in the TSR stage, the groupwise time-series dictionary matrix ***D***^1^ obtained during the training stage was used to represent $S_{\text{test}}^{1}$ by solving a typical l-1 regularized LASSO problem to obtain the sparse loading coefficient $\alpha_{\text{test}}^{1}$ (Fig. 1*b2*). In the SSR stage, the dictionary matrix ***D***^2^ obtained from the training stage was then used to obtain the loading coefficient $\alpha_{\text{test}}^{2}$ of testing set (Fig. 1*c2*). The acquisition of $\alpha_{i\;\text{test}}^{i}$ was the deterministic LASSO solution as the Equation 3 shows, where *i* represents 1 or 2. The values of λ1 and λ2 were set as the same way of the training stage (λ1 = 0.05, λ2 = 0.1):

(3) $$\min\frac{1}{2}\|S_{test}^{t}-D^{i}a_{test}^{t}{\|}_{F}^{2}+\lambda i\|\alpha_{\text{test}}^{i}{\|}_{1,1}.$$

The proposed HTSSR framework reduced the size of original fMRI data dramatically while maintained the intrinsic temporal and spatial information, thus the intrinsic features (the loading coefficient) we exacted via our framework can represent differences of functional brain activity patterns.

### Identification of temporal features and representative functional networks

The temporal features and functional networks can be estimated by the proposed framework. In TSR, the $S_{i,\text{t}}^{1}$ can be factorized into ***D***^1^ and ***α***^1^, where *i* represents *i*-th subjects, t represents t kind of task, t∈ Φ = {*E, M, G, L, R, S, W*}. Then, the transpose of ***α***^1^ could be factorized into ***D***^2^ and ***α***^2^ as the Equation 4 shows. So, we can obtain the Equation 5 as follows:

(4) $$S_{i,\text{t}}^{2}={\left(\alpha_{i,y}^{1}\right)}^{T}=D^{2}\times \alpha_{i,\text{t}}^{2}$$

(5) $$S_{i,t}^{1}=D^{1}\text{×}\alpha_{i,t}^{1}=D^{1}\text{ ×}{\left(D^{2}\text{ ×}\alpha_{i,t}^{2}\right)}^{T}=D^{1}\text{ ×}{\left(\alpha_{i,t}^{2}\right)}^{T}\text{×}{\left(D^{2}\right)}^{T}.$$

Considering all subjects sharing the same groupwise dictionary, the intrinsic difference of various tasks depends on the loading coefficients ***α***^2^. Further, since ***D***^2^ contained intrinsic groupwise spatial patterns, the temporal information of origin signal $S_{i,\text{t}}^{1}$ should exist in the first two items in Equation 5, that is, ***D***^1^ and ( $\alpha_{i,\text{t}}^{2}{)}^{T}$. In order to obtain the groupwise temporal pattern of various tasks, we averaged the loading coefficient ( $\alpha_{i,\text{t}}^{2}{)}^{T}$ in each subject and finally got the Equation 6. That means, task-specific temporal course is the weighted average of the loading coefficient of each subject and the group-wise common temporal dictionary:

(6) $$D_{\text{t}}=D^{1}\text{ ×}\frac{1}{p}\sum_{\left(i=1\right)}^{p}{\left(\alpha_{i,t}^{2}\right)}^{T},s.t.t\in \Phi =\{E,M,G,L,R,S,W_{}\}.$$

Based on prior task paradigms, we can obtain the Pearson correlation coefficient between the task paradigms and the task-specific temporal course, which was defined as

(7) $${\text{P}}_{\text{corr, j}}\text{=corr }(D_{\text{t, j}}\text{, TASK}).$$

Essentially, P_corr, j_ measures the temporal similarity between temporal course of the *j*-th component in ***D***_t_ and the t-task paradigms stimulus curve, where a larger value means better correspondence between the component and the stimulus. As the common dictionary ***D***^2^ contains intrinsic groupwise functional patterns derived from SSR, the atoms in ***D***^2^ could be used to define the functional spatial maps (Fig. 1*e*).

We also identified the spatial matching rate to measure the similarity between spatial patterns derived by our proposed framework and GLM-derived activation patterns. Specifically, the GLM-based activations were performed individually and group-wisely using FSL FEAT (http://www.fmrib.ox.ac.uk/fsl/feat5/index.html), and the group-level GLM-based results were used for comparison. The details of GLM analysis can be found in previous literature (Lv et al., 2015b). The overlapping rate with the template was defined quantitatively as Equation 8:

(8) $$R(X,T)=\frac{\left|X\;\cap \;T\right|\;}{\left|\text{T}\right|},$$

where *X* is the spatial functional networks of the derived component of our proposed framework and *T* is the GLM-derived activation template.

### SVM-based classification method

To classify multitask brain signals, we first trained the SVM classifier using loading coefficient ***α***^2^ derived from training set, as ***α***^2^ contained both temporal and spatial information embedded in multitasks fMRI signals. To evaluate the performance of proposed framework, we conducted multitask classification analysis on independent testing set, where the $\alpha_{\text{test}}^{2}$ was fed into the trained SVM model to derive the classification rate. Specifically, according to the true label of different seven tasks for each loading coefficient $\alpha_{\text{test}}^{2}$, the classification accuracy was calculated by the proportion of samples that are predicted correctly. The SVM classifier was established based on the LIBSVM toolbox (Chang and Lin, 2011) for multitask classification. As the number of features was relatively large, the linear kernel was selected as the SVM kernel. In addition, all the other parameters were set as default values.

### Ratio of activation (ROA)-based analysis

The final goal was to find the discriminative features for classification. Inspired by the successes of using ROA in two types of fMRI signals classification (Zhang et al., 2016), we proposed a novel ROA metric as follows: each row in loading coefficients ***α***^2^ represents the active level of the corresponding atoms in ***S***^2^, that is, to what extent the *i*-th row in loading coefficients ***α***^2^ is activated in the *j*-th atom in ***S***^2,^ and the ROA of the *i*-th loading coefficients ***α***^2^ was defined as follows:

(9) $${\text{ROA}}_{i}=\left|log\frac{1}{T}\overset{T}{\underset{t=1}{\sum }}\overset{T}{\underset{k=t+1}{\sum }}\frac{{\left|\alpha \left(\text{i},\text{j}\right)\right|}_{0},\;jth\;column\;belongs\;to\;task\left(t\right)}{{\left|\alpha \left(\text{i},\text{j}\right)\right|}_{0},\;jth\;column\;belongs\;to\;task\left(k\right)}\right|$$

In Equation 9, *T* represents task index, which refers to seven in our work. Task (1) to Task (7) represent seven different tasks (emotion, motor, gambling, language, relational, social, and work memory). The ROA was obtained by counting the number of non-zero entries of the rows in ***S***^2^ which have been labeled as seven tasks. High ROA value indicates that the corresponding atom in ***S***^2^ is highly active in specific task.

In order to verify the components with higher ROA value capture greater capacity in classifying the multitask fMRI signals, we designed an experiment as follows. After sorting the ROA values of all the components (rows in loading coefficients ***α***^2^) from high to low, we iteratively employed more rows sorted by their ROA values in ***α***^2^ as the feature inputs to train a SVM classifier, that is, the components with higher ROA values would be used to train preferentially. The corresponding components in $\alpha_{\text{test}}^{2}$ of testing set were entered into the trained SVM model to derive the classification accuracy. Here, we adopted the same classification scheme depicted above (SVM-based classification method).

### Code accessibility

The MATLAB code of HTSSR framework and ROA-based analysis described in the paper can be accessed in Extended Data 1 (MATLAB code).

10.1523/ENEURO.0478-21.2022.ed1 Extended Data 1 The code. Download Extended Data 1, ZIP file.

## Results

**Figure 2.**
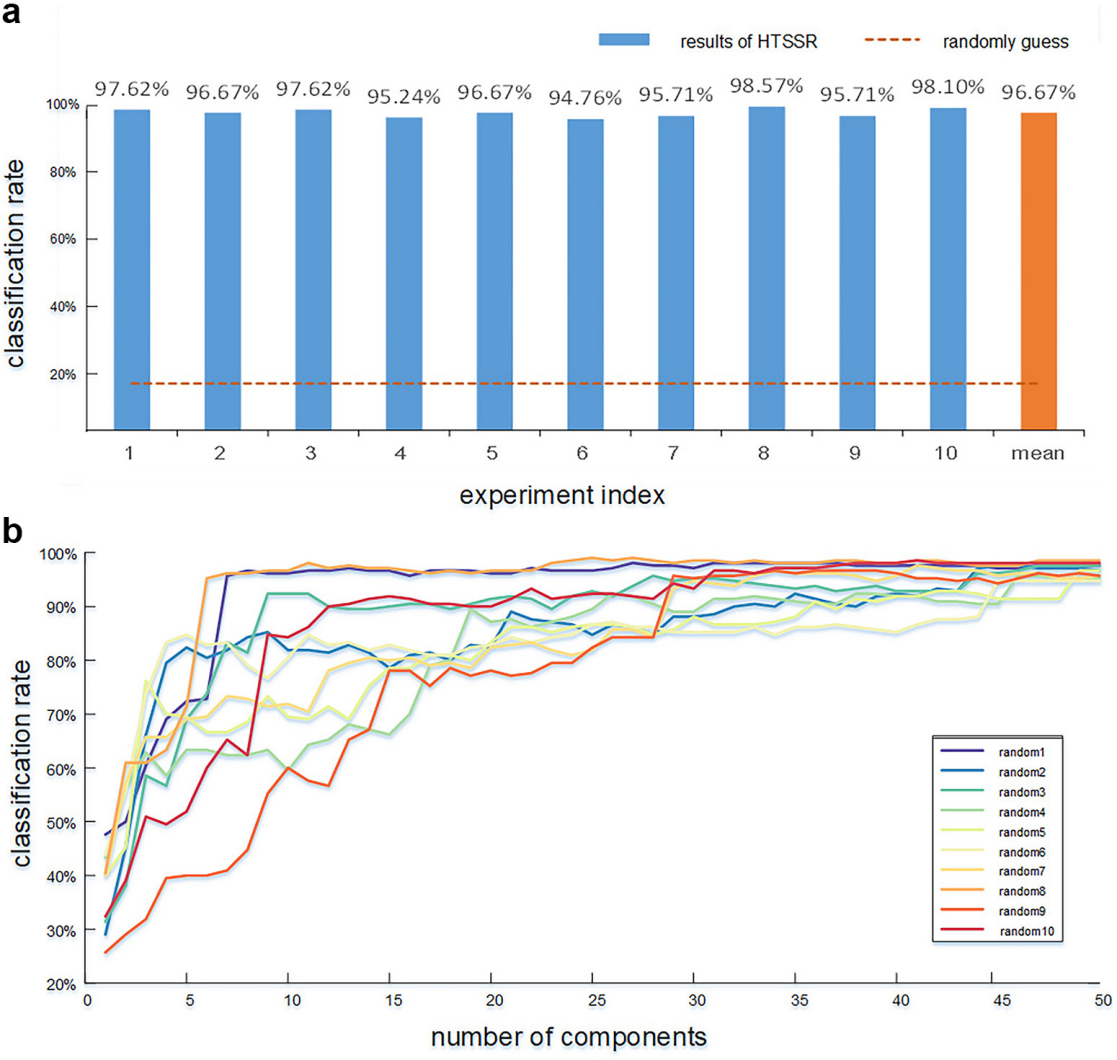
Classification analysis on testing set. ***a***, Classification accuracies for 10 independent experiments via hybrid temporal and spatial sparse representations (HTSSR) framework. The blue bar represents the classification accuracy of each experiment, and the orange bar is the average accuracy of 10 experiments. The dotted line represents the probability of random guesses (1/7 ≈ 14.29%). ***b***, Classification rate for SVM-based classification on testing dataset using different number of components sorted by their ROA values. The different colored lines represent the ROA curves for 10 independent experiments. The *x*-axis is the number of components selected for the classification, and the *y*-axis is classification accuracy. See also Extended Data Figures 2-1, 2-2.

By applying the proposed HTSSR framework to seven tfMRI data from the HCP dataset, our results revealed that all the tfMRI signals can be effectively differentiated, and the intrinsic spatial/temporal patterns underlying their fundamental differences in signal composition could be characterized by the corresponding loading coefficients ***α***^2^. Intriguingly, although we only selected a few components with high ROA values in ***α***^2^ as inputs for classification, the seven tasks can be accurately classified, and the average accuracy of 10 independent experiments on different testing sets was 96.67 ± 1.22% (mean ± SD; Fig. 2*a*). Moreover, our proposed framework cannot only classify seven tasks accurately, but also can effectively identify four types of functional components: task-evoked components, resting-state functional components, integrated functional components, and artifact components. In addition, as the resting state and artifact components were very useful in the clinical populations, we further investigated and discussed their role in multitask classification. Finally, to improve the interpretability of the classification results, we further investigated the underlying network mechanism of the classification capability of each functional component.

10.1523/ENEURO.0478-21.2022.f2-1 Extended Data Figure 2-1 Classification rate of eliminating resting state and artifact components. Download Figure 2-1, TIF file.

10.1523/ENEURO.0478-21.2022.f2-2 Extended Data Figure 2-2 Examples of functional activations derived by λ1 = 0.5. Download Figure 2-2, TIF file.

### Classification and ROA-based analysis

Our proposed framework can accurately classify seven tasks on testing set and the classification accuracy of 10 experiments ranged from 94.67% to 98.57%, with an average accuracy of 96.67% (Fig. 2*a*), demonstrating our proposed framework can effectively uncover the inherent differences in composition patterns of multitask fMRI signals. These inherent differences between tasks can be revealed by the loading coefficient, which is distinctive and descriptive enough to classify tfMRI data accurately.

As depicted above (ROA-based analysis), we iteratively fed more components from the loading coefficient $\alpha_{\text{test}}^{2}$ of testing set as the feature inputs for the SVM classifier, where these components were assorted by their ROA values independently derived from training set. The classification results of 10 independent experiments on testing set are shown in Figure 2*b*. When the number of features used reached at ten, the average accuracy of all the curves increased monotonically and can reach at 80% (55–96%), and then reached 90% with 30 atoms (85–98%). Finally, the accuracy was almost close to 100% as more components were included. As the performance curve exhibited a plateau with more than thirty components, we concluded that the additional components with smaller ROA value contribute little to the differentiation power. The results show that our method can effectively disclose the key components that play great roles in successful classification.

### Task-evoked functional components

**Figure 3.**
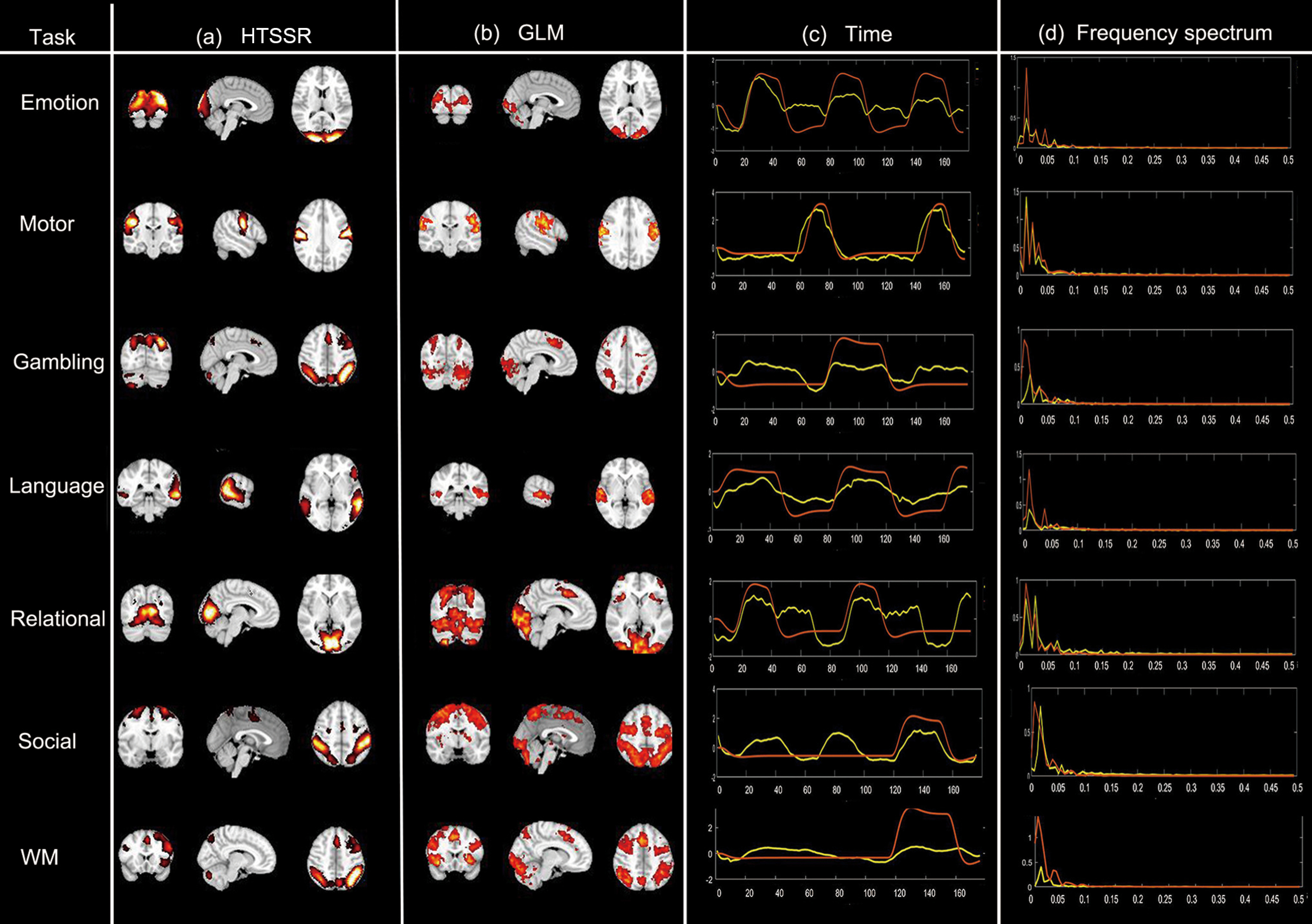
Identified task-evoked functional components of seven tasks (results of one experiment). ***a***, Identified task-evoked components by hybrid temporal and spatial sparse representations (HTSSR) framework. ***b***, Corresponding GLM-derived activation maps. ***c***, Learned time courses of the task-evoked components (yellow) and task design paradigms curves (red). ***d***, frequency spectrum of the components (yellow) and frequency spectrum of the task design (red). See also Extended Data Figures 3-1, 3-2, 3-3, 3-4, 3-5, 3-6, 3-7, 3-8, 3-9.

The most predominant functional components identified by our framework are the task-evoked functional components, including Emotion, Motor, Gambling, Language, Relational, Social, and Working memory. Specifically, the derived temporal patterns are relatively consistent with the task design paradigms for most of tasks (emotion, motor, language, and WM) although only 10% voxel signals were used during TSR (Fig. 3*c*) and their associated spatial distributions (Fig. 3*a*) are also relatively consistent with the results from the groupwise GLM-derived activations (Fig. 3*b*). In addition, the frequency spectrum of its time courses is highly concentrated on the task design frequency (Fig. 3*d*). However, despite for relatively consistent functional components derived from proposed method compared with GLM-based results, there still exist some disparities, especially in gambling, and relational tasks (Fig. 3*a*).

Considering the fact that 10% random voxels may probably introduce these disparities although it reduces the computational burden, we thus conducted the proposed model 10 times to assess the robustness of proposed model and the consistency of the activation patterns, where different training and testing sets were employed and 10% of voxels of whole brain for each subject were randomly selected for each experiment. The results show that while there were slight differences in spatial activation pattern and strength, the overall functional activations show high consistency across 10 experiments (Extended Data Fig. 3-1). Meanwhile, the revealed temporal patterns of 10 experimental results were generally consistent with task design paradigms across 10 experimental results (Extended Data Fig. 3-2). Furthermore, we computed the Pearson correlation coefficient between the derived time courses and task paradigms (PCCTC), and the overlap rates of the functional activation maps with GLM results for seven tasks and 10 experiments, separately (Table 1). The average Pearson correlation coefficient between the time courses of seven identified task-evoked components and the task paradigms is 0.72 ± 0.05 (mean ± SD). In terms of spatial similarity, the average overlap rate of seven task-evoked components across 10 experiments is 0.67 ± 0.10 (mean ± SD). The defined brain activation and temporal patterns of 10 experimental results can be found in Extended Data Figures 3-1, 3-2. In general, these results show that while adopting 10% randomly selected voxels for our sparse decomposition method can slightly affect the derived functional activation patterns, the overall pattern can be very consistent across different tests, further demonstrating the robustness of the proposed framework.

**Table 1:** The average Pearson correlation coefficients of the time courses (PCCTC) and the overlap rates of the functional networks of seven tasks for 10 experiments (mean ± SD)

Task	Emotion	Motor	Gambling	Language	Relational	Social	WM
Event	1	6	2	2	2	1	2
PCCTC	0.81 ± 0.05	0.91 ± 0.02	0.51 ± 0.09	0.87 ± 0.02	0.63 ± 0.04	0.66 ± 0.10	0.66 ± 0.06
Overlap	0.89 ± 0.05	0.70 ± 0.09	0.49 ± 0.13	0.62 ± 0.14	0.54 ± 0.13	0.79 ± 0.11	0.63 ± 0.08

10.1523/ENEURO.0478-21.2022.f3-1 Extended Data Figure 3-1 Brain activation of seven tasks for 10 experiments. Download Figure 3-1, TIF file.

10.1523/ENEURO.0478-21.2022.f3-2 Extended Data Figure 3-2 Representative temporal patterns of seven tasks for 10 experiments. Download Figure 3-2, TIF file.

Overall, based on the temporal, spatial, frequency-domain characteristic results, we concluded that our framework could effectively identify task-evoked functional components from large scale combined multitask fMRI data. Additional results of identified task-evoked functional components could be found in Extended Data Figures 3-3, 3-4, 3-5, 3-6, 3-7, 3-8.

10.1523/ENEURO.0478-21.2022.f3-3 Extended Data Figure 3-3 Task-evoked network for the emotion task. a, Identified task-evoked components by HTSSR framework. b, Corresponding GLM-derived activation maps. c, Learned time courses of the task-evoked components (yellow), task design paradigms curves (red). d, frequency spectrum of the components (yellow), frequency spectrum of the task design (red). Download Figure 3-3, TIF file.

**Figure 4.**
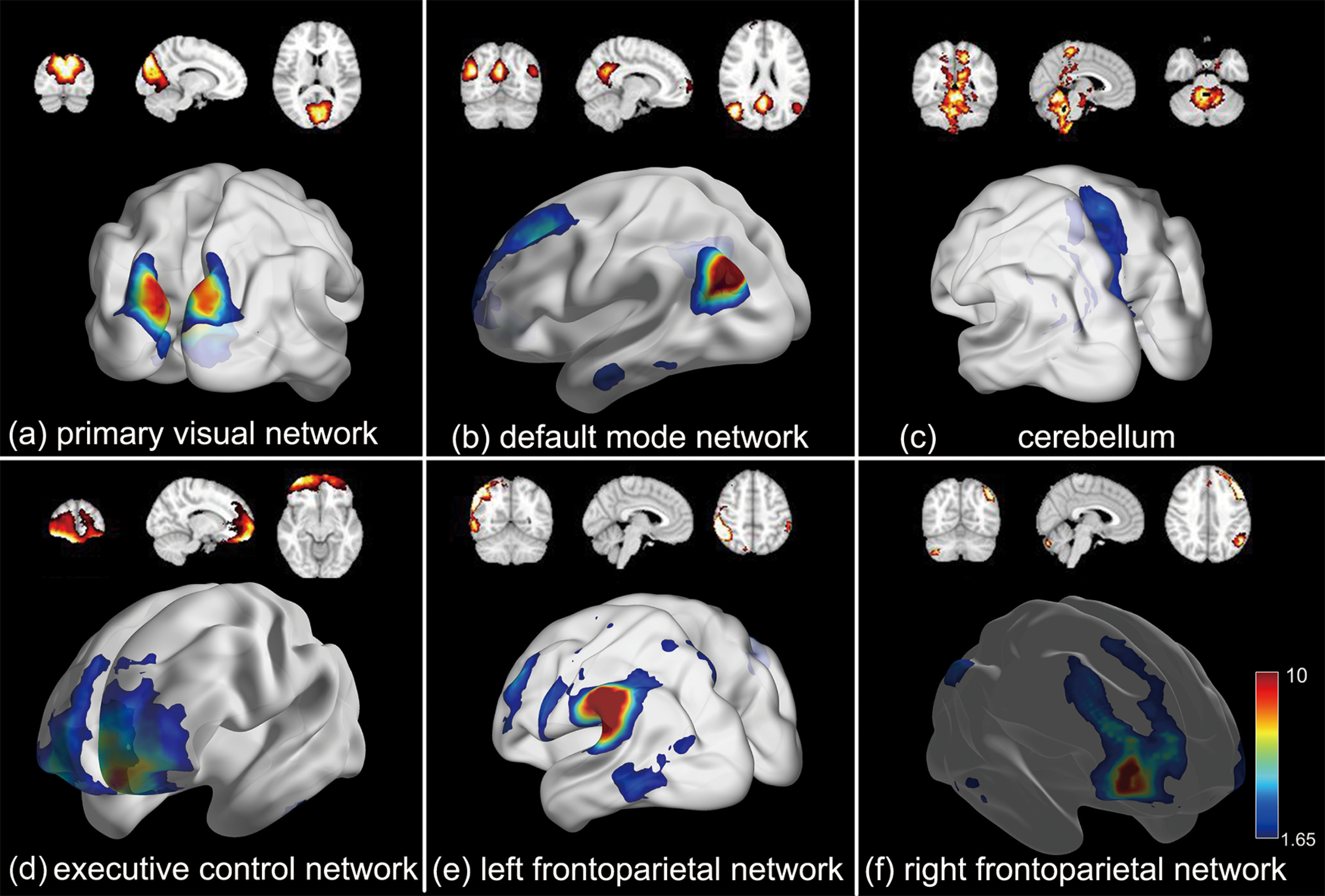
Six RSNs in the tfMRI dataset identified by our method, including (***a***) primary visual network, (***b***) default mode network (DMN), (***c***) cerebellum, (***d***) executive control network, (***e***) left frontoparietal network (lFPN), and (***f***) right frontoparietal network (rFPN).

10.1523/ENEURO.0478-21.2022.f3-4 Extended Data Figure 3-4 Task-evoked network for the motor task. a, Identified task-evoked components by HTSSR framework. b, Corresponding GLM-derived activation maps. c, Learned time courses of the task-evoked components (yellow), task design paradigms curves (red). d, frequency spectrum of the components (yellow), frequency spectrum of the task design (red). Download Figure 3-4, TIF file.

**Figure 5.**
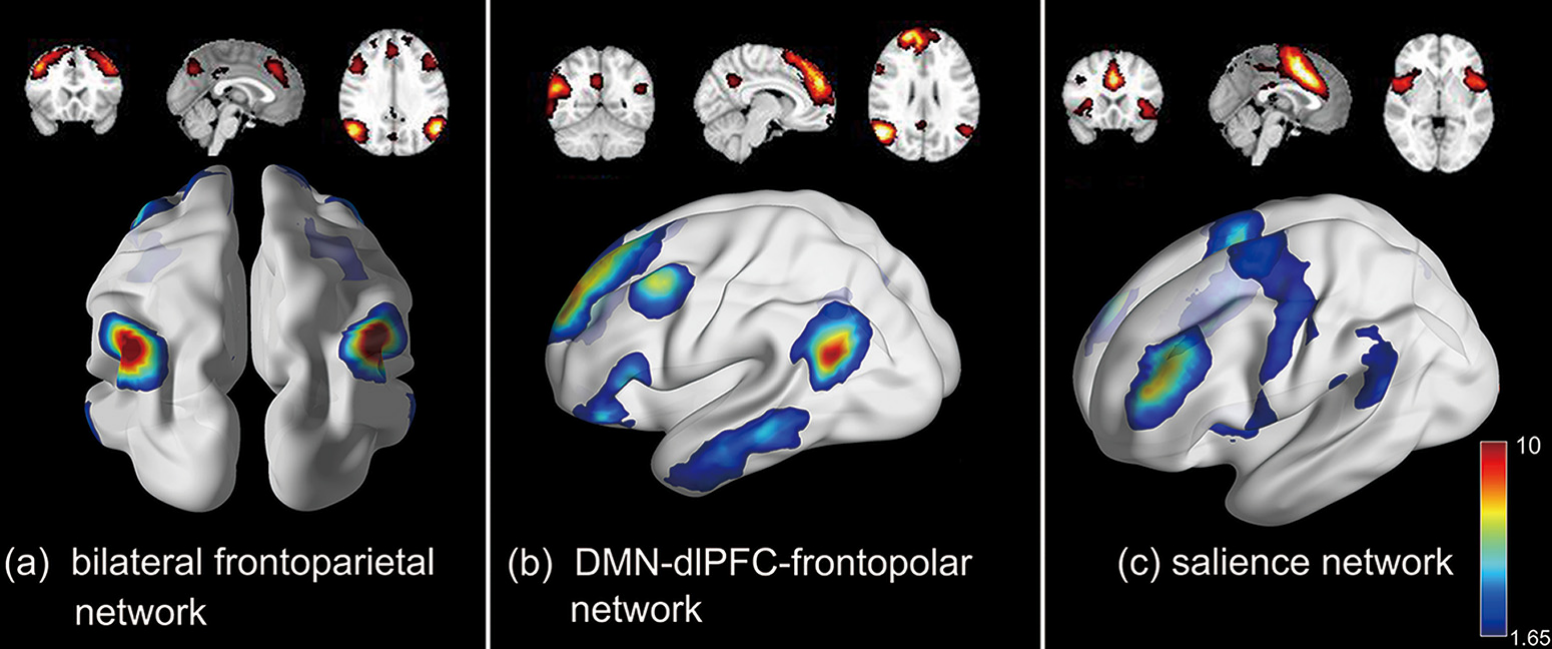
Three integrated functional networks identified by our framework, including (***a***) bilateral frontoparietal network (FPN), (***b***) network blend with a default mode network (DMN), dorsolateral prefrontal cortex (dlPFC), and frontopolar area, and (***c***) salience network (SN).

10.1523/ENEURO.0478-21.2022.f3-5 Extended Data Figure 3-5 Task-evoked network for the gambling task. a, Identified task-evoked components by HTSSR framework. b, Corresponding GLM-derived activation maps. c, Learned time courses of the task-evoked components (yellow), task design paradigms curves (red). d, frequency spectrum of the components (yellow), frequency spectrum of the task design (red). Download Figure 3-5, TIF file.

### Resting-state functional components

In addition to task-evoked component, our framework can reliably define resting-state networks (RSNs). Several brain networks that have been established previously were identified, including primary visual network (Fig. 4*a*), default mode network (DMN; Fig. 4*b*), cerebellum (Fig. 4*c*), executive control network (Fig. 4*d*), left frontoparietal network (lFPN; Fig. 4*e*), and right FPN (rFPN; Fig. 4*f*; Damoiseaux et al., 2006) . The 3D brain networks were visualized with the BrainNet Viewer (http://www.nitrc.org/projects/bnv/; Xia et al., 2013). Our results further demonstrate that when participants are processing specific cognitive tasks, the RSNs are also consistently “active” (Deco et al., 2013).

### Integrated functional component

Besides task evolved and resting-state functional components, some complex interconnected networks so-called integrated functional component can be found in our work as well. Figure 5*a* shows a bilateral FPN, which might indicate the interaction between lFPN and rFPN. The frontoparietal network (FPN) is critical for our ability to coordinate behavior in a rapid, accurate, and flexible goal-driven manner (Marek and Dosenbach, 2018). Figure 5*b* illustrates a network blended with a DMN, dorsolateral prefrontal cortex (dlPFC) and frontopolar area. Some studies demonstrated that the dlPFC has robust fMRI functional connectivity and reciprocal anatomic connections with the posterior DMN core regions: posterior parietal cortex (PPC) and posterior cingulate cortex (PCC) in marmoset (Liu et al., 2019). This complex network shown in Figure 5*b* may be associated with mental processes that require rigorous computation, control, and decision-making. Figure 5*c* shows another complex network named salience network (SN), which plays a crucial role in identifying the most biologically and cognitively relevant events for adaptive guiding attention and behavior, and constitutes a key interface for cognitive, homeostatic, motivational, and affective systems (Seeley et al., 2007). These integrated functional components were activated during the task and could reflect the interactions between different brain regions/network, indicating that our framework cannot only define traditional task-evoked and resting-state functional components, but also reveal the interconnections between brain regions/networks.

### Artifact-related component

**Figure 6.**
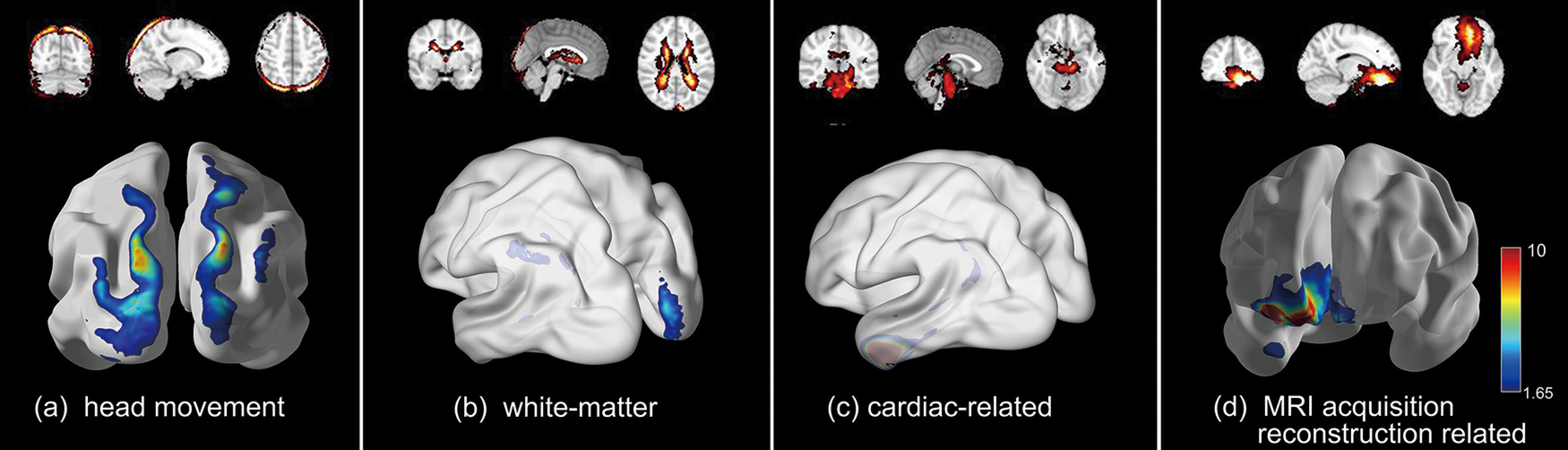
Artifact-related components detected by our framework, including (**a**) head movement, (***b***) white-matter, (***c***) cardiac-related, and (***d***) magnetic resonance imaging (MRI) acquisition/reconstruction related.

Our framework cannot only define meaningful networks, but also detect artifact-related components related to head movement (Fig. 6*a*), white-matter (Fig. 6*b*), cardiac-related (Fig. 6*c*), and MRI acquisition/reconstruction related (Fig. 6*d*). Head movement and cardiac artifact-related components are mainly caused by physiology and subject motion during MRI acquisition (Fig. 6*a*,*c*; Griffanti et al., 2014). In addition, white-matter and MRI acquisition/reconstruction artifact-related components could be caused by the MRI hardware or software (Fig. 6*b*,*d*; Griffanti et al., 2014; Salimi-Khorshidi et al., 2014). Detecting and separating these artifact-related component make sense for finding meaningful networks.

### The functional role of resting state and artifact components in multitask classification

We here further investigated the role of resting state and artifact components in multitask classification. Specifically, we excluded these two kinds of components from all the defined components of training set respectively, and put the rest components into the SVM model to train the classifier, resulting in four types of component groups used for classification (“all the components,” “excluding artifact components,” “excluding resting state components,” and “excluding both artifact and resting state components”). Afterwards, we selected and imported these four types of components of the testing set into the trained model to obtain classification rate, respectively. Overall, we conducted this experiment five times on different training and testing sets. The results show that the classification rates are relatively high and there is little difference in the classification rate among different cases (Extended Data Fig. 2-1). In two testing sets, excluding resting state components results in lower accuracy, but the effect is not significant. The main reason for this slight effect is that these two types of components account for a small proportion of all defined components (only 10 components in total). Thus, task-related components play the most important role in classification analysis.

### The underlying network mechanism of key components with high classification accuracy

**Figure 7.**
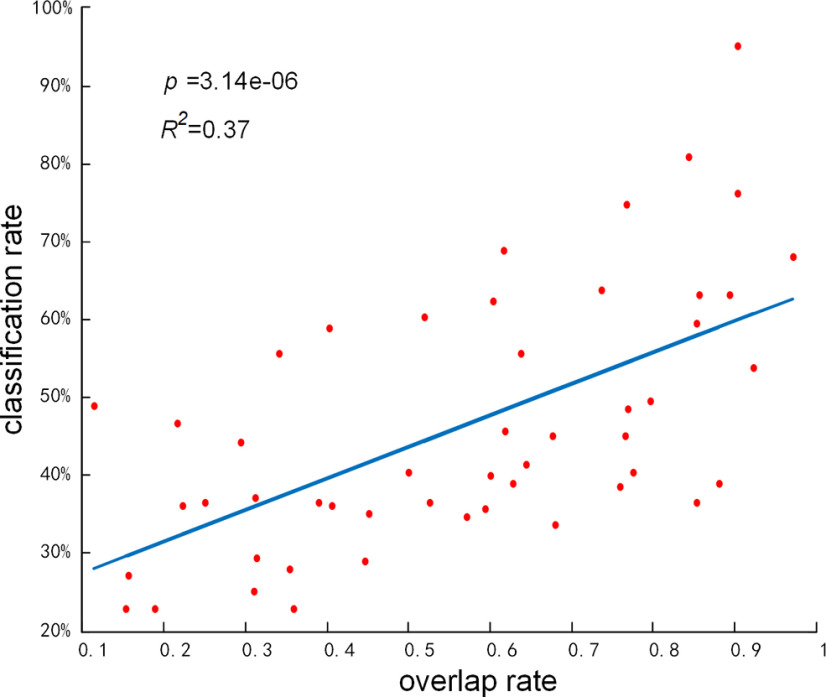
Correlation between classification performance and spatial overlap rate of each functional network. Red points present k2 components (total 50 in our work) derived from SSR stage, and the blue line presents the regression line of these components.

To further explore the neural implications of key components with greater classification capacity, we investigated whether there is significant correlation between the classification accuracy and the overlap rate of each component, where the overlap rate of component is defined as the spatial matching rate with GLM-derived activation patterns or RSNs templates. As shown in Figure 7, *y*-axis represents the accuracy of using each independent component in $\alpha_{\text{test}}^{2}$ for multitask classification reflecting their classification capacity, and *x*-axis refers to the spatial overlap rate of corresponding atom in dictionary ***D***^2^. Each red point represents the component in loading coefficient ***α***^2^ derived from SSR stage. Note that the classification accuracy is significantly correlated with the overlap rate of each component (*R*^2^ = 0.37, *p *=* *3.14e-06; Fig. 7). These results thus suggest that a strengthened overlap rate predicts greater classification capacity of a functional component, indicating the underlying network mechanism of classification ability for derived functional components and gaining the interpretability of the proposed framework.

## Discussion

In this study, we proposed a framework using HTSSR to examine fundamental differences in multitask fMRI signal composition patterns that can effectively classify seven task fMRI signals with an average accuracy of 96.67%. In addition, our framework also identified interpretable and distinctive spatial-temporal components critical for differentiating diverse tfMRI signals, and disclosed the neural implications of these key components. Furthermore, our framework can effectively detect various networks including task-related components, RSNs, integrated complex functional components and artifact-related components, further suggesting the effectiveness of proposed method.

Considering the challenges and pitfalls of existing studies, our framework provided a suitable way to classify multitask fMRI signals and characterize the key components for classification and their underlying network mechanism. First, regarding to enormous number of voxels in group-wise fMRI signals, our framework only randomly selected 10% of whole-brain signals for each subject in TSR stage, which greatly reduced the matrix dimensions of millions of data points for group-wise signals, and dramatically lessened the computational burden while preserving the inherent features of tfMRI signals. Consequently, with only 10% signals adopted as training sample, our framework can still effectively and efficiently differentiate the group-wise multitask fMRI signals. Second, in terms of tackling the issues of interindividual variability and the noise of fMRI signals, our framework successfully derived meaningful functional activation patterns by extracting group-wise common dictionary from fMRI signals of all the subjects with great interindividual variability. Meanwhile, the two-stage sparse representations framework can effectively remove most noises. Third, regarding to the limited sample size of task-based fMRI datasets and clinical populations, our framework is effective on decoding multitasks fMRI signals for small cohort datasets. Finally, our study further defined the critical components in multitask classification and their neural implications. Specifically, our results uncovered the significant correlation between classification accuracy and the overlap rate with well-defined network templates of each component, indicating the underlying neural mechanism of key components with great classification capacity.

Despite the promising classification performance of the HTSSR framework, there also exist some limitations. First, while most of the functional components corresponding to the events of task designs have been detected by our framework, there are still a few activation maps that have not been found compared with GLM-based results, such as 0BK_FACE design of WM. Second, some functional components and associated temporal patterns derived by our framework are not perfectly consistent with those defined by GLM-based method. For instance, compared with the original design, time course of relational task has an additional task block (Extended Data Fig. 3-9*c*), and its associated activation map misses some regions such as the dorsal anterior cingulate cortex and inferior prefrontal gyrus (Extended Data Fig. 3-9*a*). One reason causing these disparities is that to find the key features for task classification, the number of dictionary atoms in the second stage of our method was set to 50, resulting in only 50 functional components defined. In contrast, the atom number was usually set to 400 in previous task-based activation identification studies using sparse decomposition method (Lv et al., 2015a; Zhao et al., 2018). In addition, for the classification purpose, our method concatenated and aggregated the tfMRI signals of seven tasks together for the model training, which leads to truncations of some tasks, instead of applying the sparse decomposition method to single and complete task fMRI data alone (Lv et al., 2015a). The truncation would lead to incomplete task designs (Extended Data Fig. 1-1), thus impacting the characterization of task-related functional activations. For example, four events were excluded in WM task because of the data truncation, the activations of which would not be detected inevitably. On the other hand, another reason might be that we randomly selected 10% voxels of each subject for model training and testing. Thus, while previous functional activations detection studies using sparse representation framework manifest great consistency with GLM-based results, the limited number of dictionary atoms, truncations of tfMRI signals, and 10% randomly selected voxels for group-wise training of our method might result in disparities between functional components defined and GLM-based results. Nevertheless, despite the existing disparities, the main purpose of our study was to develop an efficient and effective framework for multitask classification and uncover the interpretable and discriminative fMRI composition patterns.

10.1523/ENEURO.0478-21.2022.f3-9 Extended Data Figure 3-9 The brain activation and temporal patterns of relational task (the enlarged view of the relational task of Fig. 3). Download Figure 3-9, TIF file.

Third, regarding to the parameters setting issue of HTSSR framework, another limitation of our study is manual setting of sparse parameters λ for two stage sparse representation. As there is no golden criterion for the selection of λ in sparse representation algorithm, we here systematically varied the λ settings and assessed their impact on the classification performance using separate training and testing sets. According to the parameter setting in previous FBNs identification studies using sparse representation methods (Lv et al., 2015a; Zhang et al., 2017; Ge et al., 2018), we assessed the impact of parameter settings by systematically varying λ1 (0.05, 0.1, 0.5) and λ2 (0.05, 0.1, 0.5). To avoid information leakage, we randomly selected 30 subjects from the whole dataset as training set and trained the model using training set alone with different combinations of λ1 and λ2, and then conducted the classification analysis on the testing set composed of remaining subjects, using all parameters derived from the trained model. The classification results with different parameter ssettings are shown in Table 2. The classification rates for most combinations of λ1 and λ2 are consistently high, except for the “λ1 = 0.5.” By manually inspecting all the functional activations derived with λ1 = 0.5 (Extended Data Fig. 2-2), we find that the functional activation maps become very sparse with no meaningful activation patterns under these parameter settings. Thus, when the sparsity penalty was set too large, the key functional components in differentiating the multitask signals cannot be characterized, consequently leading to poor classification performance. However, the overall classification performance was quite stable and satisfactory with reasonable λ settings. Therefore, in our study, the λ1 and λ2 were set to 0.05 and 0.1, respectively. In the future, we would like to further develop an automatic optimization strategy for parameter setting of the proposed method.

**Table 2:** The classification rates on testing set using different parameter settings of HTSSR model

λ1\λ2	0.05	0.1	0.5
0.05	99.05%	98.57%	99.52%
0.1	99.52%	99.52%	99.05%
0.5	14.29%	14.29%	14.29%

Overall, our proposed framework provided an effective and interpretable tool for classifying multitask fMRI data. In the future, this framework can be easily applied to a wide range of neuroimaging research with a small dataset, such as mental state classification or brain disorders diagnosis.

10.1523/ENEURO.0478-21.2022.f3-8 Extended Data Figure 3-8 Task-evoked network for the WM task. a, Identified task-evoked components by HTSSR framework. b, Corresponding GLM-derived activation maps. c, Learned time courses of the task-evoked components (yellow), task design paradigms curves (red). d, frequency spectrum of the components (yellow), frequency spectrum of the task design (red). Download Figure 3-8, TIF file.

10.1523/ENEURO.0478-21.2022.f3-7 Extended Data Figure 3-7 Task-evoked network for the relational task. a, Identified task-evoked components by HTSSR framework. b, Corresponding GLM-derived activation maps. c, Learned time courses of the task-evoked components (yellow), task design paradigms curves (red). d, frequency spectrum of the components (yellow), frequency spectrum of the task design (red). Download Figure 3-7, TIF file.

10.1523/ENEURO.0478-21.2022.f3-6 Extended Data Figure 3-6 Task-evoked network for the language task. a, Identified task-evoked components by HTSSR framework. b, Corresponding GLM-derived activation maps. c, Learned time courses of the task-evoked components (yellow), task design paradigms curves (red). d, frequency spectrum of the components (yellow), frequency spectrum of the task design (red). Download Figure 3-6, TIF file.

## Synthesis

Reviewing Editor: Christoph Michel, Universite de Geneve Decisions are customarily a result of the Reviewing Editor and the peer reviewers coming together and discussing their recommendations until a consensus is reached. When revisions are invited, a fact-based synthesis statement explaining their decision and outlining what is needed to prepare a revision will be listed below. The following reviewer(s) agreed to reveal their identity: Bhaskar Sen, Matthew Sherwood.

Both reviewers are satisfied with the additional analysis and description of the results and consider the paper acceptable. However, one reviewer asked for thorough proofreading. Indeed, there are a couple of grammatical mistakes, particularly in the newly added paragraphs. Please pay attention to these grammatical errors when correcting the proofs.
