# Supplementary figures and images for: Multitask fMRI Data Classification via Group-Wise Hybrid Temporal and Spatial Sparse Representations
Source: eNeuro. 2022 Jun 3;9(3):ENEURO.0478-21.2022. doi: 10.1523/ENEURO.0478-21.2022 (PMC9186416; doi:10.1523/ENEURO.0478-21.2022)

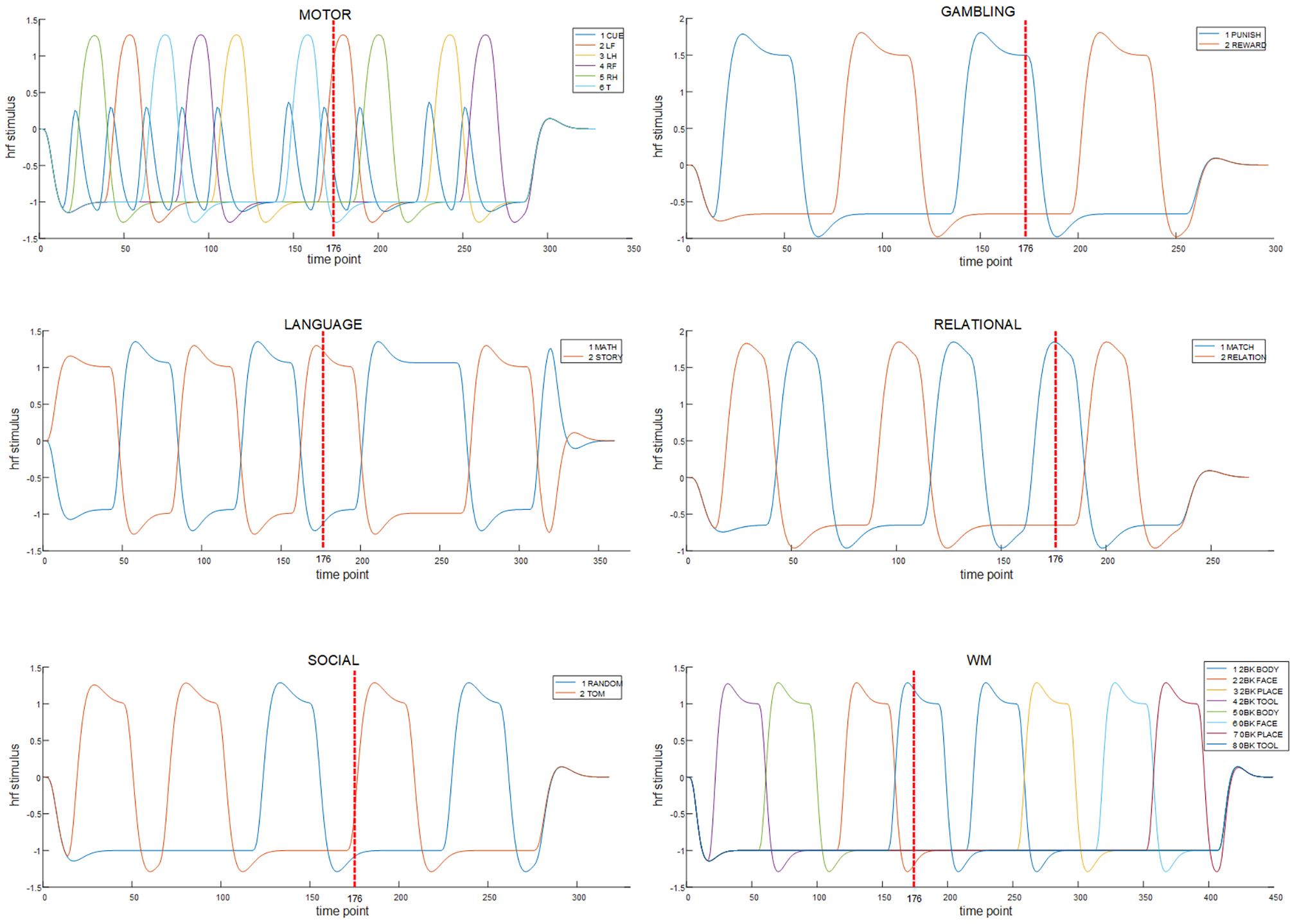

Supplement: Extended Data Figure 1-1 — Truncation of six task designs. Download Figure 1-1, TIF file. [file enu-eN-MNT-0478-21-s03.tif]

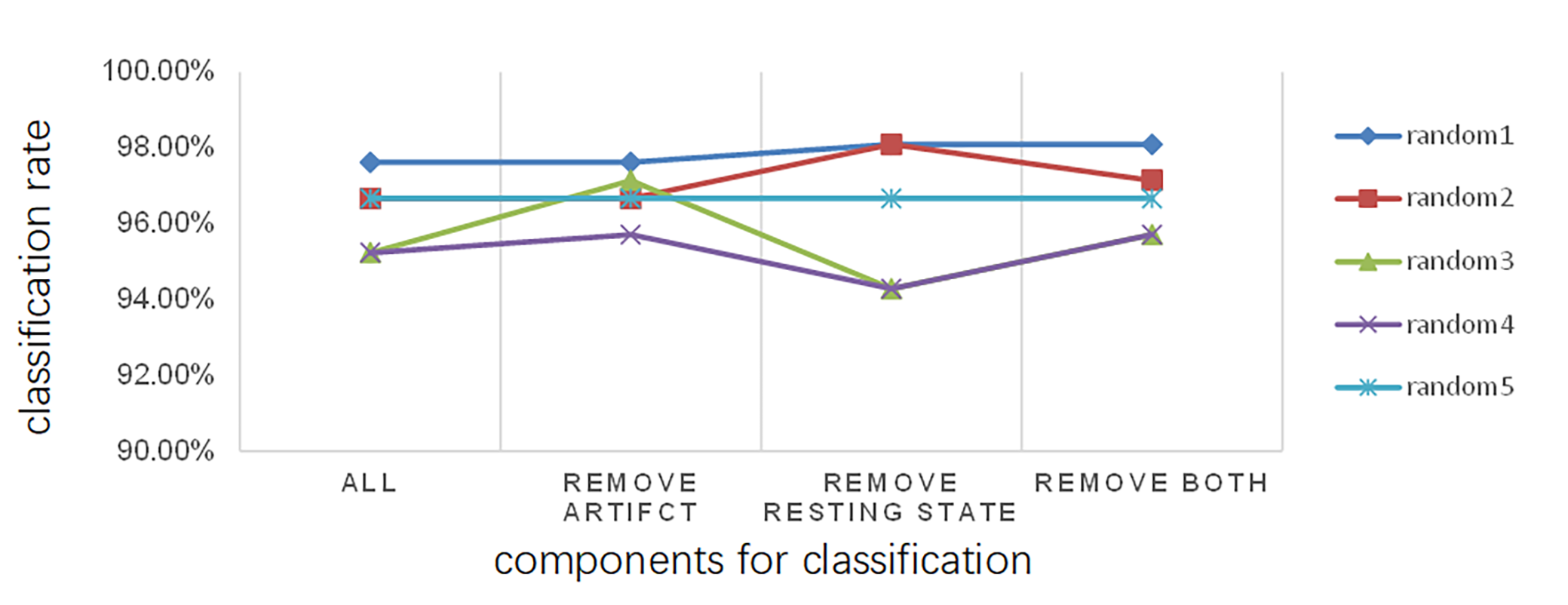

Supplement: Extended Data Figure 2-1 — Classification rate of eliminating resting state and artifact components. Download Figure 2-1, TIF file. [file enu-eN-MNT-0478-21-s04.tif]

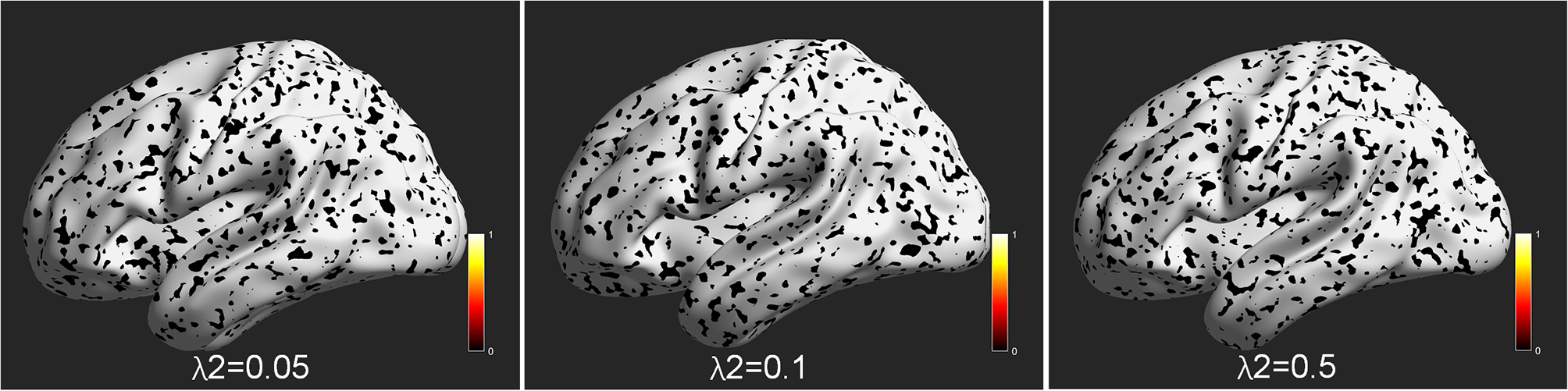

Supplement: Extended Data Figure 2-2 — Examples of functional activations derived by λ1 = 0.5. Download Figure 2-2, TIF file. [file enu-eN-MNT-0478-21-s05.tif]

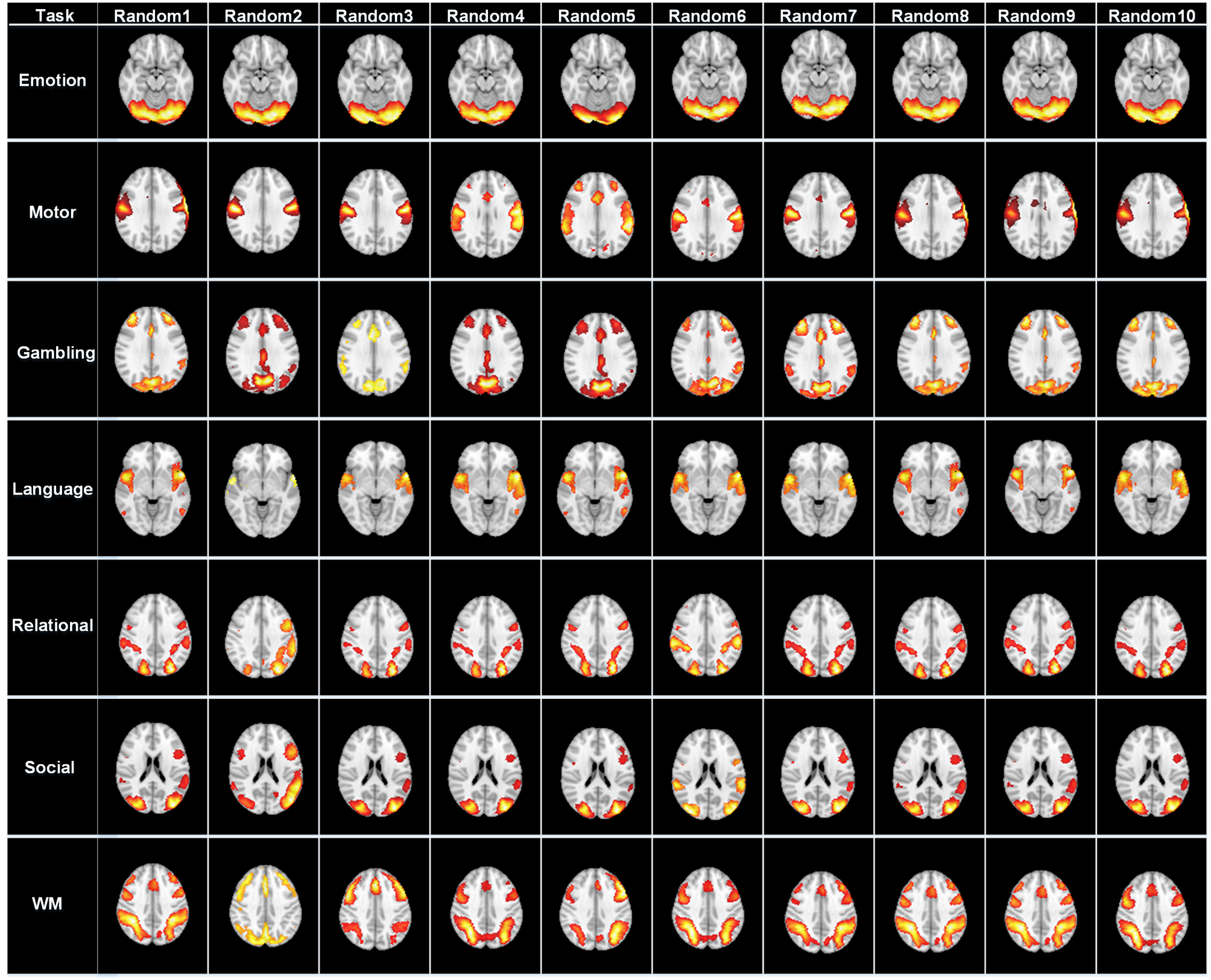

Supplement: Extended Data Figure 3-1 — Brain activation of seven tasks for 10 experiments. Download Figure 3-1, TIF file. [file enu-eN-MNT-0478-21-s06.tif]

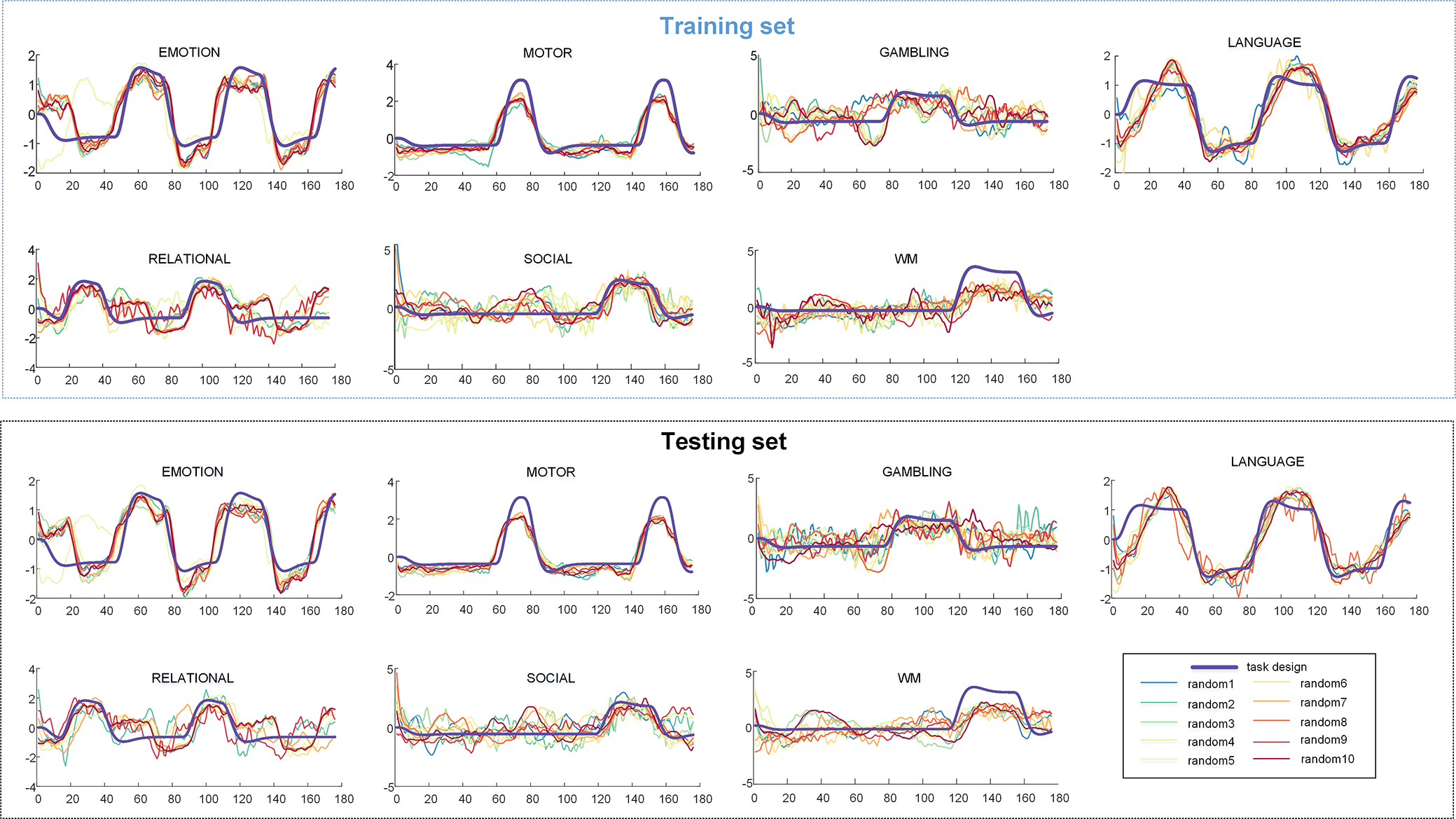

Supplement: Extended Data Figure 3-2 — Representative temporal patterns of seven tasks for 10 experiments. Download Figure 3-2, TIF file. [file enu-eN-MNT-0478-21-s07.tif]

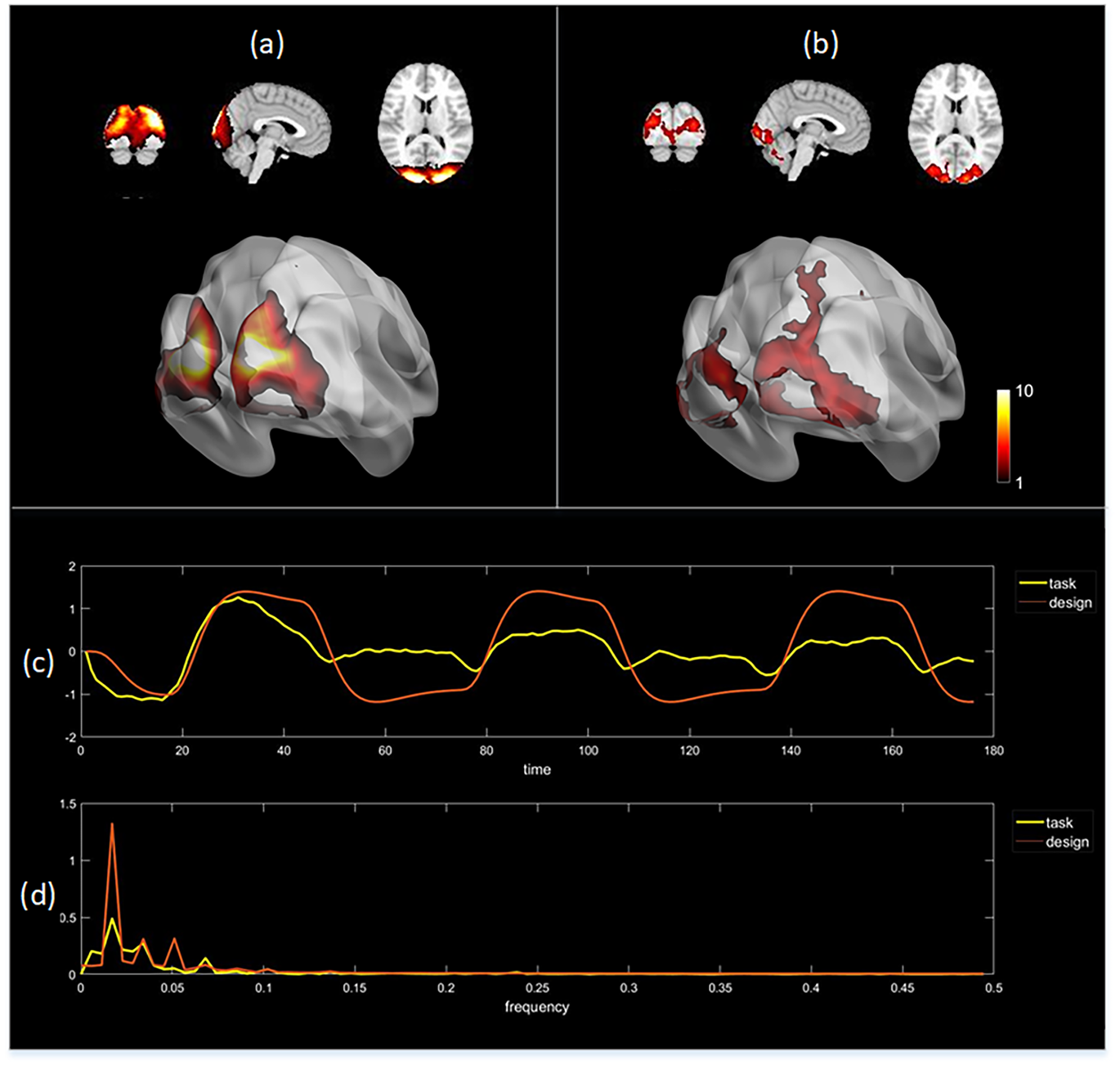

Supplement: Extended Data Figure 3-3 — Task-evoked network for the emotion task. a, Identified task-evoked components by HTSSR framework. b, Corresponding GLM-derived activation maps. c, Learned time courses of the task-evoked components (yellow), task design paradigms curves (red). d, frequency spectrum of the components (yellow), frequency spectrum of the task design (red). Download Figure 3-3, TIF file. [file enu-eN-MNT-0478-21-s08.tif]

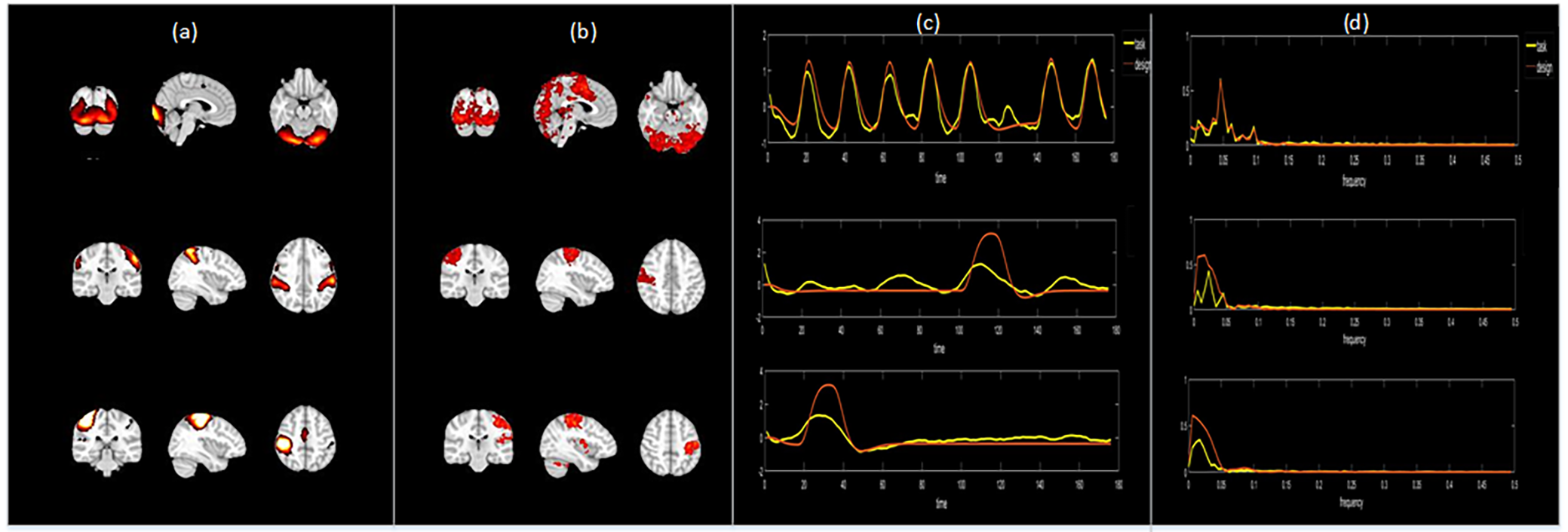

Supplement: Extended Data Figure 3-4 — Task-evoked network for the motor task. a, Identified task-evoked components by HTSSR framework. b, Corresponding GLM-derived activation maps. c, Learned time courses of the task-evoked components (yellow), task design paradigms curves (red). d, frequency spectrum of the components (yellow), frequency spectrum of the task design (red). Download Figure 3-4, TIF file. [file enu-eN-MNT-0478-21-s09.tif]

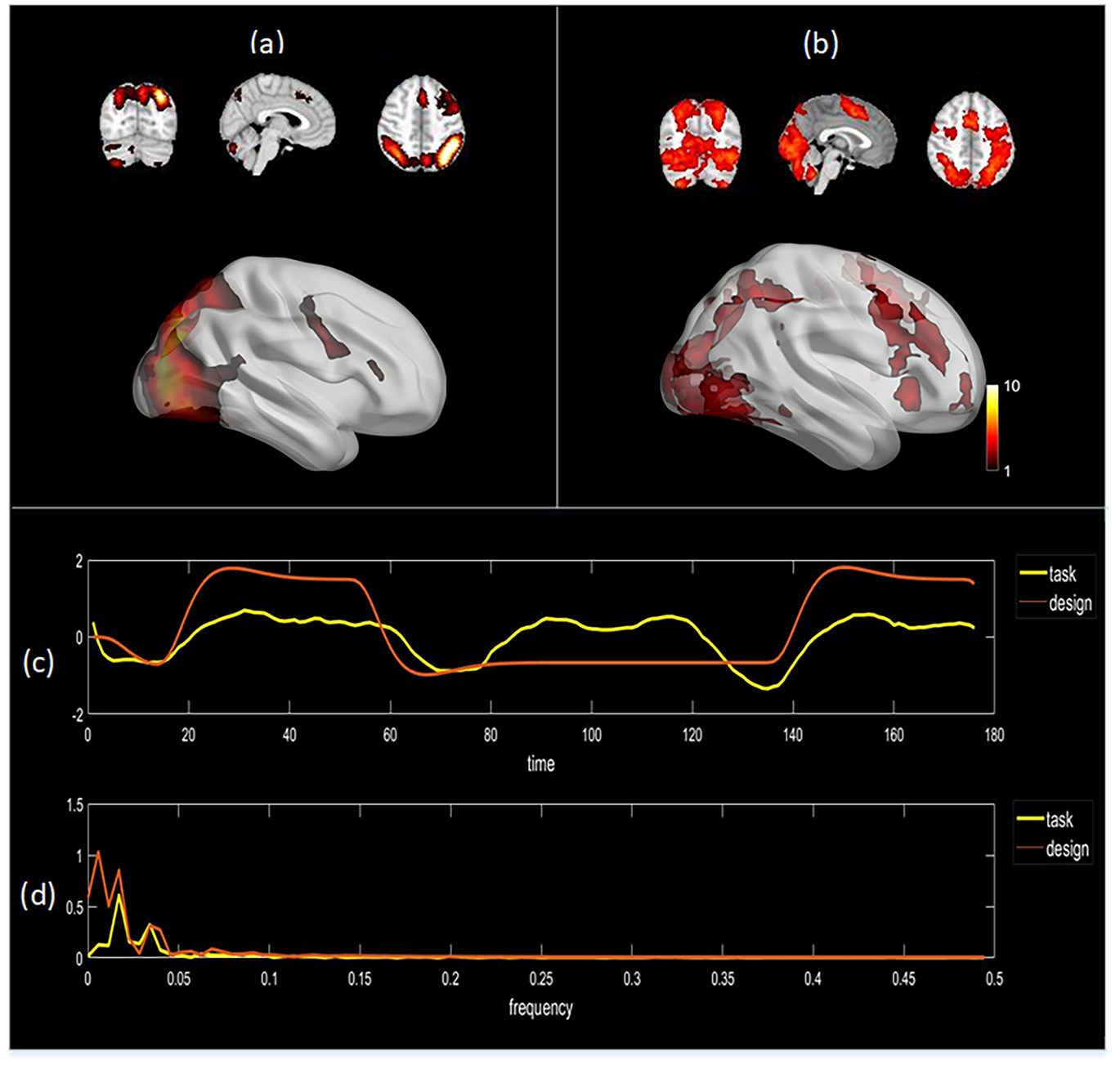

Supplement: Extended Data Figure 3-5 — Task-evoked network for the gambling task. a, Identified task-evoked components by HTSSR framework. b, Corresponding GLM-derived activation maps. c, Learned time courses of the task-evoked components (yellow), task design paradigms curves (red). d, frequency spectrum of the components (yellow), frequency spectrum of the task design (red). Download Figure 3-5, TIF file. [file enu-eN-MNT-0478-21-s10.tif]

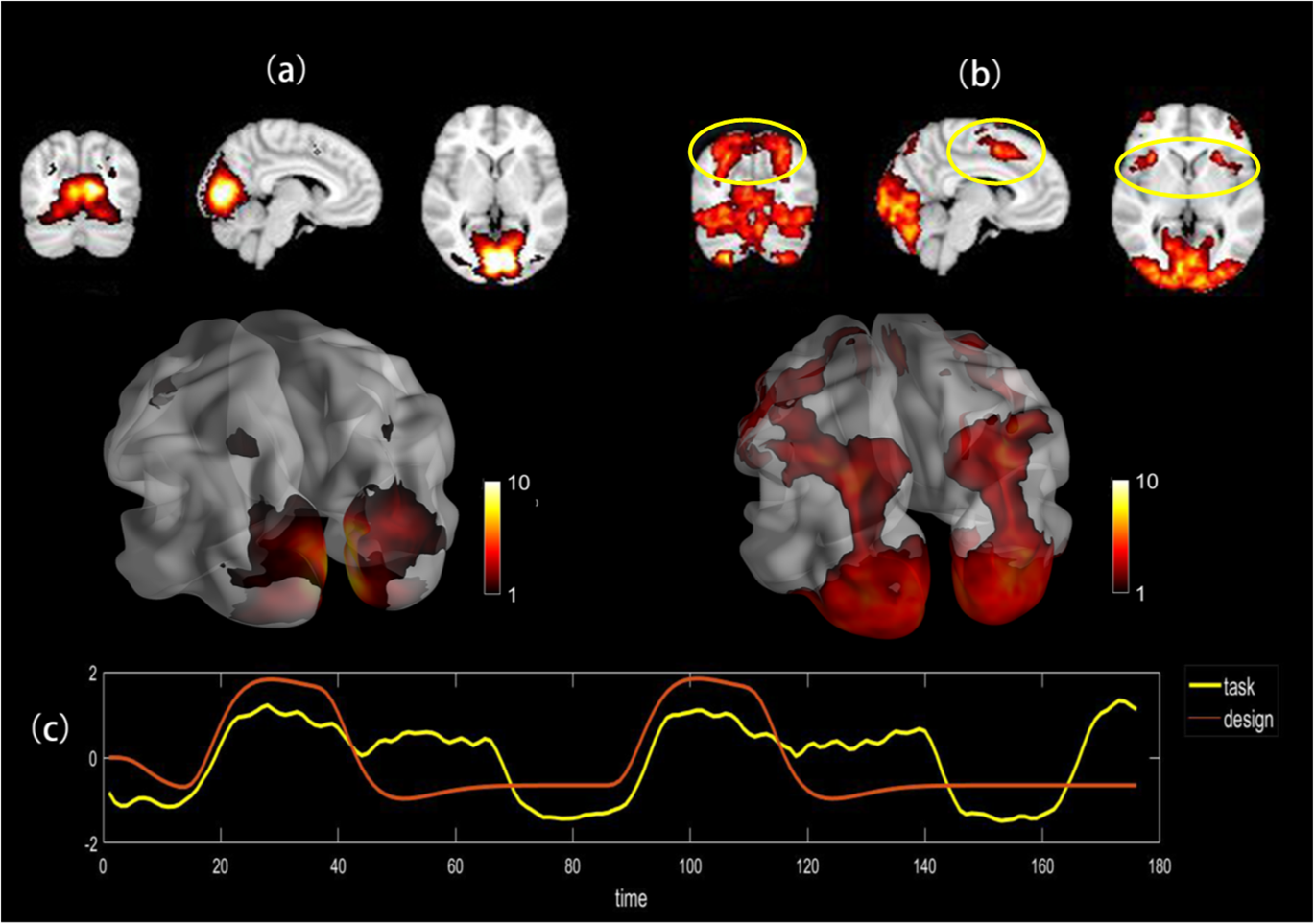

Supplement: Extended Data Figure 3-9 — The brain activation and temporal patterns of relational task (the enlarged view of the relational task of Fig. 3). Download Figure 3-9, TIF file. [file enu-eN-MNT-0478-21-s14.tif]

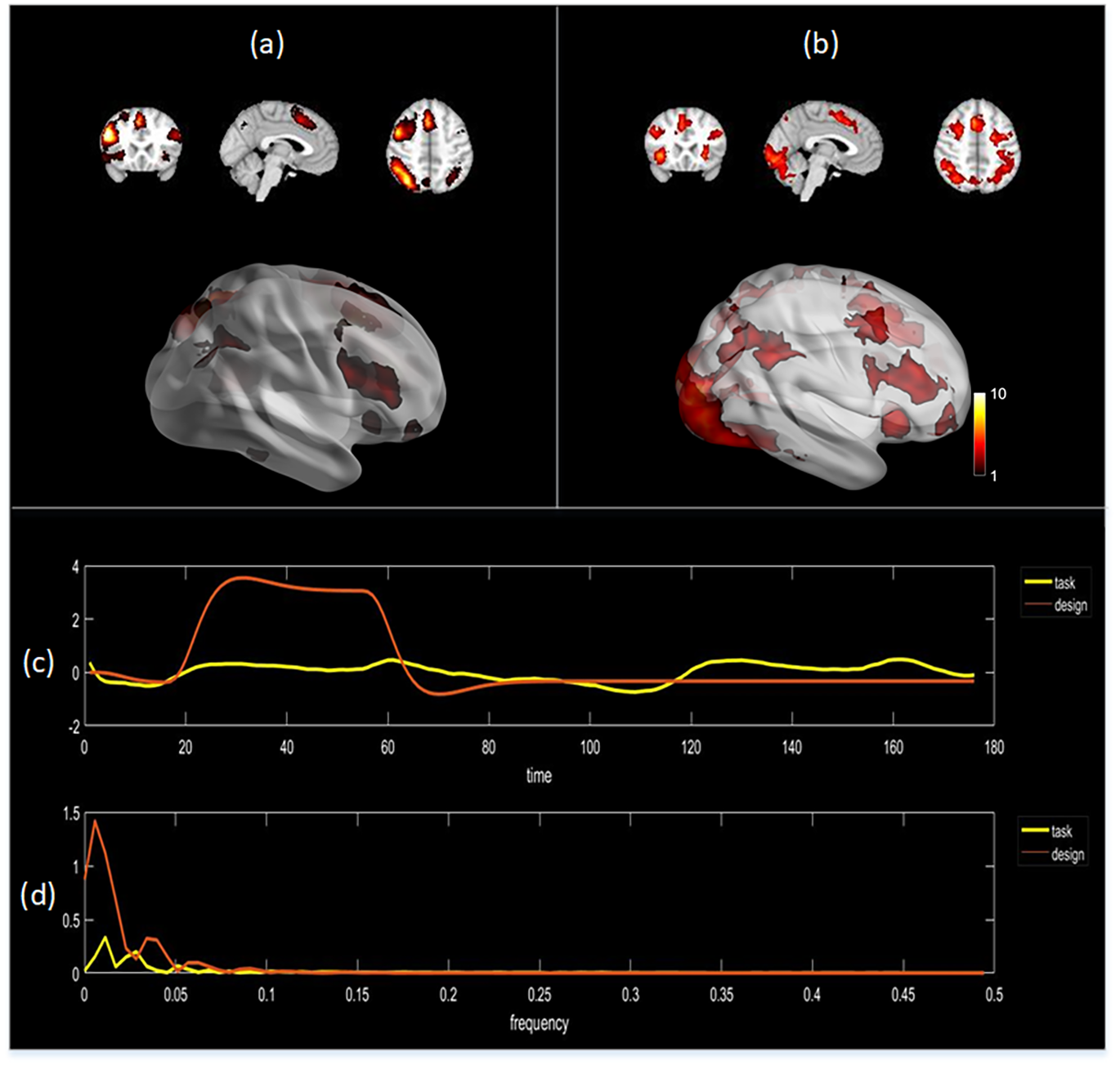

Supplement: Extended Data Figure 3-8 — Task-evoked network for the WM task. a, Identified task-evoked components by HTSSR framework. b, Corresponding GLM-derived activation maps. c, Learned time courses of the task-evoked components (yellow), task design paradigms curves (red). d, frequency spectrum of the components (yellow), frequency spectrum of the task design (red). Download Figure 3-8, TIF file. [file enu-eN-MNT-0478-21-s13.tif]

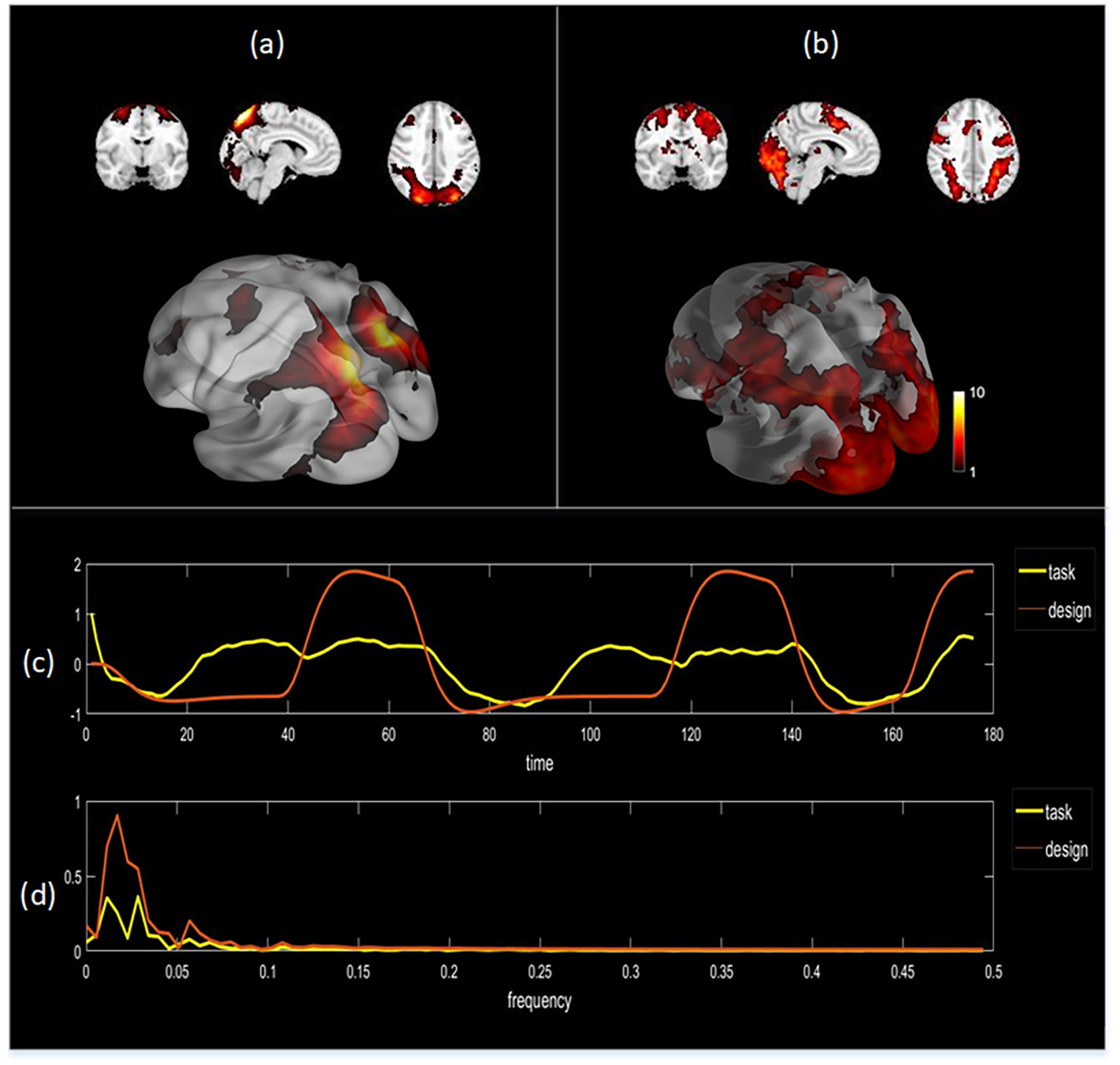

Supplement: Extended Data Figure 3-7 — Task-evoked network for the relational task. a, Identified task-evoked components by HTSSR framework. b, Corresponding GLM-derived activation maps. c, Learned time courses of the task-evoked components (yellow), task design paradigms curves (red). d, frequency spectrum of the components (yellow), frequency spectrum of the task design (red). Download Figure 3-7, TIF file. [file enu-eN-MNT-0478-21-s12.tif]

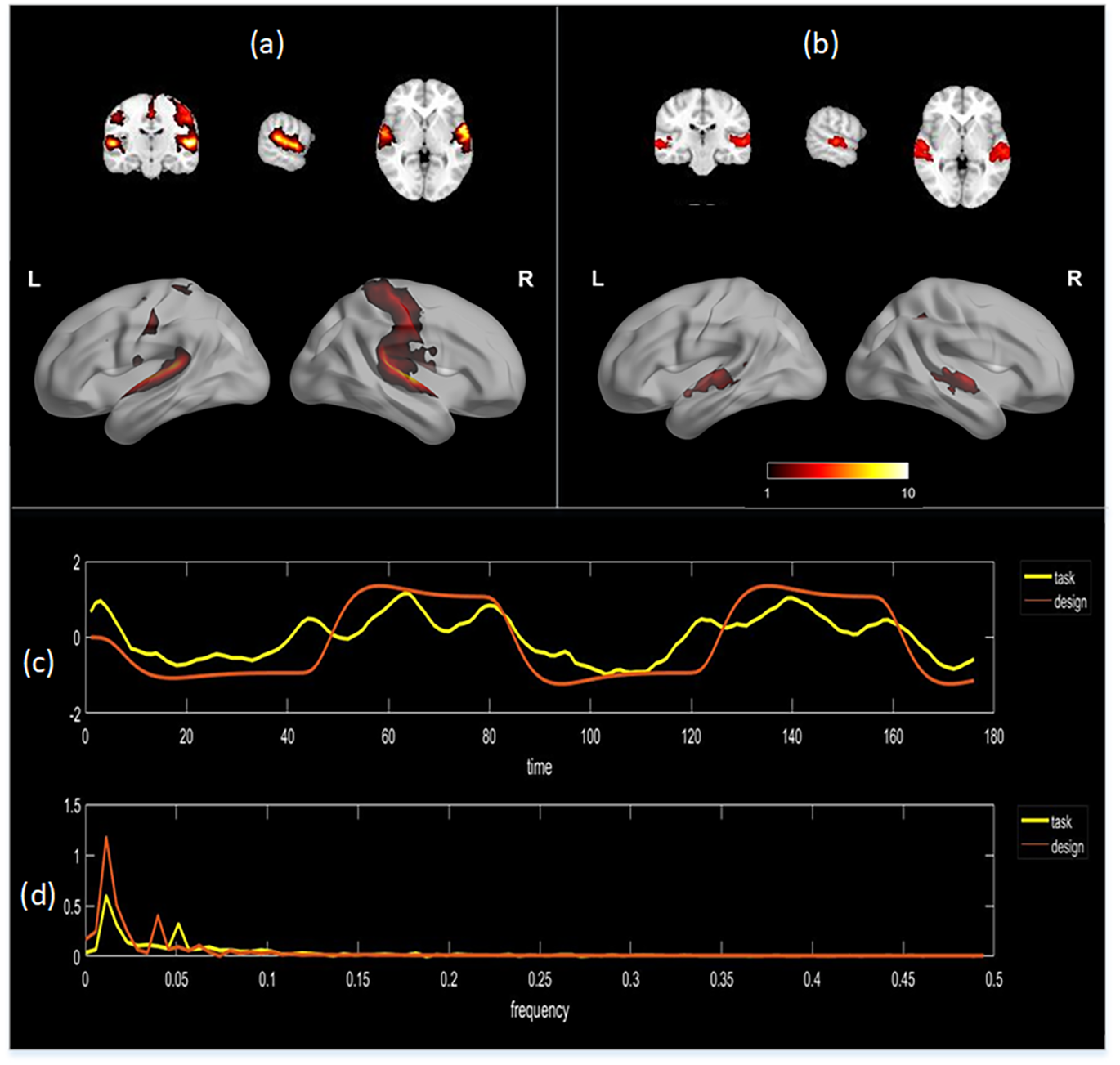

Supplement: Extended Data Figure 3-6 — Task-evoked network for the language task. a, Identified task-evoked components by HTSSR framework. b, Corresponding GLM-derived activation maps. c, Learned time courses of the task-evoked components (yellow), task design paradigms curves (red). d, frequency spectrum of the components (yellow), frequency spectrum of the task design (red). Download Figure 3-6, TIF file. [file enu-eN-MNT-0478-21-s11.tif]
