# Supplementary material for: Multitask fMRI Data Classification via Group-Wise Hybrid Temporal and Spatial Sparse Representations
Source: eNeuro. 2022 Jun 3;9(3):ENEURO.0478-21.2022. doi: 10.1523/ENEURO.0478-21.2022 (PMC9186416; doi:10.1523/ENEURO.0478-21.2022)
Supplement: Extended Data 1 — The code. Download Extended Data 1, ZIP file. [file enu-eN-MNT-0478-21-s02.zip › code/tools/spams-matlab.zip!/spams-matlab/doc/html/doc_spams.html]

SPAMS: a SPArse Modeling Software, v2.5


# SPAMS: a SPArse Modeling Software, v2.5

### Julien Mairal julien.mairal@m4x.org   Python and R interfaces by Jean-Paul Chieze   Archetypal Analysis by Yuansi Chen   and the output of numerous collaborations with Rodolphe Jenatton, Francis Bach, Jean Ponce, Guillaume Obozinski, Bin Yu, Guillermo Sapiro, and Zaid Harchaoui.

## Contents

- 1  Introduction
- 2  Installation
- 3  Dictionary Learning and Matrix Factorization Toolbox
  - 3.1  Function mexTrainDL
  - 3.2  Function mexTrainDL\_Memory
  - 3.3  Function mexStructTrainDL
  - 3.4  Function nmf
  - 3.5  Function nnsc
  - 3.6  Function mexArchetypalAnalysis
- 4  Sparse Decomposition Toolbox
  - 4.1  Function mexOMP
  - 4.2  Function mexOMPMask
  - 4.3  Function mexRidgeRegression
  - 4.4  Function mexLasso
  - 4.5  Function mexLassoWeighted
  - 4.6  Function mexLassoMask
  - 4.7  Function mexCD
  - 4.8  Function mexSOMP
  - 4.9  Function mexL1L2BCD
  - 4.10  Function mexSparseProject
  - 4.11  Function mexDecompSimplex
- 5  Proximal Toolbox
  - 5.1  Regularization Functions
  - 5.2  Function mexProximalFlat
  - 5.3  Function mexProximalTree
  - 5.4  Function mexProximalGraph
  - 5.5  Function mexProximalPathCoding
  - 5.6  Function mexEvalPathCoding
  - 5.7  Problems Addressed
    - 5.7.1  Regression Problems with the Square Loss
    - 5.7.2  Classification Problems with the Logistic Loss
    - 5.7.3  Multi-class Classification Problems with the Softmax Loss
    - 5.7.4  Multi-task Regression Problems with the Square Loss
    - 5.7.5  Multi-task Classification Problems with the Logistic Loss
    - 5.7.6  Multi-task and Multi-class Classification Problems with the Softmax Loss
  - 5.8  Function mexFistaFlat
  - 5.9  Function mexFistaTree
  - 5.10  Function mexFistaGraph
  - 5.11  Function mexFistaPathCoding
  - 5.12  Function solverPoisson
  - 5.13  Function mexIncrementalProx
  - 5.14  Function mexStochasticProx
- 6  A few Functions for manipulating images
  - 6.1  Function mexExtractPatches
  - 6.2  Function mexCombinePatches
  - 6.3  Function mexConvFistaFlat
- 7  Miscellaneous Functions
  - 7.1  Function mexConjGrad
  - 7.2  Function mexBayer
  - 7.3  Function mexCalcAAt
  - 7.4  Function mexCalcXAt
  - 7.5  Function mexCalcXY
  - 7.6  Function mexCalcXYt
  - 7.7  Function mexCalcXtY
  - 7.8  Function mexInvSym
  - 7.9  Function mexNormalize
  - 7.10  Function mexSort
  - 7.11  Function mexDisplayPatches
  - 7.12  Function mexCountPathsDAG
  - 7.13  Function mexRemoveCyclesGraph
  - 7.14  Function mexCountConnexComponents
  - 7.15  Function mexGraphOfGroupStruct
  - 7.16  Function mexGroupStructOfString
  - 7.17  Function mexReadGroupStruct
  - 7.18  Function mexTreeOfGroupStruct
  - 7.19  Function mexSimpleGroupTree
- A  Duality Gaps with Fenchel Duality

  - A.0.1  Duality Gaps without Intercepts
  - A.0.2  Duality Gaps with Intercepts

## 1  Introduction

SPAMS (SPArse Modeling Software) is an open-source optimization toolbox for
sparse estimation with licence GPLv3. It implements algorithms for solving
machine learning and signal processing problems involving sparse
regularizations.

The library is coded in C++, is compatible with Linux, Mac, and Windows 32bits
and 64bits Operating Systems. It is interfaced with Matlab, R and Python, but
can be called from any C++ application (by hacking the code a bit).

It requires an implementation of BLAS and LAPACK for performing linear algebra
operations. The ones shipped with matlab and R can be used, but also external
libraries such as atlas, the netlib implementation, or the Intel Math Kernel
Library can be used. It also exploits multi-core CPUs when this feature is
supported by the compiler, through OpenMP.

The current licence is GPLv3, which is available at
http://www.gnu.org/licenses/gpl.html. For other licensing possibilities
allowing its use in proprietary softwares, please contact the author.

Version 2.5 of SPAMS is divided into several “toolboxes” and has a few
additional miscellaneous functions:

- The Dictionary learning and matrix factorization toolbox
  contains the online learning technique of [20, 21] and its
  variants for solving various matrix factorization problems:
  - dictionary Learning for sparse coding;
  - sparse principal component analysis (seen as a sparse matrix factorization problem);
  - non-negative matrix factorization;
  - non-negative sparse coding;
  - dictionary learning with structured sparsity;
  - archetypal analysis [7, 37].
- The Sparse decomposition toolbox contains efficient implementations of
  - Orthogonal Matching Pursuit, (or Forward Selection) [35, 27];
  - the LARS/homotopy algorithm [30, 9] (variants for solving Lasso and Elastic-Net problems);
  - a weighted version of LARS;
  - OMP and LARS when data comes with a binary mask;
  - a coordinate-descent algorithm for ℓ_1_-decomposition problems [12, 10, 36];
  - a greedy solver for simultaneous signal approximation as defined in [34, 33] (SOMP);
  - a solver for simulatneous signal approximation with ℓ_1_/ℓ_2_-regularization based on block-coordinate descent;
  - a homotopy method for the Fused-Lasso Signal Approximation as defined in [10] with the homotopy method presented in the appendix of [21];
  - a tool for projecting efficiently onto a few convex sets
    inducing sparsity such as the ℓ_1_-ball using the method of
    [3, 18, 8], and Elastic-Net or Fused Lasso constraint sets as
    proposed in the appendix of [21].
  - an active-set algorithm for simplex decomposition problems [37].
- The Proximal toolbox: An implementation of proximal methods
  (ISTA and FISTA [1]) for solving a large class of sparse approximation
  problems with different combinations of loss and regularizations. One of the main
  features of this toolbox is to provide a robust stopping criterion based on
  *duality gaps* to control the quality of the optimization, whenever
  possible. It also handles sparse feature matrices for large-scale problems. The following regularizations are implemented:
  - Tikhonov regularization (squared ℓ_2_-norm);
  - ℓ_1_-norm, ℓ_2_, ℓ_∞_-norms;
  - Elastic-Net [39];
  - Fused Lasso [32];
  - tree-structured sum of ℓ_2_-norms (see [15, 16]);
  - tree-structured sum of ℓ_∞_-norms (see [15, 16]);
  - general sum of ℓ_∞_-norms (see [22, 23]);
  - mixed ℓ_1_/ℓ_2_-norms on matrices [38, 29];
  - mixed ℓ_1_/ℓ_∞_-norms on matrices [38, 29];
  - mixed ℓ_1_/ℓ_2_-norms on matrices plus ℓ_1_ [31, 11];
  - mixed ℓ_1_/ℓ_∞_-norms on matrices plus ℓ_1_;
  - group-lasso with ℓ_2_ or ℓ_∞_-norms;
  - group-lasso+ℓ_1_;
  - multi-task tree-structured sum of ℓ_∞_-norms (see [22, 23]);
  - trace norm;
  - ℓ_0_ pseudo-norm (only with ISTA);
  - tree-structured ℓ_0_ (only with ISTA);
  - rank regularization for matrices (only with ISTA);
  - the path-coding penalties of [24].
  All of these regularization functions can be used with the following losses
  - square loss;
  - square loss with missing observations;
  - logistic loss, weighted logistic loss;
  - multi-class logistic.
  This toolbox can also enforce non-negativity constraints, handle intercepts and
  sparse matrices. There are also a few additional undocumented functionalities,
  which are available in the source code.
  For some combinations of loss and regularizers, stochastic and incremental proximal
  gradient solvers are also implemented [26, 25].
- A few tools for performing linear algebra operations such as a
  conjugate gradient algorithm, manipulating sparse matrices and graphs.

The toolbox was written by Julien Mairal at INRIA, with the collaboration of
Francis Bach (INRIA), Jean Ponce (Ecole Normale Supérieure), Guillermo Sapiro
(University of Minnesota), Guillaume Obozinski (INRIA) and Rodolphe Jenatton
(INRIA).

R and Python interfaces have been written by Jean-Paul Chieze (INRIA).
The archetypal analysis implementation was written by Yuansi Chen, during
an internship at INRIA, with the collaboration of Zaid Harchaoui.

## 2  Installation

The toolbox used to come with pre-compiled binaries for various plateforms.
Now the sources are available and you will have to compile it yourself.

The user has the choice of the BLAS library, but the Intel MKL is recommended
for the best performance. Note that the builtin blas library of Matlab is a
version of the Intel MKL (not the most recent one though), and offers
a good performance.

The folder `doc` contains the documentation in pdf and html. `build/`
contains the binary files, including the help for each command.
`test_release` contains various matlab scripts which call the different
functions of this toolbox.

The software package comes also with a script bash that has to be used to
launch matlab for Linux and/or Mac OS versions. The install procedure is
described in a file called `HOW_TO_INSTALL` and in the file
`compile.m` for the Matlab version.

## 3  Dictionary Learning and Matrix Factorization Toolbox

This is the section for dictionary learning and matrix factorization, corresponding to [20, 21].

### 3.1  Function mexTrainDL

This is the main function of the toolbox, implementing the learning algorithms of [21].
Given a training set x^1^,…, . It aims at solving

|  |  |  |  |  |  |  |  |  |  |  |  |  |  |  |  |
| --- | --- | --- | --- | --- | --- | --- | --- | --- | --- | --- | --- | --- | --- | --- | --- |
|  | min; D ∈ C |  | lim; n → +∞ |  | 1; n |  | n; ∑; i=1 |  | min; α^i^ |  | ⎛  ⎜  ⎝ | 1; 2 | \|\|x^i^−Dα^i^\|\|_2_^2^ + ψ(α^i^) | ⎞  ⎟  ⎠ | .     (1) |

ψ is a sparsity-inducing regularizer and C is a constraint set for the dictionary. As shown in [21]
and in the help file below, various combinations can be used for ψ and C for solving different matrix factorization problems.
What is more, positivity constraints can be added to α as well. The function admits several modes for choosing the optimization parameters, using the parameter-free strategy proposed in [20], or using the parameters t_0_ and ρ presented
in [21]. Note that for problems of a reasonable size, and when ψ is the ℓ_1_-norm,
the function mexTrainDL\_Memory can be faster but uses more memory.

%   
% Usage:   [D [model]]=mexTrainDL(X,param[,model]);  
%          model is optional  
%  
% Name: mexTrainDL  
%  
% Description: mexTrainDL is an efficient implementation of the  
%     dictionary learning technique presented in  
%  
%     "Online Learning for Matrix Factorization and Sparse Coding"  
%     by Julien Mairal, Francis Bach, Jean Ponce and Guillermo Sapiro  
%     arXiv:0908.0050  
%       
%     "Online Dictionary Learning for Sparse Coding"        
%     by Julien Mairal, Francis Bach, Jean Ponce and Guillermo Sapiro  
%     ICML 2009.  
%  
%     Note that if you use param.mode=1 or 2, if the training set has a  
%     reasonable size and you have enough memory on your computer, you   
%     should use mexTrainDL\_Memory instead.  
%   
%  
%     It addresses the dictionary learning problems  
%        1) if param.mode=0  
%     min\_{D in C} (1/n) sum\_{i=1}^n (1/2)||x\_i-Dalpha\_i||\_2^2  s.t. ...  
%                                                  ||alpha\_i||\_1 <= lambda  
%        2) if param.mode=1  
%     min\_{D in C} (1/n) sum\_{i=1}^n  ||alpha\_i||\_1  s.t.  ...  
%                                           ||x\_i-Dalpha\_i||\_2^2 <= lambda  
%        3) if param.mode=2  
%     min\_{D in C} (1/n) sum\_{i=1}^n (1/2)||x\_i-Dalpha\_i||\_2^2 + ...   
%                                  lambda||alpha\_i||\_1 + lambda\_2||alpha\_i||\_2^2  
%        4) if param.mode=3, the sparse coding is done with OMP  
%     min\_{D in C} (1/n) sum\_{i=1}^n (1/2)||x\_i-Dalpha\_i||\_2^2  s.t. ...   
%                                                  ||alpha\_i||\_0 <= lambda  
%        5) if param.mode=4, the sparse coding is done with OMP  
%     min\_{D in C} (1/n) sum\_{i=1}^n  ||alpha\_i||\_0  s.t.  ...  
%                                           ||x\_i-Dalpha\_i||\_2^2 <= lambda  
%        6) if param.mode=5, the sparse coding is done with OMP  
%     min\_{D in C} (1/n) sum\_{i=1}^n 0.5||x\_i-Dalpha\_i||\_2^2 +lambda||alpha\_i||\_0    
%                                             
%  
%%     C is a convex set verifying  
%        1) if param.modeD=0  
%           C={  D in Real^{m x p}  s.t.  forall j,  ||d\_j||\_2^2 <= 1 }  
%        2) if param.modeD=1  
%           C={  D in Real^{m x p}  s.t.  forall j,  ||d\_j||\_2^2 + ...   
%                                                  gamma1||d\_j||\_1 <= 1 }  
%        3) if param.modeD=2  
%           C={  D in Real^{m x p}  s.t.  forall j,  ||d\_j||\_2^2 + ...   
%                                  gamma1||d\_j||\_1 + gamma2 FL(d\_j) <= 1 }  
%        4) if param.modeD=3  
%           C={  D in Real^{m x p}  s.t.  forall j,  (1-gamma1)||d\_j||\_2^2 + ...   
%                                  gamma1||d\_j||\_1 <= 1 }  
  
%  
%     Potentially, n can be very large with this algorithm.  
%  
% Inputs: X:  double m x n matrix   (input signals)  
%               m is the signal size  
%               n is the number of signals to decompose  
%         param: struct  
%            param.D: (optional) double m x p matrix   (dictionary)  
%              p is the number of elements in the dictionary  
%              When D is not provided, the dictionary is initialized   
%              with random elements from the training set.  
%           param.K (size of the dictionary, optional is param.D is provided)  
%           param.lambda  (parameter)  
%           param.lambda2  (optional, by default 0)  
%           param.iter (number of iterations).  If a negative number is   
%              provided it will perform the computation during the  
%              corresponding number of seconds. For instance param.iter=-5  
%              learns the dictionary during 5 seconds.  
%           param.mode (optional, see above, by default 2)   
%           param.posAlpha (optional, adds positivity constraints on the  
%             coefficients, false by default, not compatible with   
%             param.mode =3,4)  
%           param.modeD (optional, see above, by default 0)  
%           param.posD (optional, adds positivity constraints on the   
%             dictionary, false by default, not compatible with   
%             param.modeD=2)  
%           param.gamma1 (optional parameter for param.modeD >= 1)  
%           param.gamma2 (optional parameter for param.modeD = 2)  
%           param.batchsize (optional, size of the minibatch, by default   
%              512)  
%           param.iter\_updateD (optional, number of BCD iterations for the dictionary  
%              update step, by default 1)  
%           param.modeParam (optimization mode).  
%              1) if param.modeParam=0, the optimization uses the   
%                 parameter free strategy of the ICML paper  
%              2) if param.modeParam=1, the optimization uses the   
%                 parameters rho as in arXiv:0908.0050  
%              3) if param.modeParam=2, the optimization uses exponential   
%                 decay weights with updates of the form   
%                 A\_{t} <- rho A\_{t-1} + alpha\_t alpha\_t^T  
%           param.rho (optional) tuning parameter (see paper arXiv:0908.0050)  
%           param.t0 (optional) tuning parameter (see paper arXiv:0908.0050)  
%           param.clean (optional, true by default. prunes   
%              automatically the dictionary from unused elements).  
%           param.verbose (optional, true by default, increase verbosity)  
%           param.numThreads (optional, number of threads for exploiting  
%              multi-core / multi-cpus. By default, it takes the value -1,  
%              which automatically selects all the available CPUs/cores).  
%  
% Output:   
%         param.D: double m x p matrix   (dictionary)  
%  
% Note: this function admits a few experimental usages, which have not  
%     been extensively tested:  
%         - single precision setting   
%  
% Author: Julien Mairal, 2009

The following piece of code illustrates how to use this function.

clear all;  
  
I=double(imread('data/lena.png'))/255;  
% extract 8 x 8 patches  
X=im2col(I,[8 8],'sliding');  
X=X-repmat(mean(X),[size(X,1) 1]);  
X=X ./ repmat(sqrt(sum(X.^2)),[size(X,1) 1]);  
  
param.K=256;  % learns a dictionary with 100 elements  
param.lambda=0.15;  
param.numThreads=-1; % number of threads  
param.batchsize=400;  
param.verbose=false;  
  
param.iter=1000;  % let us see what happens after 1000 iterations.  
  
%%%%%%%%%% FIRST EXPERIMENT %%%%%%%%%%%  
tic  
D = mexTrainDL(X,param);  
t=toc;  
fprintf('time of computation for Dictionary Learning: %f\n',t);  
  
fprintf('Evaluating cost function...\n');  
alpha=mexLasso(X,D,param);  
R=mean(0.5\*sum((X-D\*alpha).^2)+param.lambda\*sum(abs(alpha)));  
ImD=displayPatches(D);  
subplot(1,3,1);  
imagesc(ImD); colormap('gray');  
fprintf('objective function: %f\n',R);  
drawnow;  
  
fprintf('\*\*\*\*\*\*\*\*\*\*\* SECOND EXPERIMENT \*\*\*\*\*\*\*\*\*\*\*\n');  
%%%%%%%%%% SECOND EXPERIMENT %%%%%%%%%%%  
% Train on half of the training set, then retrain on the second part  
X1=X(:,1:floor(size(X,2)/2));  
X2=X(:,floor(size(X,2)/2):end);  
param.iter=500;  
tic  
[D model] = mexTrainDL(X1,param);  
t=toc;  
fprintf('time of computation for Dictionary Learning: %f\n',t);  
fprintf('Evaluating cost function...\n');  
alpha=mexLasso(X,D,param);  
R=mean(0.5\*sum((X-D\*alpha).^2)+param.lambda\*sum(abs(alpha)));  
fprintf('objective function: %f\n',R);  
tic  
% Then reuse the learned model to retrain a few iterations more.  
param2=param;  
param2.D=D;  
[D model] = mexTrainDL(X2,param2,model);  
%[D] = mexTrainDL(X,param);  
t=toc;  
fprintf('time of computation for Dictionary Learning: %f\n',t);  
fprintf('Evaluating cost function...\n');  
alpha=mexLasso(X,D,param);  
R=mean(0.5\*sum((X-D\*alpha).^2)+param.lambda\*sum(abs(alpha)));  
fprintf('objective function: %f\n',R);  
  
% let us add sparsity to the dictionary itself  
fprintf('\*\*\*\*\*\*\*\*\*\*\* THIRD EXPERIMENT \*\*\*\*\*\*\*\*\*\*\*\n');  
param.modeParam=0;  
param.iter=1000;  
param.gamma1=0.3;  
param.modeD=1;  
tic  
[D] = mexTrainDL(X,param);  
t=toc;  
fprintf('time of computation for Dictionary Learning: %f\n',t);  
fprintf('Evaluating cost function...\n');  
alpha=mexLasso(X,D,param);  
R=mean(0.5\*sum((X-D\*alpha).^2)+param.lambda\*sum(abs(alpha)));  
fprintf('objective function: %f\n',R);  
tic  
subplot(1,3,2);  
ImD=displayPatches(D);  
imagesc(ImD); colormap('gray');  
drawnow;  
  
fprintf('\*\*\*\*\*\*\*\*\*\*\* FOURTH EXPERIMENT \*\*\*\*\*\*\*\*\*\*\*\n');  
param.modeParam=0;  
param.iter=1000;  
param.gamma1=0.3;  
param.modeD=3;  
tic  
[D] = mexTrainDL(X,param);  
t=toc;  
fprintf('time of computation for Dictionary Learning: %f\n',t);  
fprintf('Evaluating cost function...\n');  
alpha=mexLasso(X,D,param);  
R=mean(0.5\*sum((X-D\*alpha).^2)+param.lambda\*sum(abs(alpha)));  
fprintf('objective function: %f\n',R);  
tic  
subplot(1,3,3);  
ImD=displayPatches(D);  
imagesc(ImD); colormap('gray');  
drawnow;

### 3.2  Function mexTrainDL\_Memory

Memory-consuming version of mexTrainDL. This function is well adapted to small/medium-size problems:
It requires storing all the coefficients α and is therefore impractical
for very large datasets. However, in many situations, one can afford this memory cost and it is better to use this method, which
is faster than mexTrainDL.
Note that unlike mexTrainDL this function does not allow warm-restart.

%   
% Usage:   [D]=mexTrainDL(X,param);  
%  
% Name: mexTrainDL\_Memory  
%  
% Description: mexTrainDL\_Memory is an efficient but memory consuming   
%     variant of the dictionary learning technique presented in  
%  
%     "Online Learning for Matrix Factorization and Sparse Coding"  
%     by Julien Mairal, Francis Bach, Jean Ponce and Guillermo Sapiro  
%     arXiv:0908.0050  
%       
%     "Online Dictionary Learning for Sparse Coding"        
%     by Julien Mairal, Francis Bach, Jean Ponce and Guillermo Sapiro  
%     ICML 2009.  
%  
%     Contrary to the approaches above, the algorithm here   
%        does require to store all the coefficients from all the training  
%        signals. For this reason this variant can not be used with large  
%        training sets, but is more efficient than the regular online  
%        approach for training sets of reasonable size.  
%  
%     It addresses the dictionary learning problems  
%        1) if param.mode=1  
%     min\_{D in C} (1/n) sum\_{i=1}^n  ||alpha\_i||\_1  s.t.  ...  
%                                         ||x\_i-Dalpha\_i||\_2^2 <= lambda  
%        2) if param.mode=2  
%     min\_{D in C} (1/n) sum\_{i=1}^n (1/2)||x\_i-Dalpha\_i||\_2^2 + ...   
%                                                      lambda||alpha\_i||\_1    
%  
%     C is a convex set verifying  
%        1) if param.modeD=0  
%           C={  D in Real^{m x p}  s.t.  forall j,  ||d\_j||\_2^2 <= 1 }  
%        1) if param.modeD=1  
%           C={  D in Real^{m x p}  s.t.  forall j,  ||d\_j||\_2^2 + ...   
%                                                  gamma1||d\_j||\_1 <= 1 }  
%        1) if param.modeD=2  
%           C={  D in Real^{m x p}  s.t.  forall j,  ||d\_j||\_2^2 + ...   
%                                  gamma1||d\_j||\_1 + gamma2 FL(d\_j) <= 1 }  
%  
%     Potentially, n can be very large with this algorithm.  
%  
% Inputs: X:  double m x n matrix   (input signals)  
%               m is the signal size  
%               n is the number of signals to decompose  
%         param: struct  
%            param.D: (optional) double m x p matrix   (dictionary)  
%              p is the number of elements in the dictionary  
%              When D is not provided, the dictionary is initialized   
%              with random elements from the training set.  
%           param.K (size of the dictionary, optional is param.D is provided)  
%           param.lambda  (parameter)  
%           param.iter (number of iterations).  If a negative number is   
%              provided it will perform the computation during the  
%              corresponding number of seconds. For instance param.iter=-5  
%              learns the dictionary during 5 seconds.  
%            param.mode (optional, see above, by default 2)   
%            param.modeD (optional, see above, by default 0)  
%            param.posD (optional, adds positivity constraints on the   
%              dictionary, false by default, not compatible with   
%              param.modeD=2)  
%            param.gamma1 (optional parameter for param.modeD >= 1)  
%            param.gamma2 (optional parameter for param.modeD = 2)  
%            param.batchsize (optional, size of the minibatch, by default   
%              512)  
%            param.iter\_updateD (optional, number of BCD iterations for the dictionary   
%                update step, by default 1)  
%            param.modeParam (optimization mode).  
%              1) if param.modeParam=0, the optimization uses the   
%                 parameter free strategy of the ICML paper  
%              2) if param.modeParam=1, the optimization uses the   
%                 parameters rho as in arXiv:0908.0050  
%              3) if param.modeParam=2, the optimization uses exponential   
%                 decay weights with updates of the form   
%                 A\_{t} <- rho A\_{t-1} + alpha\_t alpha\_t^T  
%            param.rho (optional) tuning parameter (see paper arXiv:0908.0050)  
%            param.t0 (optional) tuning parameter (see paper arXiv:0908.0050)  
%            param.clean (optional, true by default. prunes   
%              automatically the dictionary from unused elements).  
%            param.numThreads (optional, number of threads for exploiting  
%              multi-core / multi-cpus. By default, it takes the value -1,  
%              which automatically selects all the available CPUs/cores).  
%  
% Output:   
%         param.D: double m x p matrix   (dictionary)  
%  
% Note: this function admits a few experimental usages, which have not  
%     been extensively tested:  
%         - single precision setting (even though the output alpha is double   
%           precision)  
%  
% Author: Julien Mairal, 2009

The following piece of code illustrates how to use this function.

clear all;  
  
I=double(imread('data/lena.png'))/255;  
% extract 8 x 8 patches  
X=im2col(I,[8 8],'sliding');  
X=X-repmat(mean(X),[size(X,1) 1]);  
X=X ./ repmat(sqrt(sum(X.^2)),[size(X,1) 1]);  
X=X(:,1:10:end);  
  
param.K=200;  % learns a dictionary with 100 elements  
param.lambda=0.15;  
param.numThreads=4; % number of threads  
  
param.iter=100;  % let us see what happens after 100 iterations.  
  
%%%%%%%%%% FIRST EXPERIMENT %%%%%%%%%%%  
tic  
D = mexTrainDL\_Memory(X,param);  
t=toc;  
fprintf('time of computation for Dictionary Learning: %f\n',t);  
  
fprintf('Evaluating cost function...\n');  
alpha=mexLasso(X,D,param);  
R=mean(0.5\*sum((X-D\*alpha).^2)+param.lambda\*sum(abs(alpha)));  
ImD=displayPatches(D);  
subplot(1,3,1);  
imagesc(ImD); colormap('gray');  
fprintf('objective function: %f\n',R);  
  
%%%%%%%%%% SECOND EXPERIMENT %%%%%%%%%%%  
tic  
D = mexTrainDL(X,param);  
t=toc;  
fprintf('time of computation for Dictionary Learning: %f\n',t);  
  
fprintf('Evaluating cost function...\n');  
alpha=mexLasso(X,D,param);  
R=mean(0.5\*sum((X-D\*alpha).^2)+param.lambda\*sum(abs(alpha)));  
ImD=displayPatches(D);  
subplot(1,3,2);  
imagesc(ImD); colormap('gray');  
fprintf('objective function: %f\n',R);

### 3.3  Function mexStructTrainDL

This function allows to use mexTrainDL with structured regularization functions
for the coefficients α. It internally uses the FISTA algorithm.

%   
% Usage:   [D [model]]=mexStructTrainDL(X,param[,model]);  
%          model is optional  
%  
% Name: mexStructTrainDL  
%  
% Description: mexStructTrainDL is an efficient implementation of the  
%     dictionary learning technique presented in  
%  
%     "Online Learning for Matrix Factorization and Sparse Coding"  
%     by Julien Mairal, Francis Bach, Jean Ponce and Guillermo Sapiro  
%     arXiv:0908.0050  
%       
%     "Online Dictionary Learning for Sparse Coding"        
%     by Julien Mairal, Francis Bach, Jean Ponce and Guillermo Sapiro  
%     ICML 2009.  
%  
%  
%     It addresses the dictionary learning problems  
%        min\_{D in C} (1/n) sum\_{i=1}^n 0.5||x\_i-Dalpha\_i||\_2^2 + lambda psi(alpha)  
%        where the regularization function psi depends on param.regul  
%        (see mexProximalFlat for the description of psi,  
%         and param.regul below for allowed values of regul)  
%  
%%     C is a convex set verifying  
%        1) if param.modeD=0  
%           C={  D in Real^{m x p}  s.t.  forall j,  ||d\_j||\_2^2 <= 1 }  
%        2) if param.modeD=1  
%           C={  D in Real^{m x p}  s.t.  forall j,  ||d\_j||\_2^2 + ...   
%                                                  gamma1||d\_j||\_1 <= 1 }  
%        3) if param.modeD=2  
%           C={  D in Real^{m x p}  s.t.  forall j,  ||d\_j||\_2^2 + ...   
%                                  gamma1||d\_j||\_1 + gamma2 FL(d\_j) <= 1 }  
%        4) if param.modeD=3  
%           C={  D in Real^{m x p}  s.t.  forall j,  (1-gamma1)||d\_j||\_2^2 + ...   
%                                  gamma1||d\_j||\_1 <= 1 }  
  
%  
%     Potentially, n can be very large with this algorithm.  
%  
% Inputs: X:  double m x n matrix   (input signals)  
%               m is the signal size  
%               n is the number of signals to decompose  
%         param: struct  
%            param.D: (optional) double m x p matrix   (dictionary)  
%              p is the number of elements in the dictionary  
%              When D is not provided, the dictionary is initialized   
%              with random elements from the training set.  
%           param.K (size of the dictionary, optional is param.D is provided)  
%           param.lambda  (parameter)  
%           param.lambda2  (optional, by default 0)  
%           param.lambda3 (optional, regularization parameter, 0 by default)  
%           param.iter (number of iterations).  If a negative number is   
%              provided it will perform the computation during the  
%              corresponding number of seconds. For instance param.iter=-5  
%              learns the dictionary during 5 seconds.  
%           param.regul choice of regularization : one of  
%               'l0' 'l1' 'l2' 'linf' 'none' 'elastic-net' 'fused-lasso'  
%               'graph' 'graph-ridge' 'graph-l2' 'tree-l0' 'tree-l2' 'tree-linf'   
%           param.tree struct (see documentation of mexProximalTree);  
%               needed for param.regul of graph kind.  
%           param.graph struct (see documentation of mexProximalGraph);  
%               needed for param.regul of tree kind.  
%           param.posAlpha (optional, adds positivity constraints on the  
%               coefficients, false by default.  
%           param.modeD (optional, see above, by default 0)  
%           param.posD (optional, adds positivity constraints on the   
%             dictionary, false by default, not compatible with   
%             param.modeD=2)  
%           param.gamma1 (optional parameter for param.modeD >= 1)  
%           param.gamma2 (optional parameter for param.modeD = 2)  
%           param.batchsize (optional, size of the minibatch, by default   
%              512)  
%           param.iter\_updateD (optional, number of BCD iterations for the dictionary  
%              update step, by default 1)  
%           param.modeParam (optimization mode).  
%              1) if param.modeParam=0, the optimization uses the   
%                 parameter free strategy of the ICML paper  
%              2) if param.modeParam=1, the optimization uses the   
%                 parameters rho as in arXiv:0908.0050  
%              3) if param.modeParam=2, the optimization uses exponential   
%                 decay weights with updates of the form   
%                 A\_{t} <- rho A\_{t-1} + alpha\_t alpha\_t^T  
%            param.ista (optional, use ista instead of fista, false by default).  
%            param.tol (optional, tolerance for stopping criteration, which is a relative duality gap  
%            param.fixed\_step (deactive the line search for L in fista and use param.K instead)  
%           param.rho (optional) tuning parameter (see paper arXiv:0908.0050)  
%           param.t0 (optional) tuning parameter (see paper arXiv:0908.0050)  
%           param.clean (optional, true by default. prunes   
%              automatically the dictionary from unused elements).  
%           param.verbose (optional, true by default, increase verbosity)  
%           param.numThreads (optional, number of threads for exploiting  
%              multi-core / multi-cpus. By default, it takes the value -1,  
%              which automatically selects all the available CPUs/cores).  
%  
% Output:   
%         param.D: double m x p matrix   (dictionary)  
%  
% Note: this function admits a few experimental usages, which have not  
%     been extensively tested:  
%         - single precision setting   
%  
% Author: Julien Mairal, 2009

The following piece of code illustrates how to use this function.

clear all;  
  
I=double(imread('data/lena.png'))/255;  
% extract 8 x 8 patches  
X=im2col(I,[8 8],'sliding');  
X=X-repmat(mean(X),[size(X,1) 1]);  
X=X ./ repmat(sqrt(sum(X.^2)),[size(X,1) 1]);  
  
param.K=64;  % learns a dictionary with 64 elements  
param.lambda=0.05;  
param.numThreads=4; % number of threads  
param.batchsize=400;  
param.tol = 1e-3  
  
param.iter=200;  %  
  
if false  
param.regul = 'l1';  
fprintf('with Fista Regression %s\n',param.regul);  
tic  
D = mexStructTrainDL(X,param);  
t=toc;  
fprintf('time of computation for Dictionary Learning: %f\n',t);  
%  
param.regul = 'l2';  
fprintf('with Fista Regression %s\n',param.regul);  
tic  
D = mexStructTrainDL(X,param);  
t=toc;  
fprintf('time of computation for Dictionary Learning: %f\n',t);  
%  
param.regul = 'elastic-net';  
fprintf('with Fista %s\n',param.regul);  
param.lambda2=0.1;  
tic  
D = mexStructTrainDL(X,param);  
t=toc;  
fprintf('time of computation for Dictionary Learning: %f\n',t);  
  
%%% GRAPH  
param.lambda=0.1; % regularization parameter  
param.tol=1e-5;  
param.K = 10  
graph.eta\_g=[1 1 1 1 1];  
graph.groups=sparse([0 0 0 1 0;  
                     0 0 0 0 0;  
                     0 0 0 0 0;  
                     0 0 0 0 0;  
                     0 0 1 0 0]);   % g5 is included in g3, and g2 is included in g4  
graph.groups\_var=sparse([1 0 0 0 0;  
                         1 0 0 0 0;  
                         1 0 0 0 0 ;  
                         1 1 0 0 0;  
                         0 1 0 1 0;  
                         0 1 0 1 0;  
                         0 1 0 0 1;  
                         0 0 0 0 1;  
                         0 0 0 0 1;  
                         0 0 1 0 0]); % represents direct inclusion relations   
  
param.graph = graph  
  
param.regul = 'graph';  
fprintf('with Fista %s\n',param.regul);  
tic  
D = mexStructTrainDL(X,param);  
t=toc;  
fprintf('time of computation for Dictionary Learning: %f\n',t);  
  
%%  
%%% TREE  
%?pause;  
param = rmfield(param,'graph');  
  
end  
  
  
param.lambda=0.1; % regularization parameter  
param.tol=1e-5;  
param.K = 10  
  
tree.own\_variables=  int32([0 0 3 5 6 6 8 9]);   % pointer to the first variable of each group  
tree.N\_own\_variables=int32([0 3 2 1 0 2 1 1]); % number of "root" variables in each group  
tree.eta\_g=[1 1 1 2 2 2 2.5 2.5];  
tree.groups=sparse([0 0 0 0 0 0 0 0; ...  
                    1 0 0 0 0 0 0 0; ...  
                    0 1 0 0 0 0 0 0; ...  
                    0 1 0 0 0 0 0 0; ...  
                    1 0 0 0 0 0 0 0; ...  
                    0 0 0 0 1 0 0 0; ...  
                    0 0 0 0 1 0 0 0; ...  
                    0 0 0 0 0 0 1 0]);  % first group should always be the root of the tree  
  
param.tree = tree;  
  
param.regul = 'tree-l0';  
fprintf('with Fista %s\n',param.regul);  
  
tic  
D = mexStructTrainDL(X,param);  
t=toc;  
fprintf('time of computation for Dictionary Learning: %f\n',t);  
  
%  
param.regul = 'tree-l2';  
fprintf('with Fista %s\n',param.regul);  
tic  
D = mexStructTrainDL(X,param);  
t=toc;  
fprintf('time of computation for Dictionary Learning: %f\n',t);  
  
%  
param.regul = 'tree-linf';  
fprintf('with Fista %s\n',param.regul);  
  
tic  
D = mexStructTrainDL(X,param);  
t=toc;  
fprintf('time of computation for Dictionary Learning: %f\n',t);

### 3.4  Function nmf

This function is an example on how to use the function mexTrainDL for the
problem of non-negative matrix factorization formulated in [17]. Note
that mexTrainDL can be replaced by mexTrainDL\_Memory in this function for
small or medium datasets.

%   
% Usage:   [U [,V]]=nmf(X,param);  
%  
% Name: nmf  
%  
% Description: mexTrainDL is an efficient implementation of the  
%     non-negative matrix factorization technique presented in   
%  
%     "Online Learning for Matrix Factorization and Sparse Coding"  
%     by Julien Mairal, Francis Bach, Jean Ponce and Guillermo Sapiro  
%     arXiv:0908.0050  
%       
%     "Online Dictionary Learning for Sparse Coding"        
%     by Julien Mairal, Francis Bach, Jean Ponce and Guillermo Sapiro  
%     ICML 2009.  
%  
%     Potentially, n can be very large with this algorithm.  
%  
% Inputs: X:  double m x n matrix   (input signals)  
%               m is the signal size  
%               n is the number of signals to decompose  
%         param: struct  
%            param.K (number of required factors)  
%            param.iter (number of iterations).  If a negative number   
%              is provided it will perform the computation during the  
%              corresponding number of seconds. For instance param.iter=-5  
%              learns the dictionary during 5 seconds.  
%            param.batchsize (optional, size of the minibatch, by default   
%               512)  
%            param.modeParam (optimization mode).  
%               1) if param.modeParam=0, the optimization uses the   
%                  parameter free strategy of the ICML paper  
%               2) if param.modeParam=1, the optimization uses the   
%                  parameters rho as in arXiv:0908.0050  
%               3) if param.modeParam=2, the optimization uses exponential   
%                  decay weights with updates of the form    
%                  A\_{t} <- rho A\_{t-1} + alpha\_t alpha\_t^T  
%            param.rho (optional) tuning parameter (see paper   
%              arXiv:0908.0050)  
%            param.t0 (optional) tuning parameter (see paper   
%              arXiv:0908.0050)  
%            param.clean (optional, true by default. prunes automatically   
%              the dictionary from unused elements).  
%            param.batch (optional, false by default, use batch learning   
%              instead of online learning)  
%            param.numThreads (optional, number of threads for exploiting  
%                 multi-core / multi-cpus. By default, it takes the value -1,  
%                 which automatically selects all the available CPUs/cores).  
%         model: struct (optional) learned model for "retraining" the data.  
%  
% Output:  
%         U: double m x p matrix     
%         V: double p x n matrix   (optional)  
%         model: struct (optional) learned model to be used for   
%           "retraining" the data.  
%  
% Author: Julien Mairal, 2009  
function [U V] = nmf(X,param)  
  
param.lambda=0;  
param.mode=2;  
param.posAlpha=1;  
param.posD=1;  
param.whiten=0;  
U=mexTrainDL(X,param);  
param.pos=1;  
if nargout == 2  
   if issparse(X) % todo allow sparse matrices X for mexLasso  
      maxbatch=ceil(10000000/size(X,1));  
      for jj = 1:maxbatch:size(X,2)  
         indbatch=jj:min((jj+maxbatch-1),size(X,2));  
         Xb=full(X(:,indbatch));  
         V(:,indbatch)=mexLasso(Xb,U,param);  
      end  
   else  
      V=mexLasso(X,U,param);  
   end  
end

The following piece of code illustrates how to use this function.

clear all;  
  
I=double(imread('data/lena.png'))/255;  
% extract 8 x 8 patches  
X=im2col(I,[16 16],'sliding');  
X=X(:,1:10:end);  
X=X ./ repmat(sqrt(sum(X.^2)),[size(X,1) 1]);  
  
param.K=49;  % learns a dictionary with 100 elements  
param.numThreads=4; % number of threads  
  
param.iter=-5;  % let us see what happens after 100 iterations.  
  
%%%%%%%%%% FIRST EXPERIMENT %%%%%%%%%%%  
tic  
[U V] = nmf(X,param);  
t=toc;  
fprintf('time of computation for Dictionary Learning: %f\n',t);  
  
fprintf('Evaluating cost function...\n');  
R=mean(0.5\*sum((X-U\*V).^2));  
ImD=displayPatches(U);  
imagesc(ImD); colormap('gray');  
fprintf('objective function: %f\n',R);

### 3.5  Function nnsc

This function is an example on how to use the function mexTrainDL for the
problem of non-negative sparse coding as defined in [14]. Note that
mexTrainDL can be replaced by mexTrainDL\_Memory in this function for small or
medium datasets.

%   
% Usage:   [U [,V]]=nnsc(X,param);  
%  
% Name: nmf  
%  
% Description: mexTrainDL is an efficient implementation of the  
%     non-negative sparse coding technique presented in   
%  
%     "Online Learning for Matrix Factorization and Sparse Coding"  
%     by Julien Mairal, Francis Bach, Jean Ponce and Guillermo Sapiro  
%     arXiv:0908.0050  
%       
%     "Online Dictionary Learning for Sparse Coding"        
%     by Julien Mairal, Francis Bach, Jean Ponce and Guillermo Sapiro  
%     ICML 2009.  
%  
%     Potentially, n can be very large with this algorithm.  
%  
% Inputs: X:  double m x n matrix   (input signals)  
%               m is the signal size  
%               n is the number of signals to decompose  
%         param: struct  
%           param.K (number of required factors)  
%           param.lambda (parameter)  
%           param.iter (number of iterations).  If a negative number   
%              is provided it will perform the computation during the  
%              corresponding number of seconds. For instance param.iter=-5  
%              learns the dictionary during 5 seconds.  
%           param.batchsize (optional, size of the minibatch, by default   
%              512)  
%           param.modeParam (optimization mode).  
%              1) if param.modeParam=0, the optimization uses the   
%                 parameter free strategy of the ICML paper  
%              2) if param.modeParam=1, the optimization uses the   
%                 parameters rho as in arXiv:0908.0050  
%              3) if param.modeParam=2, the optimization uses exponential   
%                 decay weights with updates of the form   
%                 A\_{t} <- rho A\_{t-1} + alpha\_t alpha\_t^T  
%           param.rho (optional) tuning parameter (see paper  
%              arXiv:0908.0050)  
%           param.t0 (optional) tuning parameter (see paper   
%              arXiv:0908.0050)  
%           param.clean (optional, true by default. prunes automatically   
%              the dictionary from unused elements).  
%           param.batch (optional, false by default, use batch learning   
%              instead of online learning)  
%           param.numThreads (optional, number of threads for exploiting  
%              multi-core / multi-cpus. By default, it takes the value -1,  
%              which automatically selects all the available CPUs/cores).  
%         model: struct (optional) learned model for "retraining" the data.  
%  
% Output:  
%         U: double m x p matrix     
%         V: double p x n matrix   (optional)  
%         model: struct (optional) learned model to be used for   
%            "retraining" the data.  
%  
% Author: Julien Mairal, 2009  
function [U V] = nnsc(X,param)  
  
param.mode=2;  
param.posAlpha=1;  
param.posD=1;  
param.whiten=0;  
U=mexTrainDL(X,param);  
param.pos=1;  
if nargout == 2  
   V=mexLasso(X,U,param);  
end

### 3.6  Function mexArchetypalAnalysis

The function optimizes the archetypal analysis formulation of [7]. It follows the
methodology presented in the following paper [37].

%   
% Usage:  [Z [A,B]]=mexArchetypalAnalysis(X,param);  
% Usage:  [Z [A,B]]=mexArchetypalAnalysis(X,Z,param);  
%  
% Name: mexArchetypalAnalysis  
%  
% documentation to appear soon  
%  
% Inputs: X:  double m x n matrix   (input signals)  
%               m is the signal size  
%               n is the number of signals to decompose  
% Output: Z: double %  
%  
% Author: Yuansi Chen and Julien Mairal, 2014

The following piece of code illustrates how to use this function.

clear all;  
rand('seed',0);  
I=double(imread('data/lena.png'))/255;  
% extract 8 x 8 patches  
X=im2col(I,[8 8],'sliding');  
per=randperm(size(X,2));  
X=X(:,per(1:10000));  
X=X-repmat(mean(X),[size(X,1) 1]);  
nrm=sqrt(sum(X.^2));  
me=median(nrm);  
X=X ./ repmat(max(nrm,me),[size(X,1) 1]);  
  
param.p=64;  % learns a dictionary with 64 elements  
param.robust=false;  
param.epsilon=1e-3;  % width for Huber loss  
param.computeXtX=true;  
param.stepsFISTA=0;  
param.stepsAS=10;  
param.numThreads=-1;  
  
%%%%%%%%%% FIRST EXPERIMENT %%%%%%%%%%%  
tic  
[Z A B] = mexArchetypalAnalysis(X,param);  
t=toc;  
fprintf('time of computation for Archetypal Analysis: %f\n',t);  
R=norm(X-Z\*A,'fro')^2;  
fprintf('objective function: %f\n',R);  
  
fprintf('Re-Evaluating cost function after re-updating A...\n');  
paramdecomp.computeXtX=true;  
paramdecomp.numThreads=-1;  
A2=mexDecompSimplex(X,Z,param);  
R=norm(X-Z\*A2,'fro')^2;  
fprintf('objective function: %f\n',R);  
  
%%%%%%%%%% Second EXPERIMENT %%%%%%%%%%%  
tic  
[Z2 A2] = mexArchetypalAnalysis(X,param,Z);  
t=toc;  
fprintf('time of computation for Archetypal Analysis: %f\n',t);  
fprintf('Evaluating cost function...\n');  
R=norm(X-Z2\*A2,'fro')^2;  
fprintf('objective function: %f\n',R);  
  
%%%%%%%%%% THIRD EXPERIMENT %%%%%%%%%%%  
tic  
param.robust=true;  
[Z3] = mexArchetypalAnalysis(X,param);  
t=toc;  
fprintf('time of computation for Robust Archetypal Analysis: %f\n',t);

## 4  Sparse Decomposition Toolbox

This toolbox implements several algorithms for solving signal reconstruction problems. It is mostly adapted for solving a large number of small/medium scale problems, but can be also efficient sometimes with large scale ones.

### 4.1  Function mexOMP

This is a fast implementation of the Orthogonal Matching Pursuit algorithm (or forward selection) [27, 35]. Given a matrix of signals X=[x^1^,…,x^n^] in ℝ^m × n^ and a dictionary D=[d^1^,…,d^p^] in ℝ^m × p^, the algorithm computes a matrix A=[α^1^,…,α^n^] in ℝ^p × n^,
where for each column x of X, it returns a coefficient vector α which is an approximate solution of the following NP-hard problem

min

α ∈ ℝ^p^

||x−Dα||_2_^2^   s.t.   ||α||_0_ ≤ L,
    (2)

or

min

α ∈ ℝ^p^

||α||_0_   s.t.   ||x−Dα||_2_^2^ ≤ ε,
    (3)

or

min

α ∈ ℝ^p^

1

2

||x−Dα||_2_^2^ + λ ||α||_0_.
    (4)

For efficienty reasons, the method first computes the covariance matrix
D^T^D, then for each signal, it computes D^T^x and performs the
decomposition with a Cholesky-based algorithm (see [6] for instance).

Note that mexOMP can return the “greedy” regularization path if needed (see below):

%   
% Usage:   A=mexOMP(X,D,param);  
% or       [A path]=mexOMP(X,D,param);  
%  
% Name: mexOMP  
%  
% Description: mexOMP is an efficient implementation of the  
%     Orthogonal Matching Pursuit algorithm. It is optimized  
%     for solving a large number of small or medium-sized   
%     decomposition problem (and not for a single large one).  
%     It first computes the Gram matrix D'D and then perform  
%     a Cholesky-based OMP of the input signals in parallel.  
%     X=[x^1,...,x^n] is a matrix of signals, and it returns  
%     a matrix A=[alpha^1,...,alpha^n] of coefficients.  
%       
%     it addresses for all columns x of X,   
%         min\_{alpha} ||alpha||\_0  s.t  ||x-Dalpha||\_2^2 <= eps  
%         or  
%         min\_{alpha} ||x-Dalpha||\_2^2  s.t. ||alpha||\_0 <= L  
%         or  
%         min\_{alpha} 0.5||x-Dalpha||\_2^2 + lambda||alpha||\_0   
%           
%  
% Inputs: X:  double m x n matrix   (input signals)  
%            m is the signal size  
%            n is the number of signals to decompose  
%         D:  double m x p matrix   (dictionary)  
%            p is the number of elements in the dictionary  
%            All the columns of D should have unit-norm !  
%         param: struct  
%            param.L (optional, maximum number of elements in each decomposition,   
%               min(m,p) by default)  
%            param.eps (optional, threshold on the squared l2-norm of the residual,  
%               0 by default  
%            param.lambda (optional, penalty parameter, 0 by default  
%            param.numThreads (optional, number of threads for exploiting  
%            multi-core / multi-cpus. By default, it takes the value -1,  
%            which automatically selects all the available CPUs/cores).  
%  
% Output: A: double sparse p x n matrix (output coefficients)  
%         path (optional): double dense p x L matrix (regularization path of the first signal)  
%  
% Note: this function admits a few experimental usages, which have not  
%     been extensively tested:  
%      - single precision setting (even though the output alpha is double   
%        precision)  
%      - Passing an int32 vector of length n to param.L provides  
%        a different parameter L for each input signal x\_i  
%      - Passing a double vector of length n to param.eps and or param.lambda   
%        provides a different parameter eps (or lambda) for each input signal x\_i  
%  
% Author: Julien Mairal, 2009

The following piece of code illustrates how to use this function.

clear all;  
randn('seed',0);  
  
fprintf('test mexOMP');  
  
%%%%%%%%%%%%%%%%%%%%%%%%%%%%%%%%%%%%%%%%%%%%%%  
% Decomposition of a large number of signals  
%%%%%%%%%%%%%%%%%%%%%%%%%%%%%%%%%%%%%%%%%%%%%%  
X=randn(64,100000);  
D=randn(64,200);  
D=D./repmat(sqrt(sum(D.^2)),[size(D,1) 1]);  
% parameter of the optimization procedure are chosen  
param.L=10; % not more than 10 non-zeros coefficients  
param.eps=0.1; % squared norm of the residual should be less than 0.1  
param.numThreads=-1; % number of processors/cores to use; the default choice is -1  
                    % and uses all the cores of the machine  
tic  
alpha=mexOMP(X,D,param);  
t=toc  
fprintf('%f signals processed per second\n',size(X,2)/t);  
  
%%%%%%%%%%%%%%%%%%%%%%%%%%%%%%%%%%%%%%%%%%  
% Regularization path of a single signal   
%%%%%%%%%%%%%%%%%%%%%%%%%%%%%%%%%%%%%%%%%%  
X=randn(64,1);  
D=randn(64,10);  
param.L=5;  
D=D./repmat(sqrt(sum(D.^2)),[size(D,1) 1]);  
[alpha path]=mexOMP(X,D,param);

### 4.2  Function mexOMPMask

This is a variant of mexOMP with the possibility of handling a binary mask.
Given a binary mask B=[β^1^,…,β^n^] in {0,1}^m × n^, it returns a matrix A=[α^1^,…,α^n^] such that for every column x of X, β of B, it computes a column α of A by addressing

min

α ∈ ℝ^p^

||diag(β)(x−Dα)||_2_^2^   s.t.   ||α||_0_ ≤ L,
    (5)

or

|  |  |  |  |  |
| --- | --- | --- | --- | --- |
|  | min; α ∈ ℝ^p^ | \|\|α\|\|_0_   s.t.   \|\|diag(β)(x−Dα)\|\|_2_^2^ ≤ ε | \|\|β\|\|_0_; m | ,     (6) |

or

min

α ∈ ℝ^p^

1

2

||diag(β)(x−Dα)||_2_^2^ +λ||α||_0_.
    (7)

where diag(β) is a diagonal matrix with the entries of β on the diagonal.

%   
% Usage:   A=mexOMPMask(X,D,B,param);  
% or       [A path]=mexOMPMask(X,D,B,param);  
%  
% Name: mexOMPMask  
%  
% Description: mexOMPMask is a variant of mexOMP that allow using  
%     a binary mask B  
%       
%     for all columns x of X, and columns beta of B, it computes a column   
%         alpha of A by addressing  
%         min\_{alpha} ||alpha||\_0  s.t  ||diag(beta)\*(x-Dalpha)||\_2^2   
%                                                               <= eps\*||beta||\_0/m  
%         or  
%         min\_{alpha} ||diag(beta)\*(x-Dalpha)||\_2^2  s.t. ||alpha||\_0 <= L  
%         or  
%         min\_{alpha} 0.5||diag(beta)\*(x-Dalpha)||\_2^2  + lambda||alpha||\_0  
%           
%  
% Inputs: X:  double m x n matrix   (input signals)  
%            m is the signal size  
%            n is the number of signals to decompose  
%         D:  double m x p matrix   (dictionary)  
%            p is the number of elements in the dictionary  
%            All the columns of D should have unit-norm !  
%         B:  boolean m x n matrix   (mask)  
%               p is the number of elements in the dictionary  
%         param: struct  
%            param.L (optional, maximum number of elements in each decomposition,   
%               min(m,p) by default)  
%            param.eps (optional, threshold on the squared l2-norm of the residual,  
%               0 by default  
%            param.lambda (optional, penalty parameter, 0 by default  
%            param.numThreads (optional, number of threads for exploiting  
%            multi-core / multi-cpus. By default, it takes the value -1,  
%            which automatically selects all the available CPUs/cores).  
%  
% Output: A: double sparse p x n matrix (output coefficients)  
%         path (optional): double dense p x L matrix   
%                                     (regularization path of the first signal)  
%  
% Note: this function admits a few experimental usages, which have not  
%     been extensively tested:  
%      - single precision setting (even though the output alpha is double   
%        precision)  
%      - Passing an int32 vector of length n to param.L provides  
%        a different parameter L for each input signal x\_i  
%      - Passing a double vector of length n to param.eps and or param.lambda   
%        provides a different parameter eps (or lambda) for each input signal x\_i  
%  
% Author: Julien Mairal, 2010

The following piece of code illustrates how to use this function.

clear all;  
  
randn('seed',0);  
fprintf('test mexOMPMask\n');  
  
%%%%%%%%%%%%%%%%%%%%%%%%%%%%%%%%%%%%%%%%%%%%%%  
% Decomposition of a large number of signals  
%%%%%%%%%%%%%%%%%%%%%%%%%%%%%%%%%%%%%%%%%%%%%%  
% Data are generated  
X=randn(100,100);  
X=X./repmat(sqrt(sum(X.^2)),[size(X,1) 1]);  
D=randn(100,20);  
D=D./repmat(sqrt(sum(D.^2)),[size(D,1) 1]);  
mask=(X > 0); % generating a binary mask  
  
% parameter of the optimization procedure are chosen  
param.L=20; % not more than 20 non-zeros coefficients (default: min(size(D,1),size(D,2)))  
param.eps=0.01; %  
param.numThreads=-1; % number of processors/cores to use; the default choice is -1  
                     % and uses all the cores of the machine  
tic  
alpha=mexOMPMask(X,D,mask,param);  
t=toc;  
toc  
  
fprintf('%f signals processed per second\n',size(X,2)/t);

### 4.3  Function mexRidgeRegression

This is a ridge regression solver using a conjugate gradient solver.

%   
% Usage: [W]=mexRidgeRegression(Y,X,W0,param);  
%  
% Name: mexRidgeRegression  
%  
% Description: mexFistaFlat solves sparse regularized problems.  
%         X is a design matrix of size m x p  
%         X=[x^1,...,x^n]', where the x\_i's are the rows of X  
%         Y=[y^1,...,y^n] is a matrix of size m x n  
%         It implements a conjugate gradient solver for ridge regression  
%           
% Inputs: Y:  double dense m x n matrix  
%         X:  double dense or sparse m x p matrix     
%         W0:  double dense p x n matrix or p x Nn matrix (for multi-logistic loss)  
%              initial guess  
%         param: struct  
%            param.lambda (regularization parameter)  
%            param.numThreads (optional, number of threads for exploiting  
%                multi-core / multi-cpus. By default, it takes the value -1,  
%                which automatically selects all the available CPUs/cores).  
%            param.itermax (optional, maximum number of iterations, 100 by default)  
%            param.tol (optional, tolerance for stopping criteration, which is a relative duality gap  
%                if it is available, or a relative change of parameters).  
%  
% Output:  W:  double dense p x n matrix   
  
% Author: Julien Mairal, 2013

The following piece of code illustrates how to use this function.

format compact;  
randn('seed',0);  
param.numThreads=-1; % all cores (-1 by default)  
param.lambda=0.05; % regularization parameter  
  
X=randn(100,200);  
X=X-repmat(mean(X),[size(X,1) 1]);  
X=mexNormalize(X);  
Y=randn(100,1);  
Y=Y-repmat(mean(Y),[size(Y,1) 1]);  
Y=mexNormalize(Y);  
W0=zeros(size(X,2),size(Y,2));  
% Regression experiments   
% 100 regression problems with the same design matrix X.  
fprintf('\nVarious regression experiments\n');  
fprintf('\nRidge Regression with conjugate gradient solver\n');  
tic  
[W]=mexRidgeRegression(Y,X,W0,param);  
t=toc

### 4.4  Function mexLasso

This is a fast implementation of the LARS algorithm [9] (variant for solving the Lasso) for solving the Lasso or Elastic-Net. Given a matrix of signals X=[x^1^,…,x^n^] in ℝ^m × n^ and a dictionary D in ℝ^m × p^, depending on the input parameters, the algorithm returns a matrix of coefficients A=[α^1^,…,α^n^] in ℝ^p × n^ such that for every column x of X, the corresponding column α of A is the solution of

min

α ∈ ℝ^p^

||x−Dα||_2_^2^   s.t.   ||α||_1_ ≤ λ,
    (8)

or

min

α ∈ ℝ^p^

||α||_1_   s.t.   ||x−Dα||_2_^2^ ≤ λ, 
    (9)

or

|  |  |  |  |  |  |  |
| --- | --- | --- | --- | --- | --- | --- |
|  | min; α ∈ ℝ^p^ |  | 1; 2 | \|\|x−Dα\|\|_2_^2^ + λ \|\|α\|\|_1_ + | λ_2_; 2 | \|\|α\|\|_2_^2^.      (10) |

For efficiency reasons, the method first compute the covariance matrix D^T^D, then
for each signal, it computes D^T^x and performs the decomposition with a
Cholesky-based algorithm (see [9] for instance). The implementation
has also an option to add positivity constraints on the solutions
α. When the solution is very sparse and the problem size is
reasonable, this approach can be very efficient. Moreover, it gives the
solution with an exact precision, and its performance does not depend on the
correlation of the dictionary elements, except when the solution is not unique
(the algorithm breaks in this case).

Note that mexLasso can return the whole regularization path of the first signal x_1_
and can handle implicitely the matrix D if the quantities D^T^D and D^T^x are passed
as an argument, see below:

%   
% Usage:  [A [path]]=mexLasso(X,D,param);  
%  or:    [A [path]]=mexLasso(X,Q,q,param);  
%  
% Name: mexLasso  
%  
% Description: mexLasso is an efficient implementation of the  
%     homotopy-LARS algorithm for solving the Lasso.   
%       
%     if the function is called this way [A [path]]=mexLasso(X,D,param),  
%     it aims at addressing the following problems  
%     for all columns x of X, it computes one column alpha of A  
%     that solves  
%       1) when param.mode=0  
%         min\_{alpha} ||x-Dalpha||\_2^2 s.t. ||alpha||\_1 <= lambda  
%       2) when param.mode=1  
%         min\_{alpha} ||alpha||\_1 s.t. ||x-Dalpha||\_2^2 <= lambda  
%       3) when param.mode=2  
%         min\_{alpha} 0.5||x-Dalpha||\_2^2 + lambda||alpha||\_1 +0.5 lambda2||alpha||\_2^2  
%  
%     if the function is called this way [A [path]]=mexLasso(X,Q,q,param),  
%     it solves the above optimisation problem, when Q=D'D and q=D'x.  
%  
%     Possibly, when param.pos=true, it solves the previous problems  
%     with positivity constraints on the vectors alpha  
%  
% Inputs: X:  double m x n matrix   (input signals)  
%               m is the signal size  
%               n is the number of signals to decompose  
%         D:  double m x p matrix   (dictionary)  
%               p is the number of elements in the dictionary  
%         param: struct  
%               param.lambda  (parameter)  
%               param.lambda2  (optional parameter for solving the Elastic-Net)  
%                              for mode=0 and mode=1, it adds a ridge on the Gram Matrix  
%               param.L (optional), maximum number of steps of the homotopy algorithm (can  
%                        be used as a stopping criterion)  
%               param.pos (optional, adds non-negativity constraints on the  
%                 coefficients, false by default)  
%               param.mode (see above, by default: 2)  
%               param.numThreads (optional, number of threads for exploiting  
%                 multi-core / multi-cpus. By default, it takes the value -1,  
%                 which automatically selects all the available CPUs/cores).  
%               param.cholesky (optional, default false),  choose between Cholesky   
%                 implementation or one based on the matrix inversion Lemma  
%               param.ols (optional, default false), perform an orthogonal projection  
%                 before returning the solution.  
%               param.max\_length\_path (optional) maximum length of the path, by default 4\*p  
%  
% Output: A: double sparse p x n matrix (output coefficients)  
%         path: optional,  returns the regularisation path for the first signal  
%  
% Note: this function admits a few experimental usages, which have not  
%     been extensively tested:  
%         - single precision setting (even though the output alpha is double   
%           precision)  
%  
% Author: Julien Mairal, 2009

The following piece of code illustrates how to use this function.

clear all;  
  
randn('seed',0);  
fprintf('test mexLasso\n');  
%%%%%%%%%%%%%%%%%%%%%%%%%%%%%%%%%%%%%%%%%%%%%%  
% Decomposition of a large number of signals  
%%%%%%%%%%%%%%%%%%%%%%%%%%%%%%%%%%%%%%%%%%%%%%  
% Data are generated  
X=randn(100,100000);  
X=X./repmat(sqrt(sum(X.^2)),[size(X,1) 1]);  
D=randn(100,200);  
D=D./repmat(sqrt(sum(D.^2)),[size(D,1) 1]);  
  
% parameter of the optimization procedure are chosen  
%param.L=20; % not more than 20 non-zeros coefficients (default: min(size(D,1),size(D,2)))  
param.lambda=0.15; % not more than 20 non-zeros coefficients  
param.numThreads=-1; % number of processors/cores to use; the default choice is -1  
                     % and uses all the cores of the machine  
param.mode=2;        % penalized formulation  
  
tic  
alpha=mexLasso(X,D,param);  
t=toc  
fprintf('%f signals processed per second\n',size(X,2)/t);  
  
%%%%%%%%%%%%%%%%%%%%%%%%%%%%%%%%%%%%%%%%%%  
% Regularization path of a single signal   
%%%%%%%%%%%%%%%%%%%%%%%%%%%%%%%%%%%%%%%%%%  
X=randn(64,1);  
D=randn(64,10);  
D=D./repmat(sqrt(sum(D.^2)),[size(D,1) 1]);  
param.lambda=0;  
[alpha path]=mexLasso(X,D,param);

### 4.5  Function mexLassoWeighted

This is a fast implementation of a weighted version of LARS [9]. Given a matrix of signals X=[x^1^,…,x^n^] in ℝ^m × n^, a matrix of weights W=[w^1^,…,w^n^] ∈ ℝ^p × n^, and a dictionary D in ℝ^m × p^, depending on the input parameters, the algorithm returns a matrix of coefficients A=[α^1^,…,α^n^] in ℝ^p × n^,
such that for every column x of X, w of W, it computes a column α of A, which is the solution of

min

α ∈ ℝ^p^

||x−Dα||_2_^2^   s.t.   ||diag(w)α||_1_ ≤ λ,
    (11)

or

min

α ∈ ℝ^p^

||diag(w)α||_1_   s.t.   ||x−Dα||_2_^2^ ≤ λ,
    (12)

or

min

α ∈ ℝ^p^

1

2

||x−Dα||_2_^2^ + λ ||diag(w)α||_1_.
    (13)

The implementation has also an option to add positivity constraints on
the solutions α. This function is potentially useful for
implementing efficiently the randomized Lasso of [28], or reweighted-ℓ_1_ schemes [4].

%   
% Usage:  A=mexLassoWeighted(X,D,W,param);  
%  
% Name: mexLassoWeighted.    
%  
% WARNING: This function has not been tested intensively  
%  
% Description: mexLassoWeighted is an efficient implementation of the  
%     LARS algorithm for solving the weighted Lasso. It is optimized  
%     for solving a large number of small or medium-sized   
%     decomposition problem (and not for a single large one).  
%     It first computes the Gram matrix D'D and then perform  
%     a Cholesky-based OMP of the input signals in parallel.  
%     For all columns x of X, and w of W, it computes one column alpha of A  
%     which is the solution of  
%       1) when param.mode=0  
%         min\_{alpha} ||x-Dalpha||\_2^2   s.t.    
%                                     ||diag(w)alpha||\_1 <= lambda  
%       2) when param.mode=1  
%         min\_{alpha} ||diag(w)alpha||\_1  s.t.  
%                                        ||x-Dalpha||\_2^2 <= lambda  
%       3) when param.mode=2  
%         min\_{alpha} 0.5||x-Dalpha||\_2^2  +    
%                                         lambda||diag(w)alpha||\_1   
%     Possibly, when param.pos=true, it solves the previous problems  
%     with positivity constraints on the vectors alpha  
%  
% Inputs: X:  double m x n matrix   (input signals)  
%               m is the signal size  
%               n is the number of signals to decompose  
%         D:  double m x p matrix   (dictionary)  
%               p is the number of elements in the dictionary  
%         W:  double p x n matrix   (weights)  
%         param: struct  
%            param.lambda  (parameter)  
%            param.L (optional, maximum number of elements of each   
%            decomposition)  
%            param.pos (optional, adds positivity constraints on the  
%            coefficients, false by default)  
%            param.mode (see above, by default: 2)  
%            param.numThreads (optional, number of threads for exploiting  
%            multi-core / multi-cpus. By default, it takes the value -1,  
%            which automatically selects all the available CPUs/cores).  
%  
% Output:  A: double sparse p x n matrix (output coefficients)  
%  
% Note: this function admits a few experimental usages, which have not  
%     been extensively tested:  
%         - single precision setting (even though the output alpha is double   
%           precision)  
%  
% Author: Julien Mairal, 2009

The following piece of code illustrates how to use this function.

clear all;  
  
fprintf('test Lasso weighted\n');  
randn('seed',0);  
% Data are generated  
X=randn(64,10000);  
X=X./repmat(sqrt(sum(X.^2)),[size(X,1) 1]);  
D=randn(64,256);  
D=D./repmat(sqrt(sum(D.^2)),[size(D,1) 1]);  
  
% parameter of the optimization procedure are chosen  
param.L=20; % not more than 20 non-zeros coefficients (default: min(size(D,1),size(D,2)))  
param.lambda=0.15; % not more than 20 non-zeros coefficients  
param.numThreads=8; % number of processors/cores to use; the default choice is -1  
                    % and uses all the cores of the machine  
param.mode=2;       % penalized formulation  
  
W=rand(size(D,2),size(X,2));  
  
tic  
alpha=mexLassoWeighted(X,D,W,param);  
t=toc;  
toc  
  
fprintf('%f signals processed per second\n',size(X,2)/t);

### 4.6  Function mexLassoMask

This is a variant of mexLasso with the possibility of adding a mask B=[β^1^,…,β^n^], as in mexOMPMask.
For every column x of X, β of B, it computes a column α of A, which is the solution of

min

α ∈ ℝ^p^

||diag(β)(x−Dα)||_2_^2^   s.t.   ||α||_1_ ≤ λ,
    (14)

or

|  |  |  |  |  |
| --- | --- | --- | --- | --- |
|  | min; α ∈ ℝ^p^ | \|\|α\|\|_1_   s.t.   \|\|diag(β)(x−Dα)\|\|_2_^2^ ≤ λ | \|\|β\|\|_0_; m | ,      (15) |

or

|  |  |  |  |  |  |  |  |  |
| --- | --- | --- | --- | --- | --- | --- | --- | --- |
|  | min; α ∈ ℝ^p^ |  | 1; 2 | \|\|diag(β)(x−Dα)\|\|_2_^2^ + λ | \|\|β\|\|_0_; m | \|\|α\|\|_1_ + | λ_2_; 2 | \|\|α\|\|_2_^2^.      (16) |

%   
% Usage:   A=mexLassoMask(X,D,B,param);  
%  
% Name: mexLassoMask  
%  
% Description: mexLasso is a variant of mexLasso that handles  
%     binary masks. It aims at addressing the following problems  
%     for all columns x of X, and beta of B, it computes one column alpha of A  
%     that solves  
%       1) when param.mode=0  
%         min\_{alpha} ||diag(beta)(x-Dalpha)||\_2^2 s.t. ||alpha||\_1 <= lambda  
%       2) when param.mode=1  
%         min\_{alpha} ||alpha||\_1 s.t. ||diag(beta)(x-Dalpha)||\_2^2   
%                                                              <= lambda\*||beta||\_0/m  
%       3) when param.mode=2  
%         min\_{alpha} 0.5||diag(beta)(x-Dalpha)||\_2^2 +  
%                                                 lambda\*(||beta||\_0/m)\*||alpha||\_1 +  
%                                                 (lambda2/2)||alpha||\_2^2  
%     Possibly, when param.pos=true, it solves the previous problems  
%     with positivity constraints on the vectors alpha  
%  
% Inputs: X:  double m x n matrix   (input signals)  
%               m is the signal size  
%               n is the number of signals to decompose  
%         D:  double m x p matrix   (dictionary)  
%               p is the number of elements in the dictionary  
%         B:  boolean m x n matrix   (mask)  
%               p is the number of elements in the dictionary  
%         param: struct  
%               param.lambda  (parameter)  
%               param.L (optional, maximum number of elements of each   
%                 decomposition)  
%               param.pos (optional, adds positivity constraints on the  
%                 coefficients, false by default)  
%               param.mode (see above, by default: 2)  
%               param.lambda2  (optional parameter for solving the Elastic-Net)  
%                              for mode=0 and mode=1, it adds a ridge on the Gram Matrix  
%               param.numThreads (optional, number of threads for exploiting  
%                 multi-core / multi-cpus. By default, it takes the value -1,  
%                 which automatically selects all the available CPUs/cores).  
%  
% Output: A: double sparse p x n matrix (output coefficients)  
%  
% Note: this function admits a few experimental usages, which have not  
%     been extensively tested:  
%         - single precision setting (even though the output alpha is double   
%           precision)  
%  
% Author: Julien Mairal, 2010

The following piece of code illustrates how to use this function.

clear all;  
  
randn('seed',0);  
fprintf('test mexLasso\n');  
  
%%%%%%%%%%%%%%%%%%%%%%%%%%%%%%%%%%%%%%%%%%%%%%  
% Decomposition of a large number of signals  
%%%%%%%%%%%%%%%%%%%%%%%%%%%%%%%%%%%%%%%%%%%%%%  
% Data are generated  
X=randn(100,100);  
X=X./repmat(sqrt(sum(X.^2)),[size(X,1) 1]);  
D=randn(100,20);  
D=D./repmat(sqrt(sum(D.^2)),[size(D,1) 1]);  
mask=(X > 0); % generating a binary mask  
  
% parameter of the optimization procedure are chosen  
%param.L=20; % not more than 20 non-zeros coefficients (default: min(size(D,1),size(D,2)))  
param.lambda=0.15; % not more than 20 non-zeros coefficients  
param.numThreads=-1; % number of processors/cores to use; the default choice is -1  
                     % and uses all the cores of the machine  
param.mode=2;        % penalized formulation  
  
tic  
alpha=mexLassoMask(X,D,mask,param);  
t=toc;  
toc  
  
fprintf('%f signals processed per second\n',size(X,2)/t);

### 4.7  Function mexCD

Coordinate-descent approach for solving Eq. (10) and
Eq. (9). Note that unlike mexLasso, it is not implemented to solve the Elastic-Net formulation.
To solve Eq. (9), the algorithm solves a
sequence of problems of the form (10) using simple heuristics.
Coordinate descent is very simple and in practice very powerful. It performs
better when the correlation between the dictionary elements is small.

%   
% Usage:   A=mexCD(X,D,A0,param);  
%  
% Name: mexCD  
%  
% Description: mexCD addresses l1-decomposition problem with a   
%     coordinate descent type of approach.  
%     It is optimized for solving a large number of small or medium-sized   
%     decomposition problem (and not for a single large one).  
%     It first computes the Gram matrix D'D.  
%     This method is particularly well adapted when there is low   
%     correlation between the dictionary elements and when one can benefit   
%     from a warm restart.  
%     It aims at addressing the two following problems  
%     for all columns x of X, it computes a column alpha of A such that  
%       2) when param.mode=1  
%         min\_{alpha} ||alpha||\_1 s.t. ||x-Dalpha||\_2^2 <= lambda  
%         For this constraint setting, the method solves a sequence of   
%         penalized problems (corresponding to param.mode=2) and looks  
%         for the corresponding Lagrange multplier with a simple but  
%         efficient heuristic.  
%       3) when param.mode=2  
%         min\_{alpha} 0.5||x-Dalpha||\_2^2 + lambda||alpha||\_1   
%  
% Inputs: X:  double m x n matrix   (input signals)  
%               m is the signal size  
%               n is the number of signals to decompose  
%         D:  double m x p matrix   (dictionary)  
%               p is the number of elements in the dictionary  
%               All the columns of D should have unit-norm !  
%         A0:  double sparse p x n matrix   (initial guess)  
%         param: struct  
%            param.lambda  (parameter)  
%            param.mode (optional, see above, by default 2)  
%            param.itermax (maximum number of iterations)  
%            param.tol (tolerance parameter)  
%            param.numThreads (optional, number of threads for exploiting  
%            multi-core / multi-cpus. By default, it takes the value -1,  
%            which automatically selects all the available CPUs/cores).  
%  
% Output: A: double sparse p x n matrix (output coefficients)  
%  
% Note: this function admits a few experimental usages, which have not  
%     been extensively tested:  
%         - single precision setting (even though the output alpha   
%           is double precision)  
%  
% Author: Julien Mairal, 2009

The following piece of code illustrates how to use this function.

clear all;  
  
fprintf('test mexCD\n');  
randn('seed',0);  
% Data are generated  
X=randn(64,100);  
X=X./repmat(sqrt(sum(X.^2)),[size(X,1) 1]);  
D=randn(64,100);  
D=D./repmat(sqrt(sum(D.^2)),[size(D,1) 1]);  
  
% parameter of the optimization procedure are chosen  
param.lambda=0.015; % not more than 20 non-zeros coefficients  
param.numThreads=4; % number of processors/cores to use; the default choice is -1  
param.mode=2;       % penalized formulation  
  
tic  
alpha=mexLasso(X,D,param);  
t=toc;  
toc  
E=mean(0.5\*sum((X-D\*alpha).^2)+param.lambda\*sum(abs(alpha)));  
fprintf('%f signals processed per second for LARS\n',size(X,2)/t);  
fprintf('Objective function for LARS: %g\n',E);  
  
param.tol=0.001;  
param.itermax=1000;  
tic  
alpha2=mexCD(X,D,sparse(size(alpha,1),size(alpha,2)),param);  
t=toc;  
toc  
  
fprintf('%f signals processed per second for CD\n',size(X,2)/t);  
E=mean(0.5\*sum((X-D\*alpha2).^2)+param.lambda\*sum(abs(alpha2)));  
fprintf('Objective function for CD: %g\n',E);  
fprintf('With Random Design, CD can be much faster than LARS\n');

### 4.8  Function mexSOMP

This is a fast implementation of the Simultaneous Orthogonal Matching Pursuit algorithm. Given a set of matrices X=[X^1^,…,X^n^] in ℝ^m × N^, where the X^i^’s are in ℝ^m × n_i_^, and a dictionary D in ℝ^m × p^, the algorithm returns a matrix of coefficients A=[A^1^,…,A^n^] in ℝ^p × N^ which is an approximate solution of the following NP-hard problem

|  |  |  |
| --- | --- | --- |
| ∀ i | min; A^i^ ∈ ℝ^p × n_i_^ | \|\|X^i^−DA^i^\|\|_F_^2^   s.t.   \|\|A^i^\|\|_0,∞_ ≤ L.     (17) |

or

|  |  |  |
| --- | --- | --- |
| ∀ i | min; A^i^ ∈ ℝ^p × n_i_^ | \|\|A^i^\|\|_0,∞_   s.t.   \|\|X^i^−DA^i^\|\|_F_^2^ ≤ ε n_i_.     (18) |

To be efficient, the method first compute the covariance matrix D^T^D, then for each signal, it computes D^T^X^i^ and performs the decomposition with a Cholesky-based algorithm.

%   
% Usage:   alpha=mexSOMP(X,D,list\_groups,param);  
%  
% Name: mexSOMP  
%     (this function has not been intensively tested).  
%  
% Description: mexSOMP is an efficient implementation of a  
%     Simultaneous Orthogonal Matching Pursuit algorithm. It is optimized  
%     for solving a large number of small or medium-sized   
%     decomposition problem (and not for a single large one).  
%     It first computes the Gram matrix D'D and then perform  
%     a Cholesky-based OMP of the input signals in parallel.  
%     It aims at addressing the following NP-hard problem  
%  
%     X is a matrix structured in groups of signals, which we denote  
%     by X=[X\_1,...,X\_n]  
%       
%     for all matrices X\_i of X,   
%         min\_{A\_i} ||A\_i||\_{0,infty}  s.t  ||X\_i-D A\_i||\_2^2 <= eps\*n\_i  
%         where n\_i is the number of columns of X\_i  
%  
%         or  
%  
%         min\_{A\_i} ||X\_i-D A\_i||\_2^2  s.t. ||A\_i||\_{0,infty} <= L  
%           
%  
% Inputs: X:  double m x N matrix   (input signals)  
%            m is the signal size  
%            N is the total number of signals   
%         D:  double m x p matrix   (dictionary)  
%            p is the number of elements in the dictionary  
%            All the columns of D should have unit-norm !  
%         list\_groups : int32 vector containing the indices (starting at 0)  
%            of the first elements of each groups.  
%         param: struct  
%            param.L (maximum number of elements in each decomposition)  
%            param.eps (threshold on the squared l2-norm of the residual  
%            param.numThreads (optional, number of threads for exploiting  
%            multi-core / multi-cpus. By default, it takes the value -1,  
%            which automatically selects all the available CPUs/cores).  
%  
% Output: alpha: double sparse p x N matrix (output coefficients)  
%  
% Note: this function admits a few experimental usages, which have not  
%     been extensively tested:  
%      - single precision setting (even though the output alpha is double   
%        precision)  
%  
% Author: Julien Mairal, 2010

The following piece of code illustrates how to use this function.

clear all;  
randn('seed',0);  
  
fprintf('test mexSOMP\n');  
  
%%%%%%%%%%%%%%%%%%%%%%%%%%%%%%%%%%%%%%%%%%%%%%  
% Decomposition of a large number of groups   
%%%%%%%%%%%%%%%%%%%%%%%%%%%%%%%%%%%%%%%%%%%%%%  
X=randn(64,10000);  
D=randn(64,200);  
D=D./repmat(sqrt(sum(D.^2)),[size(D,1) 1]);  
ind\_groups=int32(0:10:9999); % indices of the first signals in each group  
  
% parameter of the optimization procedure are chosen  
param.L=10; % not more than 10 non-zeros coefficients  
param.eps=0.1; % squared norm of the residual should be less than 0.1  
param.numThreads=-1; % number of processors/cores to use; the default choice is -1  
                    % and uses all the cores of the machine  
tic  
alpha=mexSOMP(X,D,ind\_groups,param);  
t=toc  
fprintf('%f signals processed per second\n',size(X,2)/t);

### 4.9  Function mexL1L2BCD

This is a fast implementation of a simultaneous signal decomposition formulation. Given a set of matrices X=[X^1^,…,X^n^] in ℝ^m × N^, where the X^i^’s are in ℝ^m × n_i_^, and a dictionary D in ℝ^m × p^, the algorithm returns a matrix of coefficients A=[A^1^,…,A^n^] in ℝ^p × N^ which is an approximate solution of the following NP-hard problem

|  |  |  |  |  |
| --- | --- | --- | --- | --- |
| ∀ i | min; A^i^ ∈ ℝ^p × n_i_^ | \|\|X^i^−DA^i^\|\|_F_^2^   s.t.   \|\|A^i^\|\|_1,2_ ≤ | λ; n_i_ | .     (19) |

or

|  |  |  |
| --- | --- | --- |
| ∀ i | min; A^i^ ∈ ℝ^p × n_i_^ | \|\|A^i^\|\|_1,2_   s.t.   \|\|X^i^−DA^i^\|\|_F_^2^ ≤ λ n_i_.     (20) |

To be efficient, the method first compute the covariance matrix D^T^D, then for each signal, it computes D^T^X^i^ and performs the decomposition with a Cholesky-based algorithm.

%   
% Usage:   alpha=mexL1L2BCD(X,D,alpha0,list\_groups,param);  
%  
% Name: mexL1L2BCD  
%     (this function has not been intensively tested).  
%  
% Description: mexL1L2BCD is a solver for a   
%     Simultaneous signal decomposition formulation based on block   
%     coordinate descent.  
%  
%     X is a matrix structured in groups of signals, which we denote  
%     by X=[X\_1,...,X\_n]  
%       
%     if param.mode=2, it solves  
%         for all matrices X\_i of X,   
%         min\_{A\_i} 0.5||X\_i-D A\_i||\_2^2 + lambda/sqrt(n\_i)||A\_i||\_{1,2}    
%         where n\_i is the number of columns of X\_i  
%     if param.mode=1, it solves  
%         min\_{A\_i} ||A\_i||\_{1,2} s.t. ||X\_i-D A\_i||\_2^2  <= n\_i lambda  
%           
%  
% Inputs: X:  double m x N matrix   (input signals)  
%            m is the signal size  
%            N is the total number of signals   
%         D:  double m x p matrix   (dictionary)  
%            p is the number of elements in the dictionary  
%         alpha0: double dense p x N matrix (initial solution)  
%         list\_groups : int32 vector containing the indices (starting at 0)  
%            of the first elements of each groups.  
%         param: struct  
%            param.lambda (regularization parameter)  
%            param.mode (see above, by default 2)  
%            param.itermax (maximum number of iterations, by default 100)  
%            param.tol (tolerance parameter, by default 0.001)  
%            param.numThreads (optional, number of threads for exploiting  
%            multi-core / multi-cpus. By default, it takes the value -1,  
%            which automatically selects all the available CPUs/cores).  
%  
% Output: alpha: double sparse p x N matrix (output coefficients)  
%  
% Note: this function admits a few experimental usages, which have not  
%     been extensively tested:  
%      - single precision setting (even though the output alpha is double   
%        precision)  
%  
% Author: Julien Mairal, 2010

The following piece of code illustrates how to use this function.

clear all;  
randn('seed',0);  
  
fprintf('test mexL1L2BCD\n');  
%%%%%%%%%%%%%%%%%%%%%%%%%%%%%%%%%%%%%%%%%%%%%%  
% Decomposition of a large number of groups   
%%%%%%%%%%%%%%%%%%%%%%%%%%%%%%%%%%%%%%%%%%%%%%  
X=randn(64,100);  
D=randn(64,200);  
D=D./repmat(sqrt(sum(D.^2)),[size(D,1) 1]);  
ind\_groups=int32(0:10:size(X,2)-1); % indices of the first signals in each group  
  
% parameter of the optimization procedure are chosen  
param.itermax=100;  
param.tol=1e-3;  
param.mode=2; % penalty mode  
param.lambda=0.15; % squared norm of the residual should be less than 0.1  
param.numThreads=-1; % number of processors/cores to use; the default choice is -1  
                    % and uses all the cores of the machine  
tic  
alpha0=zeros(size(D,2),size(X,2));  
alpha=mexL1L2BCD(X,D,alpha0,ind\_groups,param);  
t=toc  
fprintf('%f signals processed per second\n',size(X,2)/t);

### 4.10  Function mexSparseProject

This is a multi-purpose function, implementing fast algorithms for projecting
on convex sets, but it also solves the fused lasso signal approximation
problem. The proposed method is detailed in [21]. The main problems
addressed by this function are the following: Given a matrix
U=[u_1_,…,u_n_] in ℝ^m × n^, it finds a matrix
V=[v_1_,…,v_n_] in ℝ^m × n^ so that for all column u of U,
it computes a column v of V solving

min

v ∈ ℝ^m^

||u−v||_2_^2^    s.t.   ||v||_1_ ≤ τ,
    (21)

or

min

v ∈ ℝ^m^

||u−v||_2_^2^    s.t.   λ_1_||v||_1_ +λ_2_||v||_2_^2^≤ τ,
    (22)

or

min

v ∈ ℝ^m^

||u−v||_2_^2^    s.t.   λ_1_||v||_1_ +λ_2_||v||_2_^2^+ λ_3_ FL(v) ≤ τ,
    (23)

or

min

v ∈ ℝ^m^

1

2

||u−v||_2_^2^ + λ_1_||v||_1_ +λ_2_||v||_2_^2^+ λ_3_ FL(v).
    (24)

Note that for the two last cases, the method performs a small approximation.
The method follows the regularization path, goes from one kink to another, and
stop whenever the constraint is not satisfied anymore. The solution returned
by the algorithm is the one obtained at the last kink of the regularization path,
which is in practice close, but not exactly the same as the solution.
This will be corrected in a future release of the toolbox.

%   
% Usage:  V=mexSparseProject(U,param);  
%  
% Name: mexSparseProject  
%  
% Description: mexSparseProject solves various optimization   
%     problems, including projections on a few convex sets.  
%     It aims at addressing the following problems  
%     for all columns u of U in parallel  
%       1) when param.mode=1 (projection on the l1-ball)  
%           min\_v ||u-v||\_2^2  s.t.  ||v||\_1 <= thrs  
%       2) when param.mode=2  
%           min\_v ||u-v||\_2^2  s.t. ||v||\_2^2 + lamuda1||v||\_1 <= thrs  
%       3) when param.mode=3  
%           min\_v ||u-v||\_2^2  s.t  ||v||\_1 + 0.5lamuda1||v||\_2^2 <= thrs   
%       4) when param.mode=4  
%           min\_v 0.5||u-v||\_2^2 + lamuda1||v||\_1  s.t  ||v||\_2^2 <= thrs  
%       5) when param.mode=5  
%           min\_v 0.5||u-v||\_2^2 + lamuda1||v||\_1 +lamuda2 FL(v) + ...   
%                                                   0.5lamuda\_3 ||v||\_2^2  
%          where FL denotes a "fused lasso" regularization term.  
%       6) when param.mode=6  
%          min\_v ||u-v||\_2^2 s.t lamuda1||v||\_1 +lamuda2 FL(v) + ...  
%                                             0.5lamuda3||v||\_2^2 <= thrs  
%             
%        When param.pos=true and param.mode <= 4,  
%        it solves the previous problems with positivity constraints   
%  
% Inputs: U:  double m x n matrix   (input signals)  
%               m is the signal size  
%               n is the number of signals to project  
%         param: struct  
%           param.thrs (parameter)  
%           param.lambda1 (parameter)  
%           param.lambda2 (parameter)  
%           param.lambda3 (parameter)  
%           param.mode (see above)  
%           param.pos (optional, false by default)  
%           param.numThreads (optional, number of threads for exploiting  
%             multi-core / multi-cpus. By default, it takes the value -1,  
%             which automatically selects all the available CPUs/cores).  
%  
% Output: V: double m x n matrix (output matrix)  
%  
% Note: this function admits a few experimental usages, which have not  
%     been extensively tested:  
%         - single precision setting   
%  
% Author: Julien Mairal, 2009

The following piece of code illustrates how to use this function.

clear all;  
randn('seed',0);  
% Data are generated  
X=randn(20000,100);  
X=X./repmat(sqrt(sum(X.^2)),[size(X,1) 1]);  
  
% parameter of the optimization procedure are chosen  
param.numThreads=-1; % number of processors/cores to use; the default choice is -1  
                    % and uses all the cores of the machine  
  
param.pos=0;  
param.mode=1;       % projection on the l1 ball  
param.thrs=2;  
tic  
X1=mexSparseProject(X,param);  
t=toc;  
toc  
fprintf('%f signals of size %d projected per second\n',size(X,2)/t,size(X,1));  
fprintf('Checking constraint: %f, %f\n',min(sum(abs(X1))),max(sum(abs(X1))));  
  
  
param.mode=2;       % projection on the Elastic-Net  
param.lambda1=0.15;  
  
tic  
X1=mexSparseProject(X,param);  
t=toc;  
toc  
fprintf('%f signals of size %d projected per second\n',size(X,2)/t,size(X,1));  
constraints=sum((X1.^2))+param.lambda1\*sum(abs(X1));  
fprintf('Checking constraint: %f, %f\n',min(constraints),max(constraints));  
  
param.mode=6;       % projection on the FLSA  
param.lambda1=0.7;  
param.lambda2=0.7;  
param.lambda3=1.0;  
  
X=rand(2000,100);  
X=X./repmat(sqrt(sum(X.^2)),[size(X,1) 1]);  
  
tic  
X1=mexSparseProject(X,param);  
t=toc;  
toc  
fprintf('%f signals of size %d projected per second\n',size(X,2)/t,size(X,1));  
constraints=0.5\*param.lambda3\*sum(X1.^2)+param.lambda1\*sum(abs(X1))+param.lambda2\*sum(abs(X1(2:end,:)-X1(1:end-1,:)));  
fprintf('Checking constraint: %f, %f\n',mean(constraints),max(constraints));  
fprintf('Projection is approximate (stops at a kink)\n',mean(constraints),max(constraints));  
  
param.mode=6;       % projection on the FLSA  
param.lambda1=0.7;  
param.lambda2=0.7;  
param.lambda3=1.0;  
  
X=rand(2000,100);  
X=X./repmat(sqrt(sum(X.^2)),[size(X,1) 1]);  
  
tic  
X1=mexSparseProject(X,param);  
t=toc;  
toc  
fprintf('%f signals of size %d projected per second\n',size(X,2)/t,size(X,1));  
constraints=0.5\*param.lambda3\*sum(X1.^2)+param.lambda1\*sum(abs(X1))+param.lambda2\*sum(abs(X1(2:end,:)-X1(1:end-1,:)));  
fprintf('Checking constraint: %f, %f\n',mean(constraints),max(constraints));  
fprintf('Projection is approximate (stops at a kink)\n',mean(constraints),max(constraints));

### 4.11  Function mexDecompSimplex

The function implements an active-set algorithm [37] for solving

|  |  |  |  |  |  |  |
| --- | --- | --- | --- | --- | --- | --- |
|  | min; α ∈ ℝ^p^ |  | 1; 2 | \|\|x−Dα\|\|_2_^2^   s.t.   α ≥ 0   and | p; ∑; j=1 | α[j] = 1. |

%   
% Usage:  [A]=mexDecompSimplex(X,Z,param);  
%  
% Name: mexDecompSimplex  
%  
% documentation to appear soon  
%  
% Inputs: X:  double m x n matrix   (input signals)  
%               m is the signal size  
%               n is the number of signals to decompose  
% Output: Z: double %  
%  
% Author: Yuansi Chen and Julien Mairal, 2014

## 5  Proximal Toolbox

The previous toolbox we have presented is well
adapted for solving a large number of small and medium-scale sparse
decomposition problems with the square loss, which is typical from the
classical dictionary learning framework. We now present
a new software package that is adapted for solving a wide range of
possibly large-scale learning problems, with several combinations of losses and
regularization terms. The method implements the proximal methods
of [1], and includes the proximal solvers for the tree-structured
regularization of [15], and the solver of [22] for
general structured sparse regularization.
The solver for structured sparse regularization norms includes a C++ max-flow
implementation of the push-relabel algorithm of [13], with
heuristics proposed by [5].

This implementation also provides robust stopping criteria based on
*duality gaps*, which are presented in Appendix A. It can handle intercepts (unregularized variables). The general formulation that our software
can solve take the form

|  |  |  |  |
| --- | --- | --- | --- |
| min; w ∈ ℝ^p^ | [g(w) | ▵; = | f(w) + λψ(w)], |

where f is a smooth loss function and ψ is a regularization function.
When one optimizes a matrix W in ℝ^p × r^ instead of
a vector w in ℝ^p^, we will write

|  |  |  |  |
| --- | --- | --- | --- |
| min; W ∈ ℝ^p × r^ | [g(W) | ▵; = | f(W) + λψ(W)]. |

Note that the software can possibly handle nonnegativity constraints.

We start by presenting the type of regularization implemented in the software

### 5.1  Regularization Functions

Our software can handle the following regularization functions ψ for vectors w in ℝ^p^:

- The Tikhonov regularization: ψ(w) =^▵^ 1/2||w||_2_^2^.
- The ℓ_1_-norm: ψ(w) =^▵^ ||w||_1_.
- The Elastic-Net: ψ(w) =^▵^ ||w||_1_+γ||w||_2_^2^.
- The Fused-Lasso: ψ(w) =^▵^ ||w||_1_+γ||w||_2_^2^+γ_2_∑_i=1_^p−1^|w_i+1_−w_i_|.
- The group Lasso: ψ(w) =^▵^ ∑_g ∈ G_ η_g_ ||w_g_||_2_, where G are groups of variables.
- The group Lasso with ℓ_∞_-norm: ψ(w) =^▵^ ∑_g ∈ G_ η_g_ ||w_g_||_∞_, where G are groups of variables.
- The sparse group Lasso: same as above but with an additional ℓ_1_ term.
- The tree-structured sum of ℓ_2_-norms: ψ(w) =^▵^ ∑_g ∈ G_ η_g_ ||w_g_||_2_, where G is a tree-structured set of groups [15], and the η_g_ are positive weights.
- The tree-structured sum of ℓ_∞_-norms: ψ(w) =^▵^ ∑_g ∈ G_ η_g_ ||w_g_||_∞_. See [15]
- General sum of ℓ_∞_-norms: ψ(w) =^▵^ ∑_g ∈ G_ η_g_ ||w_g_||_∞_, where no assumption are made on the groups G.
- The path-coding penalties of [24].
- the ℓ_1_-constraint.
- a few undocumented ones.

Our software also handles regularization functions ψ on matrices W in ℝ^p × r^ (note that W can be transposed in these formulations). In particular,

- The ℓ_1_/ℓ_2_-norm: ψ(W) =^▵^ ∑_i=1_^p^ ||W_i_||_2_, where W_i_ denotes the i-th row of W.
- The ℓ_1_/ℓ_∞_-norm: ψ(W) =^▵^ ∑_i=1_^p^ ||W_i_||_∞_,
- The ℓ_1_/ℓ_2_+ℓ_1_-norm: ψ(W) =^▵^ ∑_i=1_^p^ ||W_i_||_2_ + λ_2_ ∑_i,j_|W_ij_|.
- The ℓ_1_/ℓ_∞_+ℓ_1_-norm: ψ(W) =^▵^ ∑_i=1_^p^ ||W_i_||_∞_+λ_2_ ∑_i,j_|W_ij_|,
- The ℓ_1_/ℓ_∞_-norm on rows and columns: ψ(W) =^▵^ ∑_i=1_^p^ ||W_i_||_∞_+λ_2_ ∑_j=1_^r^||W^j^||_∞_, where W^j^ denotes the j-th column of W.
- The multi-task tree-structured sum of ℓ_∞_-norms:

  |  |  |  |  |  |  |  |  |  |  |  |
  | --- | --- | --- | --- | --- | --- | --- | --- | --- | --- | --- |
  | ψ(W) | ▵; = |  | r; ∑; i=1 |  | ∑; g ∈ G | η_g_\|\|w_g_^i^\|\|_∞_+ γ | ∑; g ∈ G | η_g_ | max; j ∈ g | \|\|W_j_\|\|_∞_,     (25) |

  where the first double sums is in fact a sum of independent structured norms on the columns w^i^ of W, and the right term is a tree-structured regularization norm applied to the ℓ_∞_-norm of the rows of W, thereby inducing the tree-structured regularization at the row level. G is here a tree-structured set of groups.
- The multi-task general sum of ℓ_∞_-norms is the same as Eq. (25) except that the groups G are general overlapping groups.
- The trace norm: ψ(W) =^▵^ ||W||_\*_.

Non-convex regularizations are also implemented with the ISTA algorithm (no duality gaps are of course provided in these cases):

- The ℓ_0_-pseudo-norm: ψ(w) =^▵^ ||w||_0_.
- The rank: ψ(W) =^▵^ randk(W).
- The tree-structured ℓ_0_-pseudo-norm: ψ(w) =^▵^ ∑_g ∈ G_ δ_wg ≠ 0_.

All of these regularization terms for vectors or matrices can be coupled with
nonnegativity constraints. It is also possible to add an intercept, which one
wishes not to regularize, and we will include this possibility in the next
sections. There are also a few hidden undocumented options which are available in the source code.

We now present 3 functions for computing proximal operators associated to the previous regularization functions.

### 5.2  Function mexProximalFlat

This function computes the proximal operators associated to many regularization functions, for input signals U=[u^1^,…,u^n^] in ℝ^p × n^, it finds a matrix V=[v^1^,…,v^n^] in ℝ^p × n^ such that:

•  If one chooses a regularization function on vectors, for every column u of U, it computes one column v of V solving

min

v ∈ ℝ^p^

1

2

||u−v||_2_^2^ + λ ||v||_0_,
    (26)

or

min

v ∈ ℝ^p^

1

2

||u−v||_2_^2^ + λ ||v||_1_,
    (27)

or

|  |  |  |  |  |  |  |
| --- | --- | --- | --- | --- | --- | --- |
|  | min; v ∈ ℝ^p^ |  | 1; 2 | \|\|u−v\|\|_2_^2^ + | λ; 2 | \|\|v\|\|_2_^2^,     (28) |

or

min

v ∈ ℝ^p^

1

2

||u−v||_2_^2^ + λ ||v||_1_ + λ_2_||v||_2_^2^,
    (29)

or

|  |  |  |  |  |  |  |
| --- | --- | --- | --- | --- | --- | --- |
|  | min; v ∈ ℝ^p^ |  | 1; 2 | \|\|u−v\|\|_2_^2^ + λ | p−1; ∑; j=1 | \|v_j+1_^i^−v_j_^i^\|+λ_2_ \|\|v\|\|_1_ + λ_3_\|\|v\|\|_2_^2^,     (30) |

or

|  |  |  |  |  |  |  |
| --- | --- | --- | --- | --- | --- | --- |
|  | min; v ∈ ℝ^p^ |  | 1; 2 | \|\|u−v\|\|_2_^2^ + λ | ∑; g ∈ T | δ^g^(v),     (31) |

where T is a tree-structured set of groups (see [16]), and δ^g^(v) = 0 if v_g_=0 and 1 otherwise.
It can also solve

|  |  |  |  |  |  |  |
| --- | --- | --- | --- | --- | --- | --- |
|  | min; v ∈ ℝ^p^ |  | 1; 2 | \|\|u−v\|\|_2_^2^ + λ | ∑; g ∈ T | η^g^ \|\|v_g_\|\|_2_,     (32) |

or

|  |  |  |  |  |  |  |
| --- | --- | --- | --- | --- | --- | --- |
|  | min; v ∈ ℝ^p^ |  | 1; 2 | \|\|u−v\|\|_2_^2^ + λ | ∑; g ∈ T | η^g^ \|\|v_g_\|\|_∞_,     (33) |

or

|  |  |  |  |  |  |  |
| --- | --- | --- | --- | --- | --- | --- |
|  | min; v ∈ ℝ^p^ |  | 1; 2 | \|\|u−v\|\|_2_^2^ + λ | ∑; g ∈ G | η^g^ \|\|v_g_\|\|_∞_,     (34) |

where G is any kind of set of groups.

This function can also solve the following proximal operators on matrices

|  |  |  |  |  |  |  |
| --- | --- | --- | --- | --- | --- | --- |
|  | min; V ∈ ℝ^p × n^ |  | 1; 2 | \|\|U−V\|\|_F_^2^ + λ | p; ∑; i=1 | \|\|V_i_\|\|_2_,      (35) |

where V_i_ is the i-th row of V, or

|  |  |  |  |  |  |  |
| --- | --- | --- | --- | --- | --- | --- |
|  | min; V ∈ ℝ^p × n^ |  | 1; 2 | \|\|U−V\|\|_F_^2^ + λ | p; ∑; i=1 | \|\|V_i_\|\|_∞_,      (36) |

or

|  |  |  |  |  |  |  |  |  |  |
| --- | --- | --- | --- | --- | --- | --- | --- | --- | --- |
|  | min; V ∈ ℝ^p × n^ |  | 1; 2 | \|\|U−V\|\|_F_^2^ + λ | p; ∑; i=1 | \|\|V_i_\|\|_2_ +λ_2_ | p; ∑; i=1 | n; ∑; j=1 | \|V_ij_\|,      (37) |

or

|  |  |  |  |  |  |  |  |  |  |
| --- | --- | --- | --- | --- | --- | --- | --- | --- | --- |
|  | min; V ∈ ℝ^p × n^ |  | 1; 2 | \|\|U−V\|\|_F_^2^ + λ | p; ∑; i=1 | \|\|V_i_\|\|_∞_+λ_2_ | p; ∑; i=1 | n; ∑; j=1 | \|V_ij_\|,      (38) |

or

|  |  |  |  |  |  |  |  |  |
| --- | --- | --- | --- | --- | --- | --- | --- | --- |
|  | min; V ∈ ℝ^p × n^ |  | 1; 2 | \|\|U−V\|\|_F_^2^ + λ | p; ∑; i=1 | \|\|V_i_\|\|_∞_+λ_2_ | n; ∑; j=1 | \|\|V^j^\|\|_∞_.     (39) |

where V^j^ is the j-th column of V.

See details below:

%   
% Usage:  [V [val\_regularizer]]=mexProximalFlat(U,param);  
%  
% Name: mexProximalFlat  
%  
% Description: mexProximalFlat computes proximal operators. Depending  
%         on the value of param.regul, it computes   
%  
%         Given an input matrix U=[u^1,\ldots,u^n], it computes a matrix   
%         V=[v^1,\ldots,v^n] such that  
%         if one chooses a regularization functions on vectors, it computes  
%         for each column u of U, a column v of V solving  
%         if param.regul='l0'  
%             argmin 0.5||u-v||\_2^2 + lambda||v||\_0  
%         if param.regul='l1'  
%             argmin 0.5||u-v||\_2^2 + lambda||v||\_1  
%         if param.regul='l2'  
%             argmin 0.5||u-v||\_2^2 + 0.5lambda||v||\_2^2  
%         if param.regul='elastic-net'  
%             argmin 0.5||u-v||\_2^2 + lambda||v||\_1 + lambda\_2||v||\_2^2  
%         if param.regul='fused-lasso'  
%             argmin 0.5||u-v||\_2^2 + lambda FL(v) + ...  
%                               ...  lambda\_2||v||\_1 + lambda\_3||v||\_2^2  
%         if param.regul='linf'  
%             argmin 0.5||u-v||\_2^2 + lambda||v||\_inf  
%         if param.regul='l1-constraint'  
%             argmin 0.5||u-v||\_2^2 s.t. ||v||\_1 <= lambda  
%         if param.regul='l2-not-squared'  
%             argmin 0.5||u-v||\_2^2 + lambda||v||\_2  
%         if param.regul='group-lasso-l2'    
%             argmin 0.5||u-v||\_2^2 + lambda sum\_g ||v\_g||\_2   
%             where the groups are either defined by param.groups or by param.size\_group,  
%         if param.regul='group-lasso-linf'  
%             argmin 0.5||u-v||\_2^2 + lambda sum\_g ||v\_g||\_inf  
%         if param.regul='sparse-group-lasso-l2'    
%             argmin 0.5||u-v||\_2^2 + lambda sum\_g ||v\_g||\_2 + lambda\_2 ||v||\_1  
%             where the groups are either defined by param.groups or by param.size\_group,  
%         if param.regul='sparse-group-lasso-linf'  
%             argmin 0.5||u-v||\_2^2 + lambda sum\_g ||v\_g||\_inf + lambda\_2 ||v||\_1  
%         if param.regul='trace-norm-vec'   
%             argmin 0.5||u-v||\_2^2 + lambda ||mat(v)||\_\*   
%            where mat(v) has param.size\_group rows  
%  
%         if one chooses a regularization function on matrices  
%         if param.regul='l1l2',  V=   
%             argmin 0.5||U-V||\_F^2 + lambda||V||\_{1/2}  
%         if param.regul='l1linf',  V=   
%             argmin 0.5||U-V||\_F^2 + lambda||V||\_{1/inf}  
%         if param.regul='l1l2+l1',  V=   
%             argmin 0.5||U-V||\_F^2 + lambda||V||\_{1/2} + lambda\_2||V||\_{1/1}  
%         if param.regul='l1linf+l1',  V=   
%             argmin 0.5||U-V||\_F^2 + lambda||V||\_{1/inf} + lambda\_2||V||\_{1/1}  
%         if param.regul='l1linf+row-column',  V=   
%             argmin 0.5||U-V||\_F^2 + lambda||V||\_{1/inf} + lambda\_2||V'||\_{1/inf}  
%         if param.regul='trace-norm',  V=   
%             argmin 0.5||U-V||\_F^2 + lambda||V||\_\*  
%         if param.regul='rank',  V=   
%             argmin 0.5||U-V||\_F^2 + lambda rank(V)  
%         if param.regul='none',  V=   
%             argmin 0.5||U-V||\_F^2   
%           
%         for all these regularizations, it is possible to enforce non-negativity constraints  
%         with the option param.pos, and to prevent the last row of U to be regularized, with  
%         the option param.intercept  
%  
% Inputs: U:  double m x n matrix   (input signals)  
%               m is the signal size  
%         param: struct  
%               param.lambda  (regularization parameter)  
%               param.regul (choice of regularization, see above)  
%               param.lambda2  (optional, regularization parameter)  
%               param.lambda3  (optional, regularization parameter)  
%               param.verbose (optional, verbosity level, false by default)  
%               param.intercept (optional, last row of U is not regularized,  
%                 false by default)  
%               param.transpose (optional, transpose the matrix in the regularization function)  
%               param.size\_group (optional, for regularization functions assuming a group  
%                 structure). It is a scalar. When param.groups is not specified, it assumes  
%                 that the groups are the sets of consecutive elements of size param.size\_group  
%               param.groups (int32, optional, for regularization functions assuming a group  
%                 structure. It is an int32 vector of size m containing the group indices of the  
%                 variables (first group is 1).  
%               param.pos (optional, adds positivity constraints on the  
%                 coefficients, false by default)  
%               param.numThreads (optional, number of threads for exploiting  
%                 multi-core / multi-cpus. By default, it takes the value -1,  
%                 which automatically selects all the available CPUs/cores).  
%  
% Output: V: double m x n matrix (output coefficients)  
%         val\_regularizer: double 1 x n vector (value of the regularization  
%         term at the optimum).  
%  
% Author: Julien Mairal, 2010

The following piece of code illustrates how to use this function.

U=randn(100,1000);  
  
param.lambda=0.1; % regularization parameter  
param.num\_threads=-1; % all cores (-1 by default)  
param.verbose=true;   % verbosity, false by default  
  
% test l0  
fprintf('\nprox l0\n');  
param.regul='l0';  
param.pos=false;       % false by default  
param.intercept=false; % false by default  
alpha=mexProximalFlat(U,param);  
  
% test l1  
fprintf('\nprox l1, intercept, positivity constraint\n');  
param.regul='l1';  
param.pos=true;       % can be used with all the other regularizations  
param.intercept=true; % can be used with all the other regularizations       
alpha=mexProximalFlat(U,param);  
  
% test l2  
fprintf('\nprox squared-l2\n');  
param.regul='l2';  
param.pos=false;  
param.intercept=false;  
alpha=mexProximalFlat(U,param);  
  
% test elastic-net  
fprintf('\nprox elastic-net\n');  
param.regul='elastic-net';  
param.lambda2=0.1;  
alpha=mexProximalFlat(U,param);  
  
% test fused-lasso  
fprintf('\nprox fused lasso\n');  
param.regul='fused-lasso';  
param.lambda2=0.1;  
param.lambda3=0.1;  
alpha=mexProximalFlat(U,param);  
  
% test l1l2  
fprintf('\nprox mixed norm l1/l2\n');  
param.regul='l1l2';  
alpha=mexProximalFlat(U,param);  
  
% test l1linf  
fprintf('\nprox mixed norm l1/linf\n');  
param.regul='l1linf';  
alpha=mexProximalFlat(U,param);  
  
% test l1l2+l1  
fprintf('\nprox mixed norm l1/l2 + l1\n');  
param.regul='l1l2+l1';  
param.lambda2=0.1;  
alpha=mexProximalFlat(U,param);  
  
% test l1linf+l1  
fprintf('\nprox mixed norm l1/linf + l1\n');  
param.regul='l1linf+l1';  
param.lambda2=0.1;  
alpha=mexProximalFlat(U,param);  
  
% test l1linf-row-column  
fprintf('\nprox mixed norm l1/linf on rows and columns\n');  
param.regul='l1linf-row-column';  
param.lambda2=0.1;  
alpha=mexProximalFlat(U,param);  
  
% test none  
fprintf('\nprox no regularization\n');  
param.regul='none';  
alpha=mexProximalFlat(U,param);

### 5.3  Function mexProximalTree

This function computes the proximal operators associated to tree-structured regularization functions, for input signals U=[u^1^,…,u^n^] in ℝ^p × n^, and a tree-structured set of groups [15], it computes a matrix V=[v^1^,…,v^n^] in ℝ^p × n^. When one uses a regularization function on vectors, it computes a column v of V for every column u of U:

|  |  |  |  |  |  |  |
| --- | --- | --- | --- | --- | --- | --- |
|  | min; v ∈ ℝ^p^ |  | 1; 2 | \|\|u−v\|\|_2_^2^ + λ | ∑; g ∈ T | η^g^ \|\|v_g_\|\|_2_,     (40) |

or

|  |  |  |  |  |  |  |
| --- | --- | --- | --- | --- | --- | --- |
|  | min; v ∈ ℝ^p^ |  | 1; 2 | \|\|u−v\|\|_2_^2^ + λ | ∑; g ∈ T | η^g^ \|\|v_g_\|\|_∞_,     (41) |

or

|  |  |  |  |  |  |  |
| --- | --- | --- | --- | --- | --- | --- |
|  | min; v ∈ ℝ^p^ |  | 1; 2 | \|\|u−v\|\|_2_^2^ + λ | ∑; g ∈ T | δ^g^(v),     (42) |

where δ^g^(v)=0 if v_g_=0 and 1 otherwise (see appendix of [16]).

When the multi-task tree-structured regularization function is used, it solves

|  |  |  |  |  |  |  |  |  |  |  |  |
| --- | --- | --- | --- | --- | --- | --- | --- | --- | --- | --- | --- |
|  | min; V ∈ ℝ^p× n^ |  | 1; 2 | \|\|U−V\|\|_F_^2^ + λ | n; ∑; i=1 | ∑; g ∈ T | η^g^ \|\|v_g_^i^\|\|_∞_+ λ_2_ | ∑; g ∈ T |  | max; j ∈ g | \|\|v_g_^j^\|\|_∞_,     (43) |

which is a formulation presented in [22].

This function can also be used for computing the proximal operators addressed by mexProximalFlat (it will just not take into account the tree structure). The way the tree is incoded is presented below, (and examples are given in the file test\_ProximalTree.m, with more usage details:

%   
% Usage:  [V [val\_regularizer]]=mexProximalTree(U,tree,param);  
%  
% Name: mexProximalTree  
%  
% Description: mexProximalTree computes a proximal operator. Depending  
%         on the value of param.regul, it computes   
%  
%         Given an input matrix U=[u^1,\ldots,u^n], and a tree-structured set of groups T,  
%         it returns a matrix V=[v^1,\ldots,v^n]:  
%           
%         when the regularization function is for vectors,  
%         for every column u of U, it compute a column v of V solving  
%         if param.regul='tree-l0'  
%             argmin 0.5||u-v||\_2^2 + lambda \sum\_{g \in T} \delta^g(v)  
%         if param.regul='tree-l2'  
%           for all i, v^i =   
%             argmin 0.5||u-v||\_2^2 + lambda\sum\_{g \in T} \eta\_g||v\_g||\_2  
%         if param.regul='tree-linf'  
%           for all i, v^i =   
%             argmin 0.5||u-v||\_2^2 + lambda\sum\_{g \in T} \eta\_g||v\_g||\_inf  
%  
%         when the regularization function is for matrices:  
%         if param.regul='multi-task-tree'  
%            V=argmin 0.5||U-V||\_F^2 + lambda \sum\_{i=1}^n\sum\_{g \in T} \eta\_g||v^i\_g||\_inf + ...  
%                                                lambda\_2 \sum\_{g \in T} \eta\_g max\_{j in g}||V\_j||\_{inf}  
%           
%         it can also be used with any non-tree-structured regularization addressed by mexProximalFlat  
%  
%         for all these regularizations, it is possible to enforce non-negativity constraints  
%         with the option param.pos, and to prevent the last row of U to be regularized, with  
%         the option param.intercept  
%  
% Inputs: U:  double m x n matrix   (input signals)  
%               m is the signal size  
%         tree: struct  
%               with four fields, eta\_g, groups, own\_variables and N\_own\_variables.  
%  
%               The tree structure requires a particular organization of groups and variables  
%                  \* Let us denote by N = |T|, the number of groups.  
%                    the groups should be ordered T={g1,g2,\ldots,gN} such that if gi is included  
%                    in gj, then j <= i. g1 should be the group at the root of the tree   
%                    and contains every variable.  
%                  \* Every group is a set of  contiguous indices for instance   
%                    gi={3,4,5} or gi={4,5,6,7} or gi={4}, but not {3,5};  
%                  \* We define root(gi) as the indices of the variables that are in gi,  
%                    but not in its descendants. For instance for  
%                    T={ g1={1,2,3,4},g2={2,3},g3={4} }, then, root(g1)={1},   
%                    root(g2)={2,3}, root(g3)={4},  
%                    We assume that for all i, root(gi) is a set of contigous variables  
%                  \* We assume that the smallest of root(gi) is also the smallest index of gi.  
%  
%                  For instance,   
%                    T={ g1={1,2,3,4},g2={2,3},g3={4} }, is a valid set of groups.  
%                    but we can not have  
%                    T={ g1={1,2,3,4},g2={1,2},g3={3} }, since root(g1)={4} and 4 is not the  
%                    smallest element in g1.  
%  
%               We do not lose generality with these assumptions since they can be fullfilled for any  
%               tree-structured set of groups after a permutation of variables and a correct ordering of the  
%               groups.  
%               see more examples in test\_ProximalTree.m of valid tree-structured sets of groups.  
%                 
%               The first fields sets the weights for every group  
%                  tree.eta\_g            double N vector   
%    
%               The next field sets inclusion relations between groups   
%               (but not between groups and variables):  
%                  tree.groups           sparse (double or boolean) N x N matrix    
%                  the (i,j) entry is non-zero if and only if i is different than j and   
%                  gi is included in gj.  
%                  the first column corresponds to the group at the root of the tree.  
%  
%               The next field define the smallest index of each group gi,   
%               which is also the smallest index of root(gi)  
%               tree.own\_variables    int32 N vector  
%  
%               The next field define for each group gi, the size of root(gi)  
%               tree.N\_own\_variables  int32 N vector   
%  
%               examples are given in test\_ProximalTree.m  
%  
%         param: struct  
%               param.lambda  (regularization parameter)  
%               param.regul (choice of regularization, see above)  
%               param.lambda2  (optional, regularization parameter)  
%               param.lambda3  (optional, regularization parameter)  
%               param.verbose (optional, verbosity level, false by default)  
%               param.intercept (optional, last row of U is not regularized,  
%                 false by default)  
%               param.pos (optional, adds positivity constraints on the  
%                 coefficients, false by default)  
%               param.transpose (optional, transpose the matrix in the regularization function)  
%               param.size\_group (optional, for regularization functions assuming a group  
%                 structure). It is a scalar. When param.groups is not specified, it assumes  
%                 that the groups are the sets of consecutive elements of size param.size\_group  
%               param.numThreads (optional, number of threads for exploiting  
%                 multi-core / multi-cpus. By default, it takes the value -1,  
%                 which automatically selects all the available CPUs/cores).  
%  
% Output: V: double m x n matrix (output coefficients)  
%         val\_regularizer: double 1 x n vector (value of the regularization  
%         term at the optimum).  
%  
%  
% Author: Julien Mairal, 2010

The following piece of code illustrates how to use this function.

U=randn(10,1000);  
  
param.lambda=0.1; % regularization parameter  
param.num\_threads=-1; % all cores (-1 by default)  
param.verbose=true;   % verbosity, false by default  
param.pos=false;       % can be used with all the other regularizations  
param.intercept=false; % can be used with all the other regularizations       
  
fprintf('First tree example\n');  
% Example 1 of tree structure  
% tree structured groups:  
% g1= {0 1 2 3 4 5 6 7 8 9}  
% g2= {2 3 4}  
% g3= {5 6 7 8 9}  
tree.own\_variables=int32([0 2 5]);   % pointer to the first variable of each group  
tree.N\_own\_variables=int32([2 3 5]); % number of "root" variables in each group  
                              % (variables that are in a group, but not in its descendants).  
                              % for instance root(g1)={0,1}, root(g2)={2 3 4}, root(g3)={5 6 7 8 9}  
tree.eta\_g=[1 1 1];           % weights for each group, they should be non-zero to use fenchel duality  
tree.groups=sparse([0 0 0; ...  
                    1 0 0; ...  
                    1 0 0]);    % first group should always be the root of the tree  
                                % non-zero entriees mean inclusion relation ship, here g2 is a children of g1,  
                                % g3 is a children of g1  
  
fprintf('\ntest prox tree-l0\n');  
param.regul='tree-l0';  
alpha=mexProximalTree(U,tree,param);  
  
fprintf('\ntest prox tree-l2\n');  
param.regul='tree-l2';  
alpha=mexProximalTree(U,tree,param);  
  
fprintf('\ntest prox tree-linf\n');  
param.regul='tree-linf';  
alpha=mexProximalTree(U,tree,param);  
  
fprintf('Second tree example\n');  
% Example 2 of tree structure  
% tree structured groups:  
% g1= {0 1 2 3 4 5 6 7 8 9}    root(g1) = { };  
% g2= {0 1 2 3 4 5}            root(g2) = {0 1 2};  
% g3= {3 4}                    root(g3) = {3 4};  
% g4= {5}                      root(g4) = {5};  
% g5= {6 7 8 9}                root(g5) = { };  
% g6= {6 7}                    root(g6) = {6 7};  
% g7= {8 9}                    root(g7) = {8};  
% g8 = {9}                     root(g8) = {9};  
tree.own\_variables=  int32([0 0 3 5 6 6 8 9]);   % pointer to the first variable of each group  
tree.N\_own\_variables=int32([0 3 2 1 0 2 1 1]); % number of "root" variables in each group  
tree.eta\_g=[1 1 1 2 2 2 2.5 2.5];  
tree.groups=sparse([0 0 0 0 0 0 0 0; ...  
                    1 0 0 0 0 0 0 0; ...  
                    0 1 0 0 0 0 0 0; ...  
                    0 1 0 0 0 0 0 0; ...  
                    1 0 0 0 0 0 0 0; ...  
                    0 0 0 0 1 0 0 0; ...  
                    0 0 0 0 1 0 0 0; ...  
                    0 0 0 0 0 0 1 0]);  % first group should always be the root of the tree  
  
fprintf('\ntest prox tree-l0\n');  
param.regul='tree-l0';  
alpha=mexProximalTree(U,tree,param);  
  
fprintf('\ntest prox tree-l2\n');  
param.regul='tree-l2';  
alpha=mexProximalTree(U,tree,param);  
  
fprintf('\ntest prox tree-linf\n');  
param.regul='tree-linf';  
alpha=mexProximalTree(U,tree,param);  
  
% mexProximalTree also works with non-tree-structured regularization functions  
fprintf('\nprox l1, intercept, positivity constraint\n');  
param.regul='l1';  
param.pos=true;       % can be used with all the other regularizations  
param.intercept=true; % can be used with all the other regularizations       
alpha=mexProximalTree([U; ones(1,size(U,2))],tree,param);  
  
% Example of multi-task tree  
fprintf('\nprox multi-task tree\n');  
param.pos=false;  
param.intercept=false;  
param.lambda2=param.lambda;  
param.regul='multi-task-tree';  % with linf  
alpha=mexProximalTree(U,tree,param);  
  
  
tree.own\_variables=int32([0 1 2 3 4 5 6]);   % pointer to the first variable of each group  
tree.N\_own\_variables=int32([1 1 1 1 1 1]); % number of "root" variables in each group  
                              % (variables that are in a group, but not in its descendants).  
                              % for instance root(g1)={0,1}, root(g2)={2 3 4}, root(g3)={5 6 7 8 9}  
tree.eta\_g=[1 1 1 1 1 1];           % weights for each group, they should be non-zero to use fenchel duality  
tree.groups=sparse([0 0 0; ...  
                    1 0 0; ...  
                    1 0 0]);    % first group should always be the root of the tree  
                                % non-zero entriees mean inclusion relation ship, here g2 is a children of g1,  
                                % g3 is a children of g1

### 5.4  Function mexProximalGraph

This function computes the proximal operators associated to structured sparse regularization, for input signals U=[u^1^,…,u^n^] in ℝ^p × n^, and a set of groups [22], it returns a matrix V=[v^1^,…,v^n^] in ℝ^p × n^.
When one uses a regularization function on vectors, it computes a column v of V for every column u of U:

|  |  |  |  |  |  |  |
| --- | --- | --- | --- | --- | --- | --- |
|  | min; v ∈ ℝ^p^ |  | 1; 2 | \|\|u−v\|\|_2_^2^ + λ | ∑; g ∈ G | η^g^ \|\|v_g_\|\|_∞_,     (44) |

or with a regularization function on matrices, it computes V solving

|  |  |  |  |  |  |  |  |  |  |  |  |
| --- | --- | --- | --- | --- | --- | --- | --- | --- | --- | --- | --- |
|  | min; V ∈ ℝ^p× n^ |  | 1; 2 | \|\|U−V\|\|_F_^2^ + λ | n; ∑; i=1 | ∑; g ∈ G | η^g^ \|\|v_g_^i^\|\|_∞_+ λ_2_ | ∑; g ∈ G |  | max; j ∈ g | \|\|v_g_^j^\|\|_∞_,     (45) |

This function can also be used for computing the proximal operators addressed by mexProximalFlat. The way the graph is incoded is presented below (and also in the example file test\_ProximalGraph.m, with more usage details:

%   
% Usage:   [V [val\_regularizer]]=mexProximalGraph(U,graph,param);  
%  
% Name: mexProximalGraph  
%  
% Description: mexProximalGraph computes a proximal operator. Depending  
%         on the value of param.regul, it computes   
%  
%         Given an input matrix U=[u^1,\ldots,u^n], and a set of groups G,  
%         it computes a matrix V=[v^1,\ldots,v^n] such that  
%  
%         if param.regul='graph'  
%         for every column u of U, it computes a column v of V solving  
%             argmin 0.5||u-v||\_2^2 + lambda\sum\_{g \in G} \eta\_g||v\_g||\_inf  
%  
%         if param.regul='graph+ridge'  
%         for every column u of U, it computes a column v of V solving  
%             argmin 0.5||u-v||\_2^2 + lambda\sum\_{g \in G} \eta\_g||v\_g||\_inf + lambda\_2||v||\_2^2  
%  
%  
%         if param.regul='multi-task-graph'  
%            V=argmin 0.5||U-V||\_F^2 + lambda \sum\_{i=1}^n\sum\_{g \in G} \eta\_g||v^i\_g||\_inf + ...  
%                                                lambda\_2 \sum\_{g \in G} \eta\_g max\_{j in g}||V\_j||\_{inf}  
%           
%         it can also be used with any regularization addressed by mexProximalFlat  
%  
%         for all these regularizations, it is possible to enforce non-negativity constraints  
%         with the option param.pos, and to prevent the last row of U to be regularized, with  
%         the option param.intercept  
%  
% Inputs: U:  double p x n matrix   (input signals)  
%               m is the signal size  
%         graph: struct  
%               with three fields, eta\_g, groups, and groups\_var  
%  
%               The first fields sets the weights for every group  
%                  graph.eta\_g            double N vector   
%    
%               The next field sets inclusion relations between groups   
%               (but not between groups and variables):  
%                  graph.groups           sparse (double or boolean) N x N matrix    
%                  the (i,j) entry is non-zero if and only if i is different than j and   
%                  gi is included in gj.  
%                 
%               The next field sets inclusion relations between groups and variables  
%                  graph.groups\_var       sparse (double or boolean) p x N matrix  
%                  the (i,j) entry is non-zero if and only if the variable i is included   
%                  in gj, but not in any children of gj.  
%  
%               examples are given in test\_ProximalGraph.m  
%  
%         param: struct  
%               param.lambda  (regularization parameter)  
%               param.regul (choice of regularization, see above)  
%               param.lambda2  (optional, regularization parameter)  
%               param.lambda3  (optional, regularization parameter)  
%               param.verbose (optional, verbosity level, false by default)  
%               param.intercept (optional, last row of U is not regularized,  
%                 false by default)  
%               param.pos (optional, adds positivity constraints on the  
%                 coefficients, false by default)  
%               param.numThreads (optional, number of threads for exploiting  
%                 multi-core / multi-cpus. By default, it takes the value -1,  
%                 which automatically selects all the available CPUs/cores).  
%  
% Output: V: double p x n matrix (output coefficients)  
%         val\_regularizer: double 1 x n vector (value of the regularization  
%         term at the optimum).  
%  
% Author: Julien Mairal, 2010

The following piece of code illustrates how to use this function.

U=randn(10,1000);  
  
param.lambda=0.1; % regularization parameter  
param.num\_threads=-1; % all cores (-1 by default)  
param.verbose=true;   % verbosity, false by default  
param.pos=false;       % can be used with all the other regularizations  
param.intercept=false; % can be used with all the other regularizations       
  
fprintf('First graph example\n');  
% Example 1 of graph structure  
% groups:  
% g1= {0 1 2 3}  
% g2= {3 4 5 6}  
% g3= {6 7 8 9}  
graph.eta\_g=[1 1 1];  
graph.groups=sparse(zeros(3));  
graph.groups\_var=sparse([1 0 0;  
                         1 0 0;  
                         1 0 0;  
                         1 1 0;  
                         0 1 0;  
                         0 1 0;  
                         0 1 1;  
                         0 0 1;  
                         0 0 1;  
                         0 0 1]);  
  
fprintf('\ntest prox graph\n');  
param.regul='graph';  
alpha=mexProximalGraph(U,graph,param);  
  
% Example 2 of graph structure  
% groups:  
% g1= {0 1 2 3}  
% g2= {3 4 5 6}  
% g3= {6 7 8 9}  
% g4= {0 1 2 3 4 5}  
% g5= {6 7 8}  
graph.eta\_g=[1 1 1 1 1];  
graph.groups=sparse([0 0 0 1 0;  
                     0 0 0 0 0;  
                     0 0 0 0 0;  
                     0 0 0 0 0;  
                     0 0 1 0 0]);   % g5 is included in g3, and g2 is included in g4  
graph.groups\_var=sparse([1 0 0 0 0;  
                         1 0 0 0 0;  
                         1 0 0 0 0 ;  
                         1 1 0 0 0;  
                         0 1 0 1 0;  
                         0 1 0 1 0;  
                         0 1 0 0 1;  
                         0 0 0 0 1;  
                         0 0 0 0 1;  
                         0 0 1 0 0]); % represents direct inclusion relations   
                                      % between groups (columns) and variables (rows)  
  
fprintf('\ntest prox graph\n');  
param.regul='graph';  
alpha=mexProximalGraph(U,graph,param);  
  
fprintf('\ntest prox multi-task-graph\n');  
param.regul='multi-task-graph';  
param.lambda2=0.1;  
alpha=mexProximalGraph(U,graph,param);  
  
fprintf('\ntest no regularization\n');  
param.regul='none';  
alpha=mexProximalGraph(U,graph,param);

### 5.5  Function mexProximalPathCoding

This function computes the proximal operators associated to the path coding penalties of [24].

%   
% Usage:   [V [val\_regularizer]]=mexProximalPathCoding(U,DAG,param);  
%  
% Name: mexProximalPathCoding  
%  
% Description: mexProximalPathCoding computes a proximal operator for  
%         the path coding penalties of http://arxiv.org/abs/1204.4539  
%  
%         Given an input matrix U=[u^1,\ldots,u^n],   
%  
%  
% Inputs: U:  double p x n matrix   (input signals)  
%               m is the signal size  
%         DAG:  struct  
%               with three fields, weights, start\_weights, stop\_weights  
%         for a graph with |V| nodes and |E| arcs,  
%         DAG.weights: sparse double |V| x |V| matrix. Adjacency  
%               matrix. The non-zero entries represent costs on arcs  
%               linking two nodes.  
%         DAG.start\_weights: dense double |V| vector. Represent the costs  
%               of starting a path from a specific node.  
%         DAG.stop\_weights: dense double |V| vector. Represent the costs  
%               of ending a path at a specific node.  
%  
%         if param.regul='graph-path-l0', non-convex penalty  
%         if param.regul='graph-path-conv', convex penalty  
%  
%         param: struct  
%               param.lambda  (regularization parameter)  
%               param.regul (choice of regularization, see above)  
%               param.verbose (optional, verbosity level, false by default)  
%               param.intercept (optional, last row of U is not regularized,  
%                 false by default)  
%               param.pos (optional, adds positivity constraints on the  
%                 coefficients, false by default)  
%               param.precision (optional, by default a very large integer.  
%                 It returns approximate proximal operator by choosing a small integer,  
%                 for example, 100 or 1000.  
%               param.numThreads (optional, number of threads for exploiting  
%                 multi-core / multi-cpus. By default, it takes the value -1,  
%                 which automatically selects all the available CPUs/cores).  
%  
% Output: V: double p x n matrix (output coefficients)  
%         val\_regularizer: double 1 x n vector (value of the regularization  
%         term at the optimum).  
%  
% Author: Julien Mairal, 2012

The following piece of code illustrates how to use this function.

clear all;  
rand('seed',0);  
randn('seed',0);  
fprintf('test mexProximalPathCoding\n');  
p=100;  
% generate a DAG  
G=sprand(p,p,0.02);  
G=mexRemoveCyclesGraph(G);  
fprintf('\n');  
  
% generate a data matrix  
U=randn(p,10);  
U=U-mean(U(:));  
U=mexNormalize(U);  
  
% input graph  
graph.weights=G;  
graph.stop\_weights=zeros(1,p);  
graph.start\_weights=10\*ones(1,p);  
  
% FISTA parameters  
param.num\_threads=-1; % all cores (-1 by default)  
param.verbose=true;   % verbosity, false by default  
param.lambda=0.05; % regularization parameter  
  
fprintf('Proximal convex path penalty\n');  
param.regul='graph-path-conv';  
tic  
[V1 optim]=mexProximalPathCoding(U,graph,param);  
t=toc;  
num=mexCountConnexComponents(graph.weights,V1(:,1));  
fprintf('Num of connected components: %d\n',num);  
  
fprintf('Proximal non-convex path penalty\n');  
param.regul='graph-path-l0';  
param.lambda=0.005;  
tic  
[V2 optim]=mexProximalPathCoding(U,graph,param);  
t=toc;  
num=mexCountConnexComponents(graph.weights,V2(:,1));  
fprintf('Num of connected components: %d\n',num);  
  
graph.start\_weights=1\*ones(1,p);  
param.lambda=0.05;  
fprintf('Proximal convex path penalty\n');  
param.regul='graph-path-conv';  
tic  
[V1 optim]=mexProximalPathCoding(U,graph,param);  
t=toc;  
num=mexCountConnexComponents(graph.weights,V1(:,1));  
fprintf('Num of connected components: %d\n',num);  
  
fprintf('Proximal non-convex path penalty\n');  
param.regul='graph-path-l0';  
param.lambda=0.005;  
tic  
[V2 optim]=mexProximalPathCoding(U,graph,param);  
t=toc;  
num=mexCountConnexComponents(graph.weights,V2(:,1));  
fprintf('Num of connected components: %d\n',num);

This function is associated to a function to evaluate the penalties:

### 5.6  Function mexEvalPathCoding

%   
% Usage:   [val [paths]]=mexEvalPathCoding(U,DAG,param);  
%  
% Name: mexEvalPathCoding  
%  
% Description: mexEvalPathCoding evaluate the path coding penalies   
%         of http://arxiv.org/abs/1204.4539 and provides a path   
%         decomposition of a vector W.  
%  
%         Given an input matrix U=[u^1,\ldots,u^n],   
%  
%  
% Inputs: U:  double p x n matrix   (input signals)  
%               m is the signal size  
%         DAG:  struct  
%               with three fields, weights, start\_weights, stop\_weights  
%         for a graph with |V| nodes and |E| arcs,  
%         DAG.weights: sparse double |V| x |V| matrix. Adjacency  
%               matrix. The non-zero entries represent costs on arcs  
%               linking two nodes.  
%         DAG.start\_weights: dense double |V| vector. Represent the costs  
%               of starting a path from a specific node.  
%         DAG.stop\_weights: dense double |V| vector. Represent the costs  
%               of ending a path at a specific node.  
%  
%         if param.regul='graph-path-l0', non-convex penalty  
%         if param.regul='graph-path-conv', convex penalty  
%  
%         param: struct  
%               param.regul (choice of regularization, see above)  
%               param.verbose (optional, verbosity level, false by default)  
%               param.precision (optional, by default a very large integer.  
%                 It returns approximate proximal operator by choosing a small integer,  
%                 for example, 100 or 1000.  
%               param.numThreads (optional, number of threads for exploiting  
%                 multi-core / multi-cpus. By default, it takes the value -1,  
%                 which automatically selects all the available CPUs/cores).  
%  
% Output: V: double 1 x n vector (values of the objective function)  
%         paths: optional, double sparse p x k matrix. selected paths for the   
%                first column of U  
%  
% Author: Julien Mairal, 2012

The following piece of code illustrates how to use this function.

clear all;  
rand('seed',0);  
randn('seed',0);  
  
p=100;  
G=sprand(p,p,0.05);  
G=mexRemoveCyclesGraph(G);  
fprintf('\n');  
  
% input graph  
graph.weights=G;  
graph.stop\_weights=zeros(1,p);  
graph.start\_weights=10\*ones(1,p);  
  
param.regul='graph-path-l0';  
U=randn(p,10);  
U=U-mean(U(:));  
U=mexNormalize(U);  
param.lambda=0.005;  
[V2 optim]=mexProximalPathCoding(U,graph,param);  
[vals paths]=mexEvalPathCoding(U,graph,param);

After having presented the regularization terms which our software can handle,
we present the various formulations that we address

### 5.7  Problems Addressed

We present here regression or classification formulations and their multi-task variants.

#### 5.7.1  Regression Problems with the Square Loss

Given a training set {x^i^,y_i_}_i=1_^n^, with x^i^ ∈ ℝ^p^ and y_i_ ∈ ℝ for all i in [ 1;n ], we address

min

w ∈ ℝ^p^, b ∈ ℝ

n

∑

i=1

1

2

(y_i_−w^⊤^x^i^−b)^2^ + λψ(w),

where b is an optional variable acting as an “intercept”, which is not regularized, and ψ
can be any of the regularization functions presented above.
Let us consider the vector y in ℝ^n^ that carries the entries y_i_.
The problem without the intercept takes the following form, which we have already
encountered in the previous toolbox, but with different notations:

min

w ∈ ℝ^p^

1

2

||y−Xw||_2_^2^ + λψ(w),

where the X=[x^i^,…,x^n^]^T^ (the x^i^’s are here the rows of X).

#### 5.7.2  Classification Problems with the Logistic Loss

The next formulation that our software can solve is the regularized logistic regression formulation.
We are again given a training set {x^i^,y_i_}_i=1_^n^, with x^i^ ∈
ℝ^p^, but the variables y_i_ are now in {−1,+1} for all i in
[ 1;n ]. The optimization problem we address is

min

w ∈ ℝ^p^, b ∈ ℝ

1

n

n

∑

i=1

log(1+e^−y_i_(w⊤xi+b)^ + λψ(w),

with again ψ taken to be one of the regularization function presented above, and b is an optional intercept.

#### 5.7.3  Multi-class Classification Problems with the Softmax Loss

We have also implemented a multi-class logistic classifier (or softmax).
For a classification problem with r classes, we are given a training set {x^i^,y_i_}_i=1_^n^, where the variables x^i^ are still vectors in ℝ^p^, but the y_i_’s have integer values in {1,2,…,r}. The formulation we address is the following multi-class learning problem

|  |  |  |  |  |  |  |  |  |  |  |  |  |
| --- | --- | --- | --- | --- | --- | --- | --- | --- | --- | --- | --- | --- |
|  | min; W ∈ ℝ^p × r^, b ∈ ℝ^r^ |  | 1; n | n; ∑; i=1 | log | ⎛  ⎜  ⎝ | r; ∑; j=1 | e ^(wj−wy_i_)⊤xi + b_j_−b_yi_^ | ⎞  ⎟  ⎠ | + λ | r; ∑; j=1 | ψ(w^j^),     (46) |

where W = [w^1^,…,w^r^] and the optional vector b in ℝ^r^ carries intercepts for each class.

#### 5.7.4  Multi-task Regression Problems with the Square Loss

We are now considering a problem with r tasks, and a training set
{x^i^,y^i^}_i=1_^n^, where the variables x^i^ are still vectors in ℝ^p^, and y^i^
is a vector in ℝ^r^. We are looking for r regression vectors w^j^, for j∈ [ 1;r ], or equivalently for a matrix W=[w^1^,…,w^r^] in ℝ^p × r^. The formulation we address is the following
multi-task regression problem

min

W ∈ ℝ^p × r^, b ∈ ℝ^r^

r

∑

j=1

n

∑

i=1

1

2

(y_j_^i^−w^⊤^x^i^−b_j_)^2^ + λψ(W),

where ψ is any of the regularization function on matrices we have presented in the previous section.
Note that by introducing the appropriate variables Y, the problem without intercept could be equivalently rewritten

min

W ∈ ℝ^p × r^

1

2

||Y−XW||_F_^2^ + λψ(W).

#### 5.7.5  Multi-task Classification Problems with the Logistic Loss

The multi-task version of the logistic regression follows the same principle.
We consider r tasks, and a training set
{x^i^,y^i^}_i=1_^n^, with the x^i^’s in ℝ^p^, and the y^i^’s
are vectors in {−1,+1}^r^. We look for a matrix W=[w^1^,…,w^r^] in ℝ^p × r^. The formulation is the following
multi-task regression problem

|  |  |  |  |  |  |  |  |  |  |  |
| --- | --- | --- | --- | --- | --- | --- | --- | --- | --- | --- |
|  | min; W ∈ ℝ^p × r^, b ∈ ℝ^r^ |  | r; ∑; j=1 | 1; n | n; ∑; i=1 | log | ⎛  ⎜  ⎝ | 1+e^−y_j_i(w⊤xi+b_j_)^ | ⎞  ⎟  ⎠ | + λψ(W). |

#### 5.7.6  Multi-task and Multi-class Classification Problems with the Softmax Loss

The multi-task/multi-class version directly follows from the formulation of Eq. (46), but associates with each class a task, and as a consequence, regularizes the matrix W in a particular way:

|  |  |  |  |  |  |  |  |  |  |  |
| --- | --- | --- | --- | --- | --- | --- | --- | --- | --- | --- |
|  | min; W ∈ ℝ^p × r^, b ∈ ℝ^r^ |  | 1; n | n; ∑; i=1 | log | ⎛  ⎜  ⎝ | r; ∑; j=1 | e ^(wj−wy_i_)⊤xi + b_j_−b_yi_^ | ⎞  ⎟  ⎠ | + λψ(W). |

How duality gaps are computed for any of these formulations is presented in Appendix A.
We now present the main functions for solving these problems

### 5.8  Function mexFistaFlat

Given a matrix X=[x^1^,…,x^p^]^T^ in ℝ^m × p^, and a matrix Y=[y^1^,…,y^n^], it solves the optimization problems presented in the previous section, with the same regularization functions as mexProximalFlat.
see usage details below:

%   
% Usage: [W [optim]]=mexFistaFlat(Y,X,W0,param);  
%  
% Name: mexFistaFlat  
%  
% Description: mexFistaFlat solves sparse regularized problems.  
%         X is a design matrix of size m x p  
%         X=[x^1,...,x^n]', where the x\_i's are the rows of X  
%         Y=[y^1,...,y^n] is a matrix of size m x n  
%         It implements the algorithms FISTA, ISTA and subgradient descent.  
%           
%           - if param.loss='square' and param.regul is a regularization function for vectors,  
%             the entries of Y are real-valued,  W = [w^1,...,w^n] is a matrix of size p x n  
%             For all column y of Y, it computes a column w of W such that  
%               w = argmin 0.5||y- X w||\_2^2 + lambda psi(w)  
%  
%           - if param.loss='square' and param.regul is a regularization function for matrices  
%             the entries of Y are real-valued,  W is a matrix of size p x n.   
%             It computes the matrix W such that  
%               W = argmin 0.5||Y- X W||\_F^2 + lambda psi(W)  
%              
%           - param.loss='square-missing' : same as param.loss='square', but handles missing data  
%             represented by NaN (not a number) in the matrix Y  
%  
%           - if param.loss='logistic' and param.regul is a regularization function for vectors,  
%             the entries of Y are either -1 or +1, W = [w^1,...,w^n] is a matrix of size p x n  
%             For all column y of Y, it computes a column w of W such that  
%               w = argmin (1/m)sum\_{j=1}^m log(1+e^(-y\_j x^j' w)) + lambda psi(w),  
%             where x^j is the j-th row of X.  
%  
%           - if param.loss='logistic' and param.regul is a regularization function for matrices  
%             the entries of Y are either -1 or +1, W is a matrix of size p x n  
%               W = argmin sum\_{i=1}^n(1/m)sum\_{j=1}^m log(1+e^(-y^i\_j x^j' w^i)) + lambda psi(W)  
%  
%           - if param.loss='multi-logistic' and param.regul is a regularization function for vectors,  
%             the entries of Y are in {0,1,...,N} where N is the total number of classes  
%             W = [W^1,...,W^n] is a matrix of size p x Nn, each submatrix W^i is of size p x N  
%             for all submatrix WW of W, and column y of Y, it computes  
%               WW = argmin (1/m)sum\_{j=1}^m log(sum\_{j=1}^r e^(x^j'(ww^j-ww^{y\_j}))) + lambda sum\_{j=1}^N psi(ww^j),  
%             where ww^j is the j-th column of WW.  
%  
%           - if param.loss='multi-logistic' and param.regul is a regularization function for matrices,  
%             the entries of Y are in {0,1,...,N} where N is the total number of classes  
%             W is a matrix of size p x N, it computes  
%               W = argmin (1/m)sum\_{j=1}^m log(sum\_{j=1}^r e^(x^j'(w^j-w^{y\_j}))) + lambda psi(W)  
%             where ww^j is the j-th column of WW.  
%  
%           - param.loss='cur' : useful to perform sparse CUR matrix decompositions,   
%               W = argmin 0.5||Y-X\*W\*X||\_F^2 + lambda psi(W)  
%  
%  
%         The function psi are those used by mexProximalFlat (see documentation)  
%  
%         This function can also handle intercepts (last row of W is not regularized),  
%         and/or non-negativity constraints on W, and sparse matrices for X  
%  
% Inputs: Y:  double dense m x n matrix  
%         X:  double dense or sparse m x p matrix     
%         W0:  double dense p x n matrix or p x Nn matrix (for multi-logistic loss)  
%              initial guess  
%         param: struct  
%            param.loss (choice of loss, see above)  
%            param.regul (choice of regularization, see function mexProximalFlat)  
%            param.lambda (regularization parameter)  
%            param.lambda2 (optional, regularization parameter, 0 by default)  
%            param.lambda3 (optional, regularization parameter, 0 by default)  
%            param.verbose (optional, verbosity level, false by default)  
%            param.pos (optional, adds positivity constraints on the  
%                coefficients, false by default)  
%            param.transpose (optional, transpose the matrix in the regularization function)  
%            param.size\_group (optional, for regularization functions assuming a group  
%                 structure)  
%            param.groups (int32, optional, for regularization functions assuming a group  
%                 structure, see mexProximalFlat)  
%            param.numThreads (optional, number of threads for exploiting  
%                multi-core / multi-cpus. By default, it takes the value -1,  
%                which automatically selects all the available CPUs/cores).  
%            param.max\_it (optional, maximum number of iterations, 100 by default)  
%            param.it0 (optional, frequency for computing duality gap, every 10 iterations by default)  
%            param.tol (optional, tolerance for stopping criteration, which is a relative duality gap  
%                if it is available, or a relative change of parameters).  
%            param.gamma (optional, multiplier for increasing the parameter L in fista, 1.5 by default)  
%            param.L0 (optional, initial parameter L in fista, 0.1 by default, should be small enough)  
%            param.fixed\_step (deactive the line search for L in fista and use param.L0 instead)  
%            param.linesearch\_mode (line-search scheme when ista=true:  
%                      0: default, monotonic backtracking scheme  
%                      1: monotonic backtracking scheme, with restart at each iteration  
%                      2: Barzilai-Borwein step sizes (similar to SparSA by Wright et al.)  
%                      3: non-monotonic backtracking  
%            param.compute\_gram (optional, pre-compute X^TX, false by default).  
%            param.intercept (optional, do not regularize last row of W, false by default).  
%            param.ista (optional, use ista instead of fista, false by default).  
%            param.subgrad (optional, if not param.ista, use subradient descent instead of fista, false by default).  
%            param.a, param.b (optional, if param.subgrad, the gradient step is a/(t+b)  
%            also similar options as mexProximalFlat  
%  
%            the function also implements the ADMM algorithm via an option param.admm=true. It is not documented  
%            and you need to look at the source code to use it.  
%  
% Output:  W:  double dense p x n matrix or p x Nn matrix (for multi-logistic loss)  
%          optim: optional, double dense 4 x n matrix.  
%              first row: values of the objective functions.  
%              third row: values of the relative duality gap (if available)  
%              fourth row: number of iterations  
%  
% Author: Julien Mairal, 2010

The following piece of code illustrates how to use this function.

format compact;  
randn('seed',0);  
param.numThreads=-1; % all cores (-1 by default)  
param.verbose=true;   % verbosity, false by default  
param.lambda=0.05; % regularization parameter  
param.it0=10;      % frequency for duality gap computations  
param.max\_it=200; % maximum number of iterations  
param.L0=0.1;  
param.tol=1e-3;  
param.intercept=false;  
param.pos=false;  
param.ista=false;  
  
X=randn(100,200);  
X=X-repmat(mean(X),[size(X,1) 1]);  
X=mexNormalize(X);  
Y=randn(100,1);  
Y=Y-repmat(mean(Y),[size(Y,1) 1]);  
Y=mexNormalize(Y);  
W0=zeros(size(X,2),size(Y,2));  
% Regression experiments   
% 100 regression problems with the same design matrix X.  
fprintf('\nVarious regression experiments\n');  
param.compute\_gram=true;  
fprintf('\nFISTA + Regression l1\n');  
param.loss='square';  
param.regul='l1';  
% param.regul='group-lasso-l2';  
% param.size\_group=10;  
tic  
[W optim\_info]=mexFistaFlat(Y,X,W0,param);  
t=toc;  
fprintf('mean loss: %f, mean relative duality\_gap: %f, time: %f, number of iterations: %f\n',mean(optim\_info(1,:)),mean(optim\_info(3,:)),t,mean(optim\_info(4,:)));  
  
param.regul='l1';  
fprintf('\nISTA + Regression l1\n');  
param.ista=true;  
tic  
[W optim\_info]=mexFistaFlat(Y,X,W0,param);  
t=toc;  
fprintf('mean loss: %f, mean relative duality\_gap: %f, time: %f, number of iterations: %f\n',mean(optim\_info(1,:)),mean(optim\_info(3,:)),t,mean(optim\_info(4,:)));  
  
param.regul='l1';  
fprintf('\nISTA + Regression l1 + Barzilai Borwein (SPARSA)\n');  
param.ista=true;  
param.barzilaiborwein=true;  
tic  
[W optim\_info]=mexFistaFlat(Y,X,W0,param);  
t=toc;  
fprintf('mean loss: %f, mean relative duality\_gap: %f, time: %f, number of iterations: %f\n',mean(optim\_info(1,:)),mean(optim\_info(3,:)),t,mean(optim\_info(4,:)));  
param.barzilaiborwein=false;  
  
  
fprintf('\nSubgradient Descent + Regression l1\n');  
param.ista=false;  
param.subgrad=true;  
param.a=0.1;  
param.b=1000; % arbitrary parameters  
max\_it=param.max\_it;  
it0=param.it0;  
param.max\_it=500;  
param.it0=50;  
tic  
[W optim\_info]=mexFistaFlat(Y,X,W0,param);  
t=toc;  
param.subgrad=false;  
param.max\_it=max\_it;  
param.it0=it0;  
fprintf('mean loss: %f, mean relative duality\_gap: %f, time: %f, number of iterations: %f\n',mean(optim\_info(1,:)),mean(optim\_info(3,:)),t,mean(optim\_info(4,:)));  
  
fprintf('\nFISTA + Regression l2\n');  
param.regul='l2';  
tic  
[W optim\_info]=mexFistaFlat(Y,X,W0,param);  
t=toc;  
fprintf('mean loss: %f, mean relative duality\_gap: %f, time: %f, number of iterations: %f\n',mean(optim\_info(1,:)),mean(optim\_info(3,:)),t,mean(optim\_info(4,:)));  
  
fprintf('\nFISTA + Regression l2 + sparse feature matrix\n');  
param.regul='l2';  
tic  
[W optim\_info]=mexFistaFlat(Y,sparse(X),W0,param);  
t=toc;  
fprintf('mean loss: %f, mean relative duality\_gap: %f, time: %f, number of iterations: %f\n',mean(optim\_info(1,:)),mean(optim\_info(3,:)),t,mean(optim\_info(4,:)));  
  
  
fprintf('\nFISTA + Regression Elastic-Net\n');  
param.regul='elastic-net';  
param.lambda2=0.1;  
tic  
[W optim\_info]=mexFistaFlat(Y,X,W0,param);  
t=toc;  
fprintf('mean loss: %f, time: %f, number of iterations: %f\n',mean(optim\_info(1,:)),t,mean(optim\_info(4,:)));  
  
fprintf('\nFISTA + Group Lasso L2\n');  
param.regul='group-lasso-l2';  
param.size\_group=2;  % all the groups are of size 2  
tic  
[W optim\_info]=mexFistaFlat(Y,X,W0,param);  
t=toc;  
fprintf('mean loss: %f, mean relative duality\_gap: %f, time: %f, number of iterations: %f\n',mean(optim\_info(1,:)),mean(optim\_info(3,:)),t,mean(optim\_info(4,:)));  
  
fprintf('\nFISTA + Group Lasso L2 with variable size of groups \n');  
param.regul='group-lasso-l2';  
param2=param;  
param2.groups=int32(randi(5,1,size(X,2)));  % all the groups are of size 2  
param2.lambda=10\*param2.lambda;  
tic  
[W optim\_info]=mexFistaFlat(Y,X,W0,param2);  
t=toc;  
fprintf('mean loss: %f, mean relative duality\_gap: %f, time: %f, number of iterations: %f\n',mean(optim\_info(1,:)),mean(optim\_info(3,:)),t,mean(optim\_info(4,:)));  
  
fprintf('\nFISTA + Trace Norm\n');  
param.regul='trace-norm-vec';  
param.size\_group=5;  
tic  
[W optim\_info]=mexFistaFlat(Y,X,W0,param);  
t=toc;  
fprintf('mean loss: %f, mean relative duality\_gap: %f, time: %f, number of iterations: %f\n',mean(optim\_info(1,:)),mean(optim\_info(3,:)),t,mean(optim\_info(4,:)));  
  
fprintf('\nFISTA + Regression Fused-Lasso\n');  
param.regul='fused-lasso';  
param.lambda2=0.1;  
param.lambda3=0.1; %  
tic  
[W optim\_info]=mexFistaFlat(Y,X,W0,param);  
t=toc;  
fprintf('mean loss: %f, time: %f, number of iterations: %f\n',mean(optim\_info(1,:)),t,mean(optim\_info(4,:)));  
  
fprintf('\nFISTA + Regression no regularization\n');  
param.regul='none';  
tic  
[W optim\_info]=mexFistaFlat(Y,X,W0,param);  
t=toc;  
fprintf('mean loss: %f, time: %f, number of iterations: %f\n',mean(optim\_info(1,:)),t,mean(optim\_info(4,:)));  
  
fprintf('\nFISTA + Regression l1 with intercept \n');  
param.intercept=true;  
param.regul='l1';  
tic  
[W optim\_info]=mexFistaFlat(Y,[X ones(size(X,1),1)],[W0; zeros(1,size(W0,2))],param); % adds a column of ones to X for the intercept  
t=toc;  
fprintf('mean loss: %f, mean relative duality\_gap: %f, time: %f, number of iterations: %f\n',mean(optim\_info(1,:)),mean(optim\_info(3,:)),t,mean(optim\_info(4,:)));  
  
fprintf('\nFISTA + Regression l1 with intercept+ non-negative \n');  
param.pos=true;  
param.regul='l1';  
tic  
[W optim\_info]=mexFistaFlat(Y,[X ones(size(X,1),1)],[W0; zeros(1,size(W0,2))],param);  
t=toc;  
fprintf('mean loss: %f, time: %f, number of iterations: %f\n',mean(optim\_info(1,:)),t,mean(optim\_info(4,:)));  
param.pos=false;  
param.intercept=false;  
  
fprintf('\nISTA + Regression l0\n');  
param.regul='l0';  
tic  
[W optim\_info]=mexFistaFlat(Y,X,W0,param);  
t=toc;  
fprintf('mean loss: %f, time: %f, number of iterations: %f\n',mean(optim\_info(1,:)),t,mean(optim\_info(4,:)));  
  
fprintf('\nOne classification experiment\n');  
Y=2\*double(randn(100,1) > 0)-1;  
fprintf('\nFISTA + Logistic l1\n');  
param.regul='l1';  
param.loss='logistic';  
param.lambda=0.01;  
tic  
[W optim\_info]=mexFistaFlat(Y,X,W0,param);  
t=toc;  
fprintf('mean loss: %f, mean relative duality\_gap: %f, time: %f, number of iterations: %f\n',mean(optim\_info(1,:)),mean(optim\_info(3,:)),t,mean(optim\_info(4,:)));  
% can be used of course with other regularization functions, intercept,...  
param.regul='l1';  
param.loss='weighted-logistic';  
param.lambda=0.01;  
fprintf('\nFISTA + weighted Logistic l1 + sparse matrix\n');  
tic  
[W optim\_info]=mexFistaFlat(Y,X,W0,param);  
t=toc;  
% can be used of course with other regularization functions, intercept,...  
  
param.loss='logistic';  
fprintf('\nFISTA + Logistic l1 + sparse matrix\n');  
tic  
[W optim\_info]=mexFistaFlat(Y,sparse(X),W0,param);  
t=toc;  
fprintf('mean loss: %f, mean relative duality\_gap: %f, time: %f, number of iterations: %f\n',mean(optim\_info(1,:)),mean(optim\_info(3,:)),t,mean(optim\_info(4,:)));  
% can be used of course with other regularization functions, intercept,...  
  
% Multi-Class classification  
Y=double(ceil(5\*rand(100,1000))-1);  
param.loss='multi-logistic';  
fprintf('\nFISTA + Multi-Class Logistic l1\n');  
tic  
nclasses=max(Y(:))+1;  
W0=zeros(size(X,2),nclasses\*size(Y,2));  
[W optim\_info]=mexFistaFlat(Y,X,W0,param);  
t=toc;  
fprintf('mean loss: %f, mean relative duality\_gap: %f, time: %f, number of iterations: %f\n',mean(optim\_info(1,:)),mean(optim\_info(3,:)),t,mean(optim\_info(4,:)));  
pause  
% can be used of course with other regularization functions, intercept,...  
  
% Multi-Task regression  
Y=randn(100,100);  
Y=Y-repmat(mean(Y),[size(Y,1) 1]);  
Y=mexNormalize(Y);  
param.compute\_gram=false;  
W0=zeros(size(X,2),size(Y,2));  
param.loss='square';  
fprintf('\nFISTA + Regression l1l2 \n');  
param.regul='l1l2';  
tic  
[W optim\_info]=mexFistaFlat(Y,X,W0,param);  
toc  
fprintf('mean loss: %f, mean relative duality\_gap: %f, time: %f, number of iterations: %f\n',mean(optim\_info(1,:)),mean(optim\_info(3,:)),t,mean(optim\_info(4,:)));  
  
fprintf('\nFISTA + Regression l1linf \n');  
param.regul='l1linf';  
tic  
[W optim\_info]=mexFistaFlat(Y,X,W0,param);  
toc  
fprintf('mean loss: %f, mean relative duality\_gap: %f, time: %f, number of iterations: %f\n',mean(optim\_info(1,:)),mean(optim\_info(3,:)),t,mean(optim\_info(4,:)));  
  
fprintf('\nFISTA + Regression l1l2 + l1 \n');  
param.regul='l1l2+l1';  
param.lambda2=0.1;  
tic  
[W optim\_info]=mexFistaFlat(Y,X,W0,param);  
toc  
fprintf('mean loss: %f, time: %f, number of iterations: %f\n',mean(optim\_info(1,:)),t,mean(optim\_info(4,:)));  
  
fprintf('\nFISTA + Regression l1linf + l1 \n');  
param.regul='l1linf+l1';  
param.lambda2=0.1;  
tic  
[W optim\_info]=mexFistaFlat(Y,X,W0,param);  
toc  
fprintf('mean loss: %f, time: %f, number of iterations: %f\n',mean(optim\_info(1,:)),t,mean(optim\_info(4,:)));  
  
fprintf('\nFISTA + Regression l1linf + row + columns \n');  
param.regul='l1linf-row-column';  
param.lambda2=0.1;  
tic  
[W optim\_info]=mexFistaFlat(Y,X,W0,param);  
toc  
fprintf('mean loss: %f, mean relative duality\_gap: %f, time: %f, number of iterations: %f\n',mean(optim\_info(1,:)),mean(optim\_info(3,:)),t,mean(optim\_info(4,:)));  
  
% Multi-Task Classification  
fprintf('\nFISTA + Logistic + l1l2 \n');  
param.regul='l1l2';  
param.loss='logistic';  
Y=2\*double(randn(100,100) > 0)-1;  
tic  
[W optim\_info]=mexFistaFlat(Y,X,W0,param);  
toc  
fprintf('mean loss: %f, mean relative duality\_gap: %f, time: %f, number of iterations: %f\n',mean(optim\_info(1,:)),mean(optim\_info(3,:)),t,mean(optim\_info(4,:)));  
% Multi-Class + Multi-Task Regularization  
  
fprintf('\nFISTA + Multi-Class Logistic l1l2 \n');  
Y=double(ceil(5\*rand(100,1000))-1);  
param.loss='multi-logistic';  
param.regul='l1l2';  
tic  
nclasses=max(Y(:))+1;  
W0=zeros(size(X,2),nclasses\*size(Y,2));  
[W optim\_info]=mexFistaFlat(Y,X,W0,param);  
t=toc;  
fprintf('mean loss: %f, mean relative duality\_gap: %f, time: %f, number of iterations: %f\n',mean(optim\_info(1,:)),mean(optim\_info(3,:)),t,mean(optim\_info(4,:)));  
% can be used of course with other regularization functions, intercept,...

### 5.9  Function mexFistaTree

Given a matrix X=[x^1^,…,x^p^]^T^ in ℝ^m × p^, and a matrix Y=[y^1^,…,y^n^], it solves the optimization problems presented in the previous section, with the same regularization functions as mexProximalTree.
see usage details below:

%   
% Usage: [W [optim]]=mexFistaTree(Y,X,W0,tree,param);  
%  
% Name: mexFistaTree  
%  
% Description: mexFistaTree solves sparse regularized problems.  
%         X is a design matrix of size m x p  
%         X=[x^1,...,x^n]', where the x\_i's are the rows of X  
%         Y=[y^1,...,y^n] is a matrix of size m x n  
%         It implements the algorithms FISTA, ISTA and subgradient descent for solving  
%  
%           min\_W  loss(W) + lambda psi(W)  
%            
%         The function psi are those used by mexProximalTree (see documentation)  
%         for the loss functions, see the documentation of mexFistaFlat  
%  
%         This function can also handle intercepts (last row of W is not regularized),  
%         and/or non-negativity constraints on W and sparse matrices X  
%  
% Inputs: Y:  double dense m x n matrix  
%         X:  double dense or sparse m x p matrix     
%         W0:  double dense p x n matrix or p x Nn matrix (for multi-logistic loss)  
%              initial guess  
%         tree: struct (see documentation of mexProximalTree)  
%         param: struct  
%            param.loss (choice of loss, see above)  
%            param.regul (choice of regularization, see function mexProximalFlat)  
%            param.lambda (regularization parameter)  
%            param.lambda2 (optional, regularization parameter, 0 by default)  
%            param.lambda3 (optional, regularization parameter, 0 by default)  
%            param.verbose (optional, verbosity level, false by default)  
%            param.pos (optional, adds positivity constraints on the  
%                coefficients, false by default)  
%            param.transpose (optional, transpose the matrix in the regularization function)  
%            param.size\_group (optional, for regularization functions assuming a group  
%                 structure)  
%            param.numThreads (optional, number of threads for exploiting  
%                multi-core / multi-cpus. By default, it takes the value -1,  
%                which automatically selects all the available CPUs/cores).  
%            param.max\_it (optional, maximum number of iterations, 100 by default)  
%            param.it0 (optional, frequency for computing duality gap, every 10 iterations by default)  
%            param.tol (optional, tolerance for stopping criteration, which is a relative duality gap  
%                if it is available, or a relative change of parameters).  
%            param.gamma (optional, multiplier for increasing the parameter L in fista, 1.5 by default)  
%            param.L0 (optional, initial parameter L in fista, 0.1 by default, should be small enough)  
%            param.fixed\_step (deactive the line search for L in fista and use param.L0 instead)  
%            param.compute\_gram (optional, pre-compute X^TX, false by default).  
%            param.intercept (optional, do not regularize last row of W, false by default).  
%            param.ista (optional, use ista instead of fista, false by default).  
%            param.subgrad (optional, if not param.ista, use subradient descent instead of fista, false by default).  
%            param.a, param.b (optional, if param.subgrad, the gradient step is a/(t+b)  
%            also similar options as mexProximalTree  
%  
%            the function also implements the ADMM algorithm via an option param.admm=true. It is not documented  
%            and you need to look at the source code to use it.  
%  
% Output:  W:  double dense p x n matrix or p x Nn matrix (for multi-logistic loss)  
%          optim: optional, double dense 4 x n matrix.  
%              first row: values of the objective functions.  
%              third row: values of the relative duality gap (if available)  
%              fourth row: number of iterations  
%  
% Author: Julien Mairal, 2010

The following piece of code illustrates how to use this function.

format compact;  
param.num\_threads=-1; % all cores (-1 by default)  
param.verbose=false;   % verbosity, false by default  
param.lambda=0.001; % regularization parameter  
param.it0=10;      % frequency for duality gap computations  
param.max\_it=200; % maximum number of iterations  
param.L0=0.1;  
param.tol=1e-5;  
param.intercept=false;  
param.pos=false;  
  
% Example 2 of tree structure  
% tree structured groups:  
% g1= {0 1 2 3 4 5 6 7 8 9}    root(g1) = { };  
% g2= {0 1 2 3 4 5}            root(g2) = {0 1 2};  
% g3= {3 4}                    root(g3) = {3 4};  
% g4= {5}                      root(g4) = {5};  
% g5= {6 7 8 9}                root(g5) = { };  
% g6= {6 7}                    root(g6) = {6 7};  
% g7= {8 9}                    root(g7) = {8};  
% g8 = {9}                     root(g8) = {9};  
tree.own\_variables=  int32([0 0 3 5 6 6 8 9]);   % pointer to the first variable of each group  
tree.N\_own\_variables=int32([0 3 2 1 0 2 1 1]); % number of "root" variables in each group  
tree.eta\_g=[1 1 1 2 2 2 2.5 2.5];  
tree.groups=sparse([0 0 0 0 0 0 0 0; ...  
                    1 0 0 0 0 0 0 0; ...  
                    0 1 0 0 0 0 0 0; ...  
                    0 1 0 0 0 0 0 0; ...  
                    1 0 0 0 0 0 0 0; ...  
                    0 0 0 0 1 0 0 0; ...  
                    0 0 0 0 1 0 0 0; ...  
                    0 0 0 0 0 0 1 0]);  % first group should always be the root of the tree  
  
X=randn(100,10);  
X=X-repmat(mean(X),[size(X,1) 1]);  
X=mexNormalize(X);  
Y=randn(100,100);  
Y=Y-repmat(mean(Y),[size(Y,1) 1]);  
Y=mexNormalize(Y);  
W0=zeros(size(X,2),size(Y,2));  
% Regression experiments   
% 100 regression problems with the same design matrix X.  
fprintf('\nVarious regression experiments\n');  
param.compute\_gram=true;  
fprintf('\nFISTA + Regression tree-l2\n');  
param.loss='square';  
param.regul='tree-l2';  
tic  
[W optim\_info]=mexFistaTree(Y,X,W0,tree,param);  
t=toc;  
fprintf('mean loss: %f, time: %f, number of iterations: %f\n',mean(optim\_info(1,:)),t,mean(optim\_info(4,:)));  
  
fprintf('\nFISTA + Regression tree-linf\n');  
param.regul='tree-linf';  
tic  
[W optim\_info]=mexFistaTree(Y,X,W0,tree,param);  
t=toc;  
fprintf('mean loss: %f, mean relative duality\_gap: %f, time: %f, number of iterations: %f\n',mean(optim\_info(1,:)),mean(optim\_info(3,:)),t,mean(optim\_info(4,:)));  
  
% works also with non tree-structured regularization. tree is ignored  
fprintf('\nFISTA + Regression Fused-Lasso\n');  
param.regul='fused-lasso';  
param.lambda2=0.001;  
param.lambda3=0.001; %  
tic  
[W optim\_info]=mexFistaTree(Y,X,W0,tree,param);  
t=toc;  
fprintf('mean loss: %f, time: %f, number of iterations: %f\n',mean(optim\_info(1,:)),t,mean(optim\_info(4,:)));  
  
fprintf('\nISTA + Regression tree-l0\n');  
param.regul='tree-l0';  
tic  
[W optim\_info]=mexFistaTree(Y,X,W0,tree,param);  
t=toc;  
fprintf('mean loss: %f, time: %f, number of iterations: %f\n',mean(optim\_info(1,:)),t,mean(optim\_info(4,:)));  
  
fprintf('\nFISTA + Regression tree-l2 with intercept \n');  
param.intercept=true;  
param.regul='tree-l2';  
tic  
[W optim\_info]=mexFistaTree(Y,[X ones(size(X,1),1)],[W0; zeros(1,size(W0,2))],tree,param);  
t=toc;  
fprintf('mean loss: %f, time: %f, number of iterations: %f\n',mean(optim\_info(1,:)),t,mean(optim\_info(4,:)));  
param.intercept=false;  
  
% Classification  
fprintf('\nOne classification experiment\n');  
Y=2\*double(randn(100,size(Y,2)) > 0)-1;  
fprintf('\nFISTA + Logistic + tree-linf\n');  
param.regul='tree-linf';  
param.loss='logistic';  
param.lambda=0.001;  
tic  
[W optim\_info]=mexFistaTree(Y,X,W0,tree,param);  
t=toc;  
fprintf('mean loss: %f, mean relative duality\_gap: %f, time: %f, number of iterations: %f\n',mean(optim\_info(1,:)),mean(optim\_info(3,:)),t,mean(optim\_info(4,:)));  
% can be used of course with other regularization functions, intercept,...  
  
% Multi-Class classification  
Y=double(ceil(5\*rand(100,size(Y,2)))-1);  
param.loss='multi-logistic';  
param.regul='tree-l2';  
fprintf('\nFISTA + Multi-Class Logistic + tree-l2 \n');  
tic  
nclasses=max(Y(:))+1;  
W0=zeros(size(X,2),nclasses\*size(Y,2));  
[W optim\_info]=mexFistaTree(Y,X,W0,tree,param);  
t=toc;  
fprintf('mean loss: %f, time: %f, number of iterations: %f\n',mean(optim\_info(1,:)),t,mean(optim\_info(4,:)));  
% can be used of course with other regularization functions, intercept,...  
  
% Multi-Task regression  
Y=randn(100,size(Y,2));  
Y=Y-repmat(mean(Y),[size(Y,1) 1]);  
Y=mexNormalize(Y);  
param.compute\_gram=false;  
param.verbose=true;   % verbosity, false by default  
W0=zeros(size(X,2),size(Y,2));  
param.loss='square';  
fprintf('\nFISTA + Regression multi-task-tree \n');  
param.regul='multi-task-tree';  
param.lambda2=0.001;  
tic  
[W optim\_info]=mexFistaTree(Y,X,W0,tree,param);  
toc  
fprintf('mean loss: %f, mean relative duality\_gap: %f, time: %f, number of iterations: %f\n',mean(optim\_info(1,:)),mean(optim\_info(3,:)),t,mean(optim\_info(4,:)));  
  
% Multi-Task Classification  
fprintf('\nFISTA + Logistic + multi-task-tree \n');  
param.regul='multi-task-tree';  
param.lambda2=0.001;  
param.loss='logistic';  
Y=2\*double(randn(100,size(Y,2)) > 0)-1;  
tic  
[W optim\_info]=mexFistaTree(Y,X,W0,tree,param);  
toc  
fprintf('mean loss: %f, mean relative duality\_gap: %f, time: %f, number of iterations: %f\n',mean(optim\_info(1,:)),mean(optim\_info(3,:)),t,mean(optim\_info(4,:)));  
  
% Multi-Class + Multi-Task Regularization  
param.verbose=false;  
fprintf('\nFISTA + Multi-Class Logistic +multi-task-tree \n');  
Y=double(ceil(5\*rand(100,size(Y,2)))-1);  
param.loss='multi-logistic';  
param.regul='multi-task-tree';  
tic  
nclasses=max(Y(:))+1;  
W0=zeros(size(X,2),nclasses\*size(Y,2));  
[W optim\_info]=mexFistaTree(Y,X,W0,tree,param);  
t=toc;  
fprintf('mean loss: %f, mean relative duality\_gap: %f, time: %f, number of iterations: %f\n',mean(optim\_info(1,:)),mean(optim\_info(3,:)),t,mean(optim\_info(4,:)));  
% can be used of course with other regularization functions, intercept,...  
  
fprintf('\nFISTA + Multi-Class Logistic +multi-task-tree + sparse matrix \n');  
tic  
nclasses=max(Y(:))+1;  
W0=zeros(size(X,2),nclasses\*size(Y,2));  
[W optim\_info]=mexFistaTree(Y,sparse(X),W0,tree,param);  
t=toc;  
fprintf('mean loss: %f, mean relative duality\_gap: %f, time: %f, number of iterations: %f\n',mean(optim\_info(1,:)),mean(optim\_info(3,:)),t,mean(optim\_info(4,:)));

### 5.10  Function mexFistaGraph

Given a matrix X=[x^1^,…,x^p^]^T^ in ℝ^m × p^, and a matrix Y=[y^1^,…,y^n^], it solves the optimization problems presented in the previous section, with the same regularization functions as mexProximalGraph.
see usage details below:

%   
% Usage: [W [optim]]=mexFistaGraph(Y,X,W0,graph,param);  
%  
% Name: mexFistaGraph  
%  
% Description: mexFistaGraph solves sparse regularized problems.  
%         X is a design matrix of size m x p  
%         X=[x^1,...,x^n]', where the x\_i's are the rows of X  
%         Y=[y^1,...,y^n] is a matrix of size m x n  
%         It implements the algorithms FISTA, ISTA and subgradient descent.  
%  
%         It implements the algorithms FISTA, ISTA and subgradient descent for solving  
%  
%           min\_W  loss(W) + lambda psi(W)  
%            
%         The function psi are those used by mexProximalGraph (see documentation)  
%         for the loss functions, see the documentation of mexFistaFlat  
%           
%         This function can also handle intercepts (last row of W is not regularized),  
%         and/or non-negativity constraints on W.  
%  
% Inputs: Y:  double dense m x n matrix  
%         X:  double dense or sparse m x p matrix     
%         W0:  double dense p x n matrix or p x Nn matrix (for multi-logistic loss)  
%              initial guess  
%         graph: struct (see documentation of mexProximalGraph)  
%         param: struct  
%            param.loss (choice of loss, see above)  
%            param.regul (choice of regularization, see function mexProximalFlat)  
%            param.lambda (regularization parameter)  
%            param.lambda2 (optional, regularization parameter, 0 by default)  
%            param.lambda3 (optional, regularization parameter, 0 by default)  
%            param.verbose (optional, verbosity level, false by default)  
%            param.pos (optional, adds positivity constraints on the  
%                coefficients, false by default)  
%            param.numThreads (optional, number of threads for exploiting  
%                multi-core / multi-cpus. By default, it takes the value -1,  
%                which automatically selects all the available CPUs/cores).  
%            param.max\_it (optional, maximum number of iterations, 100 by default)  
%            param.it0 (optional, frequency for computing duality gap, every 10 iterations by default)  
%            param.tol (optional, tolerance for stopping criteration, which is a relative duality gap  
%                if it is available, or a relative change of parameters).  
%            param.gamma (optional, multiplier for increasing the parameter L in fista, 1.5 by default)  
%            param.L0 (optional, initial parameter L in fista, 0.1 by default, should be small enough)  
%            param.fixed\_step (deactive the line search for L in fista and use param.L0 instead)  
%            param.compute\_gram (optional, pre-compute X^TX, false by default).  
%            param.intercept (optional, do not regularize last row of W, false by default).  
%            param.ista (optional, use ista instead of fista, false by default).  
%            param.subgrad (optional, if not param.ista, use subradient descent instead of fista, false by default).  
%            param.a, param.b (optional, if param.subgrad, the gradient step is a/(t+b)  
%            also similar options as mexProximalTree  
%  
%            the function also implements the ADMM algorithm via an option param.admm=true. It is not documented  
%            and you need to look at the source code to use it.  
%  
%  
% Output:  W:  double dense p x n matrix or p x Nn matrix (for multi-logistic loss)  
%          optim: optional, double dense 4 x n matrix.  
%              first row: values of the objective functions.  
%              third row: values of the relative duality gap (if available)  
%              fourth row: number of iterations  
%  
% Author: Julien Mairal, 2010

The following piece of code illustrates how to use this function.

clear all;  
randn('seed',0);  
format compact;  
param.num\_threads=-1; % all cores (-1 by default)  
param.verbose=false;   % verbosity, false by default  
param.lambda=0.1; % regularization parameter  
param.it0=1;      % frequency for duality gap computations  
param.max\_it=100; % maximum number of iterations  
param.L0=0.1;  
param.tol=1e-5;  
param.intercept=false;  
param.pos=false;  
  
graph.eta\_g=[1 1 1 1 1];  
graph.groups=sparse([0 0 0 1 0;  
                     0 0 0 0 0;  
                     0 0 0 0 0;  
                     0 0 0 0 0;  
                     0 0 1 0 0]);   % g5 is included in g3, and g2 is included in g4  
graph.groups\_var=sparse([1 0 0 0 0;  
                         1 0 0 0 0;  
                         1 0 0 0 0 ;  
                         1 1 0 0 0;  
                         0 1 0 1 0;  
                         0 1 0 1 0;  
                         0 1 0 0 1;  
                         0 0 0 0 1;  
                         0 0 0 0 1;  
                         0 0 1 0 0]); % represents direct inclusion relations   
  
X=randn(100,10);  
param.verbose=true;  
%X=eye(10);  
X=X-repmat(mean(X),[size(X,1) 1]);  
X=mexNormalize(X);  
Y=randn(100,1);  
Y=Y-repmat(mean(Y),[size(Y,1) 1]);  
Y=mexNormalize(Y);  
W0=zeros(size(X,2),size(Y,2));  
% Regression experiments   
% 100 regression problems with the same design matrix X.  
fprintf('\nVarious regression experiments\n');  
param.compute\_gram=true;  
fprintf('\nFISTA + Regression graph\n');  
param.loss='square';  
param.regul='graph';  
tic  
[W optim\_info]=mexFistaGraph(Y,X,W0,graph,param);  
t=toc;  
fprintf('mean loss: %f, mean relative duality\_gap: %f, time: %f, number of iterations: %f\n',mean(optim\_info(1,:)),mean(optim\_info(3,:)),t,mean(optim\_info(4,:)));  
  
fprintf('\nADMM + Regression graph\n');  
param.admm=true;  
param.lin\_admm=true;  
param.c=1;  
param.delta=1;  
tic  
[W optim\_info]=mexFistaGraph(Y,X,W0,graph,param);  
t=toc;  
fprintf('mean loss: %f, stopping criterion: %f, time: %f, number of iterations: %f\n',mean(optim\_info(1,:)),mean(optim\_info(3,:)),t,mean(optim\_info(4,:)));  
  
param.admm=false;  
param.max\_it=5;  
param.it0=1;  
tic  
[W optim\_info]=mexFistaGraph(Y,X,W,graph,param);  
t=toc;  
fprintf('mean loss: %f, mean relative duality\_gap: %f, time: %f, number of iterations: %f\n',mean(optim\_info(1,:)),mean(optim\_info(3,:)),t,mean(optim\_info(4,:)));  
  
% works also with non graph-structured regularization. graph is ignored  
fprintf('\nFISTA + Regression Fused-Lasso\n');  
param.regul='fused-lasso';  
param.lambda2=0.01;  
param.lambda3=0.01; %  
tic  
[W optim\_info]=mexFistaGraph(Y,X,W0,graph,param);  
t=toc;  
fprintf('mean loss: %f, time: %f, number of iterations: %f\n',mean(optim\_info(1,:)),t,mean(optim\_info(4,:)));  
  
fprintf('\nFISTA + Regression graph with intercept \n');  
param.intercept=true;  
param.regul='graph';  
tic  
[W optim\_info]=mexFistaGraph(Y,[X ones(size(X,1),1)],[W0; zeros(1,size(W0,2))],graph,param);  
t=toc;  
fprintf('mean loss: %f, mean relative duality\_gap: %f, time: %f, number of iterations: %f\n',mean(optim\_info(1,:)),mean(optim\_info(3,:)),t,mean(optim\_info(4,:)));  
param.intercept=false;  
  
% Classification  
fprintf('\nOne classification experiment\n');  
Y=2\*double(randn(100,size(Y,2)) > 0)-1;  
fprintf('\nFISTA + Logistic + graph-linf\n');  
param.regul='graph';  
param.loss='logistic';  
param.lambda=0.01;  
tic  
[W optim\_info]=mexFistaGraph(Y,X,W0,graph,param);  
t=toc;  
fprintf('mean loss: %f, mean relative duality\_gap: %f, time: %f, number of iterations: %f\n',mean(optim\_info(1,:)),mean(optim\_info(3,:)),t,mean(optim\_info(4,:)));  
% can be used of course with other regularization functions, intercept,...  
  
% Multi-Class classification  
Y=double(ceil(5\*rand(100,size(Y,2)))-1);  
param.loss='multi-logistic';  
param.regul='graph';  
fprintf('\nFISTA + Multi-Class Logistic + graph \n');  
tic  
nclasses=max(Y(:))+1;  
W0=zeros(size(X,2),nclasses\*size(Y,2));  
[W optim\_info]=mexFistaGraph(Y,X,W0,graph,param);  
t=toc;  
fprintf('mean loss: %f, mean relative duality\_gap: %f, time: %f, number of iterations: %f\n',mean(optim\_info(1,:)),mean(optim\_info(3,:)),t,mean(optim\_info(4,:)));  
% can be used of course with other regularization functions, intercept,...  
  
% Multi-Task regression  
Y=randn(100,size(Y,2));  
Y=Y-repmat(mean(Y),[size(Y,1) 1]);  
Y=mexNormalize(Y);  
param.compute\_gram=false;  
param.verbose=true;   % verbosity, false by default  
W0=zeros(size(X,2),size(Y,2));  
param.loss='square';  
fprintf('\nFISTA + Regression multi-task-graph \n');  
param.regul='multi-task-graph';  
param.lambda2=0.01;  
tic  
[W optim\_info]=mexFistaGraph(Y,X,W0,graph,param);  
toc  
fprintf('mean loss: %f, mean relative duality\_gap: %f, time: %f, number of iterations: %f\n',mean(optim\_info(1,:)),mean(optim\_info(3,:)),t,mean(optim\_info(4,:)));  
  
% Multi-Task Classification  
fprintf('\nFISTA + Logistic + multi-task-graph \n');  
param.regul='multi-task-graph';  
param.lambda2=0.01;  
param.loss='logistic';  
Y=2\*double(randn(100,size(Y,2)) > 0)-1;  
tic  
[W optim\_info]=mexFistaGraph(Y,X,W0,graph,param);  
toc  
fprintf('mean loss: %f, mean relative duality\_gap: %f, time: %f, number of iterations: %f\n',mean(optim\_info(1,:)),mean(optim\_info(3,:)),t,mean(optim\_info(4,:)));  
% Multi-Class + Multi-Task Regularization  
  
param.verbose=false;  
fprintf('\nFISTA + Multi-Class Logistic +multi-task-graph \n');  
Y=double(ceil(5\*rand(100,size(Y,2)))-1);  
param.loss='multi-logistic';  
param.regul='multi-task-graph';  
tic  
nclasses=max(Y(:))+1;  
W0=zeros(size(X,2),nclasses\*size(Y,2));  
[W optim\_info]=mexFistaGraph(Y,X,W0,graph,param);  
t=toc;  
fprintf('mean loss: %f, mean relative duality\_gap: %f, time: %f, number of iterations: %f\n',mean(optim\_info(1,:)),mean(optim\_info(3,:)),t,mean(optim\_info(4,:)));  
% can be used of course with other regularization functions, intercept,...

### 5.11  Function mexFistaPathCoding

Similarly, the toolbox handles the penalties of [24] with the following function

%   
% Usage: [W [optim]]=mexFistaPathCoding(Y,X,W0,DAG,param);  
%  
% Name: mexFistaPathCoding  
%  
% Description: mexFistaPathCoding solves sparse regularized problems for the   
%         path coding penalties of http://arxiv.org/abs/1204.4539  
%         X is a design matrix of size m x p  
%         X=[x^1,...,x^n]', where the x\_i's are the rows of X  
%         Y=[y^1,...,y^n] is a matrix of size m x n  
%         It implements the algorithms FISTA, ISTA and subgradient descent.  
%  
%         It implements the algorithms FISTA, ISTA and subgradient descent for solving  
%  
%           min\_W  loss(W) + lambda psi(W)  
%            
%         The function psi are those used by mexProximalPathCoding (see documentation)  
%         for the loss functions, see the documentation of mexFistaFlat  
%           
%         This function can also handle intercepts (last row of W is not regularized),  
%         and/or non-negativity constraints on W.  
%  
% Inputs: Y:  double dense m x n matrix  
%         X:  double dense or sparse m x p matrix     
%         W0:  double dense p x n matrix or p x Nn matrix (for multi-logistic loss)  
%              initial guess  
%         DAG: struct (see documentation of mexProximalPathCoding)  
%         param: struct  
%            param.loss (choice of loss, see above)  
%            param.regul (choice of regularization, see function mexProximalPathCoding)  
%            param.lambda (regularization parameter)  
%            param.lambda2 (optional, regularization parameter, 0 by default)  
%            param.lambda3 (optional, regularization parameter, 0 by default)  
%            param.verbose (optional, verbosity level, false by default)  
%            param.pos (optional, adds positivity constraints on the  
%                coefficients, false by default)  
%            param.numThreads (optional, number of threads for exploiting  
%                multi-core / multi-cpus. By default, it takes the value -1,  
%                which automatically selects all the available CPUs/cores).  
%            param.max\_it (optional, maximum number of iterations, 100 by default)  
%            param.it0 (optional, frequency for computing duality gap, every 10 iterations by default)  
%            param.tol (optional, tolerance for stopping criteration, which is a relative duality gap  
%                if it is available, or a relative change of parameters).  
%            param.gamma (optional, multiplier for increasing the parameter L in fista, 1.5 by default)  
%            param.L0 (optional, initial parameter L in fista, 0.1 by default, should be small enough)  
%            param.fixed\_step (deactive the line search for L in fista and use param.L0 instead)  
%            param.compute\_gram (optional, pre-compute X^TX, false by default).  
%            param.intercept (optional, do not regularize last row of W, false by default).  
%            param.ista (optional, use ista instead of fista, false by default).  
%            param.subgrad (optional, if not param.ista, use subradient descent instead of fista, false by default).  
%            param.a, param.b (optional, if param.subgrad, the gradient step is a/(t+b)  
%            also similar options as mexProximalPathCoding  
%  
%  
% Output:  W:  double dense p x n matrix or p x Nn matrix (for multi-logistic loss)  
%          optim: optional, double dense 4 x n matrix.  
%              first row: values of the objective functions.  
%              third row: values of the relative duality gap (if available)  
%              fourth row: number of iterations  
%  
% Author: Julien Mairal, 2012

The following piece of code illustrates how to use this function.

clear all;  
rand('seed',0);  
randn('seed',0);  
fprintf('test mexFistaPathCoding\n');  
p=100;  
n=1000;  
% generate a DAG  
G=sprand(p,p,0.05);  
G=mexRemoveCyclesGraph(G);  
fprintf('\n');  
  
% generate a data matrix  
X=randn(n,p);  
X=X-repmat(mean(X),[size(X,1) 1]);  
X=mexNormalize(X);  
Y=randn(n,2);  
Y=Y-repmat(mean(Y),[size(Y,1) 1]);  
Y=mexNormalize(Y);  
W0=zeros(size(X,2),size(Y,2));  
  
% input graph  
graph.weights=G;  
graph.stop\_weights=zeros(1,p);  
graph.start\_weights=10\*ones(1,p);  
  
% FISTA parameters  
param.num\_threads=-1; % all cores (-1 by default)  
param.verbose=true;   % verbosity, false by default  
param.lambda=0.005; % regularization parameter  
param.it0=1;      % frequency for duality gap computations  
param.max\_it=100; % maximum number of iterations  
param.L0=0.01;  
param.tol=1e-4;  
param.precision=10000000;  
param.pos=false;  
  
fprintf('Square Loss + convex path penalty\n');  
param.loss='square';  
param.regul='graph-path-conv';  
tic  
[W1 optim\_info]=mexFistaPathCoding(Y,X,W0,graph,param);  
t=toc;  
fprintf('mean loss: %f, mean relative duality\_gap: %f, time: %f, number of iterations: %f\n',mean(optim\_info(1,:)),mean(optim\_info(3,:)),t,mean(optim\_info(4,:)));  
num=mexCountConnexComponents(graph.weights,W1(:,1));  
fprintf('Num of connected components: %d\n',num);  
  
fprintf('\n');  
fprintf('Square Loss + non-convex path penalty\n');  
param.loss='square';  
param.regul='graph-path-l0';  
param.lambda=0.0001; % regularization parameter  
param.ista=true;  
tic  
[W2 optim\_info]=mexFistaPathCoding(Y,X,W0,graph,param);  
t=toc;  
num=mexCountConnexComponents(graph.weights,W2(:,1));  
fprintf('Num of connected components: %d\n',num);  
  
fprintf('\n');  
fprintf('Note that for non-convex penalties, continuation strategies sometimes perform better:\n');  
tablambda=param.lambda\*sqrt(sqrt(sqrt(2))).^(20:-1:0);  
lambda\_orig=param.lambda;  
tic  
W2=W0;  
for ii = 1:length(tablambda)  
   param.lambda=tablambda(ii);  
   param.verbose=false;  
   [W2]=mexFistaPathCoding(Y,X,W2,graph,param);  
end  
param.verbose=true;  
param.lambda=lambda\_orig;  
[W2 optim\_info]=mexFistaPathCoding(Y,X,W2,graph,param);  
t=toc;  
num=mexCountConnexComponents(graph.weights,W2(:,1));  
param.ista=false;  
fprintf('Num of connected components: %d\n',num);

### 5.12  Function solverPoisson

The following problem is addressed here

min

w ∈ ℝ_+_^p^

p

∑

j=1

x_i_^⊤^w + δ − y_i_ log(x_i_^⊤^w +δ) + λ ψ(w).

%   
% Usage:   [W, [optim]]=solverPoisson(Y,X,W0,param);  
%  
% Name: solverPoisson  
%  
% Description: solverPoisson solves the regularized Poisson regression  
% problem for every column of Y:  
%  
%   min\_w  \sum\_i (x\_i' w) + delta  - y\_i log( x\_i' w + delta) + lambda psi(w) (1)  
%  
% delta should be positive. The method solves (1) for decreasing values of delta,  
% until it reaches the desired target value. The algorithm ISTA is used  
% with Barzilai-Borwein steps.  
%  
% Inputs: Y:  double m x n matrix (non-negative values)  
%         X:  double m x p matrix (non-negative values)  
%         W0:  double p x n matrix (non-negative values)  
%         param: struct  
%            param.tol, param.max\_it, param.verbose, param.L0,  
%            param.it0, param.lambda as for mexFistaFlat  
%            param.regul,  as for mexProximalFlat  
%  
% Output:  
%         W: double p x n matrix (non-negative values)  
  
function [W optim] = solverPoisson(Y,X,W0,param)  
param.ista=true;  
param.intercept=false;  
param.pos=true;  
param.linesearch\_mode=2;  
param.loss='poisson';  
tabdelta=logspace(0,log10(param.delta),-log10(param.delta));  
for delta = tabdelta  
   param2=param;  
   param2.delta=delta;  
   [W optim]=mexFistaFlat(Y,X,W0,param2);  
   W0=W;  
end

The following piece of code illustrates how to use this function.

rand('seed',0);  
randn('seed',0);  
format compact;  
randn('seed',0);  
param.numThreads=-1; % all cores (-1 by default)  
param.verbose=true;   % verbosity, false by default  
param.lambda=0.05; % regularization parameter  
param.it0=10;      % frequency for duality gap computations  
param.max\_it=200; % maximum number of iterations  
param.L0=0.01;  
param.tol=1e-6;  
param.delta=1e-5;  
  
X=rand(1000,2000);  
X=mexNormalize(X);  
Y=rand(1000,1);  
Y=mexNormalize(Y);  
W0=zeros(size(X,2),size(Y,2));  
% Regression experiments   
% 100 regression problems with the same design matrix X.  
fprintf('\nVarious regression experiments\n');  
fprintf('\nFISTA + Regression l1\n');  
param.regul='l1';  
tic  
[W optim\_info]=solverPoisson(Y,X,W0,param);  
t=toc;  
fprintf('mean loss: %f, mean relative duality\_gap: %f, time: %f, number of iterations: %f\n',mean(optim\_info(1,:)),mean(optim\_info(3,:)),t,mean(optim\_info(4,:)));

### 5.13  Function mexIncrementalProx

This implements the incremental solver MISO [25].

%   
% Usage:  [W [optim]]=mexIncrementalProx(y,X,W0,param);  
%  
% Name: mexIncrementalProx  
%  
% Description: mexIncremrentalProx implements the incremental algorithm MISO  
% for composite optimization in a large scale setting.  
%        X is a design matrix of size p x n  
%        y is a vector of size n   
% WARNING, X is transposed compared to the functions mexFista\*, and y is a vector  
%        param.lambda is a vector that contains nlambda different values of the  
%        regularization parameter (it can be a scalar, in that case nlambda=1)  
%        W0: is a dense matrix of size p x nlambda   It is in fact ineffective  
%        in the current release  
%        W: is the output, dense matrix of size p x nlambda  
%  
%         - if param.loss='square' and param.regul corresponds to a regularization  
%           function (currently 'l1' or 'l2'), the following problem is solved  
%           w = argmin (1/n)sum\_{i=1}^n 0.5(y\_i- x\_i^T w)^2 + lambda psi(w)  
%         - if param.loss='logistic' and param.regul corresponds to a regularization  
%           function (currently 'l1' or 'l2'), the following problem is solved  
%           w = argmin (1/n)sum\_{i=1}^n log(1+ exp(-y\_ix\_i^T w)) + lambda psi(w)  
%           Note that here, the y\_i's should be -1 or +1   
%            
%         The current release does not handle intercepts  
%  
% Inputs: y: double dense vector of size n  
%         X: dense or sparse matrix of size p x n  
%         W0: dense matrix of size p x nlambda  
%         param: struct  
%           param.loss (choice of loss)  
%           param.regul (choice of regularization function)  
%           param.lambda : vector of size nlambda  
%           param.epochs: number of passes over the data  
%           param.minibatches: size of the mini-batches: recommended value is 1  
%              if X is dense, and min(n,ceil(1/density)) if X is sparse  
%           param.warm\_restart : (path-following strategy, very efficient when  
%              providing an array of lambdas, false by default)  
%           param.normalized : (optional, can be set to true if the x\_i's have  
%              unit l2-norm, false by default)  
%           param.strategy (optional, 3 by default)  
%                          0: no heuristics, slow  (only for comparison purposes)  
%                          1: adjust the constant L on 5% of the data   
%                          2: adjust the constant L on 5% of the data + unstable heuristics (this strategy does not work)  
%                          3: adjust the constant L on 5% of the data + stable heuristic (this is by far the best choice)  
%           param.numThreads (optional, number of threads)  
%           param.verbose (optional)  
%           param.seed (optional, choice of the random seed)  
%  
% Output:  W:  double dense p x nlambda matrix  
%          optim: optional, double dense 3 x nlambda matrix.  
%              first row: values of the objective functions.  
%              third row: computational time   
%  
% Author: Julien Mairal, 2013

The following piece of code illustrates how to use this function.

clear all;  
n=400000;  
p=1000;  
density=0.01;  
  
% generate random data  
format compact;  
randn('seed',0);  
rand('seed',0);  
X=sprandn(p,n,density);  
mean\_nrm=mean(sqrt(sum(X.^2)));  
X=X/mean\_nrm;  
  
% generate some true model  
z=double(sign(full(sprandn(p,1,0.05))));  
y=X'\*z;  
  
%%%%%%%%%%%%%%%%%%%%%%%%%%%%%%%%%%%%%  
% EXPERIMENT 1: Lasso  
%%%%%%%%%%%%%%%%%%%%%%%%%%%%%%%%%%%%%  
fprintf('EXPERIMENT FOR LASSO\n');  
nrm=sqrt(sum(y.^2));  
y=y+0.01\*nrm\*randn(n,1);    % add noise to the model  
nrm=sqrt(sum(y.^2));  
y=y\*(sqrt(n)/nrm);  
  
% set optimization parameters  
clear param;  
param.regul='l1';        % many other regularization functions are available  
param.loss='square';     % only square and log are available  
param.numThreads=-1;    % uses all possible cores  
param.normalized=false;  % if the columns of X have unit norm, set to true.  
param.strategy=1;        % MISO with all heuristics  
                         % 0: no heuristics, slow  (only for comparison purposes)  
                         % 1: MISO1: adjust the constant L on 5% of the data  (good for non-strongly convex problems)  
                         % 2: adjust the constant L on 5% of the data + unstable heuristics (this strategy does not work)  
                         % 3: MISO2: adjust the constant L on 5% of the data + stable heuristic good for strongly-convex problems  
                         % 4: MISOmu: best for l2 regularization  
param.verbose=true;  
  
% set grid of lambda  
max\_lambda=max(abs(X\*y))/n;  
tablambda=max\_lambda\*(2^(1/4)).^(0:-1:-20);  % order from large to small  
tabepochs=[1 2 3 5 10];  % in this script, we compare the results obtained when changing the number of passes on the data.  
param.lambda=tablambda;  
%% The problem which will be solved is  
%%   min\_beta  1/(2n) ||y-X' beta||\_2^2 + lambda ||beta||\_1  
% the problems for different lambdas are solve INDEPENDENTLY in parallel  
fprintf('EXPERIMENT: ALL LAMBDAS WITHOUT WARM RESTART\n');  
param.warm\_restart=false;  
param.verbose=false;  
param.strategy=1;  
param.minibatches=min(n,ceil(1/density));  % size of the minibatches, requires to store twice the size of X  with strategy=1  
obj=[];  
spar=[];  
for ii=1:length(tabepochs)  
   param.epochs=tabepochs(ii);   % one pass over the data  
   fprintf('EXP WITH %d PASS OVER THE DATA\n',tabepochs(ii));  
   nlambdas=length(param.lambda);  
   Beta0=zeros(p,nlambdas);  
   tic  
   [Beta tmp]=mexIncrementalProx(y,X,Beta0,param);  
   toc  
   fprintf('Objective functions: \n');  
   obj=[obj; tmp(1,:)];  
   obj  
   fprintf('Sparsity: \n');  
   spar=[spar; sum(Beta ~= 0)];  
   spar  
end  
  
% the problems are here solved sequentially with warm restart  
% this seems to be the prefered choice.  
fprintf('EXPERIMENT: SEQUENTIAL LAMBDAS WITH WARM RESTART\n');  
fprintf('A SINGLE CORE IS USED\n');  
param.warm\_restart=true;  
param.num\_threads=1;  
obj=[];  
spar=[];  
for ii=1:length(tabepochs)  
   param.epochs=tabepochs(ii);   % one pass over the data  
   fprintf('EXP WITH %d PASS\n',tabepochs(ii));  
   nlambdas=length(param.lambda);  
   Beta0=zeros(p,nlambdas);  
   tic  
   [Beta tmp]=mexIncrementalProx(y,X,Beta0,param);  
   toc  
   fprintf('Objective functions: \n');  
   obj=[obj; tmp(1,:)];  
   obj  
   fprintf('Sparsity: \n');  
   spar=[spar; sum(Beta ~= 0)];  
   spar  
end  
  
%%%%%%%%%%%%%%%%%%%%%%%%%%%%%%%%%%%%%  
% EXPERIMENT 2: L2 logistic regression   
%%%%%%%%%%%%%%%%%%%%%%%%%%%%%%%%%%%%%  
fprintf('EXPERIMENT FOR LOGISTIC REGRESSION + l2\n');  
y=sign(y);  
param.regul='l2';        % many other regularization functions are available  
param.loss='logistic';     % only square and log are available  
param.num\_threads=-1;    % uses all possible cores  
param.strategy=4;  
param.minibatches=1; % with strategy=4, minibatches is better set to 1  
%param.strategy=3;        % MISO with all heuristics  
fprintf('EXPERIMENT: ALL LAMBDAS WITHOUT WARM RESTART\n');  
param.warm\_restart=false;  
param.verbose=false;  
obj=[];  
spar=[];  
for ii=1:length(tabepochs)  
   param.epochs=tabepochs(ii);   % one pass over the data  
   fprintf('EXP WITH %d PASS\n',tabepochs(ii));  
   nlambdas=length(param.lambda);  
   Beta0=zeros(p,nlambdas);  
   tic  
   [Beta tmp]=mexIncrementalProx(y,X,Beta0,param);  
   toc  
   yR=repmat(y,[1 nlambdas]);  
   fprintf('Objective functions: \n');  
   obj=[obj;tmp(1,:)];  
   obj  
end  
  
  
fprintf('EXPERIMENT FOR LOGISTIC REGRESSION + l2\n');  
y=sign(y);  
param.regul='l2';        % many other regularization functions are available  
param.loss='logistic';     % only square and log are available  
param.num\_threads=-1;    % uses all possible cores  
param.strategy=3;         % MISO2  
param.minibatches=min(n,ceil(1/density));  % size of the minibatches, requires to store twice the size of X   
fprintf('EXPERIMENT: ALL LAMBDAS WITHOUT WARM RESTART\n');  
param.warm\_restart=false;  
param.verbose=false;  
obj=[];  
spar=[];  
for ii=1:length(tabepochs)  
   param.epochs=tabepochs(ii);   % one pass over the data  
   fprintf('EXP WITH %d PASS\n',tabepochs(ii));  
   nlambdas=length(param.lambda);  
   Beta0=zeros(p,nlambdas);  
   tic  
   [Beta tmp]=mexIncrementalProx(y,X,Beta0,param);  
   toc  
   yR=repmat(y,[1 nlambdas]);  
   fprintf('Objective functions: \n');  
   obj=[obj;tmp(1,:)];  
   obj  
end  
  
%%%%%%%%%%%%%%%%%%%%%%%%%%%%%%%%%%%%%  
% EXPERIMENT 3: L1 logistic regression   
%%%%%%%%%%%%%%%%%%%%%%%%%%%%%%%%%%%%%  
  
y=sign(y);  
fprintf('EXPERIMENT FOR LOGISTIC REGRESSION + l1\n');  
param.regul='l1';        % many other regularization functions are available  
param.loss='logistic';     % only square and log are available  
param.num\_threads=-1;    % uses all possible cores  
param.strategy=1;  
param.minibatches=min(n,ceil(1/density));  % size of the minibatches, requires to store twice the size of X   
fprintf('EXPERIMENT: ALL LAMBDAS WITHOUT WARM RESTART\n');  
param.warm\_restart=false;  
param.verbose=false;  
param.lambda=tablambda;  
obj=[];  
spar=[];  
for ii=1:length(tabepochs)  
   param.epochs=tabepochs(ii);   % one pass over the data  
   fprintf('EXP WITH %d PASS\n',tabepochs(ii));  
   nlambdas=length(param.lambda);  
   Beta0=zeros(p,nlambdas);  
   tic  
   [Beta tmp]=mexIncrementalProx(y,X,Beta0,param);  
   toc  
   yR=repmat(y,[1 nlambdas]);  
   fprintf('Objective functions: \n');  
   obj=[obj;tmp(1,:)];  
   obj  
end

### 5.14  Function mexStochasticProx

This implements the stochastic proximal gradient solver [26].

%   
% Usage:  [W [W2]]=mexStochasticProx(y,X,W0,param);  
%  
% Name: mexStochasticProx  
%  
% Description: mexStochasticProx implements a proximal MM stochastic algorithm   
% for composite optimization in a large scale setting.  
%        X is a design matrix of size p x n  
%        y is a vector of size n   
% WARNING, X is transposed compared to the functions mexFista\*, and y is a vector  
%        param.lambda is a vector that contains nlambda different values of the  
%        regularization parameter (it can be a scalar, in that case nlambda=1)  
%        W0: is a dense matrix of size p x nlambda   It is in fact ineffective  
%        in the current release  
%        W: is the output, dense matrix of size p x nlambda  
%  
%         - if param.loss='square' and param.regul corresponds to a regularization  
%           function (currently 'l1' or 'l2'), the following problem is solved  
%           w = argmin (1/n)sum\_{i=1}^n 0.5(y\_i- x\_i^T w)^2 + lambda psi(w)  
%         - if param.loss='logistic' and param.regul corresponds to a regularization  
%           function (currently 'l1' or 'l2'), the following problem is solved  
%           w = argmin (1/n)sum\_{i=1}^n log(1+ exp(-y\_ix\_i^T w)) + lambda psi(w)  
%           Note that here, the y\_i's should be -1 or +1   
%            
%         The current release does not handle intercepts  
%  
% Inputs: y: double dense vector of size n  
%         X: dense or sparse matrix of size p x n  
%         W0: dense matrix of size p x nlambda  
%         param: struct  
%           param.loss (choice of loss)  
%           param.regul (choice of regularization function)  
%           param.lambda : vector of size nlambda  
%           param.iters : number of iterations (n corresponds to one pass over the data)  
%           param.minibatches: size of the mini-batches: recommended value is 1  
%           param.normalized : (optional, can be set to true if the x\_i's have  
%              unit l2-norm, false by default)  
%           param.weighting\_mode : (optional, 1 by default),  
%                0:  w\_t = (t\_0+1)/(t+t\_0)  
%                1:  w\_t = ((t\_0+1)/(t+t\_0))^(0.75)  
%                2:  w\_t = ((t\_0+1)/(t+t\_0))^(5)  
%           param.averaging\_mode: (optional, false by default)  
%                0: no averaging  
%                1: first averaging mode for W2  
%                2: second averaging mode for W2  
%                WARNING: averaging can be very slow for sparse solutions  
%           param.determineEta (optional, automatically choose the parameters of the  
%             learning weights w\_t, true by default)   
%           param.t0 (optional, set up t0 for weights w\_t = ((1+t0)/(t+t0))^(alpha)  
%           param.numThreads (optional, number of threads)  
%           param.verbose (optional)  
%           param.seed (optional, choice of the random seed)  
%  
% Output:  W:  double dense p x nlambda matrix (contains the solution without averaging)  
%          W2:  double dense p x nlambda matrix (contains the solution with averaging)  
%  
% Author: Julien Mairal, 2013

The following piece of code illustrates how to use this function.

clear all;  
n=400000;  
p=1000;  
density=0.01;  
  
% generate random data  
format compact;  
randn('seed',0);  
rand('seed',0);  
X=sprandn(p,n,density);  
nrm=sqrt(sum(X.^2));  
X=X/mean(nrm);  
%mean\_nrm=mean();  
%X=X/mean\_nrm;  
  
% generate some true model  
z=double(sign(full(sprandn(p,1,0.05))));  
y=X'\*z;  
  
%%%%%%%%%%%%%%%%%%%%%%%%%%%%%%%%%%%%%  
% EXPERIMENT 1: Lasso  
%%%%%%%%%%%%%%%%%%%%%%%%%%%%%%%%%%%%%  
fprintf('EXPERIMENT FOR LASSO\n');  
nrm=sqrt(sum(y.^2));  
y=y+0.01\*nrm\*randn(n,1);    % add noise to the model  
nrm=sqrt(sum(y.^2));  
y=y\*(sqrt(n)/nrm);  
  
clear param;  
param.regul='l1';        % many other regularization functions are available  
param.loss='square';     % only square and log are available  
param.numThreads=-1;    % uses all possible cores  
param.normalized=false;  % if the columns of X have unit norm, set to true.  
param.averaging\_mode=0;  % no averaging, averaging was not really useful in experiments  
param.weighting\_mode=0;  % weights are in O(1/sqrt(n))  (see help mexStochasticProx)  
param.optimized\_solver=true;  % best is not to touch this option  
param.verbose=false;  
  
% set grid of lambda  
max\_lambda=max(abs(X\*y))/n;  
tablambda=max\_lambda\*(2^(1/4)).^(0:-1:-20);  % order from large to small  
param.lambda=tablambda;    % best to order from large to small  
tabepochs=[1 2 3 5 10];  % in this script, we compare the results obtained when varying the number of passes over the data.  
  
%% The problem which will be solved is  
%%   min\_beta  1/(2n) ||y-X' beta||\_2^2 + lambda ||beta||\_1  
fprintf('EXPERIMENT: ALL LAMBDAS IN PARALLEL\n');  
% we try different experiments when varying the number of epochs.  
% the problems for different lambdas are solve INDEPENDENTLY in parallel  
obj=[];  
objav=[];  
for ii=1:length(tabepochs)  
   param.iters=tabepochs(ii)\*n;  
   fprintf('EXP WITH %d PASS\n',tabepochs(ii));  
   nlambdas=length(param.lambda);  
   Beta0=zeros(p,nlambdas);  
   tic  
   [Beta tmp]=mexStochasticProx(y,X,Beta0,param);  
   toc  
   yR=repmat(y,[1 nlambdas]);  
   fprintf('Objective functions: \n');  
   obj=[obj; 0.5\*sum((yR-X'\*Beta).^2)/n+param.lambda.\*sum(abs(Beta))];  
   obj  
   if param.averaging\_mode  
      objav=[objav; 0.5\*sum((yR-X'\*tmp).^2)/n+param.lambda.\*sum(abs(tmp))];  
      objav  
   end  
   fprintf('Sparsity: \n');  
   sum(Beta ~= 0)  
end  
  
%%%%%%%%%%%%%%%%%%%%%%%%%%%%%%%%%%%%%  
% EXPERIMENT 2: L2 logistic regression   
%%%%%%%%%%%%%%%%%%%%%%%%%%%%%%%%%%%%%  
fprintf('EXPERIMENT FOR LOGISTIC REGRESSION + l2\n');  
y=sign(y);  
param.regul='l2';  
param.loss='logistic';  
obj=[];  
objav=[];  
for ii=1:length(tabepochs)  
   param.iters=tabepochs(ii)\*n;   % one pass over the data  
   fprintf('EXP WITH %d PASS\n',tabepochs(ii));  
   nlambdas=length(param.lambda);  
   Beta0=zeros(p,nlambdas);  
   tic  
   [Beta tmp]=mexStochasticProx(y,X,Beta0,param);  
   toc  
   yR=repmat(y,[1 nlambdas]);  
   fprintf('Objective functions: \n');  
   obj=[obj;sum(log(1.0+exp(-yR .\* (X'\*Beta))))/n+0.5\*param.lambda.\*sum(abs(Beta.^2))];  
   obj  
   if param.averaging\_mode  
      objav=[objav; sum(log(1.0+exp(-yR .\* (X'\*tmp))))/n+0.5\*param.lambda.\*sum(abs(tmp))];  
      objav  
   end  
end  
  
%%%%%%%%%%%%%%%%%%%%%%%%%%%%%%%%%%%%%  
% EXPERIMENT 3: L1 logistic regression   
%%%%%%%%%%%%%%%%%%%%%%%%%%%%%%%%%%%%%  
fprintf('EXPERIMENT FOR LOGISTIC REGRESSION + l1\n');  
y=sign(y);  
param.regul='l1';        % many other regularization functions are available  
param.loss='logistic';     % only square and log are available  
obj=[];  
objav=[];  
for ii=1:length(tabepochs)  
   param.iters=tabepochs(ii)\*n;   % one pass over the data  
   fprintf('EXP WITH %d PASS\n',tabepochs(ii));  
   nlambdas=length(param.lambda);  
   Beta0=zeros(p,nlambdas);  
   tic  
   [Beta tmp]=mexStochasticProx(y,X,Beta0,param);  
   toc  
   yR=repmat(y,[1 nlambdas]);  
   fprintf('Objective functions: \n');  
   obj=[obj; sum(log(1.0+exp(-yR .\* (X'\*Beta))))/n+param.lambda.\*sum(abs(Beta))];  
   obj  
   if param.averaging\_mode  
      objav=[objav; sum(log(1.0+exp(-yR .\* (X'\*tmp))))/n+param.lambda.\*sum(abs(tmp))];  
      objav  
   end  
   fprintf('Sparsity: \n');  
   sum(Beta ~= 0)  
end

## 6  A few Functions for manipulating images

This functions are not well documented yet

### 6.1  Function mexExtractPatches

%   
% Usage:   X =mexExtractPatches(I,n,step);  
%  
% Name: mexExtractPatches  
%  
% Description: Extract n x n patches spaced every ``step'' pixels from   
% an image I of size nx x ny x nchannels  
%  
% Inputs: I:  double nx x ny x nchannels     
%  
% Output: X: double dense matrix  
%  
% Author: Julien Mairal, 2014

### 6.2  Function mexCombinePatches

%   
% Usage:   I =mexCombinePatches(X,I0,n,step,lambda,normalize);  
%  
% Name: mexCombinePatches  
%  
% Description: Combine patches extracted with mexExtractPatches into a new image   
%              I = lambda I0 + combined(X) if normalize, averaging is  
%              performed; otherwise the patches are just summed.  
%  
% Inputs: I0:  double nx x ny x nchannels     
%  
% Output: I: double nx x ny x nchannels   
%  
% Author: Julien Mairal, 2014

### 6.3  Function mexConvFistaFlat

%   
% Usage:   alpha =mexConvFistaFlat(I,D,alpha0,param);  
%  
% Name: mexConvFistaFlat  
%  
% Description: performs convolutional sparse encoding of an  
%              image I with a local dictionary D, using FISTA  
%              and similar options as mexFistaFlat  
%  
% Inputs: I:  double nx x ny x nchannels     
%         D:  dictionary    
%         alpha0: initial weights    
%  
% Output: alpha: output coefficients  
%  
% Author: Julien Mairal, 2014

## 7  Miscellaneous Functions


### 7.1  Function mexConjGrad

Implementation of a conjugate gradient for solving a linear system Ax=b
when A is positive definite. In some cases, it is faster than the Matlab
function `pcg`, especially when the library uses the Intel Math Kernel Library.

%   
% Usage:   x =mexConjGrad(A,b,x0,tol,itermax)  
%  
% Name: mexConjGrad  
%  
% Description: Conjugate gradient algorithm, sometimes faster than the   
%    equivalent Matlab function pcg. In order to solve Ax=b;  
%  
% Inputs: A:  double square n x n matrix. HAS TO BE POSITIVE DEFINITE  
%         b:  double vector of length n.  
%         x0: double vector of length n. (optional) initial guess.  
%         tol: (optional) tolerance.  
%         itermax: (optional) maximum number of iterations.  
%  
% Output: x: double vector of length n.  
%  
% Author: Julien Mairal, 2009

The following piece of code illustrates how to use this function.

A=randn(5000,500);  
A=A'\*A;  
b=ones(500,1);  
x0=b;  
tol=1e-4;  
itermax=0.5\*length(b);  
  
tic  
for ii = 1:20  
x1 = mexConjGrad(A,b,x0,tol,itermax);  
end  
t=toc;  
fprintf('mex-file time: %fs\n',t);  
  
tic  
for ii = 1:20  
x2 = pcg(A,b);  
end  
t=toc;  
fprintf('Matlab time: %fs\n',t);  
sum((x1(:)-x2(:)).^2)

### 7.2  Function mexBayer

Apply a Bayer pattern to an input image

%   
% Usage:   x =mexConjGrad(A,b,x0,tol,itermax)  
%  
% Name: mexConjGrad  
%  
% Description: Conjugate gradient algorithm, sometimes faster than the   
%    equivalent Matlab function pcg. In order to solve Ax=b;  
%  
% Inputs: A:  double square n x n matrix. HAS TO BE POSITIVE DEFINITE  
%         b:  double vector of length n.  
%         x0: double vector of length n. (optional) initial guess.  
%         tol: (optional) tolerance.  
%         itermax: (optional) maximum number of iterations.  
%  
% Output: x: double vector of length n.  
%  
% Author: Julien Mairal, 2009

The following piece of code illustrates how to use this function.

A=randn(5000,500);  
A=A'\*A;  
b=ones(500,1);  
x0=b;  
tol=1e-4;  
itermax=0.5\*length(b);  
  
tic  
for ii = 1:20  
x1 = mexConjGrad(A,b,x0,tol,itermax);  
end  
t=toc;  
fprintf('mex-file time: %fs\n',t);  
  
tic  
for ii = 1:20  
x2 = pcg(A,b);  
end  
t=toc;  
fprintf('Matlab time: %fs\n',t);  
sum((x1(:)-x2(:)).^2)

### 7.3  Function mexCalcAAt

For an input sparse matrix A, this function returns AA^T^. For some reasons, when A has a lot more columns than rows, this function can be much faster than the equivalent matlab command `X*A'`.

%   
% Usage:   AAt =mexCalcAAt(A);  
%  
% Name: mexCalcAAt  
%  
% Description: Compute efficiently AAt = A\*A', when A is sparse   
%   and has a lot more columns than rows. In some cases, it is  
%   up to 20 times faster than the equivalent Matlab expression  
%   AAt=A\*A';  
%  
% Inputs: A:  double sparse m x n matrix     
%  
% Output: AAt: double m x m matrix   
%  
% Author: Julien Mairal, 2009

The following piece of code illustrates how to use this function.

A=sprand(200,200000,0.05);  
  
tic  
AAt=mexCalcAAt(A);  
t=toc;  
fprintf('mex-file time: %fs\n',t);  
  
tic  
AAt2=A\*A';  
t=toc;  
fprintf('matlab time: %fs\n',t);  
  
sum((AAt(:)-AAt2(:)).^2)

### 7.4  Function mexCalcXAt

For an input sparse matrix A and a matrix X, this function returns XA^T^. For some reasons, when A has a lot more columns than rows, this function can be much faster than the equivalent matlab command `X*A'`.

%   
% Usage:   XAt =mexCalcXAt(X,A);  
%  
% Name: mexCalcXAt  
%  
% Description: Compute efficiently XAt = X\*A', when A is sparse and has a   
%   lot more columns than rows. In some cases, it is up to 20 times   
%   faster than the equivalent Matlab expression XAt=X\*A';  
%  
% Inputs: X:  double m x n matrix  
%         A:  double sparse p x n matrix     
%  
% Output: XAt: double m x p matrix   
%  
% Author: Julien Mairal, 2009

The following piece of code illustrates how to use this function.

X=randn(64,200000);  
A=sprand(200,200000,0.05);  
  
tic  
XAt=mexCalcXAt(X,A);  
t=toc;  
fprintf('mex-file time: %fs\n',t);  
  
tic  
XAt2=X\*A';  
t=toc;  
fprintf('mex-file time: %fs\n',t);  
  
sum((XAt(:)-XAt2(:)).^2)

### 7.5  Function mexCalcXY

For two input matrices X and Y, this function returns XY.

%   
% Usage:   Z =mexCalcXY(X,Y);  
%  
% Name: mexCalcXY  
%  
% Description: Compute Z=XY using the BLAS library used by SPAMS.  
%  
% Inputs: X:  double m x n matrix  
%         Y:  double n x p matrix     
%  
% Output: Z: double m x p matrix   
%  
% Author: Julien Mairal, 2009

The following piece of code illustrates how to use this function.

X=randn(64,200);  
Y=randn(200,20000);  
  
tic  
XY=mexCalcXY(X,Y);  
t=toc;  
fprintf('mex-file time: %fs\n',t);  
  
tic  
XY2=X\*Y;  
t=toc;  
fprintf('mex-file time: %fs\n',t);  
  
sum((XY(:)-XY2(:)).^2)

### 7.6  Function mexCalcXYt

For two input matrices X and Y, this function returns XY^T^.

%   
% Usage:   Z =mexCalcXYt(X,Y);  
%  
% Name: mexCalcXYt  
%  
% Description: Compute Z=XY' using the BLAS library used by SPAMS.  
%  
% Inputs: X:  double m x n matrix  
%         Y:  double p x n matrix     
%  
% Output: Z: double m x p matrix   
%  
% Author: Julien Mairal, 2009

The following piece of code illustrates how to use this function.

X=randn(64,200);  
Y=randn(200,20000)';  
  
tic  
XYt=mexCalcXYt(X,Y);  
t=toc;  
fprintf('mex-file time: %fs\n',t);  
  
  
tic  
XYt2=X\*Y';  
t=toc;  
fprintf('matlab-file time: %fs\n',t);  
  
sum((XYt(:)-XYt2(:)).^2)

### 7.7  Function mexCalcXtY

For two input matrices X and Y, this function returns X^T^Y.

%   
% Usage:   Z =mexCalcXtY(X,Y);  
%  
% Name: mexCalcXtY  
%  
% Description: Compute Z=X'Y using the BLAS library used by SPAMS.  
%  
% Inputs: X:  double n x m matrix  
%         Y:  double n x p matrix     
%  
% Output: Z: double m x p matrix   
%  
% Author: Julien Mairal, 2009

The following piece of code illustrates how to use this function.

X=randn(64,200)';  
Y=randn(200,20000);  
  
tic  
XtY=mexCalcXtY(X,Y);  
t=toc;  
fprintf('mex-file time: %fs\n',t);  
  
tic  
XtY2=X'\*Y;  
t=toc;  
fprintf('matlab-file time: %fs\n',t);  
  
sum((XtY(:)-XtY2(:)).^2)

### 7.8  Function mexInvSym

For an input symmetric matrices A in ℝ^n × n^, this function returns A^−1^.

%   
% Usage:   B =mexInvSym(A);  
%  
% Name: mexInvSym  
%  
% Description: returns the inverse of a symmetric matrix A  
%  
% Inputs: A:  double n x n matrix     
%  
% Output: B: double n x n matrix   
%  
% Author: Julien Mairal, 2009

The following piece of code illustrates how to use this function.

A=rand(1000,1000);  
A=A'\*A;  
  
tic  
B=mexInvSym(A);  
t=toc;  
fprintf('mex-file time: %fs\n',t);  
  
tic  
B2=inv(A);  
t=toc;  
fprintf('matlab-file time: %fs\n',t);  
  
sum((B(:)-B2(:)).^2)

### 7.9  Function mexNormalize

%   
% Usage:   Y =mexNormalize(X);  
%  
% Name: mexNormalize  
%  
% Description: rescale the columns of X so that they have  
%        unit l2-norm.  
%  
% Inputs: X:  double m x n matrix     
%  
% Output: Y: double m x n matrix   
%  
% Author: Julien Mairal, 2010

The following piece of code illustrates how to use this function.

X=rand(100,1000);  
  
tic  
Y=mexNormalize(X);  
t=toc;

### 7.10  Function mexSort

%   
% Usage:   Y =mexSort(X);  
%  
% Name: mexSort  
%  
% Description: sort the elements of X using quicksort  
%  
% Inputs: X:  double vector of size n  
%  
% Output: Y: double  vector of size n  
%  
% Author: Julien Mairal, 2010

The following piece of code illustrates how to use this function.

X=rand(1,300000);  
  
tic  
Y=mexSort(X);  
t=toc;  
toc  
  
tic  
Y2=sort(X,'ascend');  
t=toc;  
toc  
  
sum((Y2(:)-Y(:)).^2)

### 7.11  Function mexDisplayPatches

Print to the screen a matrix containing as columns image patches.

### 7.12  Function mexCountPathsDAG

This function counts the number of paths in a DAG using dynamic programming.

%   
% Usage:   num=mexCountPathsDAG(G);  
%  
% Name: mexCountPathsDAG  
%  
% Description: mexCountPathsDAG counts the number of paths   
%       in a DAG.  
%  
%       for a graph G with |V| nodes and |E| arcs,  
%       G is a double sparse adjacency matrix of size |V|x|V|,  
%       with |E| non-zero entries.  
%       (see example in test\_CountPathsDAG.m  
%  
%  
% Inputs: G:  double sparse |V| x |V| matrix (full graph)  
%  
% Output: num: number of paths  
%  
% Author: Julien Mairal, 2012

The following piece of code illustrates how to use this function.

% graph corresponding to figure 1 in arXiv:1204.4539v1  
fprintf('test mexCountPathsDAG\n');  
% this graph is a DAG  
G=[0 0 0 0 0 0 0 0 0 0 0 0 0;  
   1 0 0 0 0 0 0 0 0 0 0 0 0;  
   1 1 0 0 0 0 0 0 0 0 0 0 0;  
   1 1 0 0 0 0 0 0 0 0 0 0 0;  
   0 0 0 1 0 0 0 0 0 0 0 0 0;  
   0 1 1 0 1 0 0 0 0 0 0 0 0;  
   0 1 0 0 1 0 0 0 0 0 0 0 0;  
   0 0 0 0 0 1 1 0 0 0 0 0 0;  
   1 0 0 1 0 0 0 0 0 0 0 0 0;  
   1 0 0 0 0 0 0 0 1 0 0 0 0;  
   0 0 0 0 0 0 0 0 1 1 0 0 0;  
   0 0 0 0 0 0 0 0 1 0 1 0 0;  
   0 0 0 0 1 0 0 0 1 0 0 1 0];  
G=sparse(G);  
num=mexCountPathsDAG(G);  
fprintf('Num of paths: %d\n',num);

### 7.13  Function mexRemoveCyclesGraph

One heuristic to remove cycles from a graph.

%   
% Usage:   DAG=mexRemoveCycleGraph(G);  
%  
% Name: mexRemoveCycleGraph  
%  
% Description: mexRemoveCycleGraph heuristically removes  
%       arcs along cycles in the graph G to obtain a DAG.  
%       the arcs of G can have weights. The heuristic will  
%       remove in priority arcs with the smallest weights.  
%  
%       for a graph G with |V| nodes and |E| arcs,  
%       G is a double sparse adjacency matrix of size |V|x|V|,  
%       with |E| non-zero entries. The non-zero entries correspond  
%       to the weights of the arcs.  
%  
%       DAG is a also double sparse adjacency matrix of size |V|x|V|,  
%       but the graph is acyclic.  
%  
%       Note that another heuristic to obtain a DAG from a general   
%       graph consists of ordering the vertices.  
%  
% Inputs: G:  double sparse |V| x |V| matrix  
%  
% Output: DAG:  double sparse |V| x |V| matrix  
%  
% Author: Julien Mairal, 2012

The following piece of code illustrates how to use this function.

fprintf('test mexRemoveCyclesGraph\n');  
% this graph is not a DAG  
G=[0   0   0   0   0   0   0   0   0   0   0   0   0;  
   1   0   0   0.1 0   0   0   0.1 0   0   0   0   0;  
   1   1   0   0   0   0.1 0   0   0   0   0   0   0;  
   1   1   0   0   0   0   0   0   0   0   0   0.1 0;  
   0   0   0   1   0   0   0   0   0   0   0   0   0;  
   0   1   1   0   1   0   0   0   0   0   0   0   0;  
   0   1   0   0   1   0   0   0   0   0   0   0   0;  
   0   0   0   0   0   1   1   0   0   0   0   0   0;  
   1   0   0   1   0   0   0   0   0   0   0   0   0;  
   1   0   0   0   0   0   0   0   1   0   0   0   0;  
   0   0   0   0   0   0   0   0   1   1   0   0   0;  
   0   0   0   0   0   0   0   0   1   0   1   0   0;  
   0   0   0   0   1   0   0   0   1   0   0   1   0];  
G=sparse(G);  
DAG=mexRemoveCyclesGraph(G);  
format compact;  
fprintf('Original graph:\n');  
full(G)  
fprintf('New graph:\n');  
full(DAG)

### 7.14  Function mexCountConnexComponents

Count the number of connected components of a subgraph from a graph.

%   
% Usage:   num=mexCountConnexComponents(G,N);  
%  
% Name: mexCountConnexComponents  
%  
% Description: mexCountConnexComponents counts the number of connected  
%       components of the subgraph of G corresponding to set of nodes  
%       in N. In other words, the subgraph of G by removing from G all  
%       the nodes which are not in N.  
%  
%       for a graph G with |V| nodes and |E| arcs,  
%       G is a double sparse adjacency matrix of size |V|x|V|,  
%       with |E| non-zero entries.  
%       (see example in test\_CountConnexComponents.m)  
%       N is a dense vector of size |V|. if  N[i] is non-zero,  
%       it means that the node i is selected.  
%  
%  
% Inputs: G:  double sparse |V| x |V| matrix (full graph)  
%         N:  double full |V| vector.  
%  
% Output: num: number of connected components.  
%  
% Author: Julien Mairal, 2012

The following piece of code illustrates how to use this function.

% graph corresponding to figure 1 in arXiv:1204.4539v1  
fprintf('test mexCountConnexComponents\n');  
% this graph is a DAG  
G=[0 0 0 0 0 0 0 0 0 0 0 0 0;  
   1 0 0 0 0 0 0 0 0 0 0 0 0;  
   1 1 0 0 0 0 0 0 0 0 0 0 0;  
   1 1 0 0 0 0 0 0 0 0 0 0 0;  
   0 0 0 1 0 0 0 0 0 0 0 0 0;  
   0 1 1 0 1 0 0 0 0 0 0 0 0;  
   0 1 0 0 1 0 0 0 0 0 0 0 0;  
   0 0 0 0 0 1 1 0 0 0 0 0 0;  
   1 0 0 1 0 0 0 0 0 0 0 0 0;  
   1 0 0 0 0 0 0 0 1 0 0 0 0;  
   0 0 0 0 0 0 0 0 1 1 0 0 0;  
   0 0 0 0 0 0 0 0 1 0 1 0 0;  
   0 0 0 0 1 0 0 0 1 0 0 1 0];  
G=sparse(G);  
nodes=[0 1 1 0 0 1 0 0 1 0 1 1 0];  
num=mexCountConnexComponents(G,nodes);  
fprintf('Num of connected components: %d\n',num);  
  
% this graph is a not a DAG anymore. This function works  
% with general graphs.  
G=[0 0 0 0 0 0 0 0 0 0 0 0 0;  
   1 0 0 0 0 0 0 0 0 0 0 0 0;  
   1 1 0 1 0 0 0 0 0 0 0 0 0;  
   1 1 0 0 0 0 0 0 0 0 0 0 0;  
   0 0 0 1 0 0 0 0 0 0 0 0 0;  
   0 1 1 0 1 0 0 0 0 0 0 0 0;  
   0 1 0 0 1 0 0 0 0 0 0 0 0;  
   0 0 0 0 0 1 1 0 0 0 0 0 0;  
   1 0 0 1 0 0 0 0 0 0 0 0 0;  
   1 0 0 0 0 0 0 0 1 0 0 0 0;  
   0 0 0 0 0 0 0 0 1 1 0 0 0;  
   0 0 0 0 0 0 0 0 1 0 1 0 0;  
   0 0 0 0 1 0 0 0 1 0 0 1 0];  
nodes=[0 1 1 0 0 1 0 0 1 0 1 1 0];  
G=sparse(G);  
num=mexCountConnexComponents(G,nodes);  
fprintf('Num of connected components: %d\n',num);  
  
nodes=[0 1 1 1 0 1 0 0 1 0 1 1 0];  
num=mexCountConnexComponents(G,nodes);  
fprintf('Num of connected components: %d\n',num);

### 7.15  Function mexGraphOfGroupStruct

%   
% Usage:   groups =mexGraphOfGroupStruct(gstruct)  
%  
% Name: mexGraphOfGroupStruct  
%  
% Description: converts a group structure into the graph structure  
%    used by mexProximalGraph, mexFistaGraph or mexStructTrainDL  
%  
% Inputs: gstruct: the structure of groups as a cell array, one element per node  
%     an element is itself a 4 elements cell array:  
%       nodeid (>= 0), weight (double), array of vars associated to the node,  
%       array of children (nodeis's)  
% Output: graph: struct (see documentation of mexProximalGraph)  
%  
% Author: Jean-Paul CHIEZE, 2012

### 7.16  Function mexGroupStructOfString

%   
% Usage:   gstruct =mexGroupStructOfString(s)  
%  
% Name: mexGroupStructOfString  
%  
% Description: decode a multi-line string describing "simply" the structure of groups  
%    of variables needed by mexProximalGraph, mexProximalTree, mexFistaGraph,  
%    mexFistaTree and mexStructTrainDL and builds the corresponding group structure.  
%    Each line describes a group of variables as a node of a tree.  
%    It has up to 4 fields separated by spaces:  
%        node-id node-weight [variables-list] -> children-list  
%    Let's define Ng = number of groups, and Nv = number of variables.  
%    node-id must be in the range (0 - Ng-1), and there must be Ng nodes  
%    weight is a float  
%    variables-list : a space separated list of integers, maybe empty,  
%         but '[' and '] must be present. Numbers in the range (0 - Nv-1)  
%    children-list : a space separated list of node-id's  
%        If the list is empty, '->' may be omitted.  
%    The data must obey some rules :   
%        - A group contains the variables of the corresponding node and of the whole subtree.  
%        - Variables attached to a node are those that are not int the subtree.  
%        - If the data destination is a Graph, there may be several independant trees,  
%          and a varibale may appear in several trees.  
%    If the destination is a Tree, there must be only one tree, the root node  
%    must have id == 0 and each variable must appear only once.  
%  
% Inputs: s:  the multi-lines string  
%  
% Output: groups: cell array, one element for each node  
%                an element is itsel a 4 elements cell array:  
%                 nodeid (int >= 0), weight (double), array of vars of the node,  
%                array of children (nodeid's)  
%  
% Author: Jean-Paul CHIEZE, 2012

### 7.17  Function mexReadGroupStruct

%   
% Usage:   gstruct =mexReadGroupStruct(file)  
%  
% Name: mexReadGroupStruct  
%  
% Description: reads a text file describing "simply" the structure of groups  
%    of variables needed by mexProximalGraph, mexProximalTree, mexFistaGraph,  
%    mexFistaTree and mexStructTrainDL and builds the corresponding group structure.  
%    weight is a float  
%    variables-list : a space separated list of integers, maybe empty,  
%        but '[' and '] must be present. Numbers in the range (0 - Nv-1)  
%    children-list : a space separated list of node-id's  
%        If the list is empty, '->' may be omitted.  
%    The data must obey some rules :   
%        - A group contains the variables of the corresponding node and of the whole subtree.  
%        - Variables attached to a node are those that are not int the subtree.  
%        - If the data destination is a Graph, there may be several independant trees,  
%           and a varibale may appear in several trees.  
%    If the destination is a Tree, there must be only one tree, the root node  
%        must have id == 0 and each variable must appear only once.  
%  
% Inputs: file:  the file name  
%  
% Output: groups: cell array, one element for each node  
%                an element is itsel a 4 elements cell array:  
%                nodeid (int >= 0), weight (double), array of vars of the node,  
%                array of children (nodeid's)  
%  
% Author: Jean-Paul CHIEZE, 2012

### 7.18  Function mexTreeOfGroupStruct

%   
% Usage:   [permutations groups nbvars] =mexTreeOfGroupStruct(gstruct)  
%  
% Name: mexTreeOfGroupStruct  
%  
% Description: converts a group structure into the tree structure  
%    used by mexProximalTree, mexFistaTree or mexStructTrainDL  
%  
% Inputs: gstruct: the structure of groups as a cell array, one element per node  
%     an element is itself a 4 lements cell array:  
%       nodeid (>= 0), weight (double), array of vars associated to the node,  
%       array of children (nodeis's)  
% Output: permutations: permutation vector that must be applied to the result of the  
%               programm using the tree. Empty if no permutation is needed.  
%      tree: struct (see documentation of mexProximalTree)  
%      nbvars : number of variables in the tree  
%  
% Author: Jean-Paul CHIEZE, 2012

### 7.19  Function mexSimpleGroupTree

%   
% Usage:   gstruct =mexSimpleGroupTree(degrees)  
%  
% Name: mexSimpleGroupTree  
%  
% Description: makes a structure representing a tree given the  
%   degree of each level.  
%  
% Inputs: degrees:  int vector; degrees(i) is the number of children of each node at level i  
%  
% Output: group\_struct: cell array, one element for each node  
%                an element is itsel a 4 elements cell array :  
%                 nodeid (int >= 0), weight (double), array of vars attached to the node  
%                  (here equal to [nodeid]), array of children (nodeid's)  
%  
% Author: Jean-Paul CHIEZE, 2012

## A  Duality Gaps with Fenchel Duality

This section is taken from the appendix D of Julien Mairal’s PhD thesis [19].
We are going to use intensively Fenchel Duality (see [2]).
Let us consider the problem

|  |  |  |  |  |
| --- | --- | --- | --- | --- |
|  | min; w ∈ ℝ^p^ | [g(w) | ▵; = | f(w) + λψ(w)],     (47) |

We first notice that for all the formulations we have been
interested in, g(w) can be rewritten

g(w) = f(X^⊤^w) + λψ(w), 
    (48)

where X=[x^1^,…,x^n^] are training vectors, and f is an
appropriated smooth real-valued function of ℝ^n^,
and ψ one of the regularization functions we have introduced.

Given a primal variable w in ℝ^p^ and a dual variable κ in
ℝ^n^, we obtain using classical Fenchel duality rules [2],
that the following quantity is a duality gap for problem (47):

|  |  |  |
| --- | --- | --- |
| δ(w,κ) | ▵; = | g(w) + f^∗^(κ) + λψ^∗^(−Xκ / λ), |

where f^∗^ and ψ^∗^ are respectively the Fenchel conjugates
of f and ψ. Denoting by w^⋆^ the solution of
Eq. (47), the duality gap is interesting in the sense that
it upperbounds the difference with the optimal value of the function:

δ(w,κ) ≥  g(w)−g(w^⋆^) ≥ 0.

Similarly, we will consider pairs of primal-dual variables (W,K) when
dealing with matrices.

During the optimization, sequences of primal variables w are available,
and one wishes to exploit duality gaps for estimating the difference
g(w)−g(w^⋆^). This requires the following components:

- being able to efficiently compute f^∗^ and ψ^∗^.
- being able to obtain a “good” dual variable κ given a primal
  variable w, such that δ(w,κ) is close to
  g(w)−g(w^⋆^).

We suppose that the first point is satisfied (we will detail these computations
for every loss and regularization functions in the sequel), and explain how to
choose κ in general (details will also be given in the sequel).

Let us first consider the choice that associates with a primal variable w, the
dual variable

|  |  |  |
| --- | --- | --- |
| κ(w) | ▵; = | ∇ f(X^⊤^w),      (49) |

and let us compute δ(w,κ(w)).
First, easy computations show that for all vectors z in ℝ^n^,
f^∗^(∇f(z)) = z^⊤^∇f(z)−f(z),
which gives

|  |  |  |  |
| --- | --- | --- | --- |
| δ(w,κ(w)) | = | f(X^⊤^w) + λ ψ(w) + f^∗^(∇f(X^⊤^w)) + λψ^∗^(−X∇f(X^⊤^w)/ λ), | (50) |
|  | = | λ ψ(w) + w^⊤^X∇f(X^⊤^w) + λψ^∗^(−X∇f(X^⊤^w)/ λ). | (51) |

We now use the classical Fenchel-Young inequality (see, Proposition
3.3.4 of [2]) on the function ψ, which gives

δ(w,κ(w))  ≥  w^⊤^X∇f(X^⊤^w) − w^⊤^X∇f(X^⊤^w) = 0,

with equality if and only if −X∇f(X^⊤^w) belongs to
∂ ψ(w). Interestingly, we now that first-order optimality
conditions for Eq. (48) gives that
−X∇f(X^⊤^w^⋆^) ∈ ∂ ψ(w^⋆^).
We have now in hand a non-negative function w ↦ δ(w,κ(w)) of w, that
upperbounds g(w)−g(w^⋆^) and satisfying δ(w^⋆^,κ(w^⋆^))=0.

This is however not a sufficient property to make it a good measure
of the quality of the optimization, and further work is required,
that will be dependent on f and ψ.
We have indeed proven that δ(w^⋆^,κ(w^⋆^)) is always 0. However,
for w different than w^⋆^, δ(w^⋆^,κ(w^⋆^)) can be infinite,
making it a non-informative duality-gap. The reasons for this can be one of the following:

- The term ψ^∗^(−X∇f(X^⊤^w)/ λ) might have an infinite value.
- Intercepts make the problem more complicated. One can write the formulation with an intercept by
  adding a row to X filled with the value 1, add one dimension to the vector w, and consider
  a regularization function ψ that does regularize the last entry of
  w. This further complexifies the computation of ψ^∗^ and its
  definition, as shown in the next section.

Let us now detail how we proceed to solve these problems, but first without considering the intercept.
The analysis is similar when working with matrices W instead of vectors w.

#### A.0.1  Duality Gaps without Intercepts

Let us show how to compute the Fenchel conjugate of the functions we have introduced.
We now present the Fenchel conjugate of the loss functions f.

- The square loss

  |  |  |  |
  | --- | --- | --- |
  | f(z)= | 1; 2n | \|\|y−z\|\|_2_^2^, |

  |  |  |  |
  | --- | --- | --- |
  | f^∗^(κ)= | n; 2 | \|\|κ\|\|_2_^2^ + κ^⊤^y. |
- The logistic loss

  |  |  |  |  |
  | --- | --- | --- | --- |
  | f(z)= | 1; n | n; ∑; i=1 | log(1+e^−y_i_z_i_^) |

  f^∗^(κ)=

  ⎧  
  ⎪  
  ⎪  
  ⎨  
  ⎪  
  ⎪  
  ⎩

  +∞  if  ∃ i∈ [ 1;n ]   s.t.   y_i_κ_i_ ∉ [−1,0],

  n

  ∑

  i=1

  (1+y_i_κ_i_)log(1+y_i_κ_i_)−y_i_κ_i_log(−y_i_κ_i_)  otherwise.
- The multiclass logistic loss (or softmax). The primal variable is now a matrix Z, in ℝ^n × r^, which represents the product X^⊤^W. We denote by K the dual variable in ℝ^n × r^.

  |  |  |  |  |  |  |  |  |
  | --- | --- | --- | --- | --- | --- | --- | --- |
  | f(Z)= | 1; n | n; ∑; i=1 | log | ⎛  ⎜  ⎝ | r; ∑; j=1 | e ^Z_ij_ − Z_iyi_^ | ⎞  ⎟  ⎠ |

  f^∗^(K)=

  ⎧  
  ⎪  
  ⎪  
  ⎨  
  ⎪  
  ⎪  
  ⎩

  +∞  if  ∃ i ∈[ 1;n ]   s.t.   { K_ij_ < 0  and  j ≠ y_i_ }  or  K_iyi_ < −1,

  |  |  |  |  |  |  |  |  |
  | --- | --- | --- | --- | --- | --- | --- | --- |
  |  | n; ∑; i=1 |  | ⎡  ⎢  ⎣ | ∑; j ≠ y_i_ | K_ij_log(K_ij_) + (1+K_i yi_)log(1+K_i yi_) | ⎤  ⎥  ⎦ | . |

Our first remark is that the choice Eq. (49) ensures
that f(κ) is not infinite.

As for the regularization function, except for the Tikhonov regularization
which is self-conjugate (it is equal to its Fenchel conjugate), we have
considered functions that are norms. There exists therefore a norm ||.|| such
that ψ(w)=||w||, and we denote by ||.||_∗_ its dual-norm. In such a
case, the Fenchel conjugate of ψ for a vector γ in ℝ^p^ takes the
form

ψ^∗^(γ) =

⎧  
⎨  
⎩

|  |  |
| --- | --- |
| 0 | if  \|\|γ\|\|_∗_≤ 1, |
| +∞ | otherwise. |

It turns out that for almost all the norms we have presented, there exists (i)
either a closed form for the dual-norm or (ii) there exists an
efficient algorithm evaluating it. The only one which does not conform to this
statement is the tree-structured sum of ℓ_2_-norms, for which we do not know
how to evaluate it efficiently.

One can now slightly modify the definition of κ
to ensure that ψ^∗^(−Xκ/λ) ≠
+∞. A natural choice is

|  |  |  |  |  |  |  |  |
| --- | --- | --- | --- | --- | --- | --- | --- |
| κ(w) | ▵; = | min | ⎛  ⎜  ⎝ | 1, | λ; \|\|X∇f(X^⊤^w)\|\|_∗_ | ⎞  ⎟  ⎠ | ∇ f(X^⊤^w), |

which is the one we have implemented. With this new choice, it is easy to see
that for all vectors w in ℝ^p^, we still have f^∗^(κ) ≠ + ∞, and
finally, we also have δ(w,κ(w)) < + ∞ and
δ(w^⋆^,κ(w^⋆^))=0, making it potentially a good
duality gap.

#### A.0.2  Duality Gaps with Intercepts

Even though adding an intercept does seem a simple modification to the original
problem, it induces difficulties for finding good dual variables.

We recall that having an intercept is equivalent to having a problem of the
type (48), by adding a row to X filled with the value
1, adding one dimension to the vector w (or one row for matrices W),
and by using a regularization function that does not depend on the last entry
of w (or the last row of W).

Suppose that we are considering a problem of
type (48) of dimension p+1, but we are using a
regularization function ψ: ℝ^p+1^ → ℝ, such that for a
vector w in ℝ^p+1^, ψ(w) =^▵^ ψ(w_[ 1;p ]_),
where ψ: ℝ^p^ → ℝ is one of the regularization function we have
introduced. Then, considering a primal variable w, a dual variable κ,
and writing γ=^▵^−Xκ/λ, we are interested in computing

ψ^∗^(γ) =

⎧  
⎨  
⎩

+∞  if  γ_p+1_ ≠ 0

ψ^∗^(γ_[ 1;p ]_)  otherwise,

which means that in order the duality gap not to be infinite, one needs in addition to ensure
that γ_p+1_ be zero. Since the last row of X is filled with ones, this writes
down ∑_i=1_^p+1^ κ_i_=0.
For the formulation with matrices W and K, the constraint is similar but for every
column of K.

Let us now detail how we proceed for every loss function to find a “good”
dual variable κ satisfying this additional constraint, given a primal
variable w in ℝ^p+1^, we first define the auxiliary function

|  |  |  |
| --- | --- | --- |
| κ′(w) | ▵; = | ∇f(X^⊤^w), |

(which becomes K′(W)=^▵^ ∇f(X^⊤^W) for matrices),
and then define another auxiliary function κ″(w) as follows,
to take into account the additional constraint ∑_i=1_^p+1^ κ_i_=0.

- For the square loss, we define another auxiliary function:

  |  |  |  |  |  |
  | --- | --- | --- | --- | --- |
  | κ″(w) | ▵; = | κ′(w) − | 1; n | 1_p+1_^⊤^κ′(w)1_p+1_ |

  where 1_p+1_ is a vector of size p+1 filled with ones. This step,
  ensures that ∑_i=1_^p+1^κ″(w)_i_= 0.
- For the logistic loss, the situation is slightly more complicated since additional constraints are involved in the definition of f^∗^.

  |  |  |  |  |  |
  | --- | --- | --- | --- | --- |
  | κ″(w) | ▵; = | arg min_κ ∈ ℝ^n^_ \|\|κ−κ′(w)\|\|_2_^2^   s.t. | n; ∑; i=1 | κ_i_=0  and  ∀ i ∈ [ 1;n ], κ_i_ ∈ [−1,0]. |

  This problem can be solved in linear-time [3]
  using a similar algorithm as for the projection onto the ℓ_1_-ball,
  since it is an instance of a *quadratic knapsack problem*.
- For the multi-class logistic loss, we proceed in a similar way, for every column K^j^ of K, j ∈ [ 1;r ]:

  |  |  |  |  |  |
  | --- | --- | --- | --- | --- |
  | K^′′ j^(w) | ▵; = | arg min_κ ∈ ℝ^n^_ \|\|K^′ j^−κ′(w)\|\|_2_^2^   s.t. | n; ∑; i=1 | κ_i_=0  and |

  ∀ i ∈ [ 1;n ], {κ_i_ ≥ 0  if  j ≠ y_i_}  and  {κ_i_ ≥ −1  if  y_i_=j}.

When the function ψ is the Tykhonov regularization function, we end the process by setting κ(w)=κ″(w).
When it is a norm, we choose, as before for taking into account the constraint ||Xκ||_∗_≤ λ,

|  |  |  |  |  |  |  |  |
| --- | --- | --- | --- | --- | --- | --- | --- |
| κ(w) | ▵; = | min | ⎛  ⎜  ⎝ | 1, | λ; \|\|Xκ″(w)\|\|_∗_ | ⎞  ⎟  ⎠ | κ″(w), |

with a similar formulation for matrices W and K.

Even though finding dual variables while taking into account the intercept
requires quite a lot of engineering, notably implementing a quadratic knapsack
solver, it can be done efficiently.

## References

[1]
:   A. Beck and M. Teboulle.
    A fast iterative shrinkage-thresholding algorithm for linear inverse
    problems.
    *SIAM Journal on Imaging Sciences*, 2(1):183–202, 2009.

[2]
:   J. M. Borwein and A. S. Lewis.
    *Convex analysis and nonlinear optimization: Theory and
    examples*.
    Springer, 2006.

[3]
:   P. Brucker.
    An O(n) algorithm for quadratic knapsack problems.
    3:163–166, 1984.

[4]
:   E. J. Candès, M. Wakin, and S. Boyd.
    Enhancing sparsity by reweighted l1 minimization.
    *Journal of Fourier Analysis and Applications*, 14:877–905,
    2008.

[5]
:   B. V. Cherkassky and A. V. Goldberg.
    On implementing the push-relabel method for the maximum flow problem.
    *Algorithmica*, 19(4):390–410, 1997.

[6]
:   S. F. Cotter, J. Adler, B. Rao, and K. Kreutz-Delgado.
    Forward sequential algorithms for best basis selection.
    In *IEEE Proceedings of Vision Image and Signal Processing*,
    pages 235–244, 1999.

[7]
:   A. Cutler and L. Breiman.
    Archetypal analysis.
    *Technometrics*, 36(4):338–347, 1994.

[8]
:   J. Duchi, S. Shalev-Shwartz, Y. Singer, and T. Chandra.
    Efficient projections onto the ℓ_1_-ball for learning in high
    dimensions.
    In *Proceedings of the International Conference on Machine
    Learning (ICML)*, 2008.

[9]
:   B. Efron, T. Hastie, I. Johnstone, and R. Tibshirani.
    Least angle regression.
    *Annals of statistics*, 32(2):407–499, 2004.

[10]
:   J. Friedman, T. Hastie, H. Hölfling, and R. Tibshirani.
    Pathwise coordinate optimization.
    *Annals of statistics*, 1(2):302–332, 2007.

[11]
:   J. Friedman, T. Hastie, and R. Tibshirani.
    A note on the group lasso and a sparse group lasso.
    Technical report, Preprint arXiv:1001.0736, 2010.

[12]
:   W. J. Fu.
    Penalized regressions: The bridge versus the Lasso.
    *Journal of computational and graphical statistics*, 7:397–416,
    1998.

[13]
:   A. V. Goldberg and R. E. Tarjan.
    A new approach to the maximum flow problem.
    In *Proc. of ACM Symposium on Theory of Computing*, pages
    136–146, 1986.

[14]
:   P. O. Hoyer.
    Non-negative sparse coding.
    In *Proc. IEEE Workshop on Neural Networks for Signal
    Processing*, 2002.

[15]
:   R. Jenatton, J. Mairal, G. Obozinski, and F. Bach.
    Proximal methods for sparse hierarchical dictionary learning.
    In *Proceedings of the International Conference on Machine
    Learning (ICML)*, 2010.

[16]
:   R. Jenatton, J. Mairal, G. Obozinski, and F. Bach.
    Proximal methods for hierarchical sparse coding.
    *Journal of Machine Learning Research*, 12:2297–2334, 2011.

[17]
:   D. D. Lee and H. S. Seung.
    Algorithms for non-negative matrix factorization.
    In *Advances in Neural Information Processing Systems*, 2001.

[18]
:   N. Maculan and J. R. G. Galdino de Paula.
    A linear-time median-finding algorithm for projecting a vector on the
    simplex of Rn.
    *Operations research letters*, 8(4):219–222, 1989.

[19]
:   J. Mairal.
    *Sparse coding for machine learning, image processing and
    computer vision*.
    PhD thesis, Ecole Normale Supérieure, Cachan, 2010.

[20]
:   J. Mairal, F. Bach, J. Ponce, and G. Sapiro.
    Online dictionary learning for sparse coding.
    In *Proceedings of the International Conference on Machine
    Learning (ICML)*, 2009.

[21]
:   J. Mairal, F. Bach, J. Ponce, and G. Sapiro.
    Online learning for matrix factorization and sparse coding.
    *Journal of Machine Learning Research*, 11:19–60, 2010.

[22]
:   J. Mairal, R. Jenatton, G. Obozinski, and F. Bach.
    Network flow algorithms for structured sparsity.
    In *Advances in Neural Information Processing Systems*, 2010.

[23]
:   J. Mairal, R. Jenatton, G. Obozinski, and F. Bach.
    Convex and network flow optimization for structured sparsity.
    *Journal of Machine Learning Research*, 12:2649–2689, 2011.

[24]
:   J. Mairal and B. Yu.
    Supervised feature selection in graphs with path coding penalties and
    network flows.
    *Journal of Machine Learning Research*, 2013.

[25]
:   Julien Mairal.
    Optimization with first-order surrogate functions.
    In *International Conference on Machine Learning (ICML)*, 2013.

[26]
:   Julien Mairal.
    Stochastic majorization-minimization algorithms for large-scale
    optimization.
    In *Advances in Neural Information Processing Systems (NIPS)*,
    2013.

[27]
:   S. Mallat and Z. Zhang.
    Matching pursuit in a time-frequency dictionary.
    *IEEE Transactions on Signal Processing*, 41(12):3397–3415,
    1993.

[28]
:   N. Meinshausen and P. Buehlmann.
    Stability selection.
    Technical report.
    ArXiv:0809.2932.

[29]
:   G. Obozinski, B. Taskar, and M.I. Jordan.
    Joint covariate selection and joint subspace selection for multiple
    classification problems.
    *Statistics and Computing*, pages 1–22.

[30]
:   M. R. Osborne, B. Presnell, and B. A. Turlach.
    On the Lasso and its dual.
    *Journal of Computational and Graphical Statistics*,
    9(2):319–37, 2000.

[31]
:   P. Sprechmann, I. Ramirez, G. Sapiro, and Y. C. Eldar.
    Collaborative hierarchical sparse modeling.
    Technical report, 2010.
    Preprint arXiv:1003.0400v1.

[32]
:   R. Tibshirani, M. Saunders, S. Rosset, J. Zhu, and K. Knight.
    Sparsity and smoothness via the fused lasso.
    *Journal of the Royal Statistical Society Series B*,
    67(1):91–108, 2005.

[33]
:   J. A. Tropp.
    Algorithms for simultaneous sparse approximation. part ii: Convex
    relaxation.
    *Signal Processing, special issue "Sparse approximations in
    signal and image processing"*, 86:589–602, April 2006.

[34]
:   J. A. Tropp, A. C. Gilbert, and M. J. Strauss.
    Algorithms for simultaneous sparse approximation. part i: Greedy
    pursuit.
    *Signal Processing, special issue "sparse approximations in
    signal and image processing"*, 86:572–588, April 2006.

[35]
:   S. Weisberg.
    *Applied Linear Regression*.
    Wiley, New York, 1980.

[36]
:   T. T. Wu and K. Lange.
    Coordinate descent algorithms for Lasso penalized regression.
    *Annals of Applied Statistics*, 2(1):224–244, 2008.

[37]
:   J. Mairal Y. Chen and Z. Harchaoui.
    Fast and robust archetypal analysis for representation learning.
    In *Proceedings of the IEEE Conference on Computer Vision and
    Pattern Recognition (CVPR)*, 2014.

[38]
:   M. Yuan and Y. Lin.
    Model selection and estimation in regression with grouped variables.
    *Journal of the Royal Statistical Society Series B*, 68:49–67,
    2006.

[39]
:   H. Zou and T. Hastie.
    Regularization and variable selection via the elastic net.
    *Journal of the Royal Statistical Society Series B*,
    67(2):301–320, 2005.


---

> *This document was translated from L^A^T_E_X by* *H^E^V^E^A**.*
