# Supplementary material for: Multitask fMRI Data Classification via Group-Wise Hybrid Temporal and Spatial Sparse Representations
Source: eNeuro. 2022 Jun 3;9(3):ENEURO.0478-21.2022. doi: 10.1523/ENEURO.0478-21.2022 (PMC9186416; doi:10.1523/ENEURO.0478-21.2022)
Supplement: Extended Data 1 — The code. Download Extended Data 1, ZIP file. [file enu-eN-MNT-0478-21-s02.zip › code/tools/spams-matlab.zip!/spams-matlab/doc/html/doc_spams001.html]

Contents


---

## Contents

- 1  Introduction
- 2  Installation
- 3  Dictionary Learning and Matrix Factorization Toolbox
  - 3.1  Function mexTrainDL
  - 3.2  Function mexTrainDL\_Memory
  - 3.3  Function mexStructTrainDL
  - 3.4  Function nmf
  - 3.5  Function nnsc
  - 3.6  Function mexArchetypalAnalysis
- 4  Sparse Decomposition Toolbox
  - 4.1  Function mexOMP
  - 4.2  Function mexOMPMask
  - 4.3  Function mexRidgeRegression
  - 4.4  Function mexLasso
  - 4.5  Function mexLassoWeighted
  - 4.6  Function mexLassoMask
  - 4.7  Function mexCD
  - 4.8  Function mexSOMP
  - 4.9  Function mexL1L2BCD
  - 4.10  Function mexSparseProject
  - 4.11  Function mexDecompSimplex
- 5  Proximal Toolbox
  - 5.1  Regularization Functions
  - 5.2  Function mexProximalFlat
  - 5.3  Function mexProximalTree
  - 5.4  Function mexProximalGraph
  - 5.5  Function mexProximalPathCoding
  - 5.6  Function mexEvalPathCoding
  - 5.7  Problems Addressed
    - 5.7.1  Regression Problems with the Square Loss
    - 5.7.2  Classification Problems with the Logistic Loss
    - 5.7.3  Multi-class Classification Problems with the Softmax Loss
    - 5.7.4  Multi-task Regression Problems with the Square Loss
    - 5.7.5  Multi-task Classification Problems with the Logistic Loss
    - 5.7.6  Multi-task and Multi-class Classification Problems with the Softmax Loss
  - 5.8  Function mexFistaFlat
  - 5.9  Function mexFistaTree
  - 5.10  Function mexFistaGraph
  - 5.11  Function mexFistaPathCoding
  - 5.12  Function solverPoisson
  - 5.13  Function mexIncrementalProx
  - 5.14  Function mexStochasticProx
- 6  A few Functions for manipulating images
  - 6.1  Function mexExtractPatches
  - 6.2  Function mexCombinePatches
  - 6.3  Function mexConvFistaFlat
- 7  Miscellaneous Functions
  - 7.1  Function mexConjGrad
  - 7.2  Function mexBayer
  - 7.3  Function mexCalcAAt
  - 7.4  Function mexCalcXAt
  - 7.5  Function mexCalcXY
  - 7.6  Function mexCalcXYt
  - 7.7  Function mexCalcXtY
  - 7.8  Function mexInvSym
  - 7.9  Function mexNormalize
  - 7.10  Function mexSort
  - 7.11  Function mexDisplayPatches
  - 7.12  Function mexCountPathsDAG
  - 7.13  Function mexRemoveCyclesGraph
  - 7.14  Function mexCountConnexComponents
  - 7.15  Function mexGraphOfGroupStruct
  - 7.16  Function mexGroupStructOfString
  - 7.17  Function mexReadGroupStruct
  - 7.18  Function mexTreeOfGroupStruct
  - 7.19  Function mexSimpleGroupTree
- A  Duality Gaps with Fenchel Duality

  - A.0.1  Duality Gaps without Intercepts
  - A.0.2  Duality Gaps with Intercepts

---
