# Supplementary material for: Multitask fMRI Data Classification via Group-Wise Hybrid Temporal and Spatial Sparse Representations
Source: eNeuro. 2022 Jun 3;9(3):ENEURO.0478-21.2022. doi: 10.1523/ENEURO.0478-21.2022 (PMC9186416; doi:10.1523/ENEURO.0478-21.2022)
Supplement: Extended Data 1 — The code. Download Extended Data 1, ZIP file. [file enu-eN-MNT-0478-21-s02.zip › code/tools/spams-matlab.zip!/spams-matlab/doc/html/doc_spams002.html]

Introduction


---

## 1  Introduction

SPAMS (SPArse Modeling Software) is an open-source optimization toolbox for
sparse estimation with licence GPLv3. It implements algorithms for solving
machine learning and signal processing problems involving sparse
regularizations.

The library is coded in C++, is compatible with Linux, Mac, and Windows 32bits
and 64bits Operating Systems. It is interfaced with Matlab, R and Python, but
can be called from any C++ application (by hacking the code a bit).

It requires an implementation of BLAS and LAPACK for performing linear algebra
operations. The ones shipped with matlab and R can be used, but also external
libraries such as atlas, the netlib implementation, or the Intel Math Kernel
Library can be used. It also exploits multi-core CPUs when this feature is
supported by the compiler, through OpenMP.

The current licence is GPLv3, which is available at
http://www.gnu.org/licenses/gpl.html. For other licensing possibilities
allowing its use in proprietary softwares, please contact the author.

Version 2.5 of SPAMS is divided into several “toolboxes” and has a few
additional miscellaneous functions:

- The Dictionary learning and matrix factorization toolbox
  contains the online learning technique of [20, 21] and its
  variants for solving various matrix factorization problems:
  - dictionary Learning for sparse coding;
  - sparse principal component analysis (seen as a sparse matrix factorization problem);
  - non-negative matrix factorization;
  - non-negative sparse coding;
  - dictionary learning with structured sparsity;
  - archetypal analysis [7, 37].
- The Sparse decomposition toolbox contains efficient implementations of
  - Orthogonal Matching Pursuit, (or Forward Selection) [35, 27];
  - the LARS/homotopy algorithm [30, 9] (variants for solving Lasso and Elastic-Net problems);
  - a weighted version of LARS;
  - OMP and LARS when data comes with a binary mask;
  - a coordinate-descent algorithm for ℓ_1_-decomposition problems [12, 10, 36];
  - a greedy solver for simultaneous signal approximation as defined in [34, 33] (SOMP);
  - a solver for simulatneous signal approximation with ℓ_1_/ℓ_2_-regularization based on block-coordinate descent;
  - a homotopy method for the Fused-Lasso Signal Approximation as defined in [10] with the homotopy method presented in the appendix of [21];
  - a tool for projecting efficiently onto a few convex sets
    inducing sparsity such as the ℓ_1_-ball using the method of
    [3, 18, 8], and Elastic-Net or Fused Lasso constraint sets as
    proposed in the appendix of [21].
  - an active-set algorithm for simplex decomposition problems [37].
- The Proximal toolbox: An implementation of proximal methods
  (ISTA and FISTA [1]) for solving a large class of sparse approximation
  problems with different combinations of loss and regularizations. One of the main
  features of this toolbox is to provide a robust stopping criterion based on
  *duality gaps* to control the quality of the optimization, whenever
  possible. It also handles sparse feature matrices for large-scale problems. The following regularizations are implemented:
  - Tikhonov regularization (squared ℓ_2_-norm);
  - ℓ_1_-norm, ℓ_2_, ℓ_∞_-norms;
  - Elastic-Net [39];
  - Fused Lasso [32];
  - tree-structured sum of ℓ_2_-norms (see [15, 16]);
  - tree-structured sum of ℓ_∞_-norms (see [15, 16]);
  - general sum of ℓ_∞_-norms (see [22, 23]);
  - mixed ℓ_1_/ℓ_2_-norms on matrices [38, 29];
  - mixed ℓ_1_/ℓ_∞_-norms on matrices [38, 29];
  - mixed ℓ_1_/ℓ_2_-norms on matrices plus ℓ_1_ [31, 11];
  - mixed ℓ_1_/ℓ_∞_-norms on matrices plus ℓ_1_;
  - group-lasso with ℓ_2_ or ℓ_∞_-norms;
  - group-lasso+ℓ_1_;
  - multi-task tree-structured sum of ℓ_∞_-norms (see [22, 23]);
  - trace norm;
  - ℓ_0_ pseudo-norm (only with ISTA);
  - tree-structured ℓ_0_ (only with ISTA);
  - rank regularization for matrices (only with ISTA);
  - the path-coding penalties of [24].
  All of these regularization functions can be used with the following losses
  - square loss;
  - square loss with missing observations;
  - logistic loss, weighted logistic loss;
  - multi-class logistic.
  This toolbox can also enforce non-negativity constraints, handle intercepts and
  sparse matrices. There are also a few additional undocumented functionalities,
  which are available in the source code.
  For some combinations of loss and regularizers, stochastic and incremental proximal
  gradient solvers are also implemented [26, 25].
- A few tools for performing linear algebra operations such as a
  conjugate gradient algorithm, manipulating sparse matrices and graphs.

The toolbox was written by Julien Mairal at INRIA, with the collaboration of
Francis Bach (INRIA), Jean Ponce (Ecole Normale Supérieure), Guillermo Sapiro
(University of Minnesota), Guillaume Obozinski (INRIA) and Rodolphe Jenatton
(INRIA).

R and Python interfaces have been written by Jean-Paul Chieze (INRIA).
The archetypal analysis implementation was written by Yuansi Chen, during
an internship at INRIA, with the collaboration of Zaid Harchaoui.

---
