# Supplementary material for: Multitask fMRI Data Classification via Group-Wise Hybrid Temporal and Spatial Sparse Representations
Source: eNeuro. 2022 Jun 3;9(3):ENEURO.0478-21.2022. doi: 10.1523/ENEURO.0478-21.2022 (PMC9186416; doi:10.1523/ENEURO.0478-21.2022)
Supplement: Extended Data 1 — The code. Download Extended Data 1, ZIP file. [file enu-eN-MNT-0478-21-s02.zip › code/tools/spams-matlab.zip!/spams-matlab/doc/html/doc_spams003.html]

Installation


---

## 2  Installation

The toolbox used to come with pre-compiled binaries for various plateforms.
Now the sources are available and you will have to compile it yourself.

The user has the choice of the BLAS library, but the Intel MKL is recommended
for the best performance. Note that the builtin blas library of Matlab is a
version of the Intel MKL (not the most recent one though), and offers
a good performance.

The folder `doc` contains the documentation in pdf and html. `build/`
contains the binary files, including the help for each command.
`test_release` contains various matlab scripts which call the different
functions of this toolbox.

The software package comes also with a script bash that has to be used to
launch matlab for Linux and/or Mac OS versions. The install procedure is
described in a file called `HOW_TO_INSTALL` and in the file
`compile.m` for the Matlab version.

---
