# Supplementary material for: Multitask fMRI Data Classification via Group-Wise Hybrid Temporal and Spatial Sparse Representations
Source: eNeuro. 2022 Jun 3;9(3):ENEURO.0478-21.2022. doi: 10.1523/ENEURO.0478-21.2022 (PMC9186416; doi:10.1523/ENEURO.0478-21.2022)
Supplement: Extended Data 1 — The code. Download Extended Data 1, ZIP file. [file enu-eN-MNT-0478-21-s02.zip › code/tools/spams-matlab.zip!/spams-matlab/doc/html/doc_spams004.html]

Dictionary Learning and Matrix Factorization Toolbox


---

## 3  Dictionary Learning and Matrix Factorization Toolbox

- Function mexTrainDL
- Function mexTrainDL\_Memory
- Function mexStructTrainDL
- Function nmf
- Function nnsc
- Function mexArchetypalAnalysis

This is the section for dictionary learning and matrix factorization, corresponding to [20, 21].

### 3.1  Function mexTrainDL

This is the main function of the toolbox, implementing the learning algorithms of [21].
Given a training set x^1^,…, . It aims at solving

|  |  |  |  |  |  |  |  |  |  |  |  |  |  |  |  |
| --- | --- | --- | --- | --- | --- | --- | --- | --- | --- | --- | --- | --- | --- | --- | --- |
|  | min; D ∈ C |  | lim; n → +∞ |  | 1; n |  | n; ∑; i=1 |  | min; α^i^ |  | ⎛  ⎜  ⎝ | 1; 2 | \|\|x^i^−Dα^i^\|\|_2_^2^ + ψ(α^i^) | ⎞  ⎟  ⎠ | .     (1) |

ψ is a sparsity-inducing regularizer and C is a constraint set for the dictionary. As shown in [21]
and in the help file below, various combinations can be used for ψ and C for solving different matrix factorization problems.
What is more, positivity constraints can be added to α as well. The function admits several modes for choosing the optimization parameters, using the parameter-free strategy proposed in [20], or using the parameters t_0_ and ρ presented
in [21]. Note that for problems of a reasonable size, and when ψ is the ℓ_1_-norm,
the function mexTrainDL\_Memory can be faster but uses more memory.

%   
% Usage:   [D [model]]=mexTrainDL(X,param[,model]);  
%          model is optional  
%  
% Name: mexTrainDL  
%  
% Description: mexTrainDL is an efficient implementation of the  
%     dictionary learning technique presented in  
%  
%     "Online Learning for Matrix Factorization and Sparse Coding"  
%     by Julien Mairal, Francis Bach, Jean Ponce and Guillermo Sapiro  
%     arXiv:0908.0050  
%       
%     "Online Dictionary Learning for Sparse Coding"        
%     by Julien Mairal, Francis Bach, Jean Ponce and Guillermo Sapiro  
%     ICML 2009.  
%  
%     Note that if you use param.mode=1 or 2, if the training set has a  
%     reasonable size and you have enough memory on your computer, you   
%     should use mexTrainDL\_Memory instead.  
%   
%  
%     It addresses the dictionary learning problems  
%        1) if param.mode=0  
%     min\_{D in C} (1/n) sum\_{i=1}^n (1/2)||x\_i-Dalpha\_i||\_2^2  s.t. ...  
%                                                  ||alpha\_i||\_1 <= lambda  
%        2) if param.mode=1  
%     min\_{D in C} (1/n) sum\_{i=1}^n  ||alpha\_i||\_1  s.t.  ...  
%                                           ||x\_i-Dalpha\_i||\_2^2 <= lambda  
%        3) if param.mode=2  
%     min\_{D in C} (1/n) sum\_{i=1}^n (1/2)||x\_i-Dalpha\_i||\_2^2 + ...   
%                                  lambda||alpha\_i||\_1 + lambda\_2||alpha\_i||\_2^2  
%        4) if param.mode=3, the sparse coding is done with OMP  
%     min\_{D in C} (1/n) sum\_{i=1}^n (1/2)||x\_i-Dalpha\_i||\_2^2  s.t. ...   
%                                                  ||alpha\_i||\_0 <= lambda  
%        5) if param.mode=4, the sparse coding is done with OMP  
%     min\_{D in C} (1/n) sum\_{i=1}^n  ||alpha\_i||\_0  s.t.  ...  
%                                           ||x\_i-Dalpha\_i||\_2^2 <= lambda  
%        6) if param.mode=5, the sparse coding is done with OMP  
%     min\_{D in C} (1/n) sum\_{i=1}^n 0.5||x\_i-Dalpha\_i||\_2^2 +lambda||alpha\_i||\_0    
%                                             
%  
%%     C is a convex set verifying  
%        1) if param.modeD=0  
%           C={  D in Real^{m x p}  s.t.  forall j,  ||d\_j||\_2^2 <= 1 }  
%        2) if param.modeD=1  
%           C={  D in Real^{m x p}  s.t.  forall j,  ||d\_j||\_2^2 + ...   
%                                                  gamma1||d\_j||\_1 <= 1 }  
%        3) if param.modeD=2  
%           C={  D in Real^{m x p}  s.t.  forall j,  ||d\_j||\_2^2 + ...   
%                                  gamma1||d\_j||\_1 + gamma2 FL(d\_j) <= 1 }  
%        4) if param.modeD=3  
%           C={  D in Real^{m x p}  s.t.  forall j,  (1-gamma1)||d\_j||\_2^2 + ...   
%                                  gamma1||d\_j||\_1 <= 1 }  
  
%  
%     Potentially, n can be very large with this algorithm.  
%  
% Inputs: X:  double m x n matrix   (input signals)  
%               m is the signal size  
%               n is the number of signals to decompose  
%         param: struct  
%            param.D: (optional) double m x p matrix   (dictionary)  
%              p is the number of elements in the dictionary  
%              When D is not provided, the dictionary is initialized   
%              with random elements from the training set.  
%           param.K (size of the dictionary, optional is param.D is provided)  
%           param.lambda  (parameter)  
%           param.lambda2  (optional, by default 0)  
%           param.iter (number of iterations).  If a negative number is   
%              provided it will perform the computation during the  
%              corresponding number of seconds. For instance param.iter=-5  
%              learns the dictionary during 5 seconds.  
%           param.mode (optional, see above, by default 2)   
%           param.posAlpha (optional, adds positivity constraints on the  
%             coefficients, false by default, not compatible with   
%             param.mode =3,4)  
%           param.modeD (optional, see above, by default 0)  
%           param.posD (optional, adds positivity constraints on the   
%             dictionary, false by default, not compatible with   
%             param.modeD=2)  
%           param.gamma1 (optional parameter for param.modeD >= 1)  
%           param.gamma2 (optional parameter for param.modeD = 2)  
%           param.batchsize (optional, size of the minibatch, by default   
%              512)  
%           param.iter\_updateD (optional, number of BCD iterations for the dictionary  
%              update step, by default 1)  
%           param.modeParam (optimization mode).  
%              1) if param.modeParam=0, the optimization uses the   
%                 parameter free strategy of the ICML paper  
%              2) if param.modeParam=1, the optimization uses the   
%                 parameters rho as in arXiv:0908.0050  
%              3) if param.modeParam=2, the optimization uses exponential   
%                 decay weights with updates of the form   
%                 A\_{t} <- rho A\_{t-1} + alpha\_t alpha\_t^T  
%           param.rho (optional) tuning parameter (see paper arXiv:0908.0050)  
%           param.t0 (optional) tuning parameter (see paper arXiv:0908.0050)  
%           param.clean (optional, true by default. prunes   
%              automatically the dictionary from unused elements).  
%           param.verbose (optional, true by default, increase verbosity)  
%           param.numThreads (optional, number of threads for exploiting  
%              multi-core / multi-cpus. By default, it takes the value -1,  
%              which automatically selects all the available CPUs/cores).  
%  
% Output:   
%         param.D: double m x p matrix   (dictionary)  
%  
% Note: this function admits a few experimental usages, which have not  
%     been extensively tested:  
%         - single precision setting   
%  
% Author: Julien Mairal, 2009

The following piece of code illustrates how to use this function.

clear all;  
  
I=double(imread('data/lena.png'))/255;  
% extract 8 x 8 patches  
X=im2col(I,[8 8],'sliding');  
X=X-repmat(mean(X),[size(X,1) 1]);  
X=X ./ repmat(sqrt(sum(X.^2)),[size(X,1) 1]);  
  
param.K=256;  % learns a dictionary with 100 elements  
param.lambda=0.15;  
param.numThreads=-1; % number of threads  
param.batchsize=400;  
param.verbose=false;  
  
param.iter=1000;  % let us see what happens after 1000 iterations.  
  
%%%%%%%%%% FIRST EXPERIMENT %%%%%%%%%%%  
tic  
D = mexTrainDL(X,param);  
t=toc;  
fprintf('time of computation for Dictionary Learning: %f\n',t);  
  
fprintf('Evaluating cost function...\n');  
alpha=mexLasso(X,D,param);  
R=mean(0.5\*sum((X-D\*alpha).^2)+param.lambda\*sum(abs(alpha)));  
ImD=displayPatches(D);  
subplot(1,3,1);  
imagesc(ImD); colormap('gray');  
fprintf('objective function: %f\n',R);  
drawnow;  
  
fprintf('\*\*\*\*\*\*\*\*\*\*\* SECOND EXPERIMENT \*\*\*\*\*\*\*\*\*\*\*\n');  
%%%%%%%%%% SECOND EXPERIMENT %%%%%%%%%%%  
% Train on half of the training set, then retrain on the second part  
X1=X(:,1:floor(size(X,2)/2));  
X2=X(:,floor(size(X,2)/2):end);  
param.iter=500;  
tic  
[D model] = mexTrainDL(X1,param);  
t=toc;  
fprintf('time of computation for Dictionary Learning: %f\n',t);  
fprintf('Evaluating cost function...\n');  
alpha=mexLasso(X,D,param);  
R=mean(0.5\*sum((X-D\*alpha).^2)+param.lambda\*sum(abs(alpha)));  
fprintf('objective function: %f\n',R);  
tic  
% Then reuse the learned model to retrain a few iterations more.  
param2=param;  
param2.D=D;  
[D model] = mexTrainDL(X2,param2,model);  
%[D] = mexTrainDL(X,param);  
t=toc;  
fprintf('time of computation for Dictionary Learning: %f\n',t);  
fprintf('Evaluating cost function...\n');  
alpha=mexLasso(X,D,param);  
R=mean(0.5\*sum((X-D\*alpha).^2)+param.lambda\*sum(abs(alpha)));  
fprintf('objective function: %f\n',R);  
  
% let us add sparsity to the dictionary itself  
fprintf('\*\*\*\*\*\*\*\*\*\*\* THIRD EXPERIMENT \*\*\*\*\*\*\*\*\*\*\*\n');  
param.modeParam=0;  
param.iter=1000;  
param.gamma1=0.3;  
param.modeD=1;  
tic  
[D] = mexTrainDL(X,param);  
t=toc;  
fprintf('time of computation for Dictionary Learning: %f\n',t);  
fprintf('Evaluating cost function...\n');  
alpha=mexLasso(X,D,param);  
R=mean(0.5\*sum((X-D\*alpha).^2)+param.lambda\*sum(abs(alpha)));  
fprintf('objective function: %f\n',R);  
tic  
subplot(1,3,2);  
ImD=displayPatches(D);  
imagesc(ImD); colormap('gray');  
drawnow;  
  
fprintf('\*\*\*\*\*\*\*\*\*\*\* FOURTH EXPERIMENT \*\*\*\*\*\*\*\*\*\*\*\n');  
param.modeParam=0;  
param.iter=1000;  
param.gamma1=0.3;  
param.modeD=3;  
tic  
[D] = mexTrainDL(X,param);  
t=toc;  
fprintf('time of computation for Dictionary Learning: %f\n',t);  
fprintf('Evaluating cost function...\n');  
alpha=mexLasso(X,D,param);  
R=mean(0.5\*sum((X-D\*alpha).^2)+param.lambda\*sum(abs(alpha)));  
fprintf('objective function: %f\n',R);  
tic  
subplot(1,3,3);  
ImD=displayPatches(D);  
imagesc(ImD); colormap('gray');  
drawnow;

### 3.2  Function mexTrainDL\_Memory

Memory-consuming version of mexTrainDL. This function is well adapted to small/medium-size problems:
It requires storing all the coefficients α and is therefore impractical
for very large datasets. However, in many situations, one can afford this memory cost and it is better to use this method, which
is faster than mexTrainDL.
Note that unlike mexTrainDL this function does not allow warm-restart.

%   
% Usage:   [D]=mexTrainDL(X,param);  
%  
% Name: mexTrainDL\_Memory  
%  
% Description: mexTrainDL\_Memory is an efficient but memory consuming   
%     variant of the dictionary learning technique presented in  
%  
%     "Online Learning for Matrix Factorization and Sparse Coding"  
%     by Julien Mairal, Francis Bach, Jean Ponce and Guillermo Sapiro  
%     arXiv:0908.0050  
%       
%     "Online Dictionary Learning for Sparse Coding"        
%     by Julien Mairal, Francis Bach, Jean Ponce and Guillermo Sapiro  
%     ICML 2009.  
%  
%     Contrary to the approaches above, the algorithm here   
%        does require to store all the coefficients from all the training  
%        signals. For this reason this variant can not be used with large  
%        training sets, but is more efficient than the regular online  
%        approach for training sets of reasonable size.  
%  
%     It addresses the dictionary learning problems  
%        1) if param.mode=1  
%     min\_{D in C} (1/n) sum\_{i=1}^n  ||alpha\_i||\_1  s.t.  ...  
%                                         ||x\_i-Dalpha\_i||\_2^2 <= lambda  
%        2) if param.mode=2  
%     min\_{D in C} (1/n) sum\_{i=1}^n (1/2)||x\_i-Dalpha\_i||\_2^2 + ...   
%                                                      lambda||alpha\_i||\_1    
%  
%     C is a convex set verifying  
%        1) if param.modeD=0  
%           C={  D in Real^{m x p}  s.t.  forall j,  ||d\_j||\_2^2 <= 1 }  
%        1) if param.modeD=1  
%           C={  D in Real^{m x p}  s.t.  forall j,  ||d\_j||\_2^2 + ...   
%                                                  gamma1||d\_j||\_1 <= 1 }  
%        1) if param.modeD=2  
%           C={  D in Real^{m x p}  s.t.  forall j,  ||d\_j||\_2^2 + ...   
%                                  gamma1||d\_j||\_1 + gamma2 FL(d\_j) <= 1 }  
%  
%     Potentially, n can be very large with this algorithm.  
%  
% Inputs: X:  double m x n matrix   (input signals)  
%               m is the signal size  
%               n is the number of signals to decompose  
%         param: struct  
%            param.D: (optional) double m x p matrix   (dictionary)  
%              p is the number of elements in the dictionary  
%              When D is not provided, the dictionary is initialized   
%              with random elements from the training set.  
%           param.K (size of the dictionary, optional is param.D is provided)  
%           param.lambda  (parameter)  
%           param.iter (number of iterations).  If a negative number is   
%              provided it will perform the computation during the  
%              corresponding number of seconds. For instance param.iter=-5  
%              learns the dictionary during 5 seconds.  
%            param.mode (optional, see above, by default 2)   
%            param.modeD (optional, see above, by default 0)  
%            param.posD (optional, adds positivity constraints on the   
%              dictionary, false by default, not compatible with   
%              param.modeD=2)  
%            param.gamma1 (optional parameter for param.modeD >= 1)  
%            param.gamma2 (optional parameter for param.modeD = 2)  
%            param.batchsize (optional, size of the minibatch, by default   
%              512)  
%            param.iter\_updateD (optional, number of BCD iterations for the dictionary   
%                update step, by default 1)  
%            param.modeParam (optimization mode).  
%              1) if param.modeParam=0, the optimization uses the   
%                 parameter free strategy of the ICML paper  
%              2) if param.modeParam=1, the optimization uses the   
%                 parameters rho as in arXiv:0908.0050  
%              3) if param.modeParam=2, the optimization uses exponential   
%                 decay weights with updates of the form   
%                 A\_{t} <- rho A\_{t-1} + alpha\_t alpha\_t^T  
%            param.rho (optional) tuning parameter (see paper arXiv:0908.0050)  
%            param.t0 (optional) tuning parameter (see paper arXiv:0908.0050)  
%            param.clean (optional, true by default. prunes   
%              automatically the dictionary from unused elements).  
%            param.numThreads (optional, number of threads for exploiting  
%              multi-core / multi-cpus. By default, it takes the value -1,  
%              which automatically selects all the available CPUs/cores).  
%  
% Output:   
%         param.D: double m x p matrix   (dictionary)  
%  
% Note: this function admits a few experimental usages, which have not  
%     been extensively tested:  
%         - single precision setting (even though the output alpha is double   
%           precision)  
%  
% Author: Julien Mairal, 2009

The following piece of code illustrates how to use this function.

clear all;  
  
I=double(imread('data/lena.png'))/255;  
% extract 8 x 8 patches  
X=im2col(I,[8 8],'sliding');  
X=X-repmat(mean(X),[size(X,1) 1]);  
X=X ./ repmat(sqrt(sum(X.^2)),[size(X,1) 1]);  
X=X(:,1:10:end);  
  
param.K=200;  % learns a dictionary with 100 elements  
param.lambda=0.15;  
param.numThreads=4; % number of threads  
  
param.iter=100;  % let us see what happens after 100 iterations.  
  
%%%%%%%%%% FIRST EXPERIMENT %%%%%%%%%%%  
tic  
D = mexTrainDL\_Memory(X,param);  
t=toc;  
fprintf('time of computation for Dictionary Learning: %f\n',t);  
  
fprintf('Evaluating cost function...\n');  
alpha=mexLasso(X,D,param);  
R=mean(0.5\*sum((X-D\*alpha).^2)+param.lambda\*sum(abs(alpha)));  
ImD=displayPatches(D);  
subplot(1,3,1);  
imagesc(ImD); colormap('gray');  
fprintf('objective function: %f\n',R);  
  
%%%%%%%%%% SECOND EXPERIMENT %%%%%%%%%%%  
tic  
D = mexTrainDL(X,param);  
t=toc;  
fprintf('time of computation for Dictionary Learning: %f\n',t);  
  
fprintf('Evaluating cost function...\n');  
alpha=mexLasso(X,D,param);  
R=mean(0.5\*sum((X-D\*alpha).^2)+param.lambda\*sum(abs(alpha)));  
ImD=displayPatches(D);  
subplot(1,3,2);  
imagesc(ImD); colormap('gray');  
fprintf('objective function: %f\n',R);

### 3.3  Function mexStructTrainDL

This function allows to use mexTrainDL with structured regularization functions
for the coefficients α. It internally uses the FISTA algorithm.

%   
% Usage:   [D [model]]=mexStructTrainDL(X,param[,model]);  
%          model is optional  
%  
% Name: mexStructTrainDL  
%  
% Description: mexStructTrainDL is an efficient implementation of the  
%     dictionary learning technique presented in  
%  
%     "Online Learning for Matrix Factorization and Sparse Coding"  
%     by Julien Mairal, Francis Bach, Jean Ponce and Guillermo Sapiro  
%     arXiv:0908.0050  
%       
%     "Online Dictionary Learning for Sparse Coding"        
%     by Julien Mairal, Francis Bach, Jean Ponce and Guillermo Sapiro  
%     ICML 2009.  
%  
%  
%     It addresses the dictionary learning problems  
%        min\_{D in C} (1/n) sum\_{i=1}^n 0.5||x\_i-Dalpha\_i||\_2^2 + lambda psi(alpha)  
%        where the regularization function psi depends on param.regul  
%        (see mexProximalFlat for the description of psi,  
%         and param.regul below for allowed values of regul)  
%  
%%     C is a convex set verifying  
%        1) if param.modeD=0  
%           C={  D in Real^{m x p}  s.t.  forall j,  ||d\_j||\_2^2 <= 1 }  
%        2) if param.modeD=1  
%           C={  D in Real^{m x p}  s.t.  forall j,  ||d\_j||\_2^2 + ...   
%                                                  gamma1||d\_j||\_1 <= 1 }  
%        3) if param.modeD=2  
%           C={  D in Real^{m x p}  s.t.  forall j,  ||d\_j||\_2^2 + ...   
%                                  gamma1||d\_j||\_1 + gamma2 FL(d\_j) <= 1 }  
%        4) if param.modeD=3  
%           C={  D in Real^{m x p}  s.t.  forall j,  (1-gamma1)||d\_j||\_2^2 + ...   
%                                  gamma1||d\_j||\_1 <= 1 }  
  
%  
%     Potentially, n can be very large with this algorithm.  
%  
% Inputs: X:  double m x n matrix   (input signals)  
%               m is the signal size  
%               n is the number of signals to decompose  
%         param: struct  
%            param.D: (optional) double m x p matrix   (dictionary)  
%              p is the number of elements in the dictionary  
%              When D is not provided, the dictionary is initialized   
%              with random elements from the training set.  
%           param.K (size of the dictionary, optional is param.D is provided)  
%           param.lambda  (parameter)  
%           param.lambda2  (optional, by default 0)  
%           param.lambda3 (optional, regularization parameter, 0 by default)  
%           param.iter (number of iterations).  If a negative number is   
%              provided it will perform the computation during the  
%              corresponding number of seconds. For instance param.iter=-5  
%              learns the dictionary during 5 seconds.  
%           param.regul choice of regularization : one of  
%               'l0' 'l1' 'l2' 'linf' 'none' 'elastic-net' 'fused-lasso'  
%               'graph' 'graph-ridge' 'graph-l2' 'tree-l0' 'tree-l2' 'tree-linf'   
%           param.tree struct (see documentation of mexProximalTree);  
%               needed for param.regul of graph kind.  
%           param.graph struct (see documentation of mexProximalGraph);  
%               needed for param.regul of tree kind.  
%           param.posAlpha (optional, adds positivity constraints on the  
%               coefficients, false by default.  
%           param.modeD (optional, see above, by default 0)  
%           param.posD (optional, adds positivity constraints on the   
%             dictionary, false by default, not compatible with   
%             param.modeD=2)  
%           param.gamma1 (optional parameter for param.modeD >= 1)  
%           param.gamma2 (optional parameter for param.modeD = 2)  
%           param.batchsize (optional, size of the minibatch, by default   
%              512)  
%           param.iter\_updateD (optional, number of BCD iterations for the dictionary  
%              update step, by default 1)  
%           param.modeParam (optimization mode).  
%              1) if param.modeParam=0, the optimization uses the   
%                 parameter free strategy of the ICML paper  
%              2) if param.modeParam=1, the optimization uses the   
%                 parameters rho as in arXiv:0908.0050  
%              3) if param.modeParam=2, the optimization uses exponential   
%                 decay weights with updates of the form   
%                 A\_{t} <- rho A\_{t-1} + alpha\_t alpha\_t^T  
%            param.ista (optional, use ista instead of fista, false by default).  
%            param.tol (optional, tolerance for stopping criteration, which is a relative duality gap  
%            param.fixed\_step (deactive the line search for L in fista and use param.K instead)  
%           param.rho (optional) tuning parameter (see paper arXiv:0908.0050)  
%           param.t0 (optional) tuning parameter (see paper arXiv:0908.0050)  
%           param.clean (optional, true by default. prunes   
%              automatically the dictionary from unused elements).  
%           param.verbose (optional, true by default, increase verbosity)  
%           param.numThreads (optional, number of threads for exploiting  
%              multi-core / multi-cpus. By default, it takes the value -1,  
%              which automatically selects all the available CPUs/cores).  
%  
% Output:   
%         param.D: double m x p matrix   (dictionary)  
%  
% Note: this function admits a few experimental usages, which have not  
%     been extensively tested:  
%         - single precision setting   
%  
% Author: Julien Mairal, 2009

The following piece of code illustrates how to use this function.

clear all;  
  
I=double(imread('data/lena.png'))/255;  
% extract 8 x 8 patches  
X=im2col(I,[8 8],'sliding');  
X=X-repmat(mean(X),[size(X,1) 1]);  
X=X ./ repmat(sqrt(sum(X.^2)),[size(X,1) 1]);  
  
param.K=64;  % learns a dictionary with 64 elements  
param.lambda=0.05;  
param.numThreads=4; % number of threads  
param.batchsize=400;  
param.tol = 1e-3  
  
param.iter=200;  %  
  
if false  
param.regul = 'l1';  
fprintf('with Fista Regression %s\n',param.regul);  
tic  
D = mexStructTrainDL(X,param);  
t=toc;  
fprintf('time of computation for Dictionary Learning: %f\n',t);  
%  
param.regul = 'l2';  
fprintf('with Fista Regression %s\n',param.regul);  
tic  
D = mexStructTrainDL(X,param);  
t=toc;  
fprintf('time of computation for Dictionary Learning: %f\n',t);  
%  
param.regul = 'elastic-net';  
fprintf('with Fista %s\n',param.regul);  
param.lambda2=0.1;  
tic  
D = mexStructTrainDL(X,param);  
t=toc;  
fprintf('time of computation for Dictionary Learning: %f\n',t);  
  
%%% GRAPH  
param.lambda=0.1; % regularization parameter  
param.tol=1e-5;  
param.K = 10  
graph.eta\_g=[1 1 1 1 1];  
graph.groups=sparse([0 0 0 1 0;  
                     0 0 0 0 0;  
                     0 0 0 0 0;  
                     0 0 0 0 0;  
                     0 0 1 0 0]);   % g5 is included in g3, and g2 is included in g4  
graph.groups\_var=sparse([1 0 0 0 0;  
                         1 0 0 0 0;  
                         1 0 0 0 0 ;  
                         1 1 0 0 0;  
                         0 1 0 1 0;  
                         0 1 0 1 0;  
                         0 1 0 0 1;  
                         0 0 0 0 1;  
                         0 0 0 0 1;  
                         0 0 1 0 0]); % represents direct inclusion relations   
  
param.graph = graph  
  
param.regul = 'graph';  
fprintf('with Fista %s\n',param.regul);  
tic  
D = mexStructTrainDL(X,param);  
t=toc;  
fprintf('time of computation for Dictionary Learning: %f\n',t);  
  
%%  
%%% TREE  
%?pause;  
param = rmfield(param,'graph');  
  
end  
  
  
param.lambda=0.1; % regularization parameter  
param.tol=1e-5;  
param.K = 10  
  
tree.own\_variables=  int32([0 0 3 5 6 6 8 9]);   % pointer to the first variable of each group  
tree.N\_own\_variables=int32([0 3 2 1 0 2 1 1]); % number of "root" variables in each group  
tree.eta\_g=[1 1 1 2 2 2 2.5 2.5];  
tree.groups=sparse([0 0 0 0 0 0 0 0; ...  
                    1 0 0 0 0 0 0 0; ...  
                    0 1 0 0 0 0 0 0; ...  
                    0 1 0 0 0 0 0 0; ...  
                    1 0 0 0 0 0 0 0; ...  
                    0 0 0 0 1 0 0 0; ...  
                    0 0 0 0 1 0 0 0; ...  
                    0 0 0 0 0 0 1 0]);  % first group should always be the root of the tree  
  
param.tree = tree;  
  
param.regul = 'tree-l0';  
fprintf('with Fista %s\n',param.regul);  
  
tic  
D = mexStructTrainDL(X,param);  
t=toc;  
fprintf('time of computation for Dictionary Learning: %f\n',t);  
  
%  
param.regul = 'tree-l2';  
fprintf('with Fista %s\n',param.regul);  
tic  
D = mexStructTrainDL(X,param);  
t=toc;  
fprintf('time of computation for Dictionary Learning: %f\n',t);  
  
%  
param.regul = 'tree-linf';  
fprintf('with Fista %s\n',param.regul);  
  
tic  
D = mexStructTrainDL(X,param);  
t=toc;  
fprintf('time of computation for Dictionary Learning: %f\n',t);

### 3.4  Function nmf

This function is an example on how to use the function mexTrainDL for the
problem of non-negative matrix factorization formulated in [17]. Note
that mexTrainDL can be replaced by mexTrainDL\_Memory in this function for
small or medium datasets.

%   
% Usage:   [U [,V]]=nmf(X,param);  
%  
% Name: nmf  
%  
% Description: mexTrainDL is an efficient implementation of the  
%     non-negative matrix factorization technique presented in   
%  
%     "Online Learning for Matrix Factorization and Sparse Coding"  
%     by Julien Mairal, Francis Bach, Jean Ponce and Guillermo Sapiro  
%     arXiv:0908.0050  
%       
%     "Online Dictionary Learning for Sparse Coding"        
%     by Julien Mairal, Francis Bach, Jean Ponce and Guillermo Sapiro  
%     ICML 2009.  
%  
%     Potentially, n can be very large with this algorithm.  
%  
% Inputs: X:  double m x n matrix   (input signals)  
%               m is the signal size  
%               n is the number of signals to decompose  
%         param: struct  
%            param.K (number of required factors)  
%            param.iter (number of iterations).  If a negative number   
%              is provided it will perform the computation during the  
%              corresponding number of seconds. For instance param.iter=-5  
%              learns the dictionary during 5 seconds.  
%            param.batchsize (optional, size of the minibatch, by default   
%               512)  
%            param.modeParam (optimization mode).  
%               1) if param.modeParam=0, the optimization uses the   
%                  parameter free strategy of the ICML paper  
%               2) if param.modeParam=1, the optimization uses the   
%                  parameters rho as in arXiv:0908.0050  
%               3) if param.modeParam=2, the optimization uses exponential   
%                  decay weights with updates of the form    
%                  A\_{t} <- rho A\_{t-1} + alpha\_t alpha\_t^T  
%            param.rho (optional) tuning parameter (see paper   
%              arXiv:0908.0050)  
%            param.t0 (optional) tuning parameter (see paper   
%              arXiv:0908.0050)  
%            param.clean (optional, true by default. prunes automatically   
%              the dictionary from unused elements).  
%            param.batch (optional, false by default, use batch learning   
%              instead of online learning)  
%            param.numThreads (optional, number of threads for exploiting  
%                 multi-core / multi-cpus. By default, it takes the value -1,  
%                 which automatically selects all the available CPUs/cores).  
%         model: struct (optional) learned model for "retraining" the data.  
%  
% Output:  
%         U: double m x p matrix     
%         V: double p x n matrix   (optional)  
%         model: struct (optional) learned model to be used for   
%           "retraining" the data.  
%  
% Author: Julien Mairal, 2009  
function [U V] = nmf(X,param)  
  
param.lambda=0;  
param.mode=2;  
param.posAlpha=1;  
param.posD=1;  
param.whiten=0;  
U=mexTrainDL(X,param);  
param.pos=1;  
if nargout == 2  
   if issparse(X) % todo allow sparse matrices X for mexLasso  
      maxbatch=ceil(10000000/size(X,1));  
      for jj = 1:maxbatch:size(X,2)  
         indbatch=jj:min((jj+maxbatch-1),size(X,2));  
         Xb=full(X(:,indbatch));  
         V(:,indbatch)=mexLasso(Xb,U,param);  
      end  
   else  
      V=mexLasso(X,U,param);  
   end  
end

The following piece of code illustrates how to use this function.

clear all;  
  
I=double(imread('data/lena.png'))/255;  
% extract 8 x 8 patches  
X=im2col(I,[16 16],'sliding');  
X=X(:,1:10:end);  
X=X ./ repmat(sqrt(sum(X.^2)),[size(X,1) 1]);  
  
param.K=49;  % learns a dictionary with 100 elements  
param.numThreads=4; % number of threads  
  
param.iter=-5;  % let us see what happens after 100 iterations.  
  
%%%%%%%%%% FIRST EXPERIMENT %%%%%%%%%%%  
tic  
[U V] = nmf(X,param);  
t=toc;  
fprintf('time of computation for Dictionary Learning: %f\n',t);  
  
fprintf('Evaluating cost function...\n');  
R=mean(0.5\*sum((X-U\*V).^2));  
ImD=displayPatches(U);  
imagesc(ImD); colormap('gray');  
fprintf('objective function: %f\n',R);

### 3.5  Function nnsc

This function is an example on how to use the function mexTrainDL for the
problem of non-negative sparse coding as defined in [14]. Note that
mexTrainDL can be replaced by mexTrainDL\_Memory in this function for small or
medium datasets.

%   
% Usage:   [U [,V]]=nnsc(X,param);  
%  
% Name: nmf  
%  
% Description: mexTrainDL is an efficient implementation of the  
%     non-negative sparse coding technique presented in   
%  
%     "Online Learning for Matrix Factorization and Sparse Coding"  
%     by Julien Mairal, Francis Bach, Jean Ponce and Guillermo Sapiro  
%     arXiv:0908.0050  
%       
%     "Online Dictionary Learning for Sparse Coding"        
%     by Julien Mairal, Francis Bach, Jean Ponce and Guillermo Sapiro  
%     ICML 2009.  
%  
%     Potentially, n can be very large with this algorithm.  
%  
% Inputs: X:  double m x n matrix   (input signals)  
%               m is the signal size  
%               n is the number of signals to decompose  
%         param: struct  
%           param.K (number of required factors)  
%           param.lambda (parameter)  
%           param.iter (number of iterations).  If a negative number   
%              is provided it will perform the computation during the  
%              corresponding number of seconds. For instance param.iter=-5  
%              learns the dictionary during 5 seconds.  
%           param.batchsize (optional, size of the minibatch, by default   
%              512)  
%           param.modeParam (optimization mode).  
%              1) if param.modeParam=0, the optimization uses the   
%                 parameter free strategy of the ICML paper  
%              2) if param.modeParam=1, the optimization uses the   
%                 parameters rho as in arXiv:0908.0050  
%              3) if param.modeParam=2, the optimization uses exponential   
%                 decay weights with updates of the form   
%                 A\_{t} <- rho A\_{t-1} + alpha\_t alpha\_t^T  
%           param.rho (optional) tuning parameter (see paper  
%              arXiv:0908.0050)  
%           param.t0 (optional) tuning parameter (see paper   
%              arXiv:0908.0050)  
%           param.clean (optional, true by default. prunes automatically   
%              the dictionary from unused elements).  
%           param.batch (optional, false by default, use batch learning   
%              instead of online learning)  
%           param.numThreads (optional, number of threads for exploiting  
%              multi-core / multi-cpus. By default, it takes the value -1,  
%              which automatically selects all the available CPUs/cores).  
%         model: struct (optional) learned model for "retraining" the data.  
%  
% Output:  
%         U: double m x p matrix     
%         V: double p x n matrix   (optional)  
%         model: struct (optional) learned model to be used for   
%            "retraining" the data.  
%  
% Author: Julien Mairal, 2009  
function [U V] = nnsc(X,param)  
  
param.mode=2;  
param.posAlpha=1;  
param.posD=1;  
param.whiten=0;  
U=mexTrainDL(X,param);  
param.pos=1;  
if nargout == 2  
   V=mexLasso(X,U,param);  
end

### 3.6  Function mexArchetypalAnalysis

The function optimizes the archetypal analysis formulation of [7]. It follows the
methodology presented in the following paper [37].

%   
% Usage:  [Z [A,B]]=mexArchetypalAnalysis(X,param);  
% Usage:  [Z [A,B]]=mexArchetypalAnalysis(X,Z,param);  
%  
% Name: mexArchetypalAnalysis  
%  
% documentation to appear soon  
%  
% Inputs: X:  double m x n matrix   (input signals)  
%               m is the signal size  
%               n is the number of signals to decompose  
% Output: Z: double %  
%  
% Author: Yuansi Chen and Julien Mairal, 2014

The following piece of code illustrates how to use this function.

clear all;  
rand('seed',0);  
I=double(imread('data/lena.png'))/255;  
% extract 8 x 8 patches  
X=im2col(I,[8 8],'sliding');  
per=randperm(size(X,2));  
X=X(:,per(1:10000));  
X=X-repmat(mean(X),[size(X,1) 1]);  
nrm=sqrt(sum(X.^2));  
me=median(nrm);  
X=X ./ repmat(max(nrm,me),[size(X,1) 1]);  
  
param.p=64;  % learns a dictionary with 64 elements  
param.robust=false;  
param.epsilon=1e-3;  % width for Huber loss  
param.computeXtX=true;  
param.stepsFISTA=0;  
param.stepsAS=10;  
param.numThreads=-1;  
  
%%%%%%%%%% FIRST EXPERIMENT %%%%%%%%%%%  
tic  
[Z A B] = mexArchetypalAnalysis(X,param);  
t=toc;  
fprintf('time of computation for Archetypal Analysis: %f\n',t);  
R=norm(X-Z\*A,'fro')^2;  
fprintf('objective function: %f\n',R);  
  
fprintf('Re-Evaluating cost function after re-updating A...\n');  
paramdecomp.computeXtX=true;  
paramdecomp.numThreads=-1;  
A2=mexDecompSimplex(X,Z,param);  
R=norm(X-Z\*A2,'fro')^2;  
fprintf('objective function: %f\n',R);  
  
%%%%%%%%%% Second EXPERIMENT %%%%%%%%%%%  
tic  
[Z2 A2] = mexArchetypalAnalysis(X,param,Z);  
t=toc;  
fprintf('time of computation for Archetypal Analysis: %f\n',t);  
fprintf('Evaluating cost function...\n');  
R=norm(X-Z2\*A2,'fro')^2;  
fprintf('objective function: %f\n',R);  
  
%%%%%%%%%% THIRD EXPERIMENT %%%%%%%%%%%  
tic  
param.robust=true;  
[Z3] = mexArchetypalAnalysis(X,param);  
t=toc;  
fprintf('time of computation for Robust Archetypal Analysis: %f\n',t);

---
