# Supplementary material for: Multitask fMRI Data Classification via Group-Wise Hybrid Temporal and Spatial Sparse Representations
Source: eNeuro. 2022 Jun 3;9(3):ENEURO.0478-21.2022. doi: 10.1523/ENEURO.0478-21.2022 (PMC9186416; doi:10.1523/ENEURO.0478-21.2022)
Supplement: Extended Data 1 — The code. Download Extended Data 1, ZIP file. [file enu-eN-MNT-0478-21-s02.zip › code/tools/spams-matlab.zip!/spams-matlab/doc/html/doc_spams005.html]

Sparse Decomposition Toolbox


---

## 4  Sparse Decomposition Toolbox

- Function mexOMP
- Function mexOMPMask
- Function mexRidgeRegression
- Function mexLasso
- Function mexLassoWeighted
- Function mexLassoMask
- Function mexCD
- Function mexSOMP
- Function mexL1L2BCD
- Function mexSparseProject
- Function mexDecompSimplex

This toolbox implements several algorithms for solving signal reconstruction problems. It is mostly adapted for solving a large number of small/medium scale problems, but can be also efficient sometimes with large scale ones.

### 4.1  Function mexOMP

This is a fast implementation of the Orthogonal Matching Pursuit algorithm (or forward selection) [27, 35]. Given a matrix of signals X=[x^1^,…,x^n^] in ℝ^m × n^ and a dictionary D=[d^1^,…,d^p^] in ℝ^m × p^, the algorithm computes a matrix A=[α^1^,…,α^n^] in ℝ^p × n^,
where for each column x of X, it returns a coefficient vector α which is an approximate solution of the following NP-hard problem

min

α ∈ ℝ^p^

||x−Dα||_2_^2^   s.t.   ||α||_0_ ≤ L,
    (2)

or

min

α ∈ ℝ^p^

||α||_0_   s.t.   ||x−Dα||_2_^2^ ≤ ε,
    (3)

or

min

α ∈ ℝ^p^

1

2

||x−Dα||_2_^2^ + λ ||α||_0_.
    (4)

For efficienty reasons, the method first computes the covariance matrix
D^T^D, then for each signal, it computes D^T^x and performs the
decomposition with a Cholesky-based algorithm (see [6] for instance).

Note that mexOMP can return the “greedy” regularization path if needed (see below):

%   
% Usage:   A=mexOMP(X,D,param);  
% or       [A path]=mexOMP(X,D,param);  
%  
% Name: mexOMP  
%  
% Description: mexOMP is an efficient implementation of the  
%     Orthogonal Matching Pursuit algorithm. It is optimized  
%     for solving a large number of small or medium-sized   
%     decomposition problem (and not for a single large one).  
%     It first computes the Gram matrix D'D and then perform  
%     a Cholesky-based OMP of the input signals in parallel.  
%     X=[x^1,...,x^n] is a matrix of signals, and it returns  
%     a matrix A=[alpha^1,...,alpha^n] of coefficients.  
%       
%     it addresses for all columns x of X,   
%         min\_{alpha} ||alpha||\_0  s.t  ||x-Dalpha||\_2^2 <= eps  
%         or  
%         min\_{alpha} ||x-Dalpha||\_2^2  s.t. ||alpha||\_0 <= L  
%         or  
%         min\_{alpha} 0.5||x-Dalpha||\_2^2 + lambda||alpha||\_0   
%           
%  
% Inputs: X:  double m x n matrix   (input signals)  
%            m is the signal size  
%            n is the number of signals to decompose  
%         D:  double m x p matrix   (dictionary)  
%            p is the number of elements in the dictionary  
%            All the columns of D should have unit-norm !  
%         param: struct  
%            param.L (optional, maximum number of elements in each decomposition,   
%               min(m,p) by default)  
%            param.eps (optional, threshold on the squared l2-norm of the residual,  
%               0 by default  
%            param.lambda (optional, penalty parameter, 0 by default  
%            param.numThreads (optional, number of threads for exploiting  
%            multi-core / multi-cpus. By default, it takes the value -1,  
%            which automatically selects all the available CPUs/cores).  
%  
% Output: A: double sparse p x n matrix (output coefficients)  
%         path (optional): double dense p x L matrix (regularization path of the first signal)  
%  
% Note: this function admits a few experimental usages, which have not  
%     been extensively tested:  
%      - single precision setting (even though the output alpha is double   
%        precision)  
%      - Passing an int32 vector of length n to param.L provides  
%        a different parameter L for each input signal x\_i  
%      - Passing a double vector of length n to param.eps and or param.lambda   
%        provides a different parameter eps (or lambda) for each input signal x\_i  
%  
% Author: Julien Mairal, 2009

The following piece of code illustrates how to use this function.

clear all;  
randn('seed',0);  
  
fprintf('test mexOMP');  
  
%%%%%%%%%%%%%%%%%%%%%%%%%%%%%%%%%%%%%%%%%%%%%%  
% Decomposition of a large number of signals  
%%%%%%%%%%%%%%%%%%%%%%%%%%%%%%%%%%%%%%%%%%%%%%  
X=randn(64,100000);  
D=randn(64,200);  
D=D./repmat(sqrt(sum(D.^2)),[size(D,1) 1]);  
% parameter of the optimization procedure are chosen  
param.L=10; % not more than 10 non-zeros coefficients  
param.eps=0.1; % squared norm of the residual should be less than 0.1  
param.numThreads=-1; % number of processors/cores to use; the default choice is -1  
                    % and uses all the cores of the machine  
tic  
alpha=mexOMP(X,D,param);  
t=toc  
fprintf('%f signals processed per second\n',size(X,2)/t);  
  
%%%%%%%%%%%%%%%%%%%%%%%%%%%%%%%%%%%%%%%%%%  
% Regularization path of a single signal   
%%%%%%%%%%%%%%%%%%%%%%%%%%%%%%%%%%%%%%%%%%  
X=randn(64,1);  
D=randn(64,10);  
param.L=5;  
D=D./repmat(sqrt(sum(D.^2)),[size(D,1) 1]);  
[alpha path]=mexOMP(X,D,param);

### 4.2  Function mexOMPMask

This is a variant of mexOMP with the possibility of handling a binary mask.
Given a binary mask B=[β^1^,…,β^n^] in {0,1}^m × n^, it returns a matrix A=[α^1^,…,α^n^] such that for every column x of X, β of B, it computes a column α of A by addressing

min

α ∈ ℝ^p^

||diag(β)(x−Dα)||_2_^2^   s.t.   ||α||_0_ ≤ L,
    (5)

or

|  |  |  |  |  |
| --- | --- | --- | --- | --- |
|  | min; α ∈ ℝ^p^ | \|\|α\|\|_0_   s.t.   \|\|diag(β)(x−Dα)\|\|_2_^2^ ≤ ε | \|\|β\|\|_0_; m | ,     (6) |

or

min

α ∈ ℝ^p^

1

2

||diag(β)(x−Dα)||_2_^2^ +λ||α||_0_.
    (7)

where diag(β) is a diagonal matrix with the entries of β on the diagonal.

%   
% Usage:   A=mexOMPMask(X,D,B,param);  
% or       [A path]=mexOMPMask(X,D,B,param);  
%  
% Name: mexOMPMask  
%  
% Description: mexOMPMask is a variant of mexOMP that allow using  
%     a binary mask B  
%       
%     for all columns x of X, and columns beta of B, it computes a column   
%         alpha of A by addressing  
%         min\_{alpha} ||alpha||\_0  s.t  ||diag(beta)\*(x-Dalpha)||\_2^2   
%                                                               <= eps\*||beta||\_0/m  
%         or  
%         min\_{alpha} ||diag(beta)\*(x-Dalpha)||\_2^2  s.t. ||alpha||\_0 <= L  
%         or  
%         min\_{alpha} 0.5||diag(beta)\*(x-Dalpha)||\_2^2  + lambda||alpha||\_0  
%           
%  
% Inputs: X:  double m x n matrix   (input signals)  
%            m is the signal size  
%            n is the number of signals to decompose  
%         D:  double m x p matrix   (dictionary)  
%            p is the number of elements in the dictionary  
%            All the columns of D should have unit-norm !  
%         B:  boolean m x n matrix   (mask)  
%               p is the number of elements in the dictionary  
%         param: struct  
%            param.L (optional, maximum number of elements in each decomposition,   
%               min(m,p) by default)  
%            param.eps (optional, threshold on the squared l2-norm of the residual,  
%               0 by default  
%            param.lambda (optional, penalty parameter, 0 by default  
%            param.numThreads (optional, number of threads for exploiting  
%            multi-core / multi-cpus. By default, it takes the value -1,  
%            which automatically selects all the available CPUs/cores).  
%  
% Output: A: double sparse p x n matrix (output coefficients)  
%         path (optional): double dense p x L matrix   
%                                     (regularization path of the first signal)  
%  
% Note: this function admits a few experimental usages, which have not  
%     been extensively tested:  
%      - single precision setting (even though the output alpha is double   
%        precision)  
%      - Passing an int32 vector of length n to param.L provides  
%        a different parameter L for each input signal x\_i  
%      - Passing a double vector of length n to param.eps and or param.lambda   
%        provides a different parameter eps (or lambda) for each input signal x\_i  
%  
% Author: Julien Mairal, 2010

The following piece of code illustrates how to use this function.

clear all;  
  
randn('seed',0);  
fprintf('test mexOMPMask\n');  
  
%%%%%%%%%%%%%%%%%%%%%%%%%%%%%%%%%%%%%%%%%%%%%%  
% Decomposition of a large number of signals  
%%%%%%%%%%%%%%%%%%%%%%%%%%%%%%%%%%%%%%%%%%%%%%  
% Data are generated  
X=randn(100,100);  
X=X./repmat(sqrt(sum(X.^2)),[size(X,1) 1]);  
D=randn(100,20);  
D=D./repmat(sqrt(sum(D.^2)),[size(D,1) 1]);  
mask=(X > 0); % generating a binary mask  
  
% parameter of the optimization procedure are chosen  
param.L=20; % not more than 20 non-zeros coefficients (default: min(size(D,1),size(D,2)))  
param.eps=0.01; %  
param.numThreads=-1; % number of processors/cores to use; the default choice is -1  
                     % and uses all the cores of the machine  
tic  
alpha=mexOMPMask(X,D,mask,param);  
t=toc;  
toc  
  
fprintf('%f signals processed per second\n',size(X,2)/t);

### 4.3  Function mexRidgeRegression

This is a ridge regression solver using a conjugate gradient solver.

%   
% Usage: [W]=mexRidgeRegression(Y,X,W0,param);  
%  
% Name: mexRidgeRegression  
%  
% Description: mexFistaFlat solves sparse regularized problems.  
%         X is a design matrix of size m x p  
%         X=[x^1,...,x^n]', where the x\_i's are the rows of X  
%         Y=[y^1,...,y^n] is a matrix of size m x n  
%         It implements a conjugate gradient solver for ridge regression  
%           
% Inputs: Y:  double dense m x n matrix  
%         X:  double dense or sparse m x p matrix     
%         W0:  double dense p x n matrix or p x Nn matrix (for multi-logistic loss)  
%              initial guess  
%         param: struct  
%            param.lambda (regularization parameter)  
%            param.numThreads (optional, number of threads for exploiting  
%                multi-core / multi-cpus. By default, it takes the value -1,  
%                which automatically selects all the available CPUs/cores).  
%            param.itermax (optional, maximum number of iterations, 100 by default)  
%            param.tol (optional, tolerance for stopping criteration, which is a relative duality gap  
%                if it is available, or a relative change of parameters).  
%  
% Output:  W:  double dense p x n matrix   
  
% Author: Julien Mairal, 2013

The following piece of code illustrates how to use this function.

format compact;  
randn('seed',0);  
param.numThreads=-1; % all cores (-1 by default)  
param.lambda=0.05; % regularization parameter  
  
X=randn(100,200);  
X=X-repmat(mean(X),[size(X,1) 1]);  
X=mexNormalize(X);  
Y=randn(100,1);  
Y=Y-repmat(mean(Y),[size(Y,1) 1]);  
Y=mexNormalize(Y);  
W0=zeros(size(X,2),size(Y,2));  
% Regression experiments   
% 100 regression problems with the same design matrix X.  
fprintf('\nVarious regression experiments\n');  
fprintf('\nRidge Regression with conjugate gradient solver\n');  
tic  
[W]=mexRidgeRegression(Y,X,W0,param);  
t=toc

### 4.4  Function mexLasso

This is a fast implementation of the LARS algorithm [9] (variant for solving the Lasso) for solving the Lasso or Elastic-Net. Given a matrix of signals X=[x^1^,…,x^n^] in ℝ^m × n^ and a dictionary D in ℝ^m × p^, depending on the input parameters, the algorithm returns a matrix of coefficients A=[α^1^,…,α^n^] in ℝ^p × n^ such that for every column x of X, the corresponding column α of A is the solution of

min

α ∈ ℝ^p^

||x−Dα||_2_^2^   s.t.   ||α||_1_ ≤ λ,
    (8)

or

min

α ∈ ℝ^p^

||α||_1_   s.t.   ||x−Dα||_2_^2^ ≤ λ, 
    (9)

or

|  |  |  |  |  |  |  |
| --- | --- | --- | --- | --- | --- | --- |
|  | min; α ∈ ℝ^p^ |  | 1; 2 | \|\|x−Dα\|\|_2_^2^ + λ \|\|α\|\|_1_ + | λ_2_; 2 | \|\|α\|\|_2_^2^.      (10) |

For efficiency reasons, the method first compute the covariance matrix D^T^D, then
for each signal, it computes D^T^x and performs the decomposition with a
Cholesky-based algorithm (see [9] for instance). The implementation
has also an option to add positivity constraints on the solutions
α. When the solution is very sparse and the problem size is
reasonable, this approach can be very efficient. Moreover, it gives the
solution with an exact precision, and its performance does not depend on the
correlation of the dictionary elements, except when the solution is not unique
(the algorithm breaks in this case).

Note that mexLasso can return the whole regularization path of the first signal x_1_
and can handle implicitely the matrix D if the quantities D^T^D and D^T^x are passed
as an argument, see below:

%   
% Usage:  [A [path]]=mexLasso(X,D,param);  
%  or:    [A [path]]=mexLasso(X,Q,q,param);  
%  
% Name: mexLasso  
%  
% Description: mexLasso is an efficient implementation of the  
%     homotopy-LARS algorithm for solving the Lasso.   
%       
%     if the function is called this way [A [path]]=mexLasso(X,D,param),  
%     it aims at addressing the following problems  
%     for all columns x of X, it computes one column alpha of A  
%     that solves  
%       1) when param.mode=0  
%         min\_{alpha} ||x-Dalpha||\_2^2 s.t. ||alpha||\_1 <= lambda  
%       2) when param.mode=1  
%         min\_{alpha} ||alpha||\_1 s.t. ||x-Dalpha||\_2^2 <= lambda  
%       3) when param.mode=2  
%         min\_{alpha} 0.5||x-Dalpha||\_2^2 + lambda||alpha||\_1 +0.5 lambda2||alpha||\_2^2  
%  
%     if the function is called this way [A [path]]=mexLasso(X,Q,q,param),  
%     it solves the above optimisation problem, when Q=D'D and q=D'x.  
%  
%     Possibly, when param.pos=true, it solves the previous problems  
%     with positivity constraints on the vectors alpha  
%  
% Inputs: X:  double m x n matrix   (input signals)  
%               m is the signal size  
%               n is the number of signals to decompose  
%         D:  double m x p matrix   (dictionary)  
%               p is the number of elements in the dictionary  
%         param: struct  
%               param.lambda  (parameter)  
%               param.lambda2  (optional parameter for solving the Elastic-Net)  
%                              for mode=0 and mode=1, it adds a ridge on the Gram Matrix  
%               param.L (optional), maximum number of steps of the homotopy algorithm (can  
%                        be used as a stopping criterion)  
%               param.pos (optional, adds non-negativity constraints on the  
%                 coefficients, false by default)  
%               param.mode (see above, by default: 2)  
%               param.numThreads (optional, number of threads for exploiting  
%                 multi-core / multi-cpus. By default, it takes the value -1,  
%                 which automatically selects all the available CPUs/cores).  
%               param.cholesky (optional, default false),  choose between Cholesky   
%                 implementation or one based on the matrix inversion Lemma  
%               param.ols (optional, default false), perform an orthogonal projection  
%                 before returning the solution.  
%               param.max\_length\_path (optional) maximum length of the path, by default 4\*p  
%  
% Output: A: double sparse p x n matrix (output coefficients)  
%         path: optional,  returns the regularisation path for the first signal  
%  
% Note: this function admits a few experimental usages, which have not  
%     been extensively tested:  
%         - single precision setting (even though the output alpha is double   
%           precision)  
%  
% Author: Julien Mairal, 2009

The following piece of code illustrates how to use this function.

clear all;  
  
randn('seed',0);  
fprintf('test mexLasso\n');  
%%%%%%%%%%%%%%%%%%%%%%%%%%%%%%%%%%%%%%%%%%%%%%  
% Decomposition of a large number of signals  
%%%%%%%%%%%%%%%%%%%%%%%%%%%%%%%%%%%%%%%%%%%%%%  
% Data are generated  
X=randn(100,100000);  
X=X./repmat(sqrt(sum(X.^2)),[size(X,1) 1]);  
D=randn(100,200);  
D=D./repmat(sqrt(sum(D.^2)),[size(D,1) 1]);  
  
% parameter of the optimization procedure are chosen  
%param.L=20; % not more than 20 non-zeros coefficients (default: min(size(D,1),size(D,2)))  
param.lambda=0.15; % not more than 20 non-zeros coefficients  
param.numThreads=-1; % number of processors/cores to use; the default choice is -1  
                     % and uses all the cores of the machine  
param.mode=2;        % penalized formulation  
  
tic  
alpha=mexLasso(X,D,param);  
t=toc  
fprintf('%f signals processed per second\n',size(X,2)/t);  
  
%%%%%%%%%%%%%%%%%%%%%%%%%%%%%%%%%%%%%%%%%%  
% Regularization path of a single signal   
%%%%%%%%%%%%%%%%%%%%%%%%%%%%%%%%%%%%%%%%%%  
X=randn(64,1);  
D=randn(64,10);  
D=D./repmat(sqrt(sum(D.^2)),[size(D,1) 1]);  
param.lambda=0;  
[alpha path]=mexLasso(X,D,param);

### 4.5  Function mexLassoWeighted

This is a fast implementation of a weighted version of LARS [9]. Given a matrix of signals X=[x^1^,…,x^n^] in ℝ^m × n^, a matrix of weights W=[w^1^,…,w^n^] ∈ ℝ^p × n^, and a dictionary D in ℝ^m × p^, depending on the input parameters, the algorithm returns a matrix of coefficients A=[α^1^,…,α^n^] in ℝ^p × n^,
such that for every column x of X, w of W, it computes a column α of A, which is the solution of

min

α ∈ ℝ^p^

||x−Dα||_2_^2^   s.t.   ||diag(w)α||_1_ ≤ λ,
    (11)

or

min

α ∈ ℝ^p^

||diag(w)α||_1_   s.t.   ||x−Dα||_2_^2^ ≤ λ,
    (12)

or

min

α ∈ ℝ^p^

1

2

||x−Dα||_2_^2^ + λ ||diag(w)α||_1_.
    (13)

The implementation has also an option to add positivity constraints on
the solutions α. This function is potentially useful for
implementing efficiently the randomized Lasso of [28], or reweighted-ℓ_1_ schemes [4].

%   
% Usage:  A=mexLassoWeighted(X,D,W,param);  
%  
% Name: mexLassoWeighted.    
%  
% WARNING: This function has not been tested intensively  
%  
% Description: mexLassoWeighted is an efficient implementation of the  
%     LARS algorithm for solving the weighted Lasso. It is optimized  
%     for solving a large number of small or medium-sized   
%     decomposition problem (and not for a single large one).  
%     It first computes the Gram matrix D'D and then perform  
%     a Cholesky-based OMP of the input signals in parallel.  
%     For all columns x of X, and w of W, it computes one column alpha of A  
%     which is the solution of  
%       1) when param.mode=0  
%         min\_{alpha} ||x-Dalpha||\_2^2   s.t.    
%                                     ||diag(w)alpha||\_1 <= lambda  
%       2) when param.mode=1  
%         min\_{alpha} ||diag(w)alpha||\_1  s.t.  
%                                        ||x-Dalpha||\_2^2 <= lambda  
%       3) when param.mode=2  
%         min\_{alpha} 0.5||x-Dalpha||\_2^2  +    
%                                         lambda||diag(w)alpha||\_1   
%     Possibly, when param.pos=true, it solves the previous problems  
%     with positivity constraints on the vectors alpha  
%  
% Inputs: X:  double m x n matrix   (input signals)  
%               m is the signal size  
%               n is the number of signals to decompose  
%         D:  double m x p matrix   (dictionary)  
%               p is the number of elements in the dictionary  
%         W:  double p x n matrix   (weights)  
%         param: struct  
%            param.lambda  (parameter)  
%            param.L (optional, maximum number of elements of each   
%            decomposition)  
%            param.pos (optional, adds positivity constraints on the  
%            coefficients, false by default)  
%            param.mode (see above, by default: 2)  
%            param.numThreads (optional, number of threads for exploiting  
%            multi-core / multi-cpus. By default, it takes the value -1,  
%            which automatically selects all the available CPUs/cores).  
%  
% Output:  A: double sparse p x n matrix (output coefficients)  
%  
% Note: this function admits a few experimental usages, which have not  
%     been extensively tested:  
%         - single precision setting (even though the output alpha is double   
%           precision)  
%  
% Author: Julien Mairal, 2009

The following piece of code illustrates how to use this function.

clear all;  
  
fprintf('test Lasso weighted\n');  
randn('seed',0);  
% Data are generated  
X=randn(64,10000);  
X=X./repmat(sqrt(sum(X.^2)),[size(X,1) 1]);  
D=randn(64,256);  
D=D./repmat(sqrt(sum(D.^2)),[size(D,1) 1]);  
  
% parameter of the optimization procedure are chosen  
param.L=20; % not more than 20 non-zeros coefficients (default: min(size(D,1),size(D,2)))  
param.lambda=0.15; % not more than 20 non-zeros coefficients  
param.numThreads=8; % number of processors/cores to use; the default choice is -1  
                    % and uses all the cores of the machine  
param.mode=2;       % penalized formulation  
  
W=rand(size(D,2),size(X,2));  
  
tic  
alpha=mexLassoWeighted(X,D,W,param);  
t=toc;  
toc  
  
fprintf('%f signals processed per second\n',size(X,2)/t);

### 4.6  Function mexLassoMask

This is a variant of mexLasso with the possibility of adding a mask B=[β^1^,…,β^n^], as in mexOMPMask.
For every column x of X, β of B, it computes a column α of A, which is the solution of

min

α ∈ ℝ^p^

||diag(β)(x−Dα)||_2_^2^   s.t.   ||α||_1_ ≤ λ,
    (14)

or

|  |  |  |  |  |
| --- | --- | --- | --- | --- |
|  | min; α ∈ ℝ^p^ | \|\|α\|\|_1_   s.t.   \|\|diag(β)(x−Dα)\|\|_2_^2^ ≤ λ | \|\|β\|\|_0_; m | ,      (15) |

or

|  |  |  |  |  |  |  |  |  |
| --- | --- | --- | --- | --- | --- | --- | --- | --- |
|  | min; α ∈ ℝ^p^ |  | 1; 2 | \|\|diag(β)(x−Dα)\|\|_2_^2^ + λ | \|\|β\|\|_0_; m | \|\|α\|\|_1_ + | λ_2_; 2 | \|\|α\|\|_2_^2^.      (16) |

%   
% Usage:   A=mexLassoMask(X,D,B,param);  
%  
% Name: mexLassoMask  
%  
% Description: mexLasso is a variant of mexLasso that handles  
%     binary masks. It aims at addressing the following problems  
%     for all columns x of X, and beta of B, it computes one column alpha of A  
%     that solves  
%       1) when param.mode=0  
%         min\_{alpha} ||diag(beta)(x-Dalpha)||\_2^2 s.t. ||alpha||\_1 <= lambda  
%       2) when param.mode=1  
%         min\_{alpha} ||alpha||\_1 s.t. ||diag(beta)(x-Dalpha)||\_2^2   
%                                                              <= lambda\*||beta||\_0/m  
%       3) when param.mode=2  
%         min\_{alpha} 0.5||diag(beta)(x-Dalpha)||\_2^2 +  
%                                                 lambda\*(||beta||\_0/m)\*||alpha||\_1 +  
%                                                 (lambda2/2)||alpha||\_2^2  
%     Possibly, when param.pos=true, it solves the previous problems  
%     with positivity constraints on the vectors alpha  
%  
% Inputs: X:  double m x n matrix   (input signals)  
%               m is the signal size  
%               n is the number of signals to decompose  
%         D:  double m x p matrix   (dictionary)  
%               p is the number of elements in the dictionary  
%         B:  boolean m x n matrix   (mask)  
%               p is the number of elements in the dictionary  
%         param: struct  
%               param.lambda  (parameter)  
%               param.L (optional, maximum number of elements of each   
%                 decomposition)  
%               param.pos (optional, adds positivity constraints on the  
%                 coefficients, false by default)  
%               param.mode (see above, by default: 2)  
%               param.lambda2  (optional parameter for solving the Elastic-Net)  
%                              for mode=0 and mode=1, it adds a ridge on the Gram Matrix  
%               param.numThreads (optional, number of threads for exploiting  
%                 multi-core / multi-cpus. By default, it takes the value -1,  
%                 which automatically selects all the available CPUs/cores).  
%  
% Output: A: double sparse p x n matrix (output coefficients)  
%  
% Note: this function admits a few experimental usages, which have not  
%     been extensively tested:  
%         - single precision setting (even though the output alpha is double   
%           precision)  
%  
% Author: Julien Mairal, 2010

The following piece of code illustrates how to use this function.

clear all;  
  
randn('seed',0);  
fprintf('test mexLasso\n');  
  
%%%%%%%%%%%%%%%%%%%%%%%%%%%%%%%%%%%%%%%%%%%%%%  
% Decomposition of a large number of signals  
%%%%%%%%%%%%%%%%%%%%%%%%%%%%%%%%%%%%%%%%%%%%%%  
% Data are generated  
X=randn(100,100);  
X=X./repmat(sqrt(sum(X.^2)),[size(X,1) 1]);  
D=randn(100,20);  
D=D./repmat(sqrt(sum(D.^2)),[size(D,1) 1]);  
mask=(X > 0); % generating a binary mask  
  
% parameter of the optimization procedure are chosen  
%param.L=20; % not more than 20 non-zeros coefficients (default: min(size(D,1),size(D,2)))  
param.lambda=0.15; % not more than 20 non-zeros coefficients  
param.numThreads=-1; % number of processors/cores to use; the default choice is -1  
                     % and uses all the cores of the machine  
param.mode=2;        % penalized formulation  
  
tic  
alpha=mexLassoMask(X,D,mask,param);  
t=toc;  
toc  
  
fprintf('%f signals processed per second\n',size(X,2)/t);

### 4.7  Function mexCD

Coordinate-descent approach for solving Eq. (10) and
Eq. (9). Note that unlike mexLasso, it is not implemented to solve the Elastic-Net formulation.
To solve Eq. (9), the algorithm solves a
sequence of problems of the form (10) using simple heuristics.
Coordinate descent is very simple and in practice very powerful. It performs
better when the correlation between the dictionary elements is small.

%   
% Usage:   A=mexCD(X,D,A0,param);  
%  
% Name: mexCD  
%  
% Description: mexCD addresses l1-decomposition problem with a   
%     coordinate descent type of approach.  
%     It is optimized for solving a large number of small or medium-sized   
%     decomposition problem (and not for a single large one).  
%     It first computes the Gram matrix D'D.  
%     This method is particularly well adapted when there is low   
%     correlation between the dictionary elements and when one can benefit   
%     from a warm restart.  
%     It aims at addressing the two following problems  
%     for all columns x of X, it computes a column alpha of A such that  
%       2) when param.mode=1  
%         min\_{alpha} ||alpha||\_1 s.t. ||x-Dalpha||\_2^2 <= lambda  
%         For this constraint setting, the method solves a sequence of   
%         penalized problems (corresponding to param.mode=2) and looks  
%         for the corresponding Lagrange multplier with a simple but  
%         efficient heuristic.  
%       3) when param.mode=2  
%         min\_{alpha} 0.5||x-Dalpha||\_2^2 + lambda||alpha||\_1   
%  
% Inputs: X:  double m x n matrix   (input signals)  
%               m is the signal size  
%               n is the number of signals to decompose  
%         D:  double m x p matrix   (dictionary)  
%               p is the number of elements in the dictionary  
%               All the columns of D should have unit-norm !  
%         A0:  double sparse p x n matrix   (initial guess)  
%         param: struct  
%            param.lambda  (parameter)  
%            param.mode (optional, see above, by default 2)  
%            param.itermax (maximum number of iterations)  
%            param.tol (tolerance parameter)  
%            param.numThreads (optional, number of threads for exploiting  
%            multi-core / multi-cpus. By default, it takes the value -1,  
%            which automatically selects all the available CPUs/cores).  
%  
% Output: A: double sparse p x n matrix (output coefficients)  
%  
% Note: this function admits a few experimental usages, which have not  
%     been extensively tested:  
%         - single precision setting (even though the output alpha   
%           is double precision)  
%  
% Author: Julien Mairal, 2009

The following piece of code illustrates how to use this function.

clear all;  
  
fprintf('test mexCD\n');  
randn('seed',0);  
% Data are generated  
X=randn(64,100);  
X=X./repmat(sqrt(sum(X.^2)),[size(X,1) 1]);  
D=randn(64,100);  
D=D./repmat(sqrt(sum(D.^2)),[size(D,1) 1]);  
  
% parameter of the optimization procedure are chosen  
param.lambda=0.015; % not more than 20 non-zeros coefficients  
param.numThreads=4; % number of processors/cores to use; the default choice is -1  
param.mode=2;       % penalized formulation  
  
tic  
alpha=mexLasso(X,D,param);  
t=toc;  
toc  
E=mean(0.5\*sum((X-D\*alpha).^2)+param.lambda\*sum(abs(alpha)));  
fprintf('%f signals processed per second for LARS\n',size(X,2)/t);  
fprintf('Objective function for LARS: %g\n',E);  
  
param.tol=0.001;  
param.itermax=1000;  
tic  
alpha2=mexCD(X,D,sparse(size(alpha,1),size(alpha,2)),param);  
t=toc;  
toc  
  
fprintf('%f signals processed per second for CD\n',size(X,2)/t);  
E=mean(0.5\*sum((X-D\*alpha2).^2)+param.lambda\*sum(abs(alpha2)));  
fprintf('Objective function for CD: %g\n',E);  
fprintf('With Random Design, CD can be much faster than LARS\n');

### 4.8  Function mexSOMP

This is a fast implementation of the Simultaneous Orthogonal Matching Pursuit algorithm. Given a set of matrices X=[X^1^,…,X^n^] in ℝ^m × N^, where the X^i^’s are in ℝ^m × n_i_^, and a dictionary D in ℝ^m × p^, the algorithm returns a matrix of coefficients A=[A^1^,…,A^n^] in ℝ^p × N^ which is an approximate solution of the following NP-hard problem

|  |  |  |
| --- | --- | --- |
| ∀ i | min; A^i^ ∈ ℝ^p × n_i_^ | \|\|X^i^−DA^i^\|\|_F_^2^   s.t.   \|\|A^i^\|\|_0,∞_ ≤ L.     (17) |

or

|  |  |  |
| --- | --- | --- |
| ∀ i | min; A^i^ ∈ ℝ^p × n_i_^ | \|\|A^i^\|\|_0,∞_   s.t.   \|\|X^i^−DA^i^\|\|_F_^2^ ≤ ε n_i_.     (18) |

To be efficient, the method first compute the covariance matrix D^T^D, then for each signal, it computes D^T^X^i^ and performs the decomposition with a Cholesky-based algorithm.

%   
% Usage:   alpha=mexSOMP(X,D,list\_groups,param);  
%  
% Name: mexSOMP  
%     (this function has not been intensively tested).  
%  
% Description: mexSOMP is an efficient implementation of a  
%     Simultaneous Orthogonal Matching Pursuit algorithm. It is optimized  
%     for solving a large number of small or medium-sized   
%     decomposition problem (and not for a single large one).  
%     It first computes the Gram matrix D'D and then perform  
%     a Cholesky-based OMP of the input signals in parallel.  
%     It aims at addressing the following NP-hard problem  
%  
%     X is a matrix structured in groups of signals, which we denote  
%     by X=[X\_1,...,X\_n]  
%       
%     for all matrices X\_i of X,   
%         min\_{A\_i} ||A\_i||\_{0,infty}  s.t  ||X\_i-D A\_i||\_2^2 <= eps\*n\_i  
%         where n\_i is the number of columns of X\_i  
%  
%         or  
%  
%         min\_{A\_i} ||X\_i-D A\_i||\_2^2  s.t. ||A\_i||\_{0,infty} <= L  
%           
%  
% Inputs: X:  double m x N matrix   (input signals)  
%            m is the signal size  
%            N is the total number of signals   
%         D:  double m x p matrix   (dictionary)  
%            p is the number of elements in the dictionary  
%            All the columns of D should have unit-norm !  
%         list\_groups : int32 vector containing the indices (starting at 0)  
%            of the first elements of each groups.  
%         param: struct  
%            param.L (maximum number of elements in each decomposition)  
%            param.eps (threshold on the squared l2-norm of the residual  
%            param.numThreads (optional, number of threads for exploiting  
%            multi-core / multi-cpus. By default, it takes the value -1,  
%            which automatically selects all the available CPUs/cores).  
%  
% Output: alpha: double sparse p x N matrix (output coefficients)  
%  
% Note: this function admits a few experimental usages, which have not  
%     been extensively tested:  
%      - single precision setting (even though the output alpha is double   
%        precision)  
%  
% Author: Julien Mairal, 2010

The following piece of code illustrates how to use this function.

clear all;  
randn('seed',0);  
  
fprintf('test mexSOMP\n');  
  
%%%%%%%%%%%%%%%%%%%%%%%%%%%%%%%%%%%%%%%%%%%%%%  
% Decomposition of a large number of groups   
%%%%%%%%%%%%%%%%%%%%%%%%%%%%%%%%%%%%%%%%%%%%%%  
X=randn(64,10000);  
D=randn(64,200);  
D=D./repmat(sqrt(sum(D.^2)),[size(D,1) 1]);  
ind\_groups=int32(0:10:9999); % indices of the first signals in each group  
  
% parameter of the optimization procedure are chosen  
param.L=10; % not more than 10 non-zeros coefficients  
param.eps=0.1; % squared norm of the residual should be less than 0.1  
param.numThreads=-1; % number of processors/cores to use; the default choice is -1  
                    % and uses all the cores of the machine  
tic  
alpha=mexSOMP(X,D,ind\_groups,param);  
t=toc  
fprintf('%f signals processed per second\n',size(X,2)/t);

### 4.9  Function mexL1L2BCD

This is a fast implementation of a simultaneous signal decomposition formulation. Given a set of matrices X=[X^1^,…,X^n^] in ℝ^m × N^, where the X^i^’s are in ℝ^m × n_i_^, and a dictionary D in ℝ^m × p^, the algorithm returns a matrix of coefficients A=[A^1^,…,A^n^] in ℝ^p × N^ which is an approximate solution of the following NP-hard problem

|  |  |  |  |  |
| --- | --- | --- | --- | --- |
| ∀ i | min; A^i^ ∈ ℝ^p × n_i_^ | \|\|X^i^−DA^i^\|\|_F_^2^   s.t.   \|\|A^i^\|\|_1,2_ ≤ | λ; n_i_ | .     (19) |

or

|  |  |  |
| --- | --- | --- |
| ∀ i | min; A^i^ ∈ ℝ^p × n_i_^ | \|\|A^i^\|\|_1,2_   s.t.   \|\|X^i^−DA^i^\|\|_F_^2^ ≤ λ n_i_.     (20) |

To be efficient, the method first compute the covariance matrix D^T^D, then for each signal, it computes D^T^X^i^ and performs the decomposition with a Cholesky-based algorithm.

%   
% Usage:   alpha=mexL1L2BCD(X,D,alpha0,list\_groups,param);  
%  
% Name: mexL1L2BCD  
%     (this function has not been intensively tested).  
%  
% Description: mexL1L2BCD is a solver for a   
%     Simultaneous signal decomposition formulation based on block   
%     coordinate descent.  
%  
%     X is a matrix structured in groups of signals, which we denote  
%     by X=[X\_1,...,X\_n]  
%       
%     if param.mode=2, it solves  
%         for all matrices X\_i of X,   
%         min\_{A\_i} 0.5||X\_i-D A\_i||\_2^2 + lambda/sqrt(n\_i)||A\_i||\_{1,2}    
%         where n\_i is the number of columns of X\_i  
%     if param.mode=1, it solves  
%         min\_{A\_i} ||A\_i||\_{1,2} s.t. ||X\_i-D A\_i||\_2^2  <= n\_i lambda  
%           
%  
% Inputs: X:  double m x N matrix   (input signals)  
%            m is the signal size  
%            N is the total number of signals   
%         D:  double m x p matrix   (dictionary)  
%            p is the number of elements in the dictionary  
%         alpha0: double dense p x N matrix (initial solution)  
%         list\_groups : int32 vector containing the indices (starting at 0)  
%            of the first elements of each groups.  
%         param: struct  
%            param.lambda (regularization parameter)  
%            param.mode (see above, by default 2)  
%            param.itermax (maximum number of iterations, by default 100)  
%            param.tol (tolerance parameter, by default 0.001)  
%            param.numThreads (optional, number of threads for exploiting  
%            multi-core / multi-cpus. By default, it takes the value -1,  
%            which automatically selects all the available CPUs/cores).  
%  
% Output: alpha: double sparse p x N matrix (output coefficients)  
%  
% Note: this function admits a few experimental usages, which have not  
%     been extensively tested:  
%      - single precision setting (even though the output alpha is double   
%        precision)  
%  
% Author: Julien Mairal, 2010

The following piece of code illustrates how to use this function.

clear all;  
randn('seed',0);  
  
fprintf('test mexL1L2BCD\n');  
%%%%%%%%%%%%%%%%%%%%%%%%%%%%%%%%%%%%%%%%%%%%%%  
% Decomposition of a large number of groups   
%%%%%%%%%%%%%%%%%%%%%%%%%%%%%%%%%%%%%%%%%%%%%%  
X=randn(64,100);  
D=randn(64,200);  
D=D./repmat(sqrt(sum(D.^2)),[size(D,1) 1]);  
ind\_groups=int32(0:10:size(X,2)-1); % indices of the first signals in each group  
  
% parameter of the optimization procedure are chosen  
param.itermax=100;  
param.tol=1e-3;  
param.mode=2; % penalty mode  
param.lambda=0.15; % squared norm of the residual should be less than 0.1  
param.numThreads=-1; % number of processors/cores to use; the default choice is -1  
                    % and uses all the cores of the machine  
tic  
alpha0=zeros(size(D,2),size(X,2));  
alpha=mexL1L2BCD(X,D,alpha0,ind\_groups,param);  
t=toc  
fprintf('%f signals processed per second\n',size(X,2)/t);

### 4.10  Function mexSparseProject

This is a multi-purpose function, implementing fast algorithms for projecting
on convex sets, but it also solves the fused lasso signal approximation
problem. The proposed method is detailed in [21]. The main problems
addressed by this function are the following: Given a matrix
U=[u_1_,…,u_n_] in ℝ^m × n^, it finds a matrix
V=[v_1_,…,v_n_] in ℝ^m × n^ so that for all column u of U,
it computes a column v of V solving

min

v ∈ ℝ^m^

||u−v||_2_^2^    s.t.   ||v||_1_ ≤ τ,
    (21)

or

min

v ∈ ℝ^m^

||u−v||_2_^2^    s.t.   λ_1_||v||_1_ +λ_2_||v||_2_^2^≤ τ,
    (22)

or

min

v ∈ ℝ^m^

||u−v||_2_^2^    s.t.   λ_1_||v||_1_ +λ_2_||v||_2_^2^+ λ_3_ FL(v) ≤ τ,
    (23)

or

min

v ∈ ℝ^m^

1

2

||u−v||_2_^2^ + λ_1_||v||_1_ +λ_2_||v||_2_^2^+ λ_3_ FL(v).
    (24)

Note that for the two last cases, the method performs a small approximation.
The method follows the regularization path, goes from one kink to another, and
stop whenever the constraint is not satisfied anymore. The solution returned
by the algorithm is the one obtained at the last kink of the regularization path,
which is in practice close, but not exactly the same as the solution.
This will be corrected in a future release of the toolbox.

%   
% Usage:  V=mexSparseProject(U,param);  
%  
% Name: mexSparseProject  
%  
% Description: mexSparseProject solves various optimization   
%     problems, including projections on a few convex sets.  
%     It aims at addressing the following problems  
%     for all columns u of U in parallel  
%       1) when param.mode=1 (projection on the l1-ball)  
%           min\_v ||u-v||\_2^2  s.t.  ||v||\_1 <= thrs  
%       2) when param.mode=2  
%           min\_v ||u-v||\_2^2  s.t. ||v||\_2^2 + lamuda1||v||\_1 <= thrs  
%       3) when param.mode=3  
%           min\_v ||u-v||\_2^2  s.t  ||v||\_1 + 0.5lamuda1||v||\_2^2 <= thrs   
%       4) when param.mode=4  
%           min\_v 0.5||u-v||\_2^2 + lamuda1||v||\_1  s.t  ||v||\_2^2 <= thrs  
%       5) when param.mode=5  
%           min\_v 0.5||u-v||\_2^2 + lamuda1||v||\_1 +lamuda2 FL(v) + ...   
%                                                   0.5lamuda\_3 ||v||\_2^2  
%          where FL denotes a "fused lasso" regularization term.  
%       6) when param.mode=6  
%          min\_v ||u-v||\_2^2 s.t lamuda1||v||\_1 +lamuda2 FL(v) + ...  
%                                             0.5lamuda3||v||\_2^2 <= thrs  
%             
%        When param.pos=true and param.mode <= 4,  
%        it solves the previous problems with positivity constraints   
%  
% Inputs: U:  double m x n matrix   (input signals)  
%               m is the signal size  
%               n is the number of signals to project  
%         param: struct  
%           param.thrs (parameter)  
%           param.lambda1 (parameter)  
%           param.lambda2 (parameter)  
%           param.lambda3 (parameter)  
%           param.mode (see above)  
%           param.pos (optional, false by default)  
%           param.numThreads (optional, number of threads for exploiting  
%             multi-core / multi-cpus. By default, it takes the value -1,  
%             which automatically selects all the available CPUs/cores).  
%  
% Output: V: double m x n matrix (output matrix)  
%  
% Note: this function admits a few experimental usages, which have not  
%     been extensively tested:  
%         - single precision setting   
%  
% Author: Julien Mairal, 2009

The following piece of code illustrates how to use this function.

clear all;  
randn('seed',0);  
% Data are generated  
X=randn(20000,100);  
X=X./repmat(sqrt(sum(X.^2)),[size(X,1) 1]);  
  
% parameter of the optimization procedure are chosen  
param.numThreads=-1; % number of processors/cores to use; the default choice is -1  
                    % and uses all the cores of the machine  
  
param.pos=0;  
param.mode=1;       % projection on the l1 ball  
param.thrs=2;  
tic  
X1=mexSparseProject(X,param);  
t=toc;  
toc  
fprintf('%f signals of size %d projected per second\n',size(X,2)/t,size(X,1));  
fprintf('Checking constraint: %f, %f\n',min(sum(abs(X1))),max(sum(abs(X1))));  
  
  
param.mode=2;       % projection on the Elastic-Net  
param.lambda1=0.15;  
  
tic  
X1=mexSparseProject(X,param);  
t=toc;  
toc  
fprintf('%f signals of size %d projected per second\n',size(X,2)/t,size(X,1));  
constraints=sum((X1.^2))+param.lambda1\*sum(abs(X1));  
fprintf('Checking constraint: %f, %f\n',min(constraints),max(constraints));  
  
param.mode=6;       % projection on the FLSA  
param.lambda1=0.7;  
param.lambda2=0.7;  
param.lambda3=1.0;  
  
X=rand(2000,100);  
X=X./repmat(sqrt(sum(X.^2)),[size(X,1) 1]);  
  
tic  
X1=mexSparseProject(X,param);  
t=toc;  
toc  
fprintf('%f signals of size %d projected per second\n',size(X,2)/t,size(X,1));  
constraints=0.5\*param.lambda3\*sum(X1.^2)+param.lambda1\*sum(abs(X1))+param.lambda2\*sum(abs(X1(2:end,:)-X1(1:end-1,:)));  
fprintf('Checking constraint: %f, %f\n',mean(constraints),max(constraints));  
fprintf('Projection is approximate (stops at a kink)\n',mean(constraints),max(constraints));  
  
param.mode=6;       % projection on the FLSA  
param.lambda1=0.7;  
param.lambda2=0.7;  
param.lambda3=1.0;  
  
X=rand(2000,100);  
X=X./repmat(sqrt(sum(X.^2)),[size(X,1) 1]);  
  
tic  
X1=mexSparseProject(X,param);  
t=toc;  
toc  
fprintf('%f signals of size %d projected per second\n',size(X,2)/t,size(X,1));  
constraints=0.5\*param.lambda3\*sum(X1.^2)+param.lambda1\*sum(abs(X1))+param.lambda2\*sum(abs(X1(2:end,:)-X1(1:end-1,:)));  
fprintf('Checking constraint: %f, %f\n',mean(constraints),max(constraints));  
fprintf('Projection is approximate (stops at a kink)\n',mean(constraints),max(constraints));

### 4.11  Function mexDecompSimplex

The function implements an active-set algorithm [37] for solving

|  |  |  |  |  |  |  |
| --- | --- | --- | --- | --- | --- | --- |
|  | min; α ∈ ℝ^p^ |  | 1; 2 | \|\|x−Dα\|\|_2_^2^   s.t.   α ≥ 0   and | p; ∑; j=1 | α[j] = 1. |

%   
% Usage:  [A]=mexDecompSimplex(X,Z,param);  
%  
% Name: mexDecompSimplex  
%  
% documentation to appear soon  
%  
% Inputs: X:  double m x n matrix   (input signals)  
%               m is the signal size  
%               n is the number of signals to decompose  
% Output: Z: double %  
%  
% Author: Yuansi Chen and Julien Mairal, 2014

---
