# Supplementary material for: Multitask fMRI Data Classification via Group-Wise Hybrid Temporal and Spatial Sparse Representations
Source: eNeuro. 2022 Jun 3;9(3):ENEURO.0478-21.2022. doi: 10.1523/ENEURO.0478-21.2022 (PMC9186416; doi:10.1523/ENEURO.0478-21.2022)
Supplement: Extended Data 1 — The code. Download Extended Data 1, ZIP file. [file enu-eN-MNT-0478-21-s02.zip › code/tools/spams-matlab.zip!/spams-matlab/doc/html/doc_spams006.html]

Proximal Toolbox


---

## 5  Proximal Toolbox

- Regularization Functions
- Function mexProximalFlat
- Function mexProximalTree
- Function mexProximalGraph
- Function mexProximalPathCoding
- Function mexEvalPathCoding
- Problems Addressed
- Function mexFistaFlat
- Function mexFistaTree
- Function mexFistaGraph
- Function mexFistaPathCoding
- Function solverPoisson
- Function mexIncrementalProx
- Function mexStochasticProx

The previous toolbox we have presented is well
adapted for solving a large number of small and medium-scale sparse
decomposition problems with the square loss, which is typical from the
classical dictionary learning framework. We now present
a new software package that is adapted for solving a wide range of
possibly large-scale learning problems, with several combinations of losses and
regularization terms. The method implements the proximal methods
of [1], and includes the proximal solvers for the tree-structured
regularization of [15], and the solver of [22] for
general structured sparse regularization.
The solver for structured sparse regularization norms includes a C++ max-flow
implementation of the push-relabel algorithm of [13], with
heuristics proposed by [5].

This implementation also provides robust stopping criteria based on
*duality gaps*, which are presented in Appendix A. It can handle intercepts (unregularized variables). The general formulation that our software
can solve take the form

|  |  |  |  |
| --- | --- | --- | --- |
| min; w ∈ ℝ^p^ | [g(w) | ▵; = | f(w) + λψ(w)], |

where f is a smooth loss function and ψ is a regularization function.
When one optimizes a matrix W in ℝ^p × r^ instead of
a vector w in ℝ^p^, we will write

|  |  |  |  |
| --- | --- | --- | --- |
| min; W ∈ ℝ^p × r^ | [g(W) | ▵; = | f(W) + λψ(W)]. |

Note that the software can possibly handle nonnegativity constraints.

We start by presenting the type of regularization implemented in the software

### 5.1  Regularization Functions

Our software can handle the following regularization functions ψ for vectors w in ℝ^p^:

- The Tikhonov regularization: ψ(w) =^▵^ 1/2||w||_2_^2^.
- The ℓ_1_-norm: ψ(w) =^▵^ ||w||_1_.
- The Elastic-Net: ψ(w) =^▵^ ||w||_1_+γ||w||_2_^2^.
- The Fused-Lasso: ψ(w) =^▵^ ||w||_1_+γ||w||_2_^2^+γ_2_∑_i=1_^p−1^|w_i+1_−w_i_|.
- The group Lasso: ψ(w) =^▵^ ∑_g ∈ G_ η_g_ ||w_g_||_2_, where G are groups of variables.
- The group Lasso with ℓ_∞_-norm: ψ(w) =^▵^ ∑_g ∈ G_ η_g_ ||w_g_||_∞_, where G are groups of variables.
- The sparse group Lasso: same as above but with an additional ℓ_1_ term.
- The tree-structured sum of ℓ_2_-norms: ψ(w) =^▵^ ∑_g ∈ G_ η_g_ ||w_g_||_2_, where G is a tree-structured set of groups [15], and the η_g_ are positive weights.
- The tree-structured sum of ℓ_∞_-norms: ψ(w) =^▵^ ∑_g ∈ G_ η_g_ ||w_g_||_∞_. See [15]
- General sum of ℓ_∞_-norms: ψ(w) =^▵^ ∑_g ∈ G_ η_g_ ||w_g_||_∞_, where no assumption are made on the groups G.
- The path-coding penalties of [24].
- the ℓ_1_-constraint.
- a few undocumented ones.

Our software also handles regularization functions ψ on matrices W in ℝ^p × r^ (note that W can be transposed in these formulations). In particular,

- The ℓ_1_/ℓ_2_-norm: ψ(W) =^▵^ ∑_i=1_^p^ ||W_i_||_2_, where W_i_ denotes the i-th row of W.
- The ℓ_1_/ℓ_∞_-norm: ψ(W) =^▵^ ∑_i=1_^p^ ||W_i_||_∞_,
- The ℓ_1_/ℓ_2_+ℓ_1_-norm: ψ(W) =^▵^ ∑_i=1_^p^ ||W_i_||_2_ + λ_2_ ∑_i,j_|W_ij_|.
- The ℓ_1_/ℓ_∞_+ℓ_1_-norm: ψ(W) =^▵^ ∑_i=1_^p^ ||W_i_||_∞_+λ_2_ ∑_i,j_|W_ij_|,
- The ℓ_1_/ℓ_∞_-norm on rows and columns: ψ(W) =^▵^ ∑_i=1_^p^ ||W_i_||_∞_+λ_2_ ∑_j=1_^r^||W^j^||_∞_, where W^j^ denotes the j-th column of W.
- The multi-task tree-structured sum of ℓ_∞_-norms:

  |  |  |  |  |  |  |  |  |  |  |  |
  | --- | --- | --- | --- | --- | --- | --- | --- | --- | --- | --- |
  | ψ(W) | ▵; = |  | r; ∑; i=1 |  | ∑; g ∈ G | η_g_\|\|w_g_^i^\|\|_∞_+ γ | ∑; g ∈ G | η_g_ | max; j ∈ g | \|\|W_j_\|\|_∞_,     (25) |

  where the first double sums is in fact a sum of independent structured norms on the columns w^i^ of W, and the right term is a tree-structured regularization norm applied to the ℓ_∞_-norm of the rows of W, thereby inducing the tree-structured regularization at the row level. G is here a tree-structured set of groups.
- The multi-task general sum of ℓ_∞_-norms is the same as Eq. (25) except that the groups G are general overlapping groups.
- The trace norm: ψ(W) =^▵^ ||W||_\*_.

Non-convex regularizations are also implemented with the ISTA algorithm (no duality gaps are of course provided in these cases):

- The ℓ_0_-pseudo-norm: ψ(w) =^▵^ ||w||_0_.
- The rank: ψ(W) =^▵^ randk(W).
- The tree-structured ℓ_0_-pseudo-norm: ψ(w) =^▵^ ∑_g ∈ G_ δ_wg ≠ 0_.

All of these regularization terms for vectors or matrices can be coupled with
nonnegativity constraints. It is also possible to add an intercept, which one
wishes not to regularize, and we will include this possibility in the next
sections. There are also a few hidden undocumented options which are available in the source code.

We now present 3 functions for computing proximal operators associated to the previous regularization functions.

### 5.2  Function mexProximalFlat

This function computes the proximal operators associated to many regularization functions, for input signals U=[u^1^,…,u^n^] in ℝ^p × n^, it finds a matrix V=[v^1^,…,v^n^] in ℝ^p × n^ such that:

•  If one chooses a regularization function on vectors, for every column u of U, it computes one column v of V solving

min

v ∈ ℝ^p^

1

2

||u−v||_2_^2^ + λ ||v||_0_,
    (26)

or

min

v ∈ ℝ^p^

1

2

||u−v||_2_^2^ + λ ||v||_1_,
    (27)

or

|  |  |  |  |  |  |  |
| --- | --- | --- | --- | --- | --- | --- |
|  | min; v ∈ ℝ^p^ |  | 1; 2 | \|\|u−v\|\|_2_^2^ + | λ; 2 | \|\|v\|\|_2_^2^,     (28) |

or

min

v ∈ ℝ^p^

1

2

||u−v||_2_^2^ + λ ||v||_1_ + λ_2_||v||_2_^2^,
    (29)

or

|  |  |  |  |  |  |  |
| --- | --- | --- | --- | --- | --- | --- |
|  | min; v ∈ ℝ^p^ |  | 1; 2 | \|\|u−v\|\|_2_^2^ + λ | p−1; ∑; j=1 | \|v_j+1_^i^−v_j_^i^\|+λ_2_ \|\|v\|\|_1_ + λ_3_\|\|v\|\|_2_^2^,     (30) |

or

|  |  |  |  |  |  |  |
| --- | --- | --- | --- | --- | --- | --- |
|  | min; v ∈ ℝ^p^ |  | 1; 2 | \|\|u−v\|\|_2_^2^ + λ | ∑; g ∈ T | δ^g^(v),     (31) |

where T is a tree-structured set of groups (see [16]), and δ^g^(v) = 0 if v_g_=0 and 1 otherwise.
It can also solve

|  |  |  |  |  |  |  |
| --- | --- | --- | --- | --- | --- | --- |
|  | min; v ∈ ℝ^p^ |  | 1; 2 | \|\|u−v\|\|_2_^2^ + λ | ∑; g ∈ T | η^g^ \|\|v_g_\|\|_2_,     (32) |

or

|  |  |  |  |  |  |  |
| --- | --- | --- | --- | --- | --- | --- |
|  | min; v ∈ ℝ^p^ |  | 1; 2 | \|\|u−v\|\|_2_^2^ + λ | ∑; g ∈ T | η^g^ \|\|v_g_\|\|_∞_,     (33) |

or

|  |  |  |  |  |  |  |
| --- | --- | --- | --- | --- | --- | --- |
|  | min; v ∈ ℝ^p^ |  | 1; 2 | \|\|u−v\|\|_2_^2^ + λ | ∑; g ∈ G | η^g^ \|\|v_g_\|\|_∞_,     (34) |

where G is any kind of set of groups.

This function can also solve the following proximal operators on matrices

|  |  |  |  |  |  |  |
| --- | --- | --- | --- | --- | --- | --- |
|  | min; V ∈ ℝ^p × n^ |  | 1; 2 | \|\|U−V\|\|_F_^2^ + λ | p; ∑; i=1 | \|\|V_i_\|\|_2_,      (35) |

where V_i_ is the i-th row of V, or

|  |  |  |  |  |  |  |
| --- | --- | --- | --- | --- | --- | --- |
|  | min; V ∈ ℝ^p × n^ |  | 1; 2 | \|\|U−V\|\|_F_^2^ + λ | p; ∑; i=1 | \|\|V_i_\|\|_∞_,      (36) |

or

|  |  |  |  |  |  |  |  |  |  |
| --- | --- | --- | --- | --- | --- | --- | --- | --- | --- |
|  | min; V ∈ ℝ^p × n^ |  | 1; 2 | \|\|U−V\|\|_F_^2^ + λ | p; ∑; i=1 | \|\|V_i_\|\|_2_ +λ_2_ | p; ∑; i=1 | n; ∑; j=1 | \|V_ij_\|,      (37) |

or

|  |  |  |  |  |  |  |  |  |  |
| --- | --- | --- | --- | --- | --- | --- | --- | --- | --- |
|  | min; V ∈ ℝ^p × n^ |  | 1; 2 | \|\|U−V\|\|_F_^2^ + λ | p; ∑; i=1 | \|\|V_i_\|\|_∞_+λ_2_ | p; ∑; i=1 | n; ∑; j=1 | \|V_ij_\|,      (38) |

or

|  |  |  |  |  |  |  |  |  |
| --- | --- | --- | --- | --- | --- | --- | --- | --- |
|  | min; V ∈ ℝ^p × n^ |  | 1; 2 | \|\|U−V\|\|_F_^2^ + λ | p; ∑; i=1 | \|\|V_i_\|\|_∞_+λ_2_ | n; ∑; j=1 | \|\|V^j^\|\|_∞_.     (39) |

where V^j^ is the j-th column of V.

See details below:

%   
% Usage:  [V [val\_regularizer]]=mexProximalFlat(U,param);  
%  
% Name: mexProximalFlat  
%  
% Description: mexProximalFlat computes proximal operators. Depending  
%         on the value of param.regul, it computes   
%  
%         Given an input matrix U=[u^1,\ldots,u^n], it computes a matrix   
%         V=[v^1,\ldots,v^n] such that  
%         if one chooses a regularization functions on vectors, it computes  
%         for each column u of U, a column v of V solving  
%         if param.regul='l0'  
%             argmin 0.5||u-v||\_2^2 + lambda||v||\_0  
%         if param.regul='l1'  
%             argmin 0.5||u-v||\_2^2 + lambda||v||\_1  
%         if param.regul='l2'  
%             argmin 0.5||u-v||\_2^2 + 0.5lambda||v||\_2^2  
%         if param.regul='elastic-net'  
%             argmin 0.5||u-v||\_2^2 + lambda||v||\_1 + lambda\_2||v||\_2^2  
%         if param.regul='fused-lasso'  
%             argmin 0.5||u-v||\_2^2 + lambda FL(v) + ...  
%                               ...  lambda\_2||v||\_1 + lambda\_3||v||\_2^2  
%         if param.regul='linf'  
%             argmin 0.5||u-v||\_2^2 + lambda||v||\_inf  
%         if param.regul='l1-constraint'  
%             argmin 0.5||u-v||\_2^2 s.t. ||v||\_1 <= lambda  
%         if param.regul='l2-not-squared'  
%             argmin 0.5||u-v||\_2^2 + lambda||v||\_2  
%         if param.regul='group-lasso-l2'    
%             argmin 0.5||u-v||\_2^2 + lambda sum\_g ||v\_g||\_2   
%             where the groups are either defined by param.groups or by param.size\_group,  
%         if param.regul='group-lasso-linf'  
%             argmin 0.5||u-v||\_2^2 + lambda sum\_g ||v\_g||\_inf  
%         if param.regul='sparse-group-lasso-l2'    
%             argmin 0.5||u-v||\_2^2 + lambda sum\_g ||v\_g||\_2 + lambda\_2 ||v||\_1  
%             where the groups are either defined by param.groups or by param.size\_group,  
%         if param.regul='sparse-group-lasso-linf'  
%             argmin 0.5||u-v||\_2^2 + lambda sum\_g ||v\_g||\_inf + lambda\_2 ||v||\_1  
%         if param.regul='trace-norm-vec'   
%             argmin 0.5||u-v||\_2^2 + lambda ||mat(v)||\_\*   
%            where mat(v) has param.size\_group rows  
%  
%         if one chooses a regularization function on matrices  
%         if param.regul='l1l2',  V=   
%             argmin 0.5||U-V||\_F^2 + lambda||V||\_{1/2}  
%         if param.regul='l1linf',  V=   
%             argmin 0.5||U-V||\_F^2 + lambda||V||\_{1/inf}  
%         if param.regul='l1l2+l1',  V=   
%             argmin 0.5||U-V||\_F^2 + lambda||V||\_{1/2} + lambda\_2||V||\_{1/1}  
%         if param.regul='l1linf+l1',  V=   
%             argmin 0.5||U-V||\_F^2 + lambda||V||\_{1/inf} + lambda\_2||V||\_{1/1}  
%         if param.regul='l1linf+row-column',  V=   
%             argmin 0.5||U-V||\_F^2 + lambda||V||\_{1/inf} + lambda\_2||V'||\_{1/inf}  
%         if param.regul='trace-norm',  V=   
%             argmin 0.5||U-V||\_F^2 + lambda||V||\_\*  
%         if param.regul='rank',  V=   
%             argmin 0.5||U-V||\_F^2 + lambda rank(V)  
%         if param.regul='none',  V=   
%             argmin 0.5||U-V||\_F^2   
%           
%         for all these regularizations, it is possible to enforce non-negativity constraints  
%         with the option param.pos, and to prevent the last row of U to be regularized, with  
%         the option param.intercept  
%  
% Inputs: U:  double m x n matrix   (input signals)  
%               m is the signal size  
%         param: struct  
%               param.lambda  (regularization parameter)  
%               param.regul (choice of regularization, see above)  
%               param.lambda2  (optional, regularization parameter)  
%               param.lambda3  (optional, regularization parameter)  
%               param.verbose (optional, verbosity level, false by default)  
%               param.intercept (optional, last row of U is not regularized,  
%                 false by default)  
%               param.transpose (optional, transpose the matrix in the regularization function)  
%               param.size\_group (optional, for regularization functions assuming a group  
%                 structure). It is a scalar. When param.groups is not specified, it assumes  
%                 that the groups are the sets of consecutive elements of size param.size\_group  
%               param.groups (int32, optional, for regularization functions assuming a group  
%                 structure. It is an int32 vector of size m containing the group indices of the  
%                 variables (first group is 1).  
%               param.pos (optional, adds positivity constraints on the  
%                 coefficients, false by default)  
%               param.numThreads (optional, number of threads for exploiting  
%                 multi-core / multi-cpus. By default, it takes the value -1,  
%                 which automatically selects all the available CPUs/cores).  
%  
% Output: V: double m x n matrix (output coefficients)  
%         val\_regularizer: double 1 x n vector (value of the regularization  
%         term at the optimum).  
%  
% Author: Julien Mairal, 2010

The following piece of code illustrates how to use this function.

U=randn(100,1000);  
  
param.lambda=0.1; % regularization parameter  
param.num\_threads=-1; % all cores (-1 by default)  
param.verbose=true;   % verbosity, false by default  
  
% test l0  
fprintf('\nprox l0\n');  
param.regul='l0';  
param.pos=false;       % false by default  
param.intercept=false; % false by default  
alpha=mexProximalFlat(U,param);  
  
% test l1  
fprintf('\nprox l1, intercept, positivity constraint\n');  
param.regul='l1';  
param.pos=true;       % can be used with all the other regularizations  
param.intercept=true; % can be used with all the other regularizations       
alpha=mexProximalFlat(U,param);  
  
% test l2  
fprintf('\nprox squared-l2\n');  
param.regul='l2';  
param.pos=false;  
param.intercept=false;  
alpha=mexProximalFlat(U,param);  
  
% test elastic-net  
fprintf('\nprox elastic-net\n');  
param.regul='elastic-net';  
param.lambda2=0.1;  
alpha=mexProximalFlat(U,param);  
  
% test fused-lasso  
fprintf('\nprox fused lasso\n');  
param.regul='fused-lasso';  
param.lambda2=0.1;  
param.lambda3=0.1;  
alpha=mexProximalFlat(U,param);  
  
% test l1l2  
fprintf('\nprox mixed norm l1/l2\n');  
param.regul='l1l2';  
alpha=mexProximalFlat(U,param);  
  
% test l1linf  
fprintf('\nprox mixed norm l1/linf\n');  
param.regul='l1linf';  
alpha=mexProximalFlat(U,param);  
  
% test l1l2+l1  
fprintf('\nprox mixed norm l1/l2 + l1\n');  
param.regul='l1l2+l1';  
param.lambda2=0.1;  
alpha=mexProximalFlat(U,param);  
  
% test l1linf+l1  
fprintf('\nprox mixed norm l1/linf + l1\n');  
param.regul='l1linf+l1';  
param.lambda2=0.1;  
alpha=mexProximalFlat(U,param);  
  
% test l1linf-row-column  
fprintf('\nprox mixed norm l1/linf on rows and columns\n');  
param.regul='l1linf-row-column';  
param.lambda2=0.1;  
alpha=mexProximalFlat(U,param);  
  
% test none  
fprintf('\nprox no regularization\n');  
param.regul='none';  
alpha=mexProximalFlat(U,param);

### 5.3  Function mexProximalTree

This function computes the proximal operators associated to tree-structured regularization functions, for input signals U=[u^1^,…,u^n^] in ℝ^p × n^, and a tree-structured set of groups [15], it computes a matrix V=[v^1^,…,v^n^] in ℝ^p × n^. When one uses a regularization function on vectors, it computes a column v of V for every column u of U:

|  |  |  |  |  |  |  |
| --- | --- | --- | --- | --- | --- | --- |
|  | min; v ∈ ℝ^p^ |  | 1; 2 | \|\|u−v\|\|_2_^2^ + λ | ∑; g ∈ T | η^g^ \|\|v_g_\|\|_2_,     (40) |

or

|  |  |  |  |  |  |  |
| --- | --- | --- | --- | --- | --- | --- |
|  | min; v ∈ ℝ^p^ |  | 1; 2 | \|\|u−v\|\|_2_^2^ + λ | ∑; g ∈ T | η^g^ \|\|v_g_\|\|_∞_,     (41) |

or

|  |  |  |  |  |  |  |
| --- | --- | --- | --- | --- | --- | --- |
|  | min; v ∈ ℝ^p^ |  | 1; 2 | \|\|u−v\|\|_2_^2^ + λ | ∑; g ∈ T | δ^g^(v),     (42) |

where δ^g^(v)=0 if v_g_=0 and 1 otherwise (see appendix of [16]).

When the multi-task tree-structured regularization function is used, it solves

|  |  |  |  |  |  |  |  |  |  |  |  |
| --- | --- | --- | --- | --- | --- | --- | --- | --- | --- | --- | --- |
|  | min; V ∈ ℝ^p× n^ |  | 1; 2 | \|\|U−V\|\|_F_^2^ + λ | n; ∑; i=1 | ∑; g ∈ T | η^g^ \|\|v_g_^i^\|\|_∞_+ λ_2_ | ∑; g ∈ T |  | max; j ∈ g | \|\|v_g_^j^\|\|_∞_,     (43) |

which is a formulation presented in [22].

This function can also be used for computing the proximal operators addressed by mexProximalFlat (it will just not take into account the tree structure). The way the tree is incoded is presented below, (and examples are given in the file test\_ProximalTree.m, with more usage details:

%   
% Usage:  [V [val\_regularizer]]=mexProximalTree(U,tree,param);  
%  
% Name: mexProximalTree  
%  
% Description: mexProximalTree computes a proximal operator. Depending  
%         on the value of param.regul, it computes   
%  
%         Given an input matrix U=[u^1,\ldots,u^n], and a tree-structured set of groups T,  
%         it returns a matrix V=[v^1,\ldots,v^n]:  
%           
%         when the regularization function is for vectors,  
%         for every column u of U, it compute a column v of V solving  
%         if param.regul='tree-l0'  
%             argmin 0.5||u-v||\_2^2 + lambda \sum\_{g \in T} \delta^g(v)  
%         if param.regul='tree-l2'  
%           for all i, v^i =   
%             argmin 0.5||u-v||\_2^2 + lambda\sum\_{g \in T} \eta\_g||v\_g||\_2  
%         if param.regul='tree-linf'  
%           for all i, v^i =   
%             argmin 0.5||u-v||\_2^2 + lambda\sum\_{g \in T} \eta\_g||v\_g||\_inf  
%  
%         when the regularization function is for matrices:  
%         if param.regul='multi-task-tree'  
%            V=argmin 0.5||U-V||\_F^2 + lambda \sum\_{i=1}^n\sum\_{g \in T} \eta\_g||v^i\_g||\_inf + ...  
%                                                lambda\_2 \sum\_{g \in T} \eta\_g max\_{j in g}||V\_j||\_{inf}  
%           
%         it can also be used with any non-tree-structured regularization addressed by mexProximalFlat  
%  
%         for all these regularizations, it is possible to enforce non-negativity constraints  
%         with the option param.pos, and to prevent the last row of U to be regularized, with  
%         the option param.intercept  
%  
% Inputs: U:  double m x n matrix   (input signals)  
%               m is the signal size  
%         tree: struct  
%               with four fields, eta\_g, groups, own\_variables and N\_own\_variables.  
%  
%               The tree structure requires a particular organization of groups and variables  
%                  \* Let us denote by N = |T|, the number of groups.  
%                    the groups should be ordered T={g1,g2,\ldots,gN} such that if gi is included  
%                    in gj, then j <= i. g1 should be the group at the root of the tree   
%                    and contains every variable.  
%                  \* Every group is a set of  contiguous indices for instance   
%                    gi={3,4,5} or gi={4,5,6,7} or gi={4}, but not {3,5};  
%                  \* We define root(gi) as the indices of the variables that are in gi,  
%                    but not in its descendants. For instance for  
%                    T={ g1={1,2,3,4},g2={2,3},g3={4} }, then, root(g1)={1},   
%                    root(g2)={2,3}, root(g3)={4},  
%                    We assume that for all i, root(gi) is a set of contigous variables  
%                  \* We assume that the smallest of root(gi) is also the smallest index of gi.  
%  
%                  For instance,   
%                    T={ g1={1,2,3,4},g2={2,3},g3={4} }, is a valid set of groups.  
%                    but we can not have  
%                    T={ g1={1,2,3,4},g2={1,2},g3={3} }, since root(g1)={4} and 4 is not the  
%                    smallest element in g1.  
%  
%               We do not lose generality with these assumptions since they can be fullfilled for any  
%               tree-structured set of groups after a permutation of variables and a correct ordering of the  
%               groups.  
%               see more examples in test\_ProximalTree.m of valid tree-structured sets of groups.  
%                 
%               The first fields sets the weights for every group  
%                  tree.eta\_g            double N vector   
%    
%               The next field sets inclusion relations between groups   
%               (but not between groups and variables):  
%                  tree.groups           sparse (double or boolean) N x N matrix    
%                  the (i,j) entry is non-zero if and only if i is different than j and   
%                  gi is included in gj.  
%                  the first column corresponds to the group at the root of the tree.  
%  
%               The next field define the smallest index of each group gi,   
%               which is also the smallest index of root(gi)  
%               tree.own\_variables    int32 N vector  
%  
%               The next field define for each group gi, the size of root(gi)  
%               tree.N\_own\_variables  int32 N vector   
%  
%               examples are given in test\_ProximalTree.m  
%  
%         param: struct  
%               param.lambda  (regularization parameter)  
%               param.regul (choice of regularization, see above)  
%               param.lambda2  (optional, regularization parameter)  
%               param.lambda3  (optional, regularization parameter)  
%               param.verbose (optional, verbosity level, false by default)  
%               param.intercept (optional, last row of U is not regularized,  
%                 false by default)  
%               param.pos (optional, adds positivity constraints on the  
%                 coefficients, false by default)  
%               param.transpose (optional, transpose the matrix in the regularization function)  
%               param.size\_group (optional, for regularization functions assuming a group  
%                 structure). It is a scalar. When param.groups is not specified, it assumes  
%                 that the groups are the sets of consecutive elements of size param.size\_group  
%               param.numThreads (optional, number of threads for exploiting  
%                 multi-core / multi-cpus. By default, it takes the value -1,  
%                 which automatically selects all the available CPUs/cores).  
%  
% Output: V: double m x n matrix (output coefficients)  
%         val\_regularizer: double 1 x n vector (value of the regularization  
%         term at the optimum).  
%  
%  
% Author: Julien Mairal, 2010

The following piece of code illustrates how to use this function.

U=randn(10,1000);  
  
param.lambda=0.1; % regularization parameter  
param.num\_threads=-1; % all cores (-1 by default)  
param.verbose=true;   % verbosity, false by default  
param.pos=false;       % can be used with all the other regularizations  
param.intercept=false; % can be used with all the other regularizations       
  
fprintf('First tree example\n');  
% Example 1 of tree structure  
% tree structured groups:  
% g1= {0 1 2 3 4 5 6 7 8 9}  
% g2= {2 3 4}  
% g3= {5 6 7 8 9}  
tree.own\_variables=int32([0 2 5]);   % pointer to the first variable of each group  
tree.N\_own\_variables=int32([2 3 5]); % number of "root" variables in each group  
                              % (variables that are in a group, but not in its descendants).  
                              % for instance root(g1)={0,1}, root(g2)={2 3 4}, root(g3)={5 6 7 8 9}  
tree.eta\_g=[1 1 1];           % weights for each group, they should be non-zero to use fenchel duality  
tree.groups=sparse([0 0 0; ...  
                    1 0 0; ...  
                    1 0 0]);    % first group should always be the root of the tree  
                                % non-zero entriees mean inclusion relation ship, here g2 is a children of g1,  
                                % g3 is a children of g1  
  
fprintf('\ntest prox tree-l0\n');  
param.regul='tree-l0';  
alpha=mexProximalTree(U,tree,param);  
  
fprintf('\ntest prox tree-l2\n');  
param.regul='tree-l2';  
alpha=mexProximalTree(U,tree,param);  
  
fprintf('\ntest prox tree-linf\n');  
param.regul='tree-linf';  
alpha=mexProximalTree(U,tree,param);  
  
fprintf('Second tree example\n');  
% Example 2 of tree structure  
% tree structured groups:  
% g1= {0 1 2 3 4 5 6 7 8 9}    root(g1) = { };  
% g2= {0 1 2 3 4 5}            root(g2) = {0 1 2};  
% g3= {3 4}                    root(g3) = {3 4};  
% g4= {5}                      root(g4) = {5};  
% g5= {6 7 8 9}                root(g5) = { };  
% g6= {6 7}                    root(g6) = {6 7};  
% g7= {8 9}                    root(g7) = {8};  
% g8 = {9}                     root(g8) = {9};  
tree.own\_variables=  int32([0 0 3 5 6 6 8 9]);   % pointer to the first variable of each group  
tree.N\_own\_variables=int32([0 3 2 1 0 2 1 1]); % number of "root" variables in each group  
tree.eta\_g=[1 1 1 2 2 2 2.5 2.5];  
tree.groups=sparse([0 0 0 0 0 0 0 0; ...  
                    1 0 0 0 0 0 0 0; ...  
                    0 1 0 0 0 0 0 0; ...  
                    0 1 0 0 0 0 0 0; ...  
                    1 0 0 0 0 0 0 0; ...  
                    0 0 0 0 1 0 0 0; ...  
                    0 0 0 0 1 0 0 0; ...  
                    0 0 0 0 0 0 1 0]);  % first group should always be the root of the tree  
  
fprintf('\ntest prox tree-l0\n');  
param.regul='tree-l0';  
alpha=mexProximalTree(U,tree,param);  
  
fprintf('\ntest prox tree-l2\n');  
param.regul='tree-l2';  
alpha=mexProximalTree(U,tree,param);  
  
fprintf('\ntest prox tree-linf\n');  
param.regul='tree-linf';  
alpha=mexProximalTree(U,tree,param);  
  
% mexProximalTree also works with non-tree-structured regularization functions  
fprintf('\nprox l1, intercept, positivity constraint\n');  
param.regul='l1';  
param.pos=true;       % can be used with all the other regularizations  
param.intercept=true; % can be used with all the other regularizations       
alpha=mexProximalTree([U; ones(1,size(U,2))],tree,param);  
  
% Example of multi-task tree  
fprintf('\nprox multi-task tree\n');  
param.pos=false;  
param.intercept=false;  
param.lambda2=param.lambda;  
param.regul='multi-task-tree';  % with linf  
alpha=mexProximalTree(U,tree,param);  
  
  
tree.own\_variables=int32([0 1 2 3 4 5 6]);   % pointer to the first variable of each group  
tree.N\_own\_variables=int32([1 1 1 1 1 1]); % number of "root" variables in each group  
                              % (variables that are in a group, but not in its descendants).  
                              % for instance root(g1)={0,1}, root(g2)={2 3 4}, root(g3)={5 6 7 8 9}  
tree.eta\_g=[1 1 1 1 1 1];           % weights for each group, they should be non-zero to use fenchel duality  
tree.groups=sparse([0 0 0; ...  
                    1 0 0; ...  
                    1 0 0]);    % first group should always be the root of the tree  
                                % non-zero entriees mean inclusion relation ship, here g2 is a children of g1,  
                                % g3 is a children of g1

### 5.4  Function mexProximalGraph

This function computes the proximal operators associated to structured sparse regularization, for input signals U=[u^1^,…,u^n^] in ℝ^p × n^, and a set of groups [22], it returns a matrix V=[v^1^,…,v^n^] in ℝ^p × n^.
When one uses a regularization function on vectors, it computes a column v of V for every column u of U:

|  |  |  |  |  |  |  |
| --- | --- | --- | --- | --- | --- | --- |
|  | min; v ∈ ℝ^p^ |  | 1; 2 | \|\|u−v\|\|_2_^2^ + λ | ∑; g ∈ G | η^g^ \|\|v_g_\|\|_∞_,     (44) |

or with a regularization function on matrices, it computes V solving

|  |  |  |  |  |  |  |  |  |  |  |  |
| --- | --- | --- | --- | --- | --- | --- | --- | --- | --- | --- | --- |
|  | min; V ∈ ℝ^p× n^ |  | 1; 2 | \|\|U−V\|\|_F_^2^ + λ | n; ∑; i=1 | ∑; g ∈ G | η^g^ \|\|v_g_^i^\|\|_∞_+ λ_2_ | ∑; g ∈ G |  | max; j ∈ g | \|\|v_g_^j^\|\|_∞_,     (45) |

This function can also be used for computing the proximal operators addressed by mexProximalFlat. The way the graph is incoded is presented below (and also in the example file test\_ProximalGraph.m, with more usage details:

%   
% Usage:   [V [val\_regularizer]]=mexProximalGraph(U,graph,param);  
%  
% Name: mexProximalGraph  
%  
% Description: mexProximalGraph computes a proximal operator. Depending  
%         on the value of param.regul, it computes   
%  
%         Given an input matrix U=[u^1,\ldots,u^n], and a set of groups G,  
%         it computes a matrix V=[v^1,\ldots,v^n] such that  
%  
%         if param.regul='graph'  
%         for every column u of U, it computes a column v of V solving  
%             argmin 0.5||u-v||\_2^2 + lambda\sum\_{g \in G} \eta\_g||v\_g||\_inf  
%  
%         if param.regul='graph+ridge'  
%         for every column u of U, it computes a column v of V solving  
%             argmin 0.5||u-v||\_2^2 + lambda\sum\_{g \in G} \eta\_g||v\_g||\_inf + lambda\_2||v||\_2^2  
%  
%  
%         if param.regul='multi-task-graph'  
%            V=argmin 0.5||U-V||\_F^2 + lambda \sum\_{i=1}^n\sum\_{g \in G} \eta\_g||v^i\_g||\_inf + ...  
%                                                lambda\_2 \sum\_{g \in G} \eta\_g max\_{j in g}||V\_j||\_{inf}  
%           
%         it can also be used with any regularization addressed by mexProximalFlat  
%  
%         for all these regularizations, it is possible to enforce non-negativity constraints  
%         with the option param.pos, and to prevent the last row of U to be regularized, with  
%         the option param.intercept  
%  
% Inputs: U:  double p x n matrix   (input signals)  
%               m is the signal size  
%         graph: struct  
%               with three fields, eta\_g, groups, and groups\_var  
%  
%               The first fields sets the weights for every group  
%                  graph.eta\_g            double N vector   
%    
%               The next field sets inclusion relations between groups   
%               (but not between groups and variables):  
%                  graph.groups           sparse (double or boolean) N x N matrix    
%                  the (i,j) entry is non-zero if and only if i is different than j and   
%                  gi is included in gj.  
%                 
%               The next field sets inclusion relations between groups and variables  
%                  graph.groups\_var       sparse (double or boolean) p x N matrix  
%                  the (i,j) entry is non-zero if and only if the variable i is included   
%                  in gj, but not in any children of gj.  
%  
%               examples are given in test\_ProximalGraph.m  
%  
%         param: struct  
%               param.lambda  (regularization parameter)  
%               param.regul (choice of regularization, see above)  
%               param.lambda2  (optional, regularization parameter)  
%               param.lambda3  (optional, regularization parameter)  
%               param.verbose (optional, verbosity level, false by default)  
%               param.intercept (optional, last row of U is not regularized,  
%                 false by default)  
%               param.pos (optional, adds positivity constraints on the  
%                 coefficients, false by default)  
%               param.numThreads (optional, number of threads for exploiting  
%                 multi-core / multi-cpus. By default, it takes the value -1,  
%                 which automatically selects all the available CPUs/cores).  
%  
% Output: V: double p x n matrix (output coefficients)  
%         val\_regularizer: double 1 x n vector (value of the regularization  
%         term at the optimum).  
%  
% Author: Julien Mairal, 2010

The following piece of code illustrates how to use this function.

U=randn(10,1000);  
  
param.lambda=0.1; % regularization parameter  
param.num\_threads=-1; % all cores (-1 by default)  
param.verbose=true;   % verbosity, false by default  
param.pos=false;       % can be used with all the other regularizations  
param.intercept=false; % can be used with all the other regularizations       
  
fprintf('First graph example\n');  
% Example 1 of graph structure  
% groups:  
% g1= {0 1 2 3}  
% g2= {3 4 5 6}  
% g3= {6 7 8 9}  
graph.eta\_g=[1 1 1];  
graph.groups=sparse(zeros(3));  
graph.groups\_var=sparse([1 0 0;  
                         1 0 0;  
                         1 0 0;  
                         1 1 0;  
                         0 1 0;  
                         0 1 0;  
                         0 1 1;  
                         0 0 1;  
                         0 0 1;  
                         0 0 1]);  
  
fprintf('\ntest prox graph\n');  
param.regul='graph';  
alpha=mexProximalGraph(U,graph,param);  
  
% Example 2 of graph structure  
% groups:  
% g1= {0 1 2 3}  
% g2= {3 4 5 6}  
% g3= {6 7 8 9}  
% g4= {0 1 2 3 4 5}  
% g5= {6 7 8}  
graph.eta\_g=[1 1 1 1 1];  
graph.groups=sparse([0 0 0 1 0;  
                     0 0 0 0 0;  
                     0 0 0 0 0;  
                     0 0 0 0 0;  
                     0 0 1 0 0]);   % g5 is included in g3, and g2 is included in g4  
graph.groups\_var=sparse([1 0 0 0 0;  
                         1 0 0 0 0;  
                         1 0 0 0 0 ;  
                         1 1 0 0 0;  
                         0 1 0 1 0;  
                         0 1 0 1 0;  
                         0 1 0 0 1;  
                         0 0 0 0 1;  
                         0 0 0 0 1;  
                         0 0 1 0 0]); % represents direct inclusion relations   
                                      % between groups (columns) and variables (rows)  
  
fprintf('\ntest prox graph\n');  
param.regul='graph';  
alpha=mexProximalGraph(U,graph,param);  
  
fprintf('\ntest prox multi-task-graph\n');  
param.regul='multi-task-graph';  
param.lambda2=0.1;  
alpha=mexProximalGraph(U,graph,param);  
  
fprintf('\ntest no regularization\n');  
param.regul='none';  
alpha=mexProximalGraph(U,graph,param);

### 5.5  Function mexProximalPathCoding

This function computes the proximal operators associated to the path coding penalties of [24].

%   
% Usage:   [V [val\_regularizer]]=mexProximalPathCoding(U,DAG,param);  
%  
% Name: mexProximalPathCoding  
%  
% Description: mexProximalPathCoding computes a proximal operator for  
%         the path coding penalties of http://arxiv.org/abs/1204.4539  
%  
%         Given an input matrix U=[u^1,\ldots,u^n],   
%  
%  
% Inputs: U:  double p x n matrix   (input signals)  
%               m is the signal size  
%         DAG:  struct  
%               with three fields, weights, start\_weights, stop\_weights  
%         for a graph with |V| nodes and |E| arcs,  
%         DAG.weights: sparse double |V| x |V| matrix. Adjacency  
%               matrix. The non-zero entries represent costs on arcs  
%               linking two nodes.  
%         DAG.start\_weights: dense double |V| vector. Represent the costs  
%               of starting a path from a specific node.  
%         DAG.stop\_weights: dense double |V| vector. Represent the costs  
%               of ending a path at a specific node.  
%  
%         if param.regul='graph-path-l0', non-convex penalty  
%         if param.regul='graph-path-conv', convex penalty  
%  
%         param: struct  
%               param.lambda  (regularization parameter)  
%               param.regul (choice of regularization, see above)  
%               param.verbose (optional, verbosity level, false by default)  
%               param.intercept (optional, last row of U is not regularized,  
%                 false by default)  
%               param.pos (optional, adds positivity constraints on the  
%                 coefficients, false by default)  
%               param.precision (optional, by default a very large integer.  
%                 It returns approximate proximal operator by choosing a small integer,  
%                 for example, 100 or 1000.  
%               param.numThreads (optional, number of threads for exploiting  
%                 multi-core / multi-cpus. By default, it takes the value -1,  
%                 which automatically selects all the available CPUs/cores).  
%  
% Output: V: double p x n matrix (output coefficients)  
%         val\_regularizer: double 1 x n vector (value of the regularization  
%         term at the optimum).  
%  
% Author: Julien Mairal, 2012

The following piece of code illustrates how to use this function.

clear all;  
rand('seed',0);  
randn('seed',0);  
fprintf('test mexProximalPathCoding\n');  
p=100;  
% generate a DAG  
G=sprand(p,p,0.02);  
G=mexRemoveCyclesGraph(G);  
fprintf('\n');  
  
% generate a data matrix  
U=randn(p,10);  
U=U-mean(U(:));  
U=mexNormalize(U);  
  
% input graph  
graph.weights=G;  
graph.stop\_weights=zeros(1,p);  
graph.start\_weights=10\*ones(1,p);  
  
% FISTA parameters  
param.num\_threads=-1; % all cores (-1 by default)  
param.verbose=true;   % verbosity, false by default  
param.lambda=0.05; % regularization parameter  
  
fprintf('Proximal convex path penalty\n');  
param.regul='graph-path-conv';  
tic  
[V1 optim]=mexProximalPathCoding(U,graph,param);  
t=toc;  
num=mexCountConnexComponents(graph.weights,V1(:,1));  
fprintf('Num of connected components: %d\n',num);  
  
fprintf('Proximal non-convex path penalty\n');  
param.regul='graph-path-l0';  
param.lambda=0.005;  
tic  
[V2 optim]=mexProximalPathCoding(U,graph,param);  
t=toc;  
num=mexCountConnexComponents(graph.weights,V2(:,1));  
fprintf('Num of connected components: %d\n',num);  
  
graph.start\_weights=1\*ones(1,p);  
param.lambda=0.05;  
fprintf('Proximal convex path penalty\n');  
param.regul='graph-path-conv';  
tic  
[V1 optim]=mexProximalPathCoding(U,graph,param);  
t=toc;  
num=mexCountConnexComponents(graph.weights,V1(:,1));  
fprintf('Num of connected components: %d\n',num);  
  
fprintf('Proximal non-convex path penalty\n');  
param.regul='graph-path-l0';  
param.lambda=0.005;  
tic  
[V2 optim]=mexProximalPathCoding(U,graph,param);  
t=toc;  
num=mexCountConnexComponents(graph.weights,V2(:,1));  
fprintf('Num of connected components: %d\n',num);

This function is associated to a function to evaluate the penalties:

### 5.6  Function mexEvalPathCoding

%   
% Usage:   [val [paths]]=mexEvalPathCoding(U,DAG,param);  
%  
% Name: mexEvalPathCoding  
%  
% Description: mexEvalPathCoding evaluate the path coding penalies   
%         of http://arxiv.org/abs/1204.4539 and provides a path   
%         decomposition of a vector W.  
%  
%         Given an input matrix U=[u^1,\ldots,u^n],   
%  
%  
% Inputs: U:  double p x n matrix   (input signals)  
%               m is the signal size  
%         DAG:  struct  
%               with three fields, weights, start\_weights, stop\_weights  
%         for a graph with |V| nodes and |E| arcs,  
%         DAG.weights: sparse double |V| x |V| matrix. Adjacency  
%               matrix. The non-zero entries represent costs on arcs  
%               linking two nodes.  
%         DAG.start\_weights: dense double |V| vector. Represent the costs  
%               of starting a path from a specific node.  
%         DAG.stop\_weights: dense double |V| vector. Represent the costs  
%               of ending a path at a specific node.  
%  
%         if param.regul='graph-path-l0', non-convex penalty  
%         if param.regul='graph-path-conv', convex penalty  
%  
%         param: struct  
%               param.regul (choice of regularization, see above)  
%               param.verbose (optional, verbosity level, false by default)  
%               param.precision (optional, by default a very large integer.  
%                 It returns approximate proximal operator by choosing a small integer,  
%                 for example, 100 or 1000.  
%               param.numThreads (optional, number of threads for exploiting  
%                 multi-core / multi-cpus. By default, it takes the value -1,  
%                 which automatically selects all the available CPUs/cores).  
%  
% Output: V: double 1 x n vector (values of the objective function)  
%         paths: optional, double sparse p x k matrix. selected paths for the   
%                first column of U  
%  
% Author: Julien Mairal, 2012

The following piece of code illustrates how to use this function.

clear all;  
rand('seed',0);  
randn('seed',0);  
  
p=100;  
G=sprand(p,p,0.05);  
G=mexRemoveCyclesGraph(G);  
fprintf('\n');  
  
% input graph  
graph.weights=G;  
graph.stop\_weights=zeros(1,p);  
graph.start\_weights=10\*ones(1,p);  
  
param.regul='graph-path-l0';  
U=randn(p,10);  
U=U-mean(U(:));  
U=mexNormalize(U);  
param.lambda=0.005;  
[V2 optim]=mexProximalPathCoding(U,graph,param);  
[vals paths]=mexEvalPathCoding(U,graph,param);

After having presented the regularization terms which our software can handle,
we present the various formulations that we address

### 5.7  Problems Addressed

We present here regression or classification formulations and their multi-task variants.

#### 5.7.1  Regression Problems with the Square Loss

Given a training set {x^i^,y_i_}_i=1_^n^, with x^i^ ∈ ℝ^p^ and y_i_ ∈ ℝ for all i in [ 1;n ], we address

min

w ∈ ℝ^p^, b ∈ ℝ

n

∑

i=1

1

2

(y_i_−w^⊤^x^i^−b)^2^ + λψ(w),

where b is an optional variable acting as an “intercept”, which is not regularized, and ψ
can be any of the regularization functions presented above.
Let us consider the vector y in ℝ^n^ that carries the entries y_i_.
The problem without the intercept takes the following form, which we have already
encountered in the previous toolbox, but with different notations:

min

w ∈ ℝ^p^

1

2

||y−Xw||_2_^2^ + λψ(w),

where the X=[x^i^,…,x^n^]^T^ (the x^i^’s are here the rows of X).

#### 5.7.2  Classification Problems with the Logistic Loss

The next formulation that our software can solve is the regularized logistic regression formulation.
We are again given a training set {x^i^,y_i_}_i=1_^n^, with x^i^ ∈
ℝ^p^, but the variables y_i_ are now in {−1,+1} for all i in
[ 1;n ]. The optimization problem we address is

min

w ∈ ℝ^p^, b ∈ ℝ

1

n

n

∑

i=1

log(1+e^−y_i_(w⊤xi+b)^ + λψ(w),

with again ψ taken to be one of the regularization function presented above, and b is an optional intercept.

#### 5.7.3  Multi-class Classification Problems with the Softmax Loss

We have also implemented a multi-class logistic classifier (or softmax).
For a classification problem with r classes, we are given a training set {x^i^,y_i_}_i=1_^n^, where the variables x^i^ are still vectors in ℝ^p^, but the y_i_’s have integer values in {1,2,…,r}. The formulation we address is the following multi-class learning problem

|  |  |  |  |  |  |  |  |  |  |  |  |  |
| --- | --- | --- | --- | --- | --- | --- | --- | --- | --- | --- | --- | --- |
|  | min; W ∈ ℝ^p × r^, b ∈ ℝ^r^ |  | 1; n | n; ∑; i=1 | log | ⎛  ⎜  ⎝ | r; ∑; j=1 | e ^(wj−wy_i_)⊤xi + b_j_−b_yi_^ | ⎞  ⎟  ⎠ | + λ | r; ∑; j=1 | ψ(w^j^),     (46) |

where W = [w^1^,…,w^r^] and the optional vector b in ℝ^r^ carries intercepts for each class.

#### 5.7.4  Multi-task Regression Problems with the Square Loss

We are now considering a problem with r tasks, and a training set
{x^i^,y^i^}_i=1_^n^, where the variables x^i^ are still vectors in ℝ^p^, and y^i^
is a vector in ℝ^r^. We are looking for r regression vectors w^j^, for j∈ [ 1;r ], or equivalently for a matrix W=[w^1^,…,w^r^] in ℝ^p × r^. The formulation we address is the following
multi-task regression problem

min

W ∈ ℝ^p × r^, b ∈ ℝ^r^

r

∑

j=1

n

∑

i=1

1

2

(y_j_^i^−w^⊤^x^i^−b_j_)^2^ + λψ(W),

where ψ is any of the regularization function on matrices we have presented in the previous section.
Note that by introducing the appropriate variables Y, the problem without intercept could be equivalently rewritten

min

W ∈ ℝ^p × r^

1

2

||Y−XW||_F_^2^ + λψ(W).

#### 5.7.5  Multi-task Classification Problems with the Logistic Loss

The multi-task version of the logistic regression follows the same principle.
We consider r tasks, and a training set
{x^i^,y^i^}_i=1_^n^, with the x^i^’s in ℝ^p^, and the y^i^’s
are vectors in {−1,+1}^r^. We look for a matrix W=[w^1^,…,w^r^] in ℝ^p × r^. The formulation is the following
multi-task regression problem

|  |  |  |  |  |  |  |  |  |  |  |
| --- | --- | --- | --- | --- | --- | --- | --- | --- | --- | --- |
|  | min; W ∈ ℝ^p × r^, b ∈ ℝ^r^ |  | r; ∑; j=1 | 1; n | n; ∑; i=1 | log | ⎛  ⎜  ⎝ | 1+e^−y_j_i(w⊤xi+b_j_)^ | ⎞  ⎟  ⎠ | + λψ(W). |

#### 5.7.6  Multi-task and Multi-class Classification Problems with the Softmax Loss

The multi-task/multi-class version directly follows from the formulation of Eq. (46), but associates with each class a task, and as a consequence, regularizes the matrix W in a particular way:

|  |  |  |  |  |  |  |  |  |  |  |
| --- | --- | --- | --- | --- | --- | --- | --- | --- | --- | --- |
|  | min; W ∈ ℝ^p × r^, b ∈ ℝ^r^ |  | 1; n | n; ∑; i=1 | log | ⎛  ⎜  ⎝ | r; ∑; j=1 | e ^(wj−wy_i_)⊤xi + b_j_−b_yi_^ | ⎞  ⎟  ⎠ | + λψ(W). |

How duality gaps are computed for any of these formulations is presented in Appendix A.
We now present the main functions for solving these problems

### 5.8  Function mexFistaFlat

Given a matrix X=[x^1^,…,x^p^]^T^ in ℝ^m × p^, and a matrix Y=[y^1^,…,y^n^], it solves the optimization problems presented in the previous section, with the same regularization functions as mexProximalFlat.
see usage details below:

%   
% Usage: [W [optim]]=mexFistaFlat(Y,X,W0,param);  
%  
% Name: mexFistaFlat  
%  
% Description: mexFistaFlat solves sparse regularized problems.  
%         X is a design matrix of size m x p  
%         X=[x^1,...,x^n]', where the x\_i's are the rows of X  
%         Y=[y^1,...,y^n] is a matrix of size m x n  
%         It implements the algorithms FISTA, ISTA and subgradient descent.  
%           
%           - if param.loss='square' and param.regul is a regularization function for vectors,  
%             the entries of Y are real-valued,  W = [w^1,...,w^n] is a matrix of size p x n  
%             For all column y of Y, it computes a column w of W such that  
%               w = argmin 0.5||y- X w||\_2^2 + lambda psi(w)  
%  
%           - if param.loss='square' and param.regul is a regularization function for matrices  
%             the entries of Y are real-valued,  W is a matrix of size p x n.   
%             It computes the matrix W such that  
%               W = argmin 0.5||Y- X W||\_F^2 + lambda psi(W)  
%              
%           - param.loss='square-missing' : same as param.loss='square', but handles missing data  
%             represented by NaN (not a number) in the matrix Y  
%  
%           - if param.loss='logistic' and param.regul is a regularization function for vectors,  
%             the entries of Y are either -1 or +1, W = [w^1,...,w^n] is a matrix of size p x n  
%             For all column y of Y, it computes a column w of W such that  
%               w = argmin (1/m)sum\_{j=1}^m log(1+e^(-y\_j x^j' w)) + lambda psi(w),  
%             where x^j is the j-th row of X.  
%  
%           - if param.loss='logistic' and param.regul is a regularization function for matrices  
%             the entries of Y are either -1 or +1, W is a matrix of size p x n  
%               W = argmin sum\_{i=1}^n(1/m)sum\_{j=1}^m log(1+e^(-y^i\_j x^j' w^i)) + lambda psi(W)  
%  
%           - if param.loss='multi-logistic' and param.regul is a regularization function for vectors,  
%             the entries of Y are in {0,1,...,N} where N is the total number of classes  
%             W = [W^1,...,W^n] is a matrix of size p x Nn, each submatrix W^i is of size p x N  
%             for all submatrix WW of W, and column y of Y, it computes  
%               WW = argmin (1/m)sum\_{j=1}^m log(sum\_{j=1}^r e^(x^j'(ww^j-ww^{y\_j}))) + lambda sum\_{j=1}^N psi(ww^j),  
%             where ww^j is the j-th column of WW.  
%  
%           - if param.loss='multi-logistic' and param.regul is a regularization function for matrices,  
%             the entries of Y are in {0,1,...,N} where N is the total number of classes  
%             W is a matrix of size p x N, it computes  
%               W = argmin (1/m)sum\_{j=1}^m log(sum\_{j=1}^r e^(x^j'(w^j-w^{y\_j}))) + lambda psi(W)  
%             where ww^j is the j-th column of WW.  
%  
%           - param.loss='cur' : useful to perform sparse CUR matrix decompositions,   
%               W = argmin 0.5||Y-X\*W\*X||\_F^2 + lambda psi(W)  
%  
%  
%         The function psi are those used by mexProximalFlat (see documentation)  
%  
%         This function can also handle intercepts (last row of W is not regularized),  
%         and/or non-negativity constraints on W, and sparse matrices for X  
%  
% Inputs: Y:  double dense m x n matrix  
%         X:  double dense or sparse m x p matrix     
%         W0:  double dense p x n matrix or p x Nn matrix (for multi-logistic loss)  
%              initial guess  
%         param: struct  
%            param.loss (choice of loss, see above)  
%            param.regul (choice of regularization, see function mexProximalFlat)  
%            param.lambda (regularization parameter)  
%            param.lambda2 (optional, regularization parameter, 0 by default)  
%            param.lambda3 (optional, regularization parameter, 0 by default)  
%            param.verbose (optional, verbosity level, false by default)  
%            param.pos (optional, adds positivity constraints on the  
%                coefficients, false by default)  
%            param.transpose (optional, transpose the matrix in the regularization function)  
%            param.size\_group (optional, for regularization functions assuming a group  
%                 structure)  
%            param.groups (int32, optional, for regularization functions assuming a group  
%                 structure, see mexProximalFlat)  
%            param.numThreads (optional, number of threads for exploiting  
%                multi-core / multi-cpus. By default, it takes the value -1,  
%                which automatically selects all the available CPUs/cores).  
%            param.max\_it (optional, maximum number of iterations, 100 by default)  
%            param.it0 (optional, frequency for computing duality gap, every 10 iterations by default)  
%            param.tol (optional, tolerance for stopping criteration, which is a relative duality gap  
%                if it is available, or a relative change of parameters).  
%            param.gamma (optional, multiplier for increasing the parameter L in fista, 1.5 by default)  
%            param.L0 (optional, initial parameter L in fista, 0.1 by default, should be small enough)  
%            param.fixed\_step (deactive the line search for L in fista and use param.L0 instead)  
%            param.linesearch\_mode (line-search scheme when ista=true:  
%                      0: default, monotonic backtracking scheme  
%                      1: monotonic backtracking scheme, with restart at each iteration  
%                      2: Barzilai-Borwein step sizes (similar to SparSA by Wright et al.)  
%                      3: non-monotonic backtracking  
%            param.compute\_gram (optional, pre-compute X^TX, false by default).  
%            param.intercept (optional, do not regularize last row of W, false by default).  
%            param.ista (optional, use ista instead of fista, false by default).  
%            param.subgrad (optional, if not param.ista, use subradient descent instead of fista, false by default).  
%            param.a, param.b (optional, if param.subgrad, the gradient step is a/(t+b)  
%            also similar options as mexProximalFlat  
%  
%            the function also implements the ADMM algorithm via an option param.admm=true. It is not documented  
%            and you need to look at the source code to use it.  
%  
% Output:  W:  double dense p x n matrix or p x Nn matrix (for multi-logistic loss)  
%          optim: optional, double dense 4 x n matrix.  
%              first row: values of the objective functions.  
%              third row: values of the relative duality gap (if available)  
%              fourth row: number of iterations  
%  
% Author: Julien Mairal, 2010

The following piece of code illustrates how to use this function.

format compact;  
randn('seed',0);  
param.numThreads=-1; % all cores (-1 by default)  
param.verbose=true;   % verbosity, false by default  
param.lambda=0.05; % regularization parameter  
param.it0=10;      % frequency for duality gap computations  
param.max\_it=200; % maximum number of iterations  
param.L0=0.1;  
param.tol=1e-3;  
param.intercept=false;  
param.pos=false;  
param.ista=false;  
  
X=randn(100,200);  
X=X-repmat(mean(X),[size(X,1) 1]);  
X=mexNormalize(X);  
Y=randn(100,1);  
Y=Y-repmat(mean(Y),[size(Y,1) 1]);  
Y=mexNormalize(Y);  
W0=zeros(size(X,2),size(Y,2));  
% Regression experiments   
% 100 regression problems with the same design matrix X.  
fprintf('\nVarious regression experiments\n');  
param.compute\_gram=true;  
fprintf('\nFISTA + Regression l1\n');  
param.loss='square';  
param.regul='l1';  
% param.regul='group-lasso-l2';  
% param.size\_group=10;  
tic  
[W optim\_info]=mexFistaFlat(Y,X,W0,param);  
t=toc;  
fprintf('mean loss: %f, mean relative duality\_gap: %f, time: %f, number of iterations: %f\n',mean(optim\_info(1,:)),mean(optim\_info(3,:)),t,mean(optim\_info(4,:)));  
  
param.regul='l1';  
fprintf('\nISTA + Regression l1\n');  
param.ista=true;  
tic  
[W optim\_info]=mexFistaFlat(Y,X,W0,param);  
t=toc;  
fprintf('mean loss: %f, mean relative duality\_gap: %f, time: %f, number of iterations: %f\n',mean(optim\_info(1,:)),mean(optim\_info(3,:)),t,mean(optim\_info(4,:)));  
  
param.regul='l1';  
fprintf('\nISTA + Regression l1 + Barzilai Borwein (SPARSA)\n');  
param.ista=true;  
param.barzilaiborwein=true;  
tic  
[W optim\_info]=mexFistaFlat(Y,X,W0,param);  
t=toc;  
fprintf('mean loss: %f, mean relative duality\_gap: %f, time: %f, number of iterations: %f\n',mean(optim\_info(1,:)),mean(optim\_info(3,:)),t,mean(optim\_info(4,:)));  
param.barzilaiborwein=false;  
  
  
fprintf('\nSubgradient Descent + Regression l1\n');  
param.ista=false;  
param.subgrad=true;  
param.a=0.1;  
param.b=1000; % arbitrary parameters  
max\_it=param.max\_it;  
it0=param.it0;  
param.max\_it=500;  
param.it0=50;  
tic  
[W optim\_info]=mexFistaFlat(Y,X,W0,param);  
t=toc;  
param.subgrad=false;  
param.max\_it=max\_it;  
param.it0=it0;  
fprintf('mean loss: %f, mean relative duality\_gap: %f, time: %f, number of iterations: %f\n',mean(optim\_info(1,:)),mean(optim\_info(3,:)),t,mean(optim\_info(4,:)));  
  
fprintf('\nFISTA + Regression l2\n');  
param.regul='l2';  
tic  
[W optim\_info]=mexFistaFlat(Y,X,W0,param);  
t=toc;  
fprintf('mean loss: %f, mean relative duality\_gap: %f, time: %f, number of iterations: %f\n',mean(optim\_info(1,:)),mean(optim\_info(3,:)),t,mean(optim\_info(4,:)));  
  
fprintf('\nFISTA + Regression l2 + sparse feature matrix\n');  
param.regul='l2';  
tic  
[W optim\_info]=mexFistaFlat(Y,sparse(X),W0,param);  
t=toc;  
fprintf('mean loss: %f, mean relative duality\_gap: %f, time: %f, number of iterations: %f\n',mean(optim\_info(1,:)),mean(optim\_info(3,:)),t,mean(optim\_info(4,:)));  
  
  
fprintf('\nFISTA + Regression Elastic-Net\n');  
param.regul='elastic-net';  
param.lambda2=0.1;  
tic  
[W optim\_info]=mexFistaFlat(Y,X,W0,param);  
t=toc;  
fprintf('mean loss: %f, time: %f, number of iterations: %f\n',mean(optim\_info(1,:)),t,mean(optim\_info(4,:)));  
  
fprintf('\nFISTA + Group Lasso L2\n');  
param.regul='group-lasso-l2';  
param.size\_group=2;  % all the groups are of size 2  
tic  
[W optim\_info]=mexFistaFlat(Y,X,W0,param);  
t=toc;  
fprintf('mean loss: %f, mean relative duality\_gap: %f, time: %f, number of iterations: %f\n',mean(optim\_info(1,:)),mean(optim\_info(3,:)),t,mean(optim\_info(4,:)));  
  
fprintf('\nFISTA + Group Lasso L2 with variable size of groups \n');  
param.regul='group-lasso-l2';  
param2=param;  
param2.groups=int32(randi(5,1,size(X,2)));  % all the groups are of size 2  
param2.lambda=10\*param2.lambda;  
tic  
[W optim\_info]=mexFistaFlat(Y,X,W0,param2);  
t=toc;  
fprintf('mean loss: %f, mean relative duality\_gap: %f, time: %f, number of iterations: %f\n',mean(optim\_info(1,:)),mean(optim\_info(3,:)),t,mean(optim\_info(4,:)));  
  
fprintf('\nFISTA + Trace Norm\n');  
param.regul='trace-norm-vec';  
param.size\_group=5;  
tic  
[W optim\_info]=mexFistaFlat(Y,X,W0,param);  
t=toc;  
fprintf('mean loss: %f, mean relative duality\_gap: %f, time: %f, number of iterations: %f\n',mean(optim\_info(1,:)),mean(optim\_info(3,:)),t,mean(optim\_info(4,:)));  
  
fprintf('\nFISTA + Regression Fused-Lasso\n');  
param.regul='fused-lasso';  
param.lambda2=0.1;  
param.lambda3=0.1; %  
tic  
[W optim\_info]=mexFistaFlat(Y,X,W0,param);  
t=toc;  
fprintf('mean loss: %f, time: %f, number of iterations: %f\n',mean(optim\_info(1,:)),t,mean(optim\_info(4,:)));  
  
fprintf('\nFISTA + Regression no regularization\n');  
param.regul='none';  
tic  
[W optim\_info]=mexFistaFlat(Y,X,W0,param);  
t=toc;  
fprintf('mean loss: %f, time: %f, number of iterations: %f\n',mean(optim\_info(1,:)),t,mean(optim\_info(4,:)));  
  
fprintf('\nFISTA + Regression l1 with intercept \n');  
param.intercept=true;  
param.regul='l1';  
tic  
[W optim\_info]=mexFistaFlat(Y,[X ones(size(X,1),1)],[W0; zeros(1,size(W0,2))],param); % adds a column of ones to X for the intercept  
t=toc;  
fprintf('mean loss: %f, mean relative duality\_gap: %f, time: %f, number of iterations: %f\n',mean(optim\_info(1,:)),mean(optim\_info(3,:)),t,mean(optim\_info(4,:)));  
  
fprintf('\nFISTA + Regression l1 with intercept+ non-negative \n');  
param.pos=true;  
param.regul='l1';  
tic  
[W optim\_info]=mexFistaFlat(Y,[X ones(size(X,1),1)],[W0; zeros(1,size(W0,2))],param);  
t=toc;  
fprintf('mean loss: %f, time: %f, number of iterations: %f\n',mean(optim\_info(1,:)),t,mean(optim\_info(4,:)));  
param.pos=false;  
param.intercept=false;  
  
fprintf('\nISTA + Regression l0\n');  
param.regul='l0';  
tic  
[W optim\_info]=mexFistaFlat(Y,X,W0,param);  
t=toc;  
fprintf('mean loss: %f, time: %f, number of iterations: %f\n',mean(optim\_info(1,:)),t,mean(optim\_info(4,:)));  
  
fprintf('\nOne classification experiment\n');  
Y=2\*double(randn(100,1) > 0)-1;  
fprintf('\nFISTA + Logistic l1\n');  
param.regul='l1';  
param.loss='logistic';  
param.lambda=0.01;  
tic  
[W optim\_info]=mexFistaFlat(Y,X,W0,param);  
t=toc;  
fprintf('mean loss: %f, mean relative duality\_gap: %f, time: %f, number of iterations: %f\n',mean(optim\_info(1,:)),mean(optim\_info(3,:)),t,mean(optim\_info(4,:)));  
% can be used of course with other regularization functions, intercept,...  
param.regul='l1';  
param.loss='weighted-logistic';  
param.lambda=0.01;  
fprintf('\nFISTA + weighted Logistic l1 + sparse matrix\n');  
tic  
[W optim\_info]=mexFistaFlat(Y,X,W0,param);  
t=toc;  
% can be used of course with other regularization functions, intercept,...  
  
param.loss='logistic';  
fprintf('\nFISTA + Logistic l1 + sparse matrix\n');  
tic  
[W optim\_info]=mexFistaFlat(Y,sparse(X),W0,param);  
t=toc;  
fprintf('mean loss: %f, mean relative duality\_gap: %f, time: %f, number of iterations: %f\n',mean(optim\_info(1,:)),mean(optim\_info(3,:)),t,mean(optim\_info(4,:)));  
% can be used of course with other regularization functions, intercept,...  
  
% Multi-Class classification  
Y=double(ceil(5\*rand(100,1000))-1);  
param.loss='multi-logistic';  
fprintf('\nFISTA + Multi-Class Logistic l1\n');  
tic  
nclasses=max(Y(:))+1;  
W0=zeros(size(X,2),nclasses\*size(Y,2));  
[W optim\_info]=mexFistaFlat(Y,X,W0,param);  
t=toc;  
fprintf('mean loss: %f, mean relative duality\_gap: %f, time: %f, number of iterations: %f\n',mean(optim\_info(1,:)),mean(optim\_info(3,:)),t,mean(optim\_info(4,:)));  
pause  
% can be used of course with other regularization functions, intercept,...  
  
% Multi-Task regression  
Y=randn(100,100);  
Y=Y-repmat(mean(Y),[size(Y,1) 1]);  
Y=mexNormalize(Y);  
param.compute\_gram=false;  
W0=zeros(size(X,2),size(Y,2));  
param.loss='square';  
fprintf('\nFISTA + Regression l1l2 \n');  
param.regul='l1l2';  
tic  
[W optim\_info]=mexFistaFlat(Y,X,W0,param);  
toc  
fprintf('mean loss: %f, mean relative duality\_gap: %f, time: %f, number of iterations: %f\n',mean(optim\_info(1,:)),mean(optim\_info(3,:)),t,mean(optim\_info(4,:)));  
  
fprintf('\nFISTA + Regression l1linf \n');  
param.regul='l1linf';  
tic  
[W optim\_info]=mexFistaFlat(Y,X,W0,param);  
toc  
fprintf('mean loss: %f, mean relative duality\_gap: %f, time: %f, number of iterations: %f\n',mean(optim\_info(1,:)),mean(optim\_info(3,:)),t,mean(optim\_info(4,:)));  
  
fprintf('\nFISTA + Regression l1l2 + l1 \n');  
param.regul='l1l2+l1';  
param.lambda2=0.1;  
tic  
[W optim\_info]=mexFistaFlat(Y,X,W0,param);  
toc  
fprintf('mean loss: %f, time: %f, number of iterations: %f\n',mean(optim\_info(1,:)),t,mean(optim\_info(4,:)));  
  
fprintf('\nFISTA + Regression l1linf + l1 \n');  
param.regul='l1linf+l1';  
param.lambda2=0.1;  
tic  
[W optim\_info]=mexFistaFlat(Y,X,W0,param);  
toc  
fprintf('mean loss: %f, time: %f, number of iterations: %f\n',mean(optim\_info(1,:)),t,mean(optim\_info(4,:)));  
  
fprintf('\nFISTA + Regression l1linf + row + columns \n');  
param.regul='l1linf-row-column';  
param.lambda2=0.1;  
tic  
[W optim\_info]=mexFistaFlat(Y,X,W0,param);  
toc  
fprintf('mean loss: %f, mean relative duality\_gap: %f, time: %f, number of iterations: %f\n',mean(optim\_info(1,:)),mean(optim\_info(3,:)),t,mean(optim\_info(4,:)));  
  
% Multi-Task Classification  
fprintf('\nFISTA + Logistic + l1l2 \n');  
param.regul='l1l2';  
param.loss='logistic';  
Y=2\*double(randn(100,100) > 0)-1;  
tic  
[W optim\_info]=mexFistaFlat(Y,X,W0,param);  
toc  
fprintf('mean loss: %f, mean relative duality\_gap: %f, time: %f, number of iterations: %f\n',mean(optim\_info(1,:)),mean(optim\_info(3,:)),t,mean(optim\_info(4,:)));  
% Multi-Class + Multi-Task Regularization  
  
fprintf('\nFISTA + Multi-Class Logistic l1l2 \n');  
Y=double(ceil(5\*rand(100,1000))-1);  
param.loss='multi-logistic';  
param.regul='l1l2';  
tic  
nclasses=max(Y(:))+1;  
W0=zeros(size(X,2),nclasses\*size(Y,2));  
[W optim\_info]=mexFistaFlat(Y,X,W0,param);  
t=toc;  
fprintf('mean loss: %f, mean relative duality\_gap: %f, time: %f, number of iterations: %f\n',mean(optim\_info(1,:)),mean(optim\_info(3,:)),t,mean(optim\_info(4,:)));  
% can be used of course with other regularization functions, intercept,...

### 5.9  Function mexFistaTree

Given a matrix X=[x^1^,…,x^p^]^T^ in ℝ^m × p^, and a matrix Y=[y^1^,…,y^n^], it solves the optimization problems presented in the previous section, with the same regularization functions as mexProximalTree.
see usage details below:

%   
% Usage: [W [optim]]=mexFistaTree(Y,X,W0,tree,param);  
%  
% Name: mexFistaTree  
%  
% Description: mexFistaTree solves sparse regularized problems.  
%         X is a design matrix of size m x p  
%         X=[x^1,...,x^n]', where the x\_i's are the rows of X  
%         Y=[y^1,...,y^n] is a matrix of size m x n  
%         It implements the algorithms FISTA, ISTA and subgradient descent for solving  
%  
%           min\_W  loss(W) + lambda psi(W)  
%            
%         The function psi are those used by mexProximalTree (see documentation)  
%         for the loss functions, see the documentation of mexFistaFlat  
%  
%         This function can also handle intercepts (last row of W is not regularized),  
%         and/or non-negativity constraints on W and sparse matrices X  
%  
% Inputs: Y:  double dense m x n matrix  
%         X:  double dense or sparse m x p matrix     
%         W0:  double dense p x n matrix or p x Nn matrix (for multi-logistic loss)  
%              initial guess  
%         tree: struct (see documentation of mexProximalTree)  
%         param: struct  
%            param.loss (choice of loss, see above)  
%            param.regul (choice of regularization, see function mexProximalFlat)  
%            param.lambda (regularization parameter)  
%            param.lambda2 (optional, regularization parameter, 0 by default)  
%            param.lambda3 (optional, regularization parameter, 0 by default)  
%            param.verbose (optional, verbosity level, false by default)  
%            param.pos (optional, adds positivity constraints on the  
%                coefficients, false by default)  
%            param.transpose (optional, transpose the matrix in the regularization function)  
%            param.size\_group (optional, for regularization functions assuming a group  
%                 structure)  
%            param.numThreads (optional, number of threads for exploiting  
%                multi-core / multi-cpus. By default, it takes the value -1,  
%                which automatically selects all the available CPUs/cores).  
%            param.max\_it (optional, maximum number of iterations, 100 by default)  
%            param.it0 (optional, frequency for computing duality gap, every 10 iterations by default)  
%            param.tol (optional, tolerance for stopping criteration, which is a relative duality gap  
%                if it is available, or a relative change of parameters).  
%            param.gamma (optional, multiplier for increasing the parameter L in fista, 1.5 by default)  
%            param.L0 (optional, initial parameter L in fista, 0.1 by default, should be small enough)  
%            param.fixed\_step (deactive the line search for L in fista and use param.L0 instead)  
%            param.compute\_gram (optional, pre-compute X^TX, false by default).  
%            param.intercept (optional, do not regularize last row of W, false by default).  
%            param.ista (optional, use ista instead of fista, false by default).  
%            param.subgrad (optional, if not param.ista, use subradient descent instead of fista, false by default).  
%            param.a, param.b (optional, if param.subgrad, the gradient step is a/(t+b)  
%            also similar options as mexProximalTree  
%  
%            the function also implements the ADMM algorithm via an option param.admm=true. It is not documented  
%            and you need to look at the source code to use it.  
%  
% Output:  W:  double dense p x n matrix or p x Nn matrix (for multi-logistic loss)  
%          optim: optional, double dense 4 x n matrix.  
%              first row: values of the objective functions.  
%              third row: values of the relative duality gap (if available)  
%              fourth row: number of iterations  
%  
% Author: Julien Mairal, 2010

The following piece of code illustrates how to use this function.

format compact;  
param.num\_threads=-1; % all cores (-1 by default)  
param.verbose=false;   % verbosity, false by default  
param.lambda=0.001; % regularization parameter  
param.it0=10;      % frequency for duality gap computations  
param.max\_it=200; % maximum number of iterations  
param.L0=0.1;  
param.tol=1e-5;  
param.intercept=false;  
param.pos=false;  
  
% Example 2 of tree structure  
% tree structured groups:  
% g1= {0 1 2 3 4 5 6 7 8 9}    root(g1) = { };  
% g2= {0 1 2 3 4 5}            root(g2) = {0 1 2};  
% g3= {3 4}                    root(g3) = {3 4};  
% g4= {5}                      root(g4) = {5};  
% g5= {6 7 8 9}                root(g5) = { };  
% g6= {6 7}                    root(g6) = {6 7};  
% g7= {8 9}                    root(g7) = {8};  
% g8 = {9}                     root(g8) = {9};  
tree.own\_variables=  int32([0 0 3 5 6 6 8 9]);   % pointer to the first variable of each group  
tree.N\_own\_variables=int32([0 3 2 1 0 2 1 1]); % number of "root" variables in each group  
tree.eta\_g=[1 1 1 2 2 2 2.5 2.5];  
tree.groups=sparse([0 0 0 0 0 0 0 0; ...  
                    1 0 0 0 0 0 0 0; ...  
                    0 1 0 0 0 0 0 0; ...  
                    0 1 0 0 0 0 0 0; ...  
                    1 0 0 0 0 0 0 0; ...  
                    0 0 0 0 1 0 0 0; ...  
                    0 0 0 0 1 0 0 0; ...  
                    0 0 0 0 0 0 1 0]);  % first group should always be the root of the tree  
  
X=randn(100,10);  
X=X-repmat(mean(X),[size(X,1) 1]);  
X=mexNormalize(X);  
Y=randn(100,100);  
Y=Y-repmat(mean(Y),[size(Y,1) 1]);  
Y=mexNormalize(Y);  
W0=zeros(size(X,2),size(Y,2));  
% Regression experiments   
% 100 regression problems with the same design matrix X.  
fprintf('\nVarious regression experiments\n');  
param.compute\_gram=true;  
fprintf('\nFISTA + Regression tree-l2\n');  
param.loss='square';  
param.regul='tree-l2';  
tic  
[W optim\_info]=mexFistaTree(Y,X,W0,tree,param);  
t=toc;  
fprintf('mean loss: %f, time: %f, number of iterations: %f\n',mean(optim\_info(1,:)),t,mean(optim\_info(4,:)));  
  
fprintf('\nFISTA + Regression tree-linf\n');  
param.regul='tree-linf';  
tic  
[W optim\_info]=mexFistaTree(Y,X,W0,tree,param);  
t=toc;  
fprintf('mean loss: %f, mean relative duality\_gap: %f, time: %f, number of iterations: %f\n',mean(optim\_info(1,:)),mean(optim\_info(3,:)),t,mean(optim\_info(4,:)));  
  
% works also with non tree-structured regularization. tree is ignored  
fprintf('\nFISTA + Regression Fused-Lasso\n');  
param.regul='fused-lasso';  
param.lambda2=0.001;  
param.lambda3=0.001; %  
tic  
[W optim\_info]=mexFistaTree(Y,X,W0,tree,param);  
t=toc;  
fprintf('mean loss: %f, time: %f, number of iterations: %f\n',mean(optim\_info(1,:)),t,mean(optim\_info(4,:)));  
  
fprintf('\nISTA + Regression tree-l0\n');  
param.regul='tree-l0';  
tic  
[W optim\_info]=mexFistaTree(Y,X,W0,tree,param);  
t=toc;  
fprintf('mean loss: %f, time: %f, number of iterations: %f\n',mean(optim\_info(1,:)),t,mean(optim\_info(4,:)));  
  
fprintf('\nFISTA + Regression tree-l2 with intercept \n');  
param.intercept=true;  
param.regul='tree-l2';  
tic  
[W optim\_info]=mexFistaTree(Y,[X ones(size(X,1),1)],[W0; zeros(1,size(W0,2))],tree,param);  
t=toc;  
fprintf('mean loss: %f, time: %f, number of iterations: %f\n',mean(optim\_info(1,:)),t,mean(optim\_info(4,:)));  
param.intercept=false;  
  
% Classification  
fprintf('\nOne classification experiment\n');  
Y=2\*double(randn(100,size(Y,2)) > 0)-1;  
fprintf('\nFISTA + Logistic + tree-linf\n');  
param.regul='tree-linf';  
param.loss='logistic';  
param.lambda=0.001;  
tic  
[W optim\_info]=mexFistaTree(Y,X,W0,tree,param);  
t=toc;  
fprintf('mean loss: %f, mean relative duality\_gap: %f, time: %f, number of iterations: %f\n',mean(optim\_info(1,:)),mean(optim\_info(3,:)),t,mean(optim\_info(4,:)));  
% can be used of course with other regularization functions, intercept,...  
  
% Multi-Class classification  
Y=double(ceil(5\*rand(100,size(Y,2)))-1);  
param.loss='multi-logistic';  
param.regul='tree-l2';  
fprintf('\nFISTA + Multi-Class Logistic + tree-l2 \n');  
tic  
nclasses=max(Y(:))+1;  
W0=zeros(size(X,2),nclasses\*size(Y,2));  
[W optim\_info]=mexFistaTree(Y,X,W0,tree,param);  
t=toc;  
fprintf('mean loss: %f, time: %f, number of iterations: %f\n',mean(optim\_info(1,:)),t,mean(optim\_info(4,:)));  
% can be used of course with other regularization functions, intercept,...  
  
% Multi-Task regression  
Y=randn(100,size(Y,2));  
Y=Y-repmat(mean(Y),[size(Y,1) 1]);  
Y=mexNormalize(Y);  
param.compute\_gram=false;  
param.verbose=true;   % verbosity, false by default  
W0=zeros(size(X,2),size(Y,2));  
param.loss='square';  
fprintf('\nFISTA + Regression multi-task-tree \n');  
param.regul='multi-task-tree';  
param.lambda2=0.001;  
tic  
[W optim\_info]=mexFistaTree(Y,X,W0,tree,param);  
toc  
fprintf('mean loss: %f, mean relative duality\_gap: %f, time: %f, number of iterations: %f\n',mean(optim\_info(1,:)),mean(optim\_info(3,:)),t,mean(optim\_info(4,:)));  
  
% Multi-Task Classification  
fprintf('\nFISTA + Logistic + multi-task-tree \n');  
param.regul='multi-task-tree';  
param.lambda2=0.001;  
param.loss='logistic';  
Y=2\*double(randn(100,size(Y,2)) > 0)-1;  
tic  
[W optim\_info]=mexFistaTree(Y,X,W0,tree,param);  
toc  
fprintf('mean loss: %f, mean relative duality\_gap: %f, time: %f, number of iterations: %f\n',mean(optim\_info(1,:)),mean(optim\_info(3,:)),t,mean(optim\_info(4,:)));  
  
% Multi-Class + Multi-Task Regularization  
param.verbose=false;  
fprintf('\nFISTA + Multi-Class Logistic +multi-task-tree \n');  
Y=double(ceil(5\*rand(100,size(Y,2)))-1);  
param.loss='multi-logistic';  
param.regul='multi-task-tree';  
tic  
nclasses=max(Y(:))+1;  
W0=zeros(size(X,2),nclasses\*size(Y,2));  
[W optim\_info]=mexFistaTree(Y,X,W0,tree,param);  
t=toc;  
fprintf('mean loss: %f, mean relative duality\_gap: %f, time: %f, number of iterations: %f\n',mean(optim\_info(1,:)),mean(optim\_info(3,:)),t,mean(optim\_info(4,:)));  
% can be used of course with other regularization functions, intercept,...  
  
fprintf('\nFISTA + Multi-Class Logistic +multi-task-tree + sparse matrix \n');  
tic  
nclasses=max(Y(:))+1;  
W0=zeros(size(X,2),nclasses\*size(Y,2));  
[W optim\_info]=mexFistaTree(Y,sparse(X),W0,tree,param);  
t=toc;  
fprintf('mean loss: %f, mean relative duality\_gap: %f, time: %f, number of iterations: %f\n',mean(optim\_info(1,:)),mean(optim\_info(3,:)),t,mean(optim\_info(4,:)));

### 5.10  Function mexFistaGraph

Given a matrix X=[x^1^,…,x^p^]^T^ in ℝ^m × p^, and a matrix Y=[y^1^,…,y^n^], it solves the optimization problems presented in the previous section, with the same regularization functions as mexProximalGraph.
see usage details below:

%   
% Usage: [W [optim]]=mexFistaGraph(Y,X,W0,graph,param);  
%  
% Name: mexFistaGraph  
%  
% Description: mexFistaGraph solves sparse regularized problems.  
%         X is a design matrix of size m x p  
%         X=[x^1,...,x^n]', where the x\_i's are the rows of X  
%         Y=[y^1,...,y^n] is a matrix of size m x n  
%         It implements the algorithms FISTA, ISTA and subgradient descent.  
%  
%         It implements the algorithms FISTA, ISTA and subgradient descent for solving  
%  
%           min\_W  loss(W) + lambda psi(W)  
%            
%         The function psi are those used by mexProximalGraph (see documentation)  
%         for the loss functions, see the documentation of mexFistaFlat  
%           
%         This function can also handle intercepts (last row of W is not regularized),  
%         and/or non-negativity constraints on W.  
%  
% Inputs: Y:  double dense m x n matrix  
%         X:  double dense or sparse m x p matrix     
%         W0:  double dense p x n matrix or p x Nn matrix (for multi-logistic loss)  
%              initial guess  
%         graph: struct (see documentation of mexProximalGraph)  
%         param: struct  
%            param.loss (choice of loss, see above)  
%            param.regul (choice of regularization, see function mexProximalFlat)  
%            param.lambda (regularization parameter)  
%            param.lambda2 (optional, regularization parameter, 0 by default)  
%            param.lambda3 (optional, regularization parameter, 0 by default)  
%            param.verbose (optional, verbosity level, false by default)  
%            param.pos (optional, adds positivity constraints on the  
%                coefficients, false by default)  
%            param.numThreads (optional, number of threads for exploiting  
%                multi-core / multi-cpus. By default, it takes the value -1,  
%                which automatically selects all the available CPUs/cores).  
%            param.max\_it (optional, maximum number of iterations, 100 by default)  
%            param.it0 (optional, frequency for computing duality gap, every 10 iterations by default)  
%            param.tol (optional, tolerance for stopping criteration, which is a relative duality gap  
%                if it is available, or a relative change of parameters).  
%            param.gamma (optional, multiplier for increasing the parameter L in fista, 1.5 by default)  
%            param.L0 (optional, initial parameter L in fista, 0.1 by default, should be small enough)  
%            param.fixed\_step (deactive the line search for L in fista and use param.L0 instead)  
%            param.compute\_gram (optional, pre-compute X^TX, false by default).  
%            param.intercept (optional, do not regularize last row of W, false by default).  
%            param.ista (optional, use ista instead of fista, false by default).  
%            param.subgrad (optional, if not param.ista, use subradient descent instead of fista, false by default).  
%            param.a, param.b (optional, if param.subgrad, the gradient step is a/(t+b)  
%            also similar options as mexProximalTree  
%  
%            the function also implements the ADMM algorithm via an option param.admm=true. It is not documented  
%            and you need to look at the source code to use it.  
%  
%  
% Output:  W:  double dense p x n matrix or p x Nn matrix (for multi-logistic loss)  
%          optim: optional, double dense 4 x n matrix.  
%              first row: values of the objective functions.  
%              third row: values of the relative duality gap (if available)  
%              fourth row: number of iterations  
%  
% Author: Julien Mairal, 2010

The following piece of code illustrates how to use this function.

clear all;  
randn('seed',0);  
format compact;  
param.num\_threads=-1; % all cores (-1 by default)  
param.verbose=false;   % verbosity, false by default  
param.lambda=0.1; % regularization parameter  
param.it0=1;      % frequency for duality gap computations  
param.max\_it=100; % maximum number of iterations  
param.L0=0.1;  
param.tol=1e-5;  
param.intercept=false;  
param.pos=false;  
  
graph.eta\_g=[1 1 1 1 1];  
graph.groups=sparse([0 0 0 1 0;  
                     0 0 0 0 0;  
                     0 0 0 0 0;  
                     0 0 0 0 0;  
                     0 0 1 0 0]);   % g5 is included in g3, and g2 is included in g4  
graph.groups\_var=sparse([1 0 0 0 0;  
                         1 0 0 0 0;  
                         1 0 0 0 0 ;  
                         1 1 0 0 0;  
                         0 1 0 1 0;  
                         0 1 0 1 0;  
                         0 1 0 0 1;  
                         0 0 0 0 1;  
                         0 0 0 0 1;  
                         0 0 1 0 0]); % represents direct inclusion relations   
  
X=randn(100,10);  
param.verbose=true;  
%X=eye(10);  
X=X-repmat(mean(X),[size(X,1) 1]);  
X=mexNormalize(X);  
Y=randn(100,1);  
Y=Y-repmat(mean(Y),[size(Y,1) 1]);  
Y=mexNormalize(Y);  
W0=zeros(size(X,2),size(Y,2));  
% Regression experiments   
% 100 regression problems with the same design matrix X.  
fprintf('\nVarious regression experiments\n');  
param.compute\_gram=true;  
fprintf('\nFISTA + Regression graph\n');  
param.loss='square';  
param.regul='graph';  
tic  
[W optim\_info]=mexFistaGraph(Y,X,W0,graph,param);  
t=toc;  
fprintf('mean loss: %f, mean relative duality\_gap: %f, time: %f, number of iterations: %f\n',mean(optim\_info(1,:)),mean(optim\_info(3,:)),t,mean(optim\_info(4,:)));  
  
fprintf('\nADMM + Regression graph\n');  
param.admm=true;  
param.lin\_admm=true;  
param.c=1;  
param.delta=1;  
tic  
[W optim\_info]=mexFistaGraph(Y,X,W0,graph,param);  
t=toc;  
fprintf('mean loss: %f, stopping criterion: %f, time: %f, number of iterations: %f\n',mean(optim\_info(1,:)),mean(optim\_info(3,:)),t,mean(optim\_info(4,:)));  
  
param.admm=false;  
param.max\_it=5;  
param.it0=1;  
tic  
[W optim\_info]=mexFistaGraph(Y,X,W,graph,param);  
t=toc;  
fprintf('mean loss: %f, mean relative duality\_gap: %f, time: %f, number of iterations: %f\n',mean(optim\_info(1,:)),mean(optim\_info(3,:)),t,mean(optim\_info(4,:)));  
  
% works also with non graph-structured regularization. graph is ignored  
fprintf('\nFISTA + Regression Fused-Lasso\n');  
param.regul='fused-lasso';  
param.lambda2=0.01;  
param.lambda3=0.01; %  
tic  
[W optim\_info]=mexFistaGraph(Y,X,W0,graph,param);  
t=toc;  
fprintf('mean loss: %f, time: %f, number of iterations: %f\n',mean(optim\_info(1,:)),t,mean(optim\_info(4,:)));  
  
fprintf('\nFISTA + Regression graph with intercept \n');  
param.intercept=true;  
param.regul='graph';  
tic  
[W optim\_info]=mexFistaGraph(Y,[X ones(size(X,1),1)],[W0; zeros(1,size(W0,2))],graph,param);  
t=toc;  
fprintf('mean loss: %f, mean relative duality\_gap: %f, time: %f, number of iterations: %f\n',mean(optim\_info(1,:)),mean(optim\_info(3,:)),t,mean(optim\_info(4,:)));  
param.intercept=false;  
  
% Classification  
fprintf('\nOne classification experiment\n');  
Y=2\*double(randn(100,size(Y,2)) > 0)-1;  
fprintf('\nFISTA + Logistic + graph-linf\n');  
param.regul='graph';  
param.loss='logistic';  
param.lambda=0.01;  
tic  
[W optim\_info]=mexFistaGraph(Y,X,W0,graph,param);  
t=toc;  
fprintf('mean loss: %f, mean relative duality\_gap: %f, time: %f, number of iterations: %f\n',mean(optim\_info(1,:)),mean(optim\_info(3,:)),t,mean(optim\_info(4,:)));  
% can be used of course with other regularization functions, intercept,...  
  
% Multi-Class classification  
Y=double(ceil(5\*rand(100,size(Y,2)))-1);  
param.loss='multi-logistic';  
param.regul='graph';  
fprintf('\nFISTA + Multi-Class Logistic + graph \n');  
tic  
nclasses=max(Y(:))+1;  
W0=zeros(size(X,2),nclasses\*size(Y,2));  
[W optim\_info]=mexFistaGraph(Y,X,W0,graph,param);  
t=toc;  
fprintf('mean loss: %f, mean relative duality\_gap: %f, time: %f, number of iterations: %f\n',mean(optim\_info(1,:)),mean(optim\_info(3,:)),t,mean(optim\_info(4,:)));  
% can be used of course with other regularization functions, intercept,...  
  
% Multi-Task regression  
Y=randn(100,size(Y,2));  
Y=Y-repmat(mean(Y),[size(Y,1) 1]);  
Y=mexNormalize(Y);  
param.compute\_gram=false;  
param.verbose=true;   % verbosity, false by default  
W0=zeros(size(X,2),size(Y,2));  
param.loss='square';  
fprintf('\nFISTA + Regression multi-task-graph \n');  
param.regul='multi-task-graph';  
param.lambda2=0.01;  
tic  
[W optim\_info]=mexFistaGraph(Y,X,W0,graph,param);  
toc  
fprintf('mean loss: %f, mean relative duality\_gap: %f, time: %f, number of iterations: %f\n',mean(optim\_info(1,:)),mean(optim\_info(3,:)),t,mean(optim\_info(4,:)));  
  
% Multi-Task Classification  
fprintf('\nFISTA + Logistic + multi-task-graph \n');  
param.regul='multi-task-graph';  
param.lambda2=0.01;  
param.loss='logistic';  
Y=2\*double(randn(100,size(Y,2)) > 0)-1;  
tic  
[W optim\_info]=mexFistaGraph(Y,X,W0,graph,param);  
toc  
fprintf('mean loss: %f, mean relative duality\_gap: %f, time: %f, number of iterations: %f\n',mean(optim\_info(1,:)),mean(optim\_info(3,:)),t,mean(optim\_info(4,:)));  
% Multi-Class + Multi-Task Regularization  
  
param.verbose=false;  
fprintf('\nFISTA + Multi-Class Logistic +multi-task-graph \n');  
Y=double(ceil(5\*rand(100,size(Y,2)))-1);  
param.loss='multi-logistic';  
param.regul='multi-task-graph';  
tic  
nclasses=max(Y(:))+1;  
W0=zeros(size(X,2),nclasses\*size(Y,2));  
[W optim\_info]=mexFistaGraph(Y,X,W0,graph,param);  
t=toc;  
fprintf('mean loss: %f, mean relative duality\_gap: %f, time: %f, number of iterations: %f\n',mean(optim\_info(1,:)),mean(optim\_info(3,:)),t,mean(optim\_info(4,:)));  
% can be used of course with other regularization functions, intercept,...

### 5.11  Function mexFistaPathCoding

Similarly, the toolbox handles the penalties of [24] with the following function

%   
% Usage: [W [optim]]=mexFistaPathCoding(Y,X,W0,DAG,param);  
%  
% Name: mexFistaPathCoding  
%  
% Description: mexFistaPathCoding solves sparse regularized problems for the   
%         path coding penalties of http://arxiv.org/abs/1204.4539  
%         X is a design matrix of size m x p  
%         X=[x^1,...,x^n]', where the x\_i's are the rows of X  
%         Y=[y^1,...,y^n] is a matrix of size m x n  
%         It implements the algorithms FISTA, ISTA and subgradient descent.  
%  
%         It implements the algorithms FISTA, ISTA and subgradient descent for solving  
%  
%           min\_W  loss(W) + lambda psi(W)  
%            
%         The function psi are those used by mexProximalPathCoding (see documentation)  
%         for the loss functions, see the documentation of mexFistaFlat  
%           
%         This function can also handle intercepts (last row of W is not regularized),  
%         and/or non-negativity constraints on W.  
%  
% Inputs: Y:  double dense m x n matrix  
%         X:  double dense or sparse m x p matrix     
%         W0:  double dense p x n matrix or p x Nn matrix (for multi-logistic loss)  
%              initial guess  
%         DAG: struct (see documentation of mexProximalPathCoding)  
%         param: struct  
%            param.loss (choice of loss, see above)  
%            param.regul (choice of regularization, see function mexProximalPathCoding)  
%            param.lambda (regularization parameter)  
%            param.lambda2 (optional, regularization parameter, 0 by default)  
%            param.lambda3 (optional, regularization parameter, 0 by default)  
%            param.verbose (optional, verbosity level, false by default)  
%            param.pos (optional, adds positivity constraints on the  
%                coefficients, false by default)  
%            param.numThreads (optional, number of threads for exploiting  
%                multi-core / multi-cpus. By default, it takes the value -1,  
%                which automatically selects all the available CPUs/cores).  
%            param.max\_it (optional, maximum number of iterations, 100 by default)  
%            param.it0 (optional, frequency for computing duality gap, every 10 iterations by default)  
%            param.tol (optional, tolerance for stopping criteration, which is a relative duality gap  
%                if it is available, or a relative change of parameters).  
%            param.gamma (optional, multiplier for increasing the parameter L in fista, 1.5 by default)  
%            param.L0 (optional, initial parameter L in fista, 0.1 by default, should be small enough)  
%            param.fixed\_step (deactive the line search for L in fista and use param.L0 instead)  
%            param.compute\_gram (optional, pre-compute X^TX, false by default).  
%            param.intercept (optional, do not regularize last row of W, false by default).  
%            param.ista (optional, use ista instead of fista, false by default).  
%            param.subgrad (optional, if not param.ista, use subradient descent instead of fista, false by default).  
%            param.a, param.b (optional, if param.subgrad, the gradient step is a/(t+b)  
%            also similar options as mexProximalPathCoding  
%  
%  
% Output:  W:  double dense p x n matrix or p x Nn matrix (for multi-logistic loss)  
%          optim: optional, double dense 4 x n matrix.  
%              first row: values of the objective functions.  
%              third row: values of the relative duality gap (if available)  
%              fourth row: number of iterations  
%  
% Author: Julien Mairal, 2012

The following piece of code illustrates how to use this function.

clear all;  
rand('seed',0);  
randn('seed',0);  
fprintf('test mexFistaPathCoding\n');  
p=100;  
n=1000;  
% generate a DAG  
G=sprand(p,p,0.05);  
G=mexRemoveCyclesGraph(G);  
fprintf('\n');  
  
% generate a data matrix  
X=randn(n,p);  
X=X-repmat(mean(X),[size(X,1) 1]);  
X=mexNormalize(X);  
Y=randn(n,2);  
Y=Y-repmat(mean(Y),[size(Y,1) 1]);  
Y=mexNormalize(Y);  
W0=zeros(size(X,2),size(Y,2));  
  
% input graph  
graph.weights=G;  
graph.stop\_weights=zeros(1,p);  
graph.start\_weights=10\*ones(1,p);  
  
% FISTA parameters  
param.num\_threads=-1; % all cores (-1 by default)  
param.verbose=true;   % verbosity, false by default  
param.lambda=0.005; % regularization parameter  
param.it0=1;      % frequency for duality gap computations  
param.max\_it=100; % maximum number of iterations  
param.L0=0.01;  
param.tol=1e-4;  
param.precision=10000000;  
param.pos=false;  
  
fprintf('Square Loss + convex path penalty\n');  
param.loss='square';  
param.regul='graph-path-conv';  
tic  
[W1 optim\_info]=mexFistaPathCoding(Y,X,W0,graph,param);  
t=toc;  
fprintf('mean loss: %f, mean relative duality\_gap: %f, time: %f, number of iterations: %f\n',mean(optim\_info(1,:)),mean(optim\_info(3,:)),t,mean(optim\_info(4,:)));  
num=mexCountConnexComponents(graph.weights,W1(:,1));  
fprintf('Num of connected components: %d\n',num);  
  
fprintf('\n');  
fprintf('Square Loss + non-convex path penalty\n');  
param.loss='square';  
param.regul='graph-path-l0';  
param.lambda=0.0001; % regularization parameter  
param.ista=true;  
tic  
[W2 optim\_info]=mexFistaPathCoding(Y,X,W0,graph,param);  
t=toc;  
num=mexCountConnexComponents(graph.weights,W2(:,1));  
fprintf('Num of connected components: %d\n',num);  
  
fprintf('\n');  
fprintf('Note that for non-convex penalties, continuation strategies sometimes perform better:\n');  
tablambda=param.lambda\*sqrt(sqrt(sqrt(2))).^(20:-1:0);  
lambda\_orig=param.lambda;  
tic  
W2=W0;  
for ii = 1:length(tablambda)  
   param.lambda=tablambda(ii);  
   param.verbose=false;  
   [W2]=mexFistaPathCoding(Y,X,W2,graph,param);  
end  
param.verbose=true;  
param.lambda=lambda\_orig;  
[W2 optim\_info]=mexFistaPathCoding(Y,X,W2,graph,param);  
t=toc;  
num=mexCountConnexComponents(graph.weights,W2(:,1));  
param.ista=false;  
fprintf('Num of connected components: %d\n',num);

### 5.12  Function solverPoisson

The following problem is addressed here

min

w ∈ ℝ_+_^p^

p

∑

j=1

x_i_^⊤^w + δ − y_i_ log(x_i_^⊤^w +δ) + λ ψ(w).

%   
% Usage:   [W, [optim]]=solverPoisson(Y,X,W0,param);  
%  
% Name: solverPoisson  
%  
% Description: solverPoisson solves the regularized Poisson regression  
% problem for every column of Y:  
%  
%   min\_w  \sum\_i (x\_i' w) + delta  - y\_i log( x\_i' w + delta) + lambda psi(w) (1)  
%  
% delta should be positive. The method solves (1) for decreasing values of delta,  
% until it reaches the desired target value. The algorithm ISTA is used  
% with Barzilai-Borwein steps.  
%  
% Inputs: Y:  double m x n matrix (non-negative values)  
%         X:  double m x p matrix (non-negative values)  
%         W0:  double p x n matrix (non-negative values)  
%         param: struct  
%            param.tol, param.max\_it, param.verbose, param.L0,  
%            param.it0, param.lambda as for mexFistaFlat  
%            param.regul,  as for mexProximalFlat  
%  
% Output:  
%         W: double p x n matrix (non-negative values)  
  
function [W optim] = solverPoisson(Y,X,W0,param)  
param.ista=true;  
param.intercept=false;  
param.pos=true;  
param.linesearch\_mode=2;  
param.loss='poisson';  
tabdelta=logspace(0,log10(param.delta),-log10(param.delta));  
for delta = tabdelta  
   param2=param;  
   param2.delta=delta;  
   [W optim]=mexFistaFlat(Y,X,W0,param2);  
   W0=W;  
end

The following piece of code illustrates how to use this function.

rand('seed',0);  
randn('seed',0);  
format compact;  
randn('seed',0);  
param.numThreads=-1; % all cores (-1 by default)  
param.verbose=true;   % verbosity, false by default  
param.lambda=0.05; % regularization parameter  
param.it0=10;      % frequency for duality gap computations  
param.max\_it=200; % maximum number of iterations  
param.L0=0.01;  
param.tol=1e-6;  
param.delta=1e-5;  
  
X=rand(1000,2000);  
X=mexNormalize(X);  
Y=rand(1000,1);  
Y=mexNormalize(Y);  
W0=zeros(size(X,2),size(Y,2));  
% Regression experiments   
% 100 regression problems with the same design matrix X.  
fprintf('\nVarious regression experiments\n');  
fprintf('\nFISTA + Regression l1\n');  
param.regul='l1';  
tic  
[W optim\_info]=solverPoisson(Y,X,W0,param);  
t=toc;  
fprintf('mean loss: %f, mean relative duality\_gap: %f, time: %f, number of iterations: %f\n',mean(optim\_info(1,:)),mean(optim\_info(3,:)),t,mean(optim\_info(4,:)));

### 5.13  Function mexIncrementalProx

This implements the incremental solver MISO [25].

%   
% Usage:  [W [optim]]=mexIncrementalProx(y,X,W0,param);  
%  
% Name: mexIncrementalProx  
%  
% Description: mexIncremrentalProx implements the incremental algorithm MISO  
% for composite optimization in a large scale setting.  
%        X is a design matrix of size p x n  
%        y is a vector of size n   
% WARNING, X is transposed compared to the functions mexFista\*, and y is a vector  
%        param.lambda is a vector that contains nlambda different values of the  
%        regularization parameter (it can be a scalar, in that case nlambda=1)  
%        W0: is a dense matrix of size p x nlambda   It is in fact ineffective  
%        in the current release  
%        W: is the output, dense matrix of size p x nlambda  
%  
%         - if param.loss='square' and param.regul corresponds to a regularization  
%           function (currently 'l1' or 'l2'), the following problem is solved  
%           w = argmin (1/n)sum\_{i=1}^n 0.5(y\_i- x\_i^T w)^2 + lambda psi(w)  
%         - if param.loss='logistic' and param.regul corresponds to a regularization  
%           function (currently 'l1' or 'l2'), the following problem is solved  
%           w = argmin (1/n)sum\_{i=1}^n log(1+ exp(-y\_ix\_i^T w)) + lambda psi(w)  
%           Note that here, the y\_i's should be -1 or +1   
%            
%         The current release does not handle intercepts  
%  
% Inputs: y: double dense vector of size n  
%         X: dense or sparse matrix of size p x n  
%         W0: dense matrix of size p x nlambda  
%         param: struct  
%           param.loss (choice of loss)  
%           param.regul (choice of regularization function)  
%           param.lambda : vector of size nlambda  
%           param.epochs: number of passes over the data  
%           param.minibatches: size of the mini-batches: recommended value is 1  
%              if X is dense, and min(n,ceil(1/density)) if X is sparse  
%           param.warm\_restart : (path-following strategy, very efficient when  
%              providing an array of lambdas, false by default)  
%           param.normalized : (optional, can be set to true if the x\_i's have  
%              unit l2-norm, false by default)  
%           param.strategy (optional, 3 by default)  
%                          0: no heuristics, slow  (only for comparison purposes)  
%                          1: adjust the constant L on 5% of the data   
%                          2: adjust the constant L on 5% of the data + unstable heuristics (this strategy does not work)  
%                          3: adjust the constant L on 5% of the data + stable heuristic (this is by far the best choice)  
%           param.numThreads (optional, number of threads)  
%           param.verbose (optional)  
%           param.seed (optional, choice of the random seed)  
%  
% Output:  W:  double dense p x nlambda matrix  
%          optim: optional, double dense 3 x nlambda matrix.  
%              first row: values of the objective functions.  
%              third row: computational time   
%  
% Author: Julien Mairal, 2013

The following piece of code illustrates how to use this function.

clear all;  
n=400000;  
p=1000;  
density=0.01;  
  
% generate random data  
format compact;  
randn('seed',0);  
rand('seed',0);  
X=sprandn(p,n,density);  
mean\_nrm=mean(sqrt(sum(X.^2)));  
X=X/mean\_nrm;  
  
% generate some true model  
z=double(sign(full(sprandn(p,1,0.05))));  
y=X'\*z;  
  
%%%%%%%%%%%%%%%%%%%%%%%%%%%%%%%%%%%%%  
% EXPERIMENT 1: Lasso  
%%%%%%%%%%%%%%%%%%%%%%%%%%%%%%%%%%%%%  
fprintf('EXPERIMENT FOR LASSO\n');  
nrm=sqrt(sum(y.^2));  
y=y+0.01\*nrm\*randn(n,1);    % add noise to the model  
nrm=sqrt(sum(y.^2));  
y=y\*(sqrt(n)/nrm);  
  
% set optimization parameters  
clear param;  
param.regul='l1';        % many other regularization functions are available  
param.loss='square';     % only square and log are available  
param.numThreads=-1;    % uses all possible cores  
param.normalized=false;  % if the columns of X have unit norm, set to true.  
param.strategy=1;        % MISO with all heuristics  
                         % 0: no heuristics, slow  (only for comparison purposes)  
                         % 1: MISO1: adjust the constant L on 5% of the data  (good for non-strongly convex problems)  
                         % 2: adjust the constant L on 5% of the data + unstable heuristics (this strategy does not work)  
                         % 3: MISO2: adjust the constant L on 5% of the data + stable heuristic good for strongly-convex problems  
                         % 4: MISOmu: best for l2 regularization  
param.verbose=true;  
  
% set grid of lambda  
max\_lambda=max(abs(X\*y))/n;  
tablambda=max\_lambda\*(2^(1/4)).^(0:-1:-20);  % order from large to small  
tabepochs=[1 2 3 5 10];  % in this script, we compare the results obtained when changing the number of passes on the data.  
param.lambda=tablambda;  
%% The problem which will be solved is  
%%   min\_beta  1/(2n) ||y-X' beta||\_2^2 + lambda ||beta||\_1  
% the problems for different lambdas are solve INDEPENDENTLY in parallel  
fprintf('EXPERIMENT: ALL LAMBDAS WITHOUT WARM RESTART\n');  
param.warm\_restart=false;  
param.verbose=false;  
param.strategy=1;  
param.minibatches=min(n,ceil(1/density));  % size of the minibatches, requires to store twice the size of X  with strategy=1  
obj=[];  
spar=[];  
for ii=1:length(tabepochs)  
   param.epochs=tabepochs(ii);   % one pass over the data  
   fprintf('EXP WITH %d PASS OVER THE DATA\n',tabepochs(ii));  
   nlambdas=length(param.lambda);  
   Beta0=zeros(p,nlambdas);  
   tic  
   [Beta tmp]=mexIncrementalProx(y,X,Beta0,param);  
   toc  
   fprintf('Objective functions: \n');  
   obj=[obj; tmp(1,:)];  
   obj  
   fprintf('Sparsity: \n');  
   spar=[spar; sum(Beta ~= 0)];  
   spar  
end  
  
% the problems are here solved sequentially with warm restart  
% this seems to be the prefered choice.  
fprintf('EXPERIMENT: SEQUENTIAL LAMBDAS WITH WARM RESTART\n');  
fprintf('A SINGLE CORE IS USED\n');  
param.warm\_restart=true;  
param.num\_threads=1;  
obj=[];  
spar=[];  
for ii=1:length(tabepochs)  
   param.epochs=tabepochs(ii);   % one pass over the data  
   fprintf('EXP WITH %d PASS\n',tabepochs(ii));  
   nlambdas=length(param.lambda);  
   Beta0=zeros(p,nlambdas);  
   tic  
   [Beta tmp]=mexIncrementalProx(y,X,Beta0,param);  
   toc  
   fprintf('Objective functions: \n');  
   obj=[obj; tmp(1,:)];  
   obj  
   fprintf('Sparsity: \n');  
   spar=[spar; sum(Beta ~= 0)];  
   spar  
end  
  
%%%%%%%%%%%%%%%%%%%%%%%%%%%%%%%%%%%%%  
% EXPERIMENT 2: L2 logistic regression   
%%%%%%%%%%%%%%%%%%%%%%%%%%%%%%%%%%%%%  
fprintf('EXPERIMENT FOR LOGISTIC REGRESSION + l2\n');  
y=sign(y);  
param.regul='l2';        % many other regularization functions are available  
param.loss='logistic';     % only square and log are available  
param.num\_threads=-1;    % uses all possible cores  
param.strategy=4;  
param.minibatches=1; % with strategy=4, minibatches is better set to 1  
%param.strategy=3;        % MISO with all heuristics  
fprintf('EXPERIMENT: ALL LAMBDAS WITHOUT WARM RESTART\n');  
param.warm\_restart=false;  
param.verbose=false;  
obj=[];  
spar=[];  
for ii=1:length(tabepochs)  
   param.epochs=tabepochs(ii);   % one pass over the data  
   fprintf('EXP WITH %d PASS\n',tabepochs(ii));  
   nlambdas=length(param.lambda);  
   Beta0=zeros(p,nlambdas);  
   tic  
   [Beta tmp]=mexIncrementalProx(y,X,Beta0,param);  
   toc  
   yR=repmat(y,[1 nlambdas]);  
   fprintf('Objective functions: \n');  
   obj=[obj;tmp(1,:)];  
   obj  
end  
  
  
fprintf('EXPERIMENT FOR LOGISTIC REGRESSION + l2\n');  
y=sign(y);  
param.regul='l2';        % many other regularization functions are available  
param.loss='logistic';     % only square and log are available  
param.num\_threads=-1;    % uses all possible cores  
param.strategy=3;         % MISO2  
param.minibatches=min(n,ceil(1/density));  % size of the minibatches, requires to store twice the size of X   
fprintf('EXPERIMENT: ALL LAMBDAS WITHOUT WARM RESTART\n');  
param.warm\_restart=false;  
param.verbose=false;  
obj=[];  
spar=[];  
for ii=1:length(tabepochs)  
   param.epochs=tabepochs(ii);   % one pass over the data  
   fprintf('EXP WITH %d PASS\n',tabepochs(ii));  
   nlambdas=length(param.lambda);  
   Beta0=zeros(p,nlambdas);  
   tic  
   [Beta tmp]=mexIncrementalProx(y,X,Beta0,param);  
   toc  
   yR=repmat(y,[1 nlambdas]);  
   fprintf('Objective functions: \n');  
   obj=[obj;tmp(1,:)];  
   obj  
end  
  
%%%%%%%%%%%%%%%%%%%%%%%%%%%%%%%%%%%%%  
% EXPERIMENT 3: L1 logistic regression   
%%%%%%%%%%%%%%%%%%%%%%%%%%%%%%%%%%%%%  
  
y=sign(y);  
fprintf('EXPERIMENT FOR LOGISTIC REGRESSION + l1\n');  
param.regul='l1';        % many other regularization functions are available  
param.loss='logistic';     % only square and log are available  
param.num\_threads=-1;    % uses all possible cores  
param.strategy=1;  
param.minibatches=min(n,ceil(1/density));  % size of the minibatches, requires to store twice the size of X   
fprintf('EXPERIMENT: ALL LAMBDAS WITHOUT WARM RESTART\n');  
param.warm\_restart=false;  
param.verbose=false;  
param.lambda=tablambda;  
obj=[];  
spar=[];  
for ii=1:length(tabepochs)  
   param.epochs=tabepochs(ii);   % one pass over the data  
   fprintf('EXP WITH %d PASS\n',tabepochs(ii));  
   nlambdas=length(param.lambda);  
   Beta0=zeros(p,nlambdas);  
   tic  
   [Beta tmp]=mexIncrementalProx(y,X,Beta0,param);  
   toc  
   yR=repmat(y,[1 nlambdas]);  
   fprintf('Objective functions: \n');  
   obj=[obj;tmp(1,:)];  
   obj  
end

### 5.14  Function mexStochasticProx

This implements the stochastic proximal gradient solver [26].

%   
% Usage:  [W [W2]]=mexStochasticProx(y,X,W0,param);  
%  
% Name: mexStochasticProx  
%  
% Description: mexStochasticProx implements a proximal MM stochastic algorithm   
% for composite optimization in a large scale setting.  
%        X is a design matrix of size p x n  
%        y is a vector of size n   
% WARNING, X is transposed compared to the functions mexFista\*, and y is a vector  
%        param.lambda is a vector that contains nlambda different values of the  
%        regularization parameter (it can be a scalar, in that case nlambda=1)  
%        W0: is a dense matrix of size p x nlambda   It is in fact ineffective  
%        in the current release  
%        W: is the output, dense matrix of size p x nlambda  
%  
%         - if param.loss='square' and param.regul corresponds to a regularization  
%           function (currently 'l1' or 'l2'), the following problem is solved  
%           w = argmin (1/n)sum\_{i=1}^n 0.5(y\_i- x\_i^T w)^2 + lambda psi(w)  
%         - if param.loss='logistic' and param.regul corresponds to a regularization  
%           function (currently 'l1' or 'l2'), the following problem is solved  
%           w = argmin (1/n)sum\_{i=1}^n log(1+ exp(-y\_ix\_i^T w)) + lambda psi(w)  
%           Note that here, the y\_i's should be -1 or +1   
%            
%         The current release does not handle intercepts  
%  
% Inputs: y: double dense vector of size n  
%         X: dense or sparse matrix of size p x n  
%         W0: dense matrix of size p x nlambda  
%         param: struct  
%           param.loss (choice of loss)  
%           param.regul (choice of regularization function)  
%           param.lambda : vector of size nlambda  
%           param.iters : number of iterations (n corresponds to one pass over the data)  
%           param.minibatches: size of the mini-batches: recommended value is 1  
%           param.normalized : (optional, can be set to true if the x\_i's have  
%              unit l2-norm, false by default)  
%           param.weighting\_mode : (optional, 1 by default),  
%                0:  w\_t = (t\_0+1)/(t+t\_0)  
%                1:  w\_t = ((t\_0+1)/(t+t\_0))^(0.75)  
%                2:  w\_t = ((t\_0+1)/(t+t\_0))^(5)  
%           param.averaging\_mode: (optional, false by default)  
%                0: no averaging  
%                1: first averaging mode for W2  
%                2: second averaging mode for W2  
%                WARNING: averaging can be very slow for sparse solutions  
%           param.determineEta (optional, automatically choose the parameters of the  
%             learning weights w\_t, true by default)   
%           param.t0 (optional, set up t0 for weights w\_t = ((1+t0)/(t+t0))^(alpha)  
%           param.numThreads (optional, number of threads)  
%           param.verbose (optional)  
%           param.seed (optional, choice of the random seed)  
%  
% Output:  W:  double dense p x nlambda matrix (contains the solution without averaging)  
%          W2:  double dense p x nlambda matrix (contains the solution with averaging)  
%  
% Author: Julien Mairal, 2013

The following piece of code illustrates how to use this function.

clear all;  
n=400000;  
p=1000;  
density=0.01;  
  
% generate random data  
format compact;  
randn('seed',0);  
rand('seed',0);  
X=sprandn(p,n,density);  
nrm=sqrt(sum(X.^2));  
X=X/mean(nrm);  
%mean\_nrm=mean();  
%X=X/mean\_nrm;  
  
% generate some true model  
z=double(sign(full(sprandn(p,1,0.05))));  
y=X'\*z;  
  
%%%%%%%%%%%%%%%%%%%%%%%%%%%%%%%%%%%%%  
% EXPERIMENT 1: Lasso  
%%%%%%%%%%%%%%%%%%%%%%%%%%%%%%%%%%%%%  
fprintf('EXPERIMENT FOR LASSO\n');  
nrm=sqrt(sum(y.^2));  
y=y+0.01\*nrm\*randn(n,1);    % add noise to the model  
nrm=sqrt(sum(y.^2));  
y=y\*(sqrt(n)/nrm);  
  
clear param;  
param.regul='l1';        % many other regularization functions are available  
param.loss='square';     % only square and log are available  
param.numThreads=-1;    % uses all possible cores  
param.normalized=false;  % if the columns of X have unit norm, set to true.  
param.averaging\_mode=0;  % no averaging, averaging was not really useful in experiments  
param.weighting\_mode=0;  % weights are in O(1/sqrt(n))  (see help mexStochasticProx)  
param.optimized\_solver=true;  % best is not to touch this option  
param.verbose=false;  
  
% set grid of lambda  
max\_lambda=max(abs(X\*y))/n;  
tablambda=max\_lambda\*(2^(1/4)).^(0:-1:-20);  % order from large to small  
param.lambda=tablambda;    % best to order from large to small  
tabepochs=[1 2 3 5 10];  % in this script, we compare the results obtained when varying the number of passes over the data.  
  
%% The problem which will be solved is  
%%   min\_beta  1/(2n) ||y-X' beta||\_2^2 + lambda ||beta||\_1  
fprintf('EXPERIMENT: ALL LAMBDAS IN PARALLEL\n');  
% we try different experiments when varying the number of epochs.  
% the problems for different lambdas are solve INDEPENDENTLY in parallel  
obj=[];  
objav=[];  
for ii=1:length(tabepochs)  
   param.iters=tabepochs(ii)\*n;  
   fprintf('EXP WITH %d PASS\n',tabepochs(ii));  
   nlambdas=length(param.lambda);  
   Beta0=zeros(p,nlambdas);  
   tic  
   [Beta tmp]=mexStochasticProx(y,X,Beta0,param);  
   toc  
   yR=repmat(y,[1 nlambdas]);  
   fprintf('Objective functions: \n');  
   obj=[obj; 0.5\*sum((yR-X'\*Beta).^2)/n+param.lambda.\*sum(abs(Beta))];  
   obj  
   if param.averaging\_mode  
      objav=[objav; 0.5\*sum((yR-X'\*tmp).^2)/n+param.lambda.\*sum(abs(tmp))];  
      objav  
   end  
   fprintf('Sparsity: \n');  
   sum(Beta ~= 0)  
end  
  
%%%%%%%%%%%%%%%%%%%%%%%%%%%%%%%%%%%%%  
% EXPERIMENT 2: L2 logistic regression   
%%%%%%%%%%%%%%%%%%%%%%%%%%%%%%%%%%%%%  
fprintf('EXPERIMENT FOR LOGISTIC REGRESSION + l2\n');  
y=sign(y);  
param.regul='l2';  
param.loss='logistic';  
obj=[];  
objav=[];  
for ii=1:length(tabepochs)  
   param.iters=tabepochs(ii)\*n;   % one pass over the data  
   fprintf('EXP WITH %d PASS\n',tabepochs(ii));  
   nlambdas=length(param.lambda);  
   Beta0=zeros(p,nlambdas);  
   tic  
   [Beta tmp]=mexStochasticProx(y,X,Beta0,param);  
   toc  
   yR=repmat(y,[1 nlambdas]);  
   fprintf('Objective functions: \n');  
   obj=[obj;sum(log(1.0+exp(-yR .\* (X'\*Beta))))/n+0.5\*param.lambda.\*sum(abs(Beta.^2))];  
   obj  
   if param.averaging\_mode  
      objav=[objav; sum(log(1.0+exp(-yR .\* (X'\*tmp))))/n+0.5\*param.lambda.\*sum(abs(tmp))];  
      objav  
   end  
end  
  
%%%%%%%%%%%%%%%%%%%%%%%%%%%%%%%%%%%%%  
% EXPERIMENT 3: L1 logistic regression   
%%%%%%%%%%%%%%%%%%%%%%%%%%%%%%%%%%%%%  
fprintf('EXPERIMENT FOR LOGISTIC REGRESSION + l1\n');  
y=sign(y);  
param.regul='l1';        % many other regularization functions are available  
param.loss='logistic';     % only square and log are available  
obj=[];  
objav=[];  
for ii=1:length(tabepochs)  
   param.iters=tabepochs(ii)\*n;   % one pass over the data  
   fprintf('EXP WITH %d PASS\n',tabepochs(ii));  
   nlambdas=length(param.lambda);  
   Beta0=zeros(p,nlambdas);  
   tic  
   [Beta tmp]=mexStochasticProx(y,X,Beta0,param);  
   toc  
   yR=repmat(y,[1 nlambdas]);  
   fprintf('Objective functions: \n');  
   obj=[obj; sum(log(1.0+exp(-yR .\* (X'\*Beta))))/n+param.lambda.\*sum(abs(Beta))];  
   obj  
   if param.averaging\_mode  
      objav=[objav; sum(log(1.0+exp(-yR .\* (X'\*tmp))))/n+param.lambda.\*sum(abs(tmp))];  
      objav  
   end  
   fprintf('Sparsity: \n');  
   sum(Beta ~= 0)  
end

---
