# Supplementary material for: Multitask fMRI Data Classification via Group-Wise Hybrid Temporal and Spatial Sparse Representations
Source: eNeuro. 2022 Jun 3;9(3):ENEURO.0478-21.2022. doi: 10.1523/ENEURO.0478-21.2022 (PMC9186416; doi:10.1523/ENEURO.0478-21.2022)
Supplement: Extended Data 1 — The code. Download Extended Data 1, ZIP file. [file enu-eN-MNT-0478-21-s02.zip › code/tools/spams-matlab.zip!/spams-matlab/doc/html/doc_spams007.html]

A few Functions for manipulating images


---

## 6  A few Functions for manipulating images

- Function mexExtractPatches
- Function mexCombinePatches
- Function mexConvFistaFlat

This functions are not well documented yet

### 6.1  Function mexExtractPatches

%   
% Usage:   X =mexExtractPatches(I,n,step);  
%  
% Name: mexExtractPatches  
%  
% Description: Extract n x n patches spaced every ``step'' pixels from   
% an image I of size nx x ny x nchannels  
%  
% Inputs: I:  double nx x ny x nchannels     
%  
% Output: X: double dense matrix  
%  
% Author: Julien Mairal, 2014

### 6.2  Function mexCombinePatches

%   
% Usage:   I =mexCombinePatches(X,I0,n,step,lambda,normalize);  
%  
% Name: mexCombinePatches  
%  
% Description: Combine patches extracted with mexExtractPatches into a new image   
%              I = lambda I0 + combined(X) if normalize, averaging is  
%              performed; otherwise the patches are just summed.  
%  
% Inputs: I0:  double nx x ny x nchannels     
%  
% Output: I: double nx x ny x nchannels   
%  
% Author: Julien Mairal, 2014

### 6.3  Function mexConvFistaFlat

%   
% Usage:   alpha =mexConvFistaFlat(I,D,alpha0,param);  
%  
% Name: mexConvFistaFlat  
%  
% Description: performs convolutional sparse encoding of an  
%              image I with a local dictionary D, using FISTA  
%              and similar options as mexFistaFlat  
%  
% Inputs: I:  double nx x ny x nchannels     
%         D:  dictionary    
%         alpha0: initial weights    
%  
% Output: alpha: output coefficients  
%  
% Author: Julien Mairal, 2014

---
