# Supplementary material for: Multitask fMRI Data Classification via Group-Wise Hybrid Temporal and Spatial Sparse Representations
Source: eNeuro. 2022 Jun 3;9(3):ENEURO.0478-21.2022. doi: 10.1523/ENEURO.0478-21.2022 (PMC9186416; doi:10.1523/ENEURO.0478-21.2022)
Supplement: Extended Data 1 — The code. Download Extended Data 1, ZIP file. [file enu-eN-MNT-0478-21-s02.zip › code/tools/spams-matlab.zip!/spams-matlab/doc/html/doc_spams008.html]

Miscellaneous Functions


---

## 7  Miscellaneous Functions

- Function mexConjGrad
- Function mexBayer
- Function mexCalcAAt
- Function mexCalcXAt
- Function mexCalcXY
- Function mexCalcXYt
- Function mexCalcXtY
- Function mexInvSym
- Function mexNormalize
- Function mexSort
- Function mexDisplayPatches
- Function mexCountPathsDAG
- Function mexRemoveCyclesGraph
- Function mexCountConnexComponents
- Function mexGraphOfGroupStruct
- Function mexGroupStructOfString
- Function mexReadGroupStruct
- Function mexTreeOfGroupStruct
- Function mexSimpleGroupTree

### 7.1  Function mexConjGrad

Implementation of a conjugate gradient for solving a linear system Ax=b
when A is positive definite. In some cases, it is faster than the Matlab
function `pcg`, especially when the library uses the Intel Math Kernel Library.

%   
% Usage:   x =mexConjGrad(A,b,x0,tol,itermax)  
%  
% Name: mexConjGrad  
%  
% Description: Conjugate gradient algorithm, sometimes faster than the   
%    equivalent Matlab function pcg. In order to solve Ax=b;  
%  
% Inputs: A:  double square n x n matrix. HAS TO BE POSITIVE DEFINITE  
%         b:  double vector of length n.  
%         x0: double vector of length n. (optional) initial guess.  
%         tol: (optional) tolerance.  
%         itermax: (optional) maximum number of iterations.  
%  
% Output: x: double vector of length n.  
%  
% Author: Julien Mairal, 2009

The following piece of code illustrates how to use this function.

A=randn(5000,500);  
A=A'\*A;  
b=ones(500,1);  
x0=b;  
tol=1e-4;  
itermax=0.5\*length(b);  
  
tic  
for ii = 1:20  
x1 = mexConjGrad(A,b,x0,tol,itermax);  
end  
t=toc;  
fprintf('mex-file time: %fs\n',t);  
  
tic  
for ii = 1:20  
x2 = pcg(A,b);  
end  
t=toc;  
fprintf('Matlab time: %fs\n',t);  
sum((x1(:)-x2(:)).^2)

### 7.2  Function mexBayer

Apply a Bayer pattern to an input image

%   
% Usage:   x =mexConjGrad(A,b,x0,tol,itermax)  
%  
% Name: mexConjGrad  
%  
% Description: Conjugate gradient algorithm, sometimes faster than the   
%    equivalent Matlab function pcg. In order to solve Ax=b;  
%  
% Inputs: A:  double square n x n matrix. HAS TO BE POSITIVE DEFINITE  
%         b:  double vector of length n.  
%         x0: double vector of length n. (optional) initial guess.  
%         tol: (optional) tolerance.  
%         itermax: (optional) maximum number of iterations.  
%  
% Output: x: double vector of length n.  
%  
% Author: Julien Mairal, 2009

The following piece of code illustrates how to use this function.

A=randn(5000,500);  
A=A'\*A;  
b=ones(500,1);  
x0=b;  
tol=1e-4;  
itermax=0.5\*length(b);  
  
tic  
for ii = 1:20  
x1 = mexConjGrad(A,b,x0,tol,itermax);  
end  
t=toc;  
fprintf('mex-file time: %fs\n',t);  
  
tic  
for ii = 1:20  
x2 = pcg(A,b);  
end  
t=toc;  
fprintf('Matlab time: %fs\n',t);  
sum((x1(:)-x2(:)).^2)

### 7.3  Function mexCalcAAt

For an input sparse matrix A, this function returns AA^T^. For some reasons, when A has a lot more columns than rows, this function can be much faster than the equivalent matlab command `X*A'`.

%   
% Usage:   AAt =mexCalcAAt(A);  
%  
% Name: mexCalcAAt  
%  
% Description: Compute efficiently AAt = A\*A', when A is sparse   
%   and has a lot more columns than rows. In some cases, it is  
%   up to 20 times faster than the equivalent Matlab expression  
%   AAt=A\*A';  
%  
% Inputs: A:  double sparse m x n matrix     
%  
% Output: AAt: double m x m matrix   
%  
% Author: Julien Mairal, 2009

The following piece of code illustrates how to use this function.

A=sprand(200,200000,0.05);  
  
tic  
AAt=mexCalcAAt(A);  
t=toc;  
fprintf('mex-file time: %fs\n',t);  
  
tic  
AAt2=A\*A';  
t=toc;  
fprintf('matlab time: %fs\n',t);  
  
sum((AAt(:)-AAt2(:)).^2)

### 7.4  Function mexCalcXAt

For an input sparse matrix A and a matrix X, this function returns XA^T^. For some reasons, when A has a lot more columns than rows, this function can be much faster than the equivalent matlab command `X*A'`.

%   
% Usage:   XAt =mexCalcXAt(X,A);  
%  
% Name: mexCalcXAt  
%  
% Description: Compute efficiently XAt = X\*A', when A is sparse and has a   
%   lot more columns than rows. In some cases, it is up to 20 times   
%   faster than the equivalent Matlab expression XAt=X\*A';  
%  
% Inputs: X:  double m x n matrix  
%         A:  double sparse p x n matrix     
%  
% Output: XAt: double m x p matrix   
%  
% Author: Julien Mairal, 2009

The following piece of code illustrates how to use this function.

X=randn(64,200000);  
A=sprand(200,200000,0.05);  
  
tic  
XAt=mexCalcXAt(X,A);  
t=toc;  
fprintf('mex-file time: %fs\n',t);  
  
tic  
XAt2=X\*A';  
t=toc;  
fprintf('mex-file time: %fs\n',t);  
  
sum((XAt(:)-XAt2(:)).^2)

### 7.5  Function mexCalcXY

For two input matrices X and Y, this function returns XY.

%   
% Usage:   Z =mexCalcXY(X,Y);  
%  
% Name: mexCalcXY  
%  
% Description: Compute Z=XY using the BLAS library used by SPAMS.  
%  
% Inputs: X:  double m x n matrix  
%         Y:  double n x p matrix     
%  
% Output: Z: double m x p matrix   
%  
% Author: Julien Mairal, 2009

The following piece of code illustrates how to use this function.

X=randn(64,200);  
Y=randn(200,20000);  
  
tic  
XY=mexCalcXY(X,Y);  
t=toc;  
fprintf('mex-file time: %fs\n',t);  
  
tic  
XY2=X\*Y;  
t=toc;  
fprintf('mex-file time: %fs\n',t);  
  
sum((XY(:)-XY2(:)).^2)

### 7.6  Function mexCalcXYt

For two input matrices X and Y, this function returns XY^T^.

%   
% Usage:   Z =mexCalcXYt(X,Y);  
%  
% Name: mexCalcXYt  
%  
% Description: Compute Z=XY' using the BLAS library used by SPAMS.  
%  
% Inputs: X:  double m x n matrix  
%         Y:  double p x n matrix     
%  
% Output: Z: double m x p matrix   
%  
% Author: Julien Mairal, 2009

The following piece of code illustrates how to use this function.

X=randn(64,200);  
Y=randn(200,20000)';  
  
tic  
XYt=mexCalcXYt(X,Y);  
t=toc;  
fprintf('mex-file time: %fs\n',t);  
  
  
tic  
XYt2=X\*Y';  
t=toc;  
fprintf('matlab-file time: %fs\n',t);  
  
sum((XYt(:)-XYt2(:)).^2)

### 7.7  Function mexCalcXtY

For two input matrices X and Y, this function returns X^T^Y.

%   
% Usage:   Z =mexCalcXtY(X,Y);  
%  
% Name: mexCalcXtY  
%  
% Description: Compute Z=X'Y using the BLAS library used by SPAMS.  
%  
% Inputs: X:  double n x m matrix  
%         Y:  double n x p matrix     
%  
% Output: Z: double m x p matrix   
%  
% Author: Julien Mairal, 2009

The following piece of code illustrates how to use this function.

X=randn(64,200)';  
Y=randn(200,20000);  
  
tic  
XtY=mexCalcXtY(X,Y);  
t=toc;  
fprintf('mex-file time: %fs\n',t);  
  
tic  
XtY2=X'\*Y;  
t=toc;  
fprintf('matlab-file time: %fs\n',t);  
  
sum((XtY(:)-XtY2(:)).^2)

### 7.8  Function mexInvSym

For an input symmetric matrices A in ℝ^n × n^, this function returns A^−1^.

%   
% Usage:   B =mexInvSym(A);  
%  
% Name: mexInvSym  
%  
% Description: returns the inverse of a symmetric matrix A  
%  
% Inputs: A:  double n x n matrix     
%  
% Output: B: double n x n matrix   
%  
% Author: Julien Mairal, 2009

The following piece of code illustrates how to use this function.

A=rand(1000,1000);  
A=A'\*A;  
  
tic  
B=mexInvSym(A);  
t=toc;  
fprintf('mex-file time: %fs\n',t);  
  
tic  
B2=inv(A);  
t=toc;  
fprintf('matlab-file time: %fs\n',t);  
  
sum((B(:)-B2(:)).^2)

### 7.9  Function mexNormalize

%   
% Usage:   Y =mexNormalize(X);  
%  
% Name: mexNormalize  
%  
% Description: rescale the columns of X so that they have  
%        unit l2-norm.  
%  
% Inputs: X:  double m x n matrix     
%  
% Output: Y: double m x n matrix   
%  
% Author: Julien Mairal, 2010

The following piece of code illustrates how to use this function.

X=rand(100,1000);  
  
tic  
Y=mexNormalize(X);  
t=toc;

### 7.10  Function mexSort

%   
% Usage:   Y =mexSort(X);  
%  
% Name: mexSort  
%  
% Description: sort the elements of X using quicksort  
%  
% Inputs: X:  double vector of size n  
%  
% Output: Y: double  vector of size n  
%  
% Author: Julien Mairal, 2010

The following piece of code illustrates how to use this function.

X=rand(1,300000);  
  
tic  
Y=mexSort(X);  
t=toc;  
toc  
  
tic  
Y2=sort(X,'ascend');  
t=toc;  
toc  
  
sum((Y2(:)-Y(:)).^2)

### 7.11  Function mexDisplayPatches

Print to the screen a matrix containing as columns image patches.

### 7.12  Function mexCountPathsDAG

This function counts the number of paths in a DAG using dynamic programming.

%   
% Usage:   num=mexCountPathsDAG(G);  
%  
% Name: mexCountPathsDAG  
%  
% Description: mexCountPathsDAG counts the number of paths   
%       in a DAG.  
%  
%       for a graph G with |V| nodes and |E| arcs,  
%       G is a double sparse adjacency matrix of size |V|x|V|,  
%       with |E| non-zero entries.  
%       (see example in test\_CountPathsDAG.m  
%  
%  
% Inputs: G:  double sparse |V| x |V| matrix (full graph)  
%  
% Output: num: number of paths  
%  
% Author: Julien Mairal, 2012

The following piece of code illustrates how to use this function.

% graph corresponding to figure 1 in arXiv:1204.4539v1  
fprintf('test mexCountPathsDAG\n');  
% this graph is a DAG  
G=[0 0 0 0 0 0 0 0 0 0 0 0 0;  
   1 0 0 0 0 0 0 0 0 0 0 0 0;  
   1 1 0 0 0 0 0 0 0 0 0 0 0;  
   1 1 0 0 0 0 0 0 0 0 0 0 0;  
   0 0 0 1 0 0 0 0 0 0 0 0 0;  
   0 1 1 0 1 0 0 0 0 0 0 0 0;  
   0 1 0 0 1 0 0 0 0 0 0 0 0;  
   0 0 0 0 0 1 1 0 0 0 0 0 0;  
   1 0 0 1 0 0 0 0 0 0 0 0 0;  
   1 0 0 0 0 0 0 0 1 0 0 0 0;  
   0 0 0 0 0 0 0 0 1 1 0 0 0;  
   0 0 0 0 0 0 0 0 1 0 1 0 0;  
   0 0 0 0 1 0 0 0 1 0 0 1 0];  
G=sparse(G);  
num=mexCountPathsDAG(G);  
fprintf('Num of paths: %d\n',num);

### 7.13  Function mexRemoveCyclesGraph

One heuristic to remove cycles from a graph.

%   
% Usage:   DAG=mexRemoveCycleGraph(G);  
%  
% Name: mexRemoveCycleGraph  
%  
% Description: mexRemoveCycleGraph heuristically removes  
%       arcs along cycles in the graph G to obtain a DAG.  
%       the arcs of G can have weights. The heuristic will  
%       remove in priority arcs with the smallest weights.  
%  
%       for a graph G with |V| nodes and |E| arcs,  
%       G is a double sparse adjacency matrix of size |V|x|V|,  
%       with |E| non-zero entries. The non-zero entries correspond  
%       to the weights of the arcs.  
%  
%       DAG is a also double sparse adjacency matrix of size |V|x|V|,  
%       but the graph is acyclic.  
%  
%       Note that another heuristic to obtain a DAG from a general   
%       graph consists of ordering the vertices.  
%  
% Inputs: G:  double sparse |V| x |V| matrix  
%  
% Output: DAG:  double sparse |V| x |V| matrix  
%  
% Author: Julien Mairal, 2012

The following piece of code illustrates how to use this function.

fprintf('test mexRemoveCyclesGraph\n');  
% this graph is not a DAG  
G=[0   0   0   0   0   0   0   0   0   0   0   0   0;  
   1   0   0   0.1 0   0   0   0.1 0   0   0   0   0;  
   1   1   0   0   0   0.1 0   0   0   0   0   0   0;  
   1   1   0   0   0   0   0   0   0   0   0   0.1 0;  
   0   0   0   1   0   0   0   0   0   0   0   0   0;  
   0   1   1   0   1   0   0   0   0   0   0   0   0;  
   0   1   0   0   1   0   0   0   0   0   0   0   0;  
   0   0   0   0   0   1   1   0   0   0   0   0   0;  
   1   0   0   1   0   0   0   0   0   0   0   0   0;  
   1   0   0   0   0   0   0   0   1   0   0   0   0;  
   0   0   0   0   0   0   0   0   1   1   0   0   0;  
   0   0   0   0   0   0   0   0   1   0   1   0   0;  
   0   0   0   0   1   0   0   0   1   0   0   1   0];  
G=sparse(G);  
DAG=mexRemoveCyclesGraph(G);  
format compact;  
fprintf('Original graph:\n');  
full(G)  
fprintf('New graph:\n');  
full(DAG)

### 7.14  Function mexCountConnexComponents

Count the number of connected components of a subgraph from a graph.

%   
% Usage:   num=mexCountConnexComponents(G,N);  
%  
% Name: mexCountConnexComponents  
%  
% Description: mexCountConnexComponents counts the number of connected  
%       components of the subgraph of G corresponding to set of nodes  
%       in N. In other words, the subgraph of G by removing from G all  
%       the nodes which are not in N.  
%  
%       for a graph G with |V| nodes and |E| arcs,  
%       G is a double sparse adjacency matrix of size |V|x|V|,  
%       with |E| non-zero entries.  
%       (see example in test\_CountConnexComponents.m)  
%       N is a dense vector of size |V|. if  N[i] is non-zero,  
%       it means that the node i is selected.  
%  
%  
% Inputs: G:  double sparse |V| x |V| matrix (full graph)  
%         N:  double full |V| vector.  
%  
% Output: num: number of connected components.  
%  
% Author: Julien Mairal, 2012

The following piece of code illustrates how to use this function.

% graph corresponding to figure 1 in arXiv:1204.4539v1  
fprintf('test mexCountConnexComponents\n');  
% this graph is a DAG  
G=[0 0 0 0 0 0 0 0 0 0 0 0 0;  
   1 0 0 0 0 0 0 0 0 0 0 0 0;  
   1 1 0 0 0 0 0 0 0 0 0 0 0;  
   1 1 0 0 0 0 0 0 0 0 0 0 0;  
   0 0 0 1 0 0 0 0 0 0 0 0 0;  
   0 1 1 0 1 0 0 0 0 0 0 0 0;  
   0 1 0 0 1 0 0 0 0 0 0 0 0;  
   0 0 0 0 0 1 1 0 0 0 0 0 0;  
   1 0 0 1 0 0 0 0 0 0 0 0 0;  
   1 0 0 0 0 0 0 0 1 0 0 0 0;  
   0 0 0 0 0 0 0 0 1 1 0 0 0;  
   0 0 0 0 0 0 0 0 1 0 1 0 0;  
   0 0 0 0 1 0 0 0 1 0 0 1 0];  
G=sparse(G);  
nodes=[0 1 1 0 0 1 0 0 1 0 1 1 0];  
num=mexCountConnexComponents(G,nodes);  
fprintf('Num of connected components: %d\n',num);  
  
% this graph is a not a DAG anymore. This function works  
% with general graphs.  
G=[0 0 0 0 0 0 0 0 0 0 0 0 0;  
   1 0 0 0 0 0 0 0 0 0 0 0 0;  
   1 1 0 1 0 0 0 0 0 0 0 0 0;  
   1 1 0 0 0 0 0 0 0 0 0 0 0;  
   0 0 0 1 0 0 0 0 0 0 0 0 0;  
   0 1 1 0 1 0 0 0 0 0 0 0 0;  
   0 1 0 0 1 0 0 0 0 0 0 0 0;  
   0 0 0 0 0 1 1 0 0 0 0 0 0;  
   1 0 0 1 0 0 0 0 0 0 0 0 0;  
   1 0 0 0 0 0 0 0 1 0 0 0 0;  
   0 0 0 0 0 0 0 0 1 1 0 0 0;  
   0 0 0 0 0 0 0 0 1 0 1 0 0;  
   0 0 0 0 1 0 0 0 1 0 0 1 0];  
nodes=[0 1 1 0 0 1 0 0 1 0 1 1 0];  
G=sparse(G);  
num=mexCountConnexComponents(G,nodes);  
fprintf('Num of connected components: %d\n',num);  
  
nodes=[0 1 1 1 0 1 0 0 1 0 1 1 0];  
num=mexCountConnexComponents(G,nodes);  
fprintf('Num of connected components: %d\n',num);

### 7.15  Function mexGraphOfGroupStruct

%   
% Usage:   groups =mexGraphOfGroupStruct(gstruct)  
%  
% Name: mexGraphOfGroupStruct  
%  
% Description: converts a group structure into the graph structure  
%    used by mexProximalGraph, mexFistaGraph or mexStructTrainDL  
%  
% Inputs: gstruct: the structure of groups as a cell array, one element per node  
%     an element is itself a 4 elements cell array:  
%       nodeid (>= 0), weight (double), array of vars associated to the node,  
%       array of children (nodeis's)  
% Output: graph: struct (see documentation of mexProximalGraph)  
%  
% Author: Jean-Paul CHIEZE, 2012

### 7.16  Function mexGroupStructOfString

%   
% Usage:   gstruct =mexGroupStructOfString(s)  
%  
% Name: mexGroupStructOfString  
%  
% Description: decode a multi-line string describing "simply" the structure of groups  
%    of variables needed by mexProximalGraph, mexProximalTree, mexFistaGraph,  
%    mexFistaTree and mexStructTrainDL and builds the corresponding group structure.  
%    Each line describes a group of variables as a node of a tree.  
%    It has up to 4 fields separated by spaces:  
%        node-id node-weight [variables-list] -> children-list  
%    Let's define Ng = number of groups, and Nv = number of variables.  
%    node-id must be in the range (0 - Ng-1), and there must be Ng nodes  
%    weight is a float  
%    variables-list : a space separated list of integers, maybe empty,  
%         but '[' and '] must be present. Numbers in the range (0 - Nv-1)  
%    children-list : a space separated list of node-id's  
%        If the list is empty, '->' may be omitted.  
%    The data must obey some rules :   
%        - A group contains the variables of the corresponding node and of the whole subtree.  
%        - Variables attached to a node are those that are not int the subtree.  
%        - If the data destination is a Graph, there may be several independant trees,  
%          and a varibale may appear in several trees.  
%    If the destination is a Tree, there must be only one tree, the root node  
%    must have id == 0 and each variable must appear only once.  
%  
% Inputs: s:  the multi-lines string  
%  
% Output: groups: cell array, one element for each node  
%                an element is itsel a 4 elements cell array:  
%                 nodeid (int >= 0), weight (double), array of vars of the node,  
%                array of children (nodeid's)  
%  
% Author: Jean-Paul CHIEZE, 2012

### 7.17  Function mexReadGroupStruct

%   
% Usage:   gstruct =mexReadGroupStruct(file)  
%  
% Name: mexReadGroupStruct  
%  
% Description: reads a text file describing "simply" the structure of groups  
%    of variables needed by mexProximalGraph, mexProximalTree, mexFistaGraph,  
%    mexFistaTree and mexStructTrainDL and builds the corresponding group structure.  
%    weight is a float  
%    variables-list : a space separated list of integers, maybe empty,  
%        but '[' and '] must be present. Numbers in the range (0 - Nv-1)  
%    children-list : a space separated list of node-id's  
%        If the list is empty, '->' may be omitted.  
%    The data must obey some rules :   
%        - A group contains the variables of the corresponding node and of the whole subtree.  
%        - Variables attached to a node are those that are not int the subtree.  
%        - If the data destination is a Graph, there may be several independant trees,  
%           and a varibale may appear in several trees.  
%    If the destination is a Tree, there must be only one tree, the root node  
%        must have id == 0 and each variable must appear only once.  
%  
% Inputs: file:  the file name  
%  
% Output: groups: cell array, one element for each node  
%                an element is itsel a 4 elements cell array:  
%                nodeid (int >= 0), weight (double), array of vars of the node,  
%                array of children (nodeid's)  
%  
% Author: Jean-Paul CHIEZE, 2012

### 7.18  Function mexTreeOfGroupStruct

%   
% Usage:   [permutations groups nbvars] =mexTreeOfGroupStruct(gstruct)  
%  
% Name: mexTreeOfGroupStruct  
%  
% Description: converts a group structure into the tree structure  
%    used by mexProximalTree, mexFistaTree or mexStructTrainDL  
%  
% Inputs: gstruct: the structure of groups as a cell array, one element per node  
%     an element is itself a 4 lements cell array:  
%       nodeid (>= 0), weight (double), array of vars associated to the node,  
%       array of children (nodeis's)  
% Output: permutations: permutation vector that must be applied to the result of the  
%               programm using the tree. Empty if no permutation is needed.  
%      tree: struct (see documentation of mexProximalTree)  
%      nbvars : number of variables in the tree  
%  
% Author: Jean-Paul CHIEZE, 2012

### 7.19  Function mexSimpleGroupTree

%   
% Usage:   gstruct =mexSimpleGroupTree(degrees)  
%  
% Name: mexSimpleGroupTree  
%  
% Description: makes a structure representing a tree given the  
%   degree of each level.  
%  
% Inputs: degrees:  int vector; degrees(i) is the number of children of each node at level i  
%  
% Output: group\_struct: cell array, one element for each node  
%                an element is itsel a 4 elements cell array :  
%                 nodeid (int >= 0), weight (double), array of vars attached to the node  
%                  (here equal to [nodeid]), array of children (nodeid's)  
%  
% Author: Jean-Paul CHIEZE, 2012

---
