# Supplementary material for: Multitask fMRI Data Classification via Group-Wise Hybrid Temporal and Spatial Sparse Representations
Source: eNeuro. 2022 Jun 3;9(3):ENEURO.0478-21.2022. doi: 10.1523/ENEURO.0478-21.2022 (PMC9186416; doi:10.1523/ENEURO.0478-21.2022)
Supplement: Extended Data 1 — The code. Download Extended Data 1, ZIP file. [file enu-eN-MNT-0478-21-s02.zip › code/tools/spams-matlab.zip!/spams-matlab/doc/html/doc_spams009.html]

Duality Gaps with Fenchel Duality


---

## A  Duality Gaps with Fenchel Duality

This section is taken from the appendix D of Julien Mairal’s PhD thesis [19].
We are going to use intensively Fenchel Duality (see [2]).
Let us consider the problem

|  |  |  |  |  |
| --- | --- | --- | --- | --- |
|  | min; w ∈ ℝ^p^ | [g(w) | ▵; = | f(w) + λψ(w)],     (47) |

We first notice that for all the formulations we have been
interested in, g(w) can be rewritten

g(w) = f(X^⊤^w) + λψ(w), 
    (48)

where X=[x^1^,…,x^n^] are training vectors, and f is an
appropriated smooth real-valued function of ℝ^n^,
and ψ one of the regularization functions we have introduced.

Given a primal variable w in ℝ^p^ and a dual variable κ in
ℝ^n^, we obtain using classical Fenchel duality rules [2],
that the following quantity is a duality gap for problem (47):

|  |  |  |
| --- | --- | --- |
| δ(w,κ) | ▵; = | g(w) + f^∗^(κ) + λψ^∗^(−Xκ / λ), |

where f^∗^ and ψ^∗^ are respectively the Fenchel conjugates
of f and ψ. Denoting by w^⋆^ the solution of
Eq. (47), the duality gap is interesting in the sense that
it upperbounds the difference with the optimal value of the function:

δ(w,κ) ≥  g(w)−g(w^⋆^) ≥ 0.

Similarly, we will consider pairs of primal-dual variables (W,K) when
dealing with matrices.

During the optimization, sequences of primal variables w are available,
and one wishes to exploit duality gaps for estimating the difference
g(w)−g(w^⋆^). This requires the following components:

- being able to efficiently compute f^∗^ and ψ^∗^.
- being able to obtain a “good” dual variable κ given a primal
  variable w, such that δ(w,κ) is close to
  g(w)−g(w^⋆^).

We suppose that the first point is satisfied (we will detail these computations
for every loss and regularization functions in the sequel), and explain how to
choose κ in general (details will also be given in the sequel).

Let us first consider the choice that associates with a primal variable w, the
dual variable

|  |  |  |
| --- | --- | --- |
| κ(w) | ▵; = | ∇ f(X^⊤^w),      (49) |

and let us compute δ(w,κ(w)).
First, easy computations show that for all vectors z in ℝ^n^,
f^∗^(∇f(z)) = z^⊤^∇f(z)−f(z),
which gives

|  |  |  |  |
| --- | --- | --- | --- |
| δ(w,κ(w)) | = | f(X^⊤^w) + λ ψ(w) + f^∗^(∇f(X^⊤^w)) + λψ^∗^(−X∇f(X^⊤^w)/ λ), | (50) |
|  | = | λ ψ(w) + w^⊤^X∇f(X^⊤^w) + λψ^∗^(−X∇f(X^⊤^w)/ λ). | (51) |

We now use the classical Fenchel-Young inequality (see, Proposition
3.3.4 of [2]) on the function ψ, which gives

δ(w,κ(w))  ≥  w^⊤^X∇f(X^⊤^w) − w^⊤^X∇f(X^⊤^w) = 0,

with equality if and only if −X∇f(X^⊤^w) belongs to
∂ ψ(w). Interestingly, we now that first-order optimality
conditions for Eq. (48) gives that
−X∇f(X^⊤^w^⋆^) ∈ ∂ ψ(w^⋆^).
We have now in hand a non-negative function w ↦ δ(w,κ(w)) of w, that
upperbounds g(w)−g(w^⋆^) and satisfying δ(w^⋆^,κ(w^⋆^))=0.

This is however not a sufficient property to make it a good measure
of the quality of the optimization, and further work is required,
that will be dependent on f and ψ.
We have indeed proven that δ(w^⋆^,κ(w^⋆^)) is always 0. However,
for w different than w^⋆^, δ(w^⋆^,κ(w^⋆^)) can be infinite,
making it a non-informative duality-gap. The reasons for this can be one of the following:

- The term ψ^∗^(−X∇f(X^⊤^w)/ λ) might have an infinite value.
- Intercepts make the problem more complicated. One can write the formulation with an intercept by
  adding a row to X filled with the value 1, add one dimension to the vector w, and consider
  a regularization function ψ that does regularize the last entry of
  w. This further complexifies the computation of ψ^∗^ and its
  definition, as shown in the next section.

Let us now detail how we proceed to solve these problems, but first without considering the intercept.
The analysis is similar when working with matrices W instead of vectors w.

#### A.0.1  Duality Gaps without Intercepts

Let us show how to compute the Fenchel conjugate of the functions we have introduced.
We now present the Fenchel conjugate of the loss functions f.

- The square loss

  |  |  |  |
  | --- | --- | --- |
  | f(z)= | 1; 2n | \|\|y−z\|\|_2_^2^, |

  |  |  |  |
  | --- | --- | --- |
  | f^∗^(κ)= | n; 2 | \|\|κ\|\|_2_^2^ + κ^⊤^y. |
- The logistic loss

  |  |  |  |  |
  | --- | --- | --- | --- |
  | f(z)= | 1; n | n; ∑; i=1 | log(1+e^−y_i_z_i_^) |

  f^∗^(κ)=

  ⎧  
  ⎪  
  ⎪  
  ⎨  
  ⎪  
  ⎪  
  ⎩

  +∞  if  ∃ i∈ [ 1;n ]   s.t.   y_i_κ_i_ ∉ [−1,0],

  n

  ∑

  i=1

  (1+y_i_κ_i_)log(1+y_i_κ_i_)−y_i_κ_i_log(−y_i_κ_i_)  otherwise.
- The multiclass logistic loss (or softmax). The primal variable is now a matrix Z, in ℝ^n × r^, which represents the product X^⊤^W. We denote by K the dual variable in ℝ^n × r^.

  |  |  |  |  |  |  |  |  |
  | --- | --- | --- | --- | --- | --- | --- | --- |
  | f(Z)= | 1; n | n; ∑; i=1 | log | ⎛  ⎜  ⎝ | r; ∑; j=1 | e ^Z_ij_ − Z_iyi_^ | ⎞  ⎟  ⎠ |

  f^∗^(K)=

  ⎧  
  ⎪  
  ⎪  
  ⎨  
  ⎪  
  ⎪  
  ⎩

  +∞  if  ∃ i ∈[ 1;n ]   s.t.   { K_ij_ < 0  and  j ≠ y_i_ }  or  K_iyi_ < −1,

  |  |  |  |  |  |  |  |  |
  | --- | --- | --- | --- | --- | --- | --- | --- |
  |  | n; ∑; i=1 |  | ⎡  ⎢  ⎣ | ∑; j ≠ y_i_ | K_ij_log(K_ij_) + (1+K_i yi_)log(1+K_i yi_) | ⎤  ⎥  ⎦ | . |

Our first remark is that the choice Eq. (49) ensures
that f(κ) is not infinite.

As for the regularization function, except for the Tikhonov regularization
which is self-conjugate (it is equal to its Fenchel conjugate), we have
considered functions that are norms. There exists therefore a norm ||.|| such
that ψ(w)=||w||, and we denote by ||.||_∗_ its dual-norm. In such a
case, the Fenchel conjugate of ψ for a vector γ in ℝ^p^ takes the
form

ψ^∗^(γ) =

⎧  
⎨  
⎩

|  |  |
| --- | --- |
| 0 | if  \|\|γ\|\|_∗_≤ 1, |
| +∞ | otherwise. |

It turns out that for almost all the norms we have presented, there exists (i)
either a closed form for the dual-norm or (ii) there exists an
efficient algorithm evaluating it. The only one which does not conform to this
statement is the tree-structured sum of ℓ_2_-norms, for which we do not know
how to evaluate it efficiently.

One can now slightly modify the definition of κ
to ensure that ψ^∗^(−Xκ/λ) ≠
+∞. A natural choice is

|  |  |  |  |  |  |  |  |
| --- | --- | --- | --- | --- | --- | --- | --- |
| κ(w) | ▵; = | min | ⎛  ⎜  ⎝ | 1, | λ; \|\|X∇f(X^⊤^w)\|\|_∗_ | ⎞  ⎟  ⎠ | ∇ f(X^⊤^w), |

which is the one we have implemented. With this new choice, it is easy to see
that for all vectors w in ℝ^p^, we still have f^∗^(κ) ≠ + ∞, and
finally, we also have δ(w,κ(w)) < + ∞ and
δ(w^⋆^,κ(w^⋆^))=0, making it potentially a good
duality gap.

#### A.0.2  Duality Gaps with Intercepts

Even though adding an intercept does seem a simple modification to the original
problem, it induces difficulties for finding good dual variables.

We recall that having an intercept is equivalent to having a problem of the
type (48), by adding a row to X filled with the value
1, adding one dimension to the vector w (or one row for matrices W),
and by using a regularization function that does not depend on the last entry
of w (or the last row of W).

Suppose that we are considering a problem of
type (48) of dimension p+1, but we are using a
regularization function ψ: ℝ^p+1^ → ℝ, such that for a
vector w in ℝ^p+1^, ψ(w) =^▵^ ψ(w_[ 1;p ]_),
where ψ: ℝ^p^ → ℝ is one of the regularization function we have
introduced. Then, considering a primal variable w, a dual variable κ,
and writing γ=^▵^−Xκ/λ, we are interested in computing

ψ^∗^(γ) =

⎧  
⎨  
⎩

+∞  if  γ_p+1_ ≠ 0

ψ^∗^(γ_[ 1;p ]_)  otherwise,

which means that in order the duality gap not to be infinite, one needs in addition to ensure
that γ_p+1_ be zero. Since the last row of X is filled with ones, this writes
down ∑_i=1_^p+1^ κ_i_=0.
For the formulation with matrices W and K, the constraint is similar but for every
column of K.

Let us now detail how we proceed for every loss function to find a “good”
dual variable κ satisfying this additional constraint, given a primal
variable w in ℝ^p+1^, we first define the auxiliary function

|  |  |  |
| --- | --- | --- |
| κ′(w) | ▵; = | ∇f(X^⊤^w), |

(which becomes K′(W)=^▵^ ∇f(X^⊤^W) for matrices),
and then define another auxiliary function κ″(w) as follows,
to take into account the additional constraint ∑_i=1_^p+1^ κ_i_=0.

- For the square loss, we define another auxiliary function:

  |  |  |  |  |  |
  | --- | --- | --- | --- | --- |
  | κ″(w) | ▵; = | κ′(w) − | 1; n | 1_p+1_^⊤^κ′(w)1_p+1_ |

  where 1_p+1_ is a vector of size p+1 filled with ones. This step,
  ensures that ∑_i=1_^p+1^κ″(w)_i_= 0.
- For the logistic loss, the situation is slightly more complicated since additional constraints are involved in the definition of f^∗^.

  |  |  |  |  |  |
  | --- | --- | --- | --- | --- |
  | κ″(w) | ▵; = | arg min_κ ∈ ℝ^n^_ \|\|κ−κ′(w)\|\|_2_^2^   s.t. | n; ∑; i=1 | κ_i_=0  and  ∀ i ∈ [ 1;n ], κ_i_ ∈ [−1,0]. |

  This problem can be solved in linear-time [3]
  using a similar algorithm as for the projection onto the ℓ_1_-ball,
  since it is an instance of a *quadratic knapsack problem*.
- For the multi-class logistic loss, we proceed in a similar way, for every column K^j^ of K, j ∈ [ 1;r ]:

  |  |  |  |  |  |
  | --- | --- | --- | --- | --- |
  | K^′′ j^(w) | ▵; = | arg min_κ ∈ ℝ^n^_ \|\|K^′ j^−κ′(w)\|\|_2_^2^   s.t. | n; ∑; i=1 | κ_i_=0  and |

  ∀ i ∈ [ 1;n ], {κ_i_ ≥ 0  if  j ≠ y_i_}  and  {κ_i_ ≥ −1  if  y_i_=j}.

When the function ψ is the Tykhonov regularization function, we end the process by setting κ(w)=κ″(w).
When it is a norm, we choose, as before for taking into account the constraint ||Xκ||_∗_≤ λ,

|  |  |  |  |  |  |  |  |
| --- | --- | --- | --- | --- | --- | --- | --- |
| κ(w) | ▵; = | min | ⎛  ⎜  ⎝ | 1, | λ; \|\|Xκ″(w)\|\|_∗_ | ⎞  ⎟  ⎠ | κ″(w), |

with a similar formulation for matrices W and K.

Even though finding dual variables while taking into account the intercept
requires quite a lot of engineering, notably implementing a quadratic knapsack
solver, it can be done efficiently.

---
