# Supplementary material for: Multitask fMRI Data Classification via Group-Wise Hybrid Temporal and Spatial Sparse Representations
Source: eNeuro. 2022 Jun 3;9(3):ENEURO.0478-21.2022. doi: 10.1523/ENEURO.0478-21.2022 (PMC9186416; doi:10.1523/ENEURO.0478-21.2022)
Supplement: Extended Data 1 — The code. Download Extended Data 1, ZIP file. [file enu-eN-MNT-0478-21-s02.zip › code/tools/spams-matlab.zip!/spams-matlab/doc/html/index.html]

SPAMS: a SPArse Modeling Software, v2.5


# SPAMS: a SPArse Modeling Software, v2.5

### Julien Mairal julien.mairal@m4x.org   Python and R interfaces by Jean-Paul Chieze   Archetypal Analysis by Yuansi Chen   and the output of numerous collaborations with Rodolphe Jenatton, Francis Bach, Jean Ponce, Guillaume Obozinski, Bin Yu, Guillermo Sapiro, and Zaid Harchaoui.

- Contents
- Introduction
- Installation
- Dictionary Learning and Matrix Factorization Toolbox
  - Function mexTrainDL
  - Function mexTrainDL\_Memory
  - Function mexStructTrainDL
  - Function nmf
  - Function nnsc
  - Function mexArchetypalAnalysis
- Sparse Decomposition Toolbox
  - Function mexOMP
  - Function mexOMPMask
  - Function mexRidgeRegression
  - Function mexLasso
  - Function mexLassoWeighted
  - Function mexLassoMask
  - Function mexCD
  - Function mexSOMP
  - Function mexL1L2BCD
  - Function mexSparseProject
  - Function mexDecompSimplex
- Proximal Toolbox
  - Regularization Functions
  - Function mexProximalFlat
  - Function mexProximalTree
  - Function mexProximalGraph
  - Function mexProximalPathCoding
  - Function mexEvalPathCoding
  - Problems Addressed
  - Function mexFistaFlat
  - Function mexFistaTree
  - Function mexFistaGraph
  - Function mexFistaPathCoding
  - Function solverPoisson
  - Function mexIncrementalProx
  - Function mexStochasticProx
- A few Functions for manipulating images
  - Function mexExtractPatches
  - Function mexCombinePatches
  - Function mexConvFistaFlat
- Miscellaneous Functions
  - Function mexConjGrad
  - Function mexBayer
  - Function mexCalcAAt
  - Function mexCalcXAt
  - Function mexCalcXY
  - Function mexCalcXYt
  - Function mexCalcXtY
  - Function mexInvSym
  - Function mexNormalize
  - Function mexSort
  - Function mexDisplayPatches
  - Function mexCountPathsDAG
  - Function mexRemoveCyclesGraph
  - Function mexCountConnexComponents
  - Function mexGraphOfGroupStruct
  - Function mexGroupStructOfString
  - Function mexReadGroupStruct
  - Function mexTreeOfGroupStruct
  - Function mexSimpleGroupTree
- Duality Gaps with Fenchel Duality
- References


---

> *This document was translated from L^A^T_E_X by* *H^E^V^E^A**.*
